# Supplementary material for: Photometallobiocatalytic Asymmetric Radical‐Mediated Cross‐Coupling of Organotrifluoroborate Salts and Pyridotriazoles
Source: Angew Chem Int Ed Engl. 2026 Aug 22;65(42):e7251439. doi: 10.1002/anie.7251439 (PMC13653585; doi:10.1002/anie.7251439)

Supporting Information for

Photometallobiocatalytic Asymmetric Radical-Mediated Cross-Coupling of Organotrifluoroborate Salts and Pyridotriazoles

Huanan Wang[1], Binh Khanh Mai[2], Xingjie Zhang[1], Chongtao Li[1], Peng Liu[2] and Yang Yang[1,2,3,4]*

[1]Department of Chemistry and Biochemistry, University of California Santa Barbara, Santa Barbara, California 93106, United States

[2]Department of Chemistry, University of Pittsburgh, Pittsburgh, Pennsylvania 15260, United States

[2]Department of Bioengineering, University of California Santa Barbara, Santa Barbara, California 93106, United States

[3]Biomolecular Science and Engineering (BMSE) Program, University of California Santa Barbara, Santa Barbara, California 93106, United States

[4]Howard Hughes Medical Institute, University of California Santa Barbara, Santa Barbara, California 93106, United States

*Corresponding author. E-mail: yang@chem.ucsb.edu

Table of Contents

I. General methods 3

II. General experimental procedure 10

III. Selected results for reaction development. 12

IV. DNA and protein sequences 17

V. Kinetic and fluorescence anisotropy titration data 21

VI. Synthesis and characterization of substrates 34

VII. Synthesis and characterization of products 35

VIII. GC-MS calibration curves of products 49

IX. HPLC traces of products 61

X. Computational studies 84

XI. References 96

XII. ^{1}H, ^{13}C and ^{19}F spectra of compounds 101

I. General methods

General. Unless otherwise noted, all chemicals and reagents were obtained from commercial suppliers (Sigma-Aldrich, VWR, Ambeed, Combi-Blocks, Oakwood, Chemscene, Thermo Fisher) and used without further purification. THF, toluene, DMF, diisopropylamine, *n*-BuLi (2.5 M in hexanes) were obtained from Sigma Aldrich (SureSeal). Silica gel chromatography was performed using SiliaFlash® Irregular Silica Gel, P60 40–63 µm, 60 Å, from SiliCycle. ^{1}H and ^{13}C NMR spectra were recorded on a Bruker 400 MHz instrument in $CDCl_3$ or DMSO-d_6 and referenced to residual solvent signals. ^{1}H NMR data are reported as follows: chemical shift (δ ppm), multiplicity (s = singlet, d = doublet, t = triplet, q = quartet, p = pentet, sext = sextet, hept = heptet, m = multiplet, dd = doublet of doublets, ddd = doublet of doublet of doublets, dt = doublet of triplets), coupling constant (Hz), and integration. ^{19}F NMR (^{1}H decoupled) spectra (where applicable) were recorded on a Bruker 400 MHz instrument and referenced to $CFCl_3$ as an external standard. Small scale *E. coli* cell lysis was performed via sonication using a Qsonica Q500 sonicator equipped with a standard QSonica 24-tip horn (Part #4579). If fewer than 24 samples are present, unused tips are submerged in water during sonication. *E. coli* cell lysis on a large scale was performed via sonication using a Qsonica Q500 or Q700 sonicator equipped with a standard probe (tip diameter = ½’’). Optical rotation was recorded on a Rudolph Research Laboratory AUTOPOL IV polarimeter. High-resolution mass spectrometry (HRMS) data were obtained at the University of California Santa Barbara Mass Spectrometry Facility. High-resolution accurate mass (HRAS) EI data was acquired using a Waters Micromass LCT Premier time-of-flight (TOF) mass spectrometer. Masses of positively charged ions were calibrated using methanol solutions of polyethylene glycol or polyethylene glycol monomethyl ether as an internal standard. Masses of negatively charged ions were calibrated using aqueous sodium formate or sodium/cesium iodide as an internal standard as appropriate. All samples were dissolved in methanol and were directly infused unless otherwise noted. Synthetic reactions were monitored by thin layer chromatography (TLC, SilicaPlate G TLC Glass-Backed Silica, 250 µm, 20 x 20 cm, F254, catalog number: TLG-R10011B-323) using a UV lamp or an appropriate TLC staining agent for visualization. All biocatalytic reactions were conducted in clear 1-dram or 2-dram glass vial from VWR (catalog number: 66011-041 and 14-959-35C, respectively) with magnetic stirring (500 rpm) using a Heidolph stir plate. A Kessil LED PR160L-525 nm (max 45 W) was used as the visible light source.

E. coli cells were grown in Luria-Bertani (LB) medium or Hyperbroth (HB) (AthenaES, Lot# 0107-27118) with 0.1 mg/mL ampicillin (amp) (media annotated as LB_{amp} or HB_{amp}). Primer sequences for site-saturation mutagenesis are provided below. T5 exonuclease, Phusion DNA polymerase, and *Taq* DNA ligase were purchased from New England Biolabs (NEB, Ipswich, MA). Potassium Phosphate Buffer (abbreviated as KPi buffer) was used as the buffering system for protein storage unless otherwise noted.

Chromatography. Analytical chiral HPLC was conducted using a Shimadzu i-series (66 MPa) instrument with HPLC-grade hexanes and isopropanol (Sigma Aldrich) as the mobile phase. Enantiomers were separated using one of the following chiral columns: Chiralpak IA (4.6 mm × 25 cm, 5 micron), Chiralpak IB-N5 (4.6 mm × 25 cm, 5 micron), Chiralpak IC (4.6 mm × 25 cm, 5 micron), Chiralpak IG (4.6 mm × 25 cm, 5 micron), Chiralpak IH (4.6 mm × 25 cm, 5 micron), Chiralpak OD-H (4.6 mm × 25 cm, 5 micron) and Chiralpak OJ-H (4.6 mm × 25 cm, 5 micron). Chiral GC was conducted using a Shimadzu GC-2030 GC system equipped with an FID detector and with an Agilent CycloSil-B column (30 m × 0.32 mm, 0.25 µm film). Gas chromatography-mass spectrometry (GC-MS) analyses were carried out using a Shimadzu GCMS-QP2020NX system with a GC-2030 front end and a J&W HP-5ms column (30 m × 0.25 mm, 0.25 µm film).

Cloning and mutagenesis. Site-saturation mutagenesis (SSM) and screening were used for directed evolution. The plasmid pET-22b(+) was used as the cloning and expression vector for heme proteins, including various cytochromes P450, globins and cytochromes *c* described in this study. The cytochrome *c* maturation plasmid pEC86[1] was used as part of a two-plasmid system to express prokaryotic cytochrome *c* proteins. Genes of metalloproteins were codon optimized using *E coli*. as the host organism and purchased as gBlocks from GeneralBio as plasmids in a desired vector. Heme proteins described in this study were cloned into pET-22b(+) between *Nde* I and *Xho* I with a C-terminal 6X His-tag. Single site-saturation mutagenesis was performed using the "22c-trick" method as described by Kille et al.[2] PCR products were digested with DpnI, gel purified, and ligated using a Gibson mix prepared from 5X isothermal (ISO) reaction buffer (25% PEG-8000, 500 mM Tris-HCl pH 7.5, 50 mM $MgCl_2$, 50 mM DTT, 1 mM each of the dNTPs, and 5 mM NAD), T5 exonuclease, Phusion DNA polymerase, and *Taq* DNA ligase. The ligation mixture was used directly to transform electrocompetent *E. coli* strain *E. cloni* BL21(DE3) cells (Lucigen).

Expression of *Ape*Pgb variants in 24-well plates. Single colonies from LB_{amp} agar plates were picked using sterile toothpicks and cultured in deep-well 96-well plates (Greiner catalog number: 780270-FD; total volume per well of this plate: 2.2 mL) containing LB_{amp} (400 µL/well) at 37 °C and 230 rpm for 10-12 h. The HB_{amp} (4 mL/well) medium in a 24-well plate was then inoculated with an aliquot (200 µL/well, 5% v:v) of overnight culture from the 96-well plate. Each 96-well plate was used to inoculate a total of four 24-well plate. The 24-well plates for protein expression were allowed to shake at 37 °C and 250 rpm for 2.5 h. The plates were cooled on ice for 20 min and the cultures were induced with 0.50 mM isopropyl *β*-D-1-thiogalactopyranoside (IPTG, final concentration) and 1.0 mM 5-aminolevulinic acid (ALA, final concentration). Protein expression was conducted at 20 °C and 200 rpm for 20 h.

Reaction screening in a 96-well plate format. *E. coli* (*E. cloni* BL21(DE3)) cells in 24-well plates were pelleted (4,000 *g*, 4 min, 4 °C) using an Eppendorf tabletop centrifuge 5910R and then resuspended in KPi buffer (1.0 mL/well) by gentle shaking using a Fisher Scientific microplate shaker (800 rpm, 3–5 min). The cell suspension was lysed by sonication using a Qsonica Q500 sonicator equipped with a 24-tip horn. To lyse the cell suspensions in a 24-well plate, four iterations of sonication using this 24-tip horn were carried out. Cell lysis conditions: 45% amplitude, 2 secs on, 4 secs off, 6 min in total (sonication time per cycle: 1 min). To pellet the cell debris, lysates were centrifuged using an Eppendorf 5910R tabletop centrifuge (4,000 *g*, 20 min, 4 °C). Using an Eppendorf Xplorer 12-channel electronic pipette (50–1000 µL), the clarified cell-free lysates (750 µL per well) were then transferred to shell vials (1 mL) in a Paradox 96-position reactor purchased from Analytical Sales and then transferred into an anaerobic chamber for reaction setup.

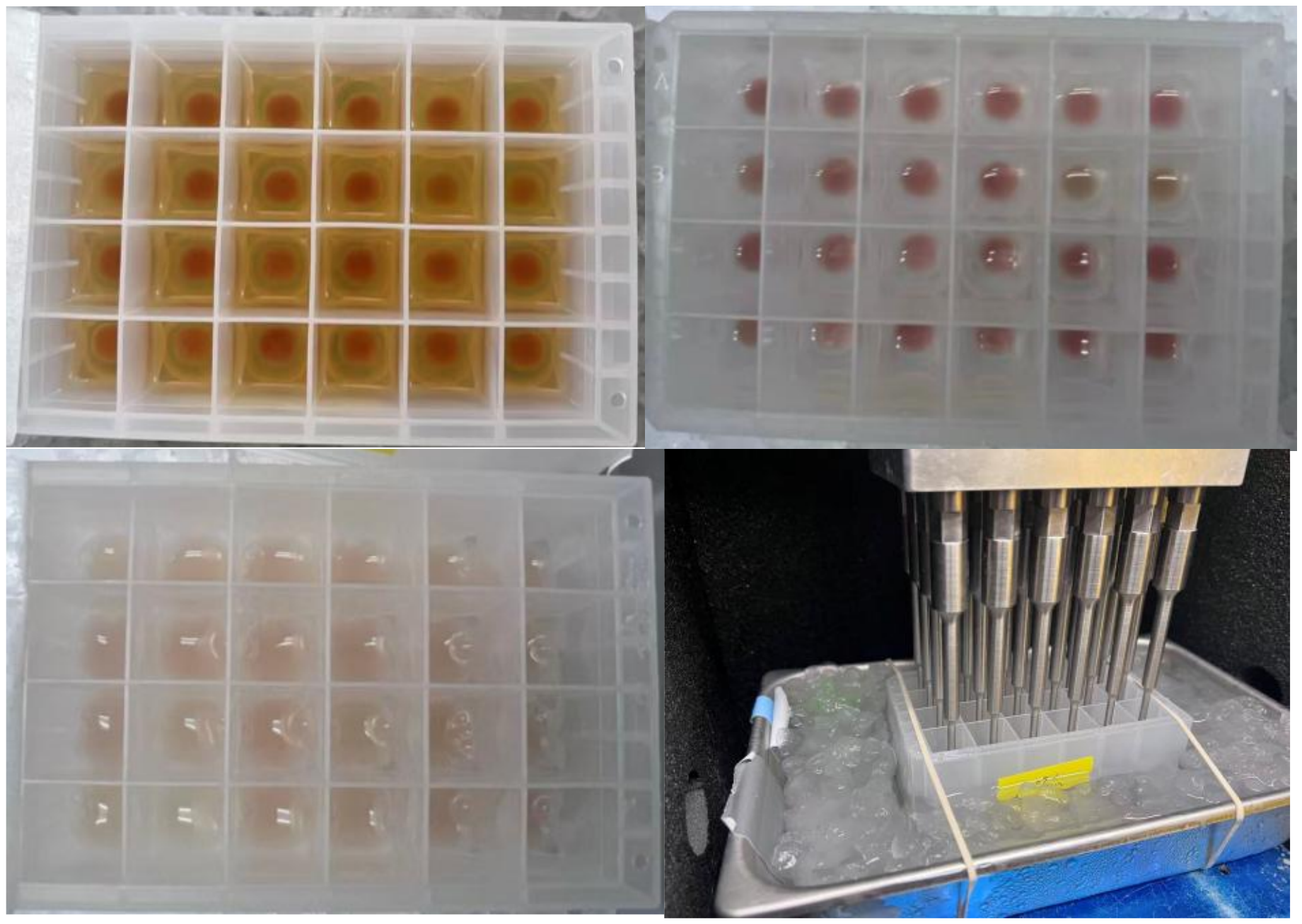

Figure S1. Experimental setup for high-throughput sonication using a 24-tip horn.

In an anaerobic chamber, the following stock solutions were prepared: photocatalyst (20 mM in degassed DMSO), organotrifluoroborate substrate (100 mM in degassed DMSO), pyridotriazole substrate (200 mM in degassed DMSO). A total of 96 shell vials were placed in a 96-position Paradox reactor. To each shell vial were added 20 μL organotrifluoroborate salt stock solution, 20 μL pyridotriazole stock solution, and 10 μL photocatalyst stock solution using a 0.5–10 μL Eppendorf Xplorer 12-channel pipette. The total reaction volume was 800 μL; the final concentrations of each reaction component were as follows: 2.5 mM organotrifluoroborate salt, 5.0 mM pyridotriazole, and 0.25 mM photocatalyst. The 96-position Paradox reactor was illuminated with a 530 nm Lumidox 96-position LED array purchased from Analytical Sales (power output: 40 mW) and gently shaken on a Corning digital microplate shaker at room temperature and 380 rpm for 4 h. Upon the completion of reaction, the 96-position Paradox reactor was taken out of the anaerobic chamber and the reactions were worked up and analyzed by chiral HPLC, chiral GC or GC-MS by following the procedure detailed below. A representative experimental setup is shown below.

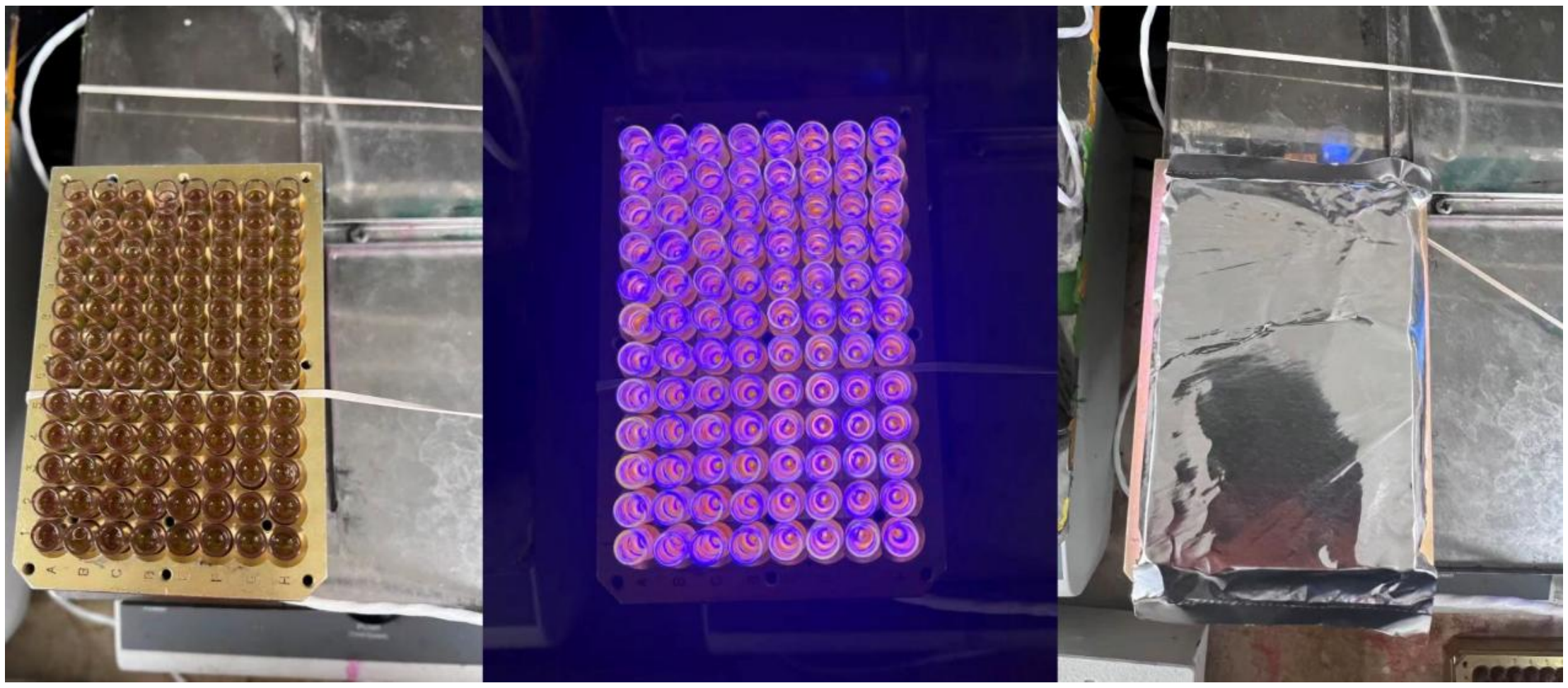

Figure S2. Reaction screening in a 96-well plate format.

Product formation, enantioselectivity screening using normal-phase chiral HPLC. The reaction mixtures in the 96-position Paradox reactor were carefully and completely transferred to a deep-well 96-well plate using an Eppendorf Xplorer 12-channel pipette (50–1000 μL). EtOAc (600 μL/well) with mesitylene (1 mM in EtOAc) as the internal standard was then added to each well of the 96-well plate using a 12-channel pipette. This 96-well plate containing the resulting mixture was tightly sealed with a reusable silicone mat, shaken vigorously (30 times), and then centrifuged with an Eppendorf 5910R tabletop centrifuge (4500 rpm, 20 min) to separate the organic and aqueous layers. The organic supernatant (200 μL/well) was transferred to 500 μL vial inserts using an Eppendorf Xplorer 12-channel electronic pipette (15–300 μL). The inserts were then placed in 2 mL vials and analyzed by normal-phase chiral HPLC using conditions detailed below.

Determination of yields using GC-MS analysis. Stock solutions of authentic products (100 mM in DMSO) were prepared (at least 20 mg sample was weighed to make a 100 mM stock solution in DMSO). 1 L extraction solvent with internal standard was freshly prepared (1 mmol (139 μL) of mesitylene was added to 1 L EtOAc. To a microcentrifuge tube were added 1 mL of KPi buffer, 2, 4, 8, 16, 32, 40 μL product stock solution, respectively, and 0.6 mL extraction solvent. The mixture was vortexed (20 s for 3 times) and centrifuged (15,000 rpm, 5 min) using an Eppendorf microcentrifuge 5424R to separate the organic and aqueous layers. The organic layer was transferred to a vial with a 500 μL insert for GC-MS analysis.

Experimental procedure for the expression of *Ape*Pgb variants

Method A. A single colony from LB_{amp} agar plate was picked using a sterile toothpick and cultured in LB_{amp} media (30 mL) at 37 °C and 230 rpm for 11–12 h. This preculture was used to inoculate 0.6 L HB_{amp} media (AthenaES, Lot# 0107-27118) in a 2 L Erlenmeyer flask. The expression culture was incubated at 37 °C and 230 rpm for 2.5–3 h, until OD_{600} reached 0.9–1.2. The culture was cooled on ice for 20 min and induced with 0.50 mM isopropyl-*β*-D-thiogalactopyranoside (IPTG) and 1.0 mM 5-aminolevulinic acid (ALA) (final concentrations). Protein expression was conducted at 20 °C and 200 rpm for 20 h. *E. coli* cells were harvested by centrifugation at 8,000 rpm and 4 °C for 8 min using a Thermo Scientific Sorvall Lynx 6000 superspeed centrifuge. The cell pellet was then flash frozen with liquid nitrogen and stored in a –80 °C freezer until further use. Using this protocol, the heme loading of *Ape*Pgb variants was 40–45%.

Method B. The plasmid pChuA was used as part of a two-plasmid system to facilitate heme incorporation into *Ape*Pgb.[3] Electrocompetent *E. coli* BL21(DE3) cells were transformed with pET-22b(+)-*Ape*Pgb (ampicillin resistance) and pChuA (kanamycin resistance). A single colony from $LB_{amp/kan}$ agar plate was picked using a sterile toothpick and cultured in $LB_{amp/kan}$ media (30 mL) at 37 °C and 230 rpm for 11–12 h. This preculture was used to inoculate 0.6 L $HB_{amp/kan}$ media (AthenaES, Lot# 0107-31065) in a 2 L Erlenmeyer flask. The expression culture was incubated at 37 °C and 230 rpm for 2.5-3 h, until OD_{600} reached 0.9–1.2. The culture was cooled on ice for 20 min and induced with 0.50 mM isopropyl-*β*-D-thiogalactopyranoside (IPTG), 1.0 mM 5-aminolevulinic acid (ALA) (final concentrations) and 30 mg/L hemin (3.0 g/L stock solution in DMSO) (final concentrations). Protein expression was conducted at 20 °C and 200 rpm for 20 h. *E. coli* cells were harvested by centrifugation at 8,000 rpm and 4 °C for 8 min using a Thermo Scientific Sorvall Lynx 6000 superspeed centrifuge. The cell pellet was then flash frozen with liquid nitrogen and stored in a –80 °C freezer until further use. Using this protocol, the heme loading of *Ape*Pgb variants was 75–80%.

Experimental procedure for the purification of *Ape*Pgb variants. Frozen cells were resuspended in buffer A (25 mM Tris-HCl, 100 mM NaCl, 25 mM imidazole, pH 7.4, 2–3 mL/g wet cells). The mixture was suspended and lysed by sonication using a Qsonica Q500 or Q700 sonicator equipped with a standard probe (tip diameter = ½’’). To pellet cell debris, lysates were centrifuged using a Lynx 6000 superspeed centrifuge (15,000 rpm, 2 h, 4 °C). *Ape*Pgb variants

containing a C-terminal 6×His tag was purified with a Ni-NTA column (5 mL HisTrap HP, GE Healthcare, Piscataway, NJ) using an AKTA Start protein purification system (GE healthcare). The column was first flushed with buffer A (25 mM Tris-HCl, 100 mM NaCl, 25 mM imidazole, pH 7.4) for 10 column volumes (CVs), then eluted with 30% buffer B (25 mM Tris-HCl, 100 mM NaCl, 500 mM imidazole, pH 7.4)/70% buffer A (168 mM imidazole in this elution buffer consisting of 70% buffer A and 30% buffer B) over 10 column volumes (CVs). Fractions containing the desired *Ape*Pgb variant were combined. The protein solution was concentrated and subjected to three rounds of buffer exchange using the storage buffer (buffer C, 200 mM KPi, pH 7.4) using ultracentrifuge filters (10 kDa molecular weight cut-off, Amicon Ultra, MilliporeSigma) to remove excess salt and imidazole. Protein concentration was first measured with Nanodrop and normalized to ca. 20–50 mg/mL. Concentrated proteins were aliquoted and flash-frozen in liquid N_2 and stored at –80 °C until further use. Protein concentration was also determined by the bicinchoninic acid (BCA) assay[4] prior to their use in biocatalytic reactions. The BCA assay was performed using Thermo Scientific's BCA protein assay kit (catalog #: A55864).

Hemochrome assay for the determination of heme protein concentration. *E. coli* cells expressing heme protein resuspended in KPi buffer (200 mM, pH 7.4) were lysed by sonication using a QSonica Q500 sonicator equipped with a 24-tip horn (6 min in total, 2 sec on, 4 sec off, 45% amplitude); samples were submerged in wet ice for this process. The resulting lysed solution was centrifuged (15,000 rpm, 2 h, 4 °C) using an Eppendorf centrifuge 5425R to remove the cell debris. The supernatant (clarified lysate) was separated from the pellet and kept on ice until use. In a conical tube, a solution of 0.2 M NaOH, 40% (v/v) pyridine, 0.5 mM $K_3Fe(CN)_6$ was prepared (pyridine-NaOH-$K_3Fe(CN)_6$ solution). In another 1.5 mL centrifuge tube, a solution of 0.5 M $Na_2S_2O_4$ (sodium dithionite) was prepared in 0.1 M NaOH. 500 µL clarified lysate in KPi buffer and 500 µL pyridine-NaOH-$K_3Fe(CN)_6$ solution were transferred to a cuvette and carefully mixed to avoid air bubbles. The UV-Vis spectrum of the oxidized Fe^{III} state was recorded immediately. To the cuvette was then added 10 µL sodium dithionite solution. The cuvette was sealed with parafilm and the UV-Vis spectrum of the reduced Fe^{II} state was recorded immediately. A cuvette containing 500 µL KPi buffer, 500 µL pyridine-NaOH-$K_3Fe(CN)_6$ solution was used as a reference for all absorbance measurements. Concentrations of *Ape*Pgb variants were determined using a published extinction coefficient for heme *b* ($\varepsilon_{550(reduced)-600(oxidized)}$ = 34.7 $mM^{-1}cm^{-15}$).[5]

II. General experimental procedure

General experimental procedure for photometallobiocatalytic C–C coupling. The metalloprotein variant was allowed to thaw and kept on top of ice. In a Coy anaerobic chamber, the following stock solutions were prepared: organotrifluoroborate salt RBF_3K **1** (100 mM in degassed DMSO), photocatalyst (20 mM in degassed DMSO), and pyridotriazole compound **2** (200 mM in degassed DMSO). To a 2-dram vial containing a stir bar were added the KPi buffer (200 mM, pH 7.4), 20 μL RBF_3K stock solution, 20 μL pyridotriazole compound stock solution, 10 μL photocatalyst stock solution, followed by the metalloprotein stock solution (the specific volume of the metalloprotein stock solution was dependent on the concentration of the specific metalloprotein sample; a typical concentration of *Ape*Pgb variant is 800 μM and a typical volume is 50 μL (1 mol%)). The total reaction volume was 1.0 mL; the final concentrations of each reaction component were as follows: 2.0 mM organotrifluoroborate salt **1**, 4.0 mM pyridotriazole **2**, 20 μM metalloprotein catalyst, and 200 μM photoredox catalyst. The reaction vial was then sealed and removed from the Coy anaerobic chamber and submerged in a water bath at room temperature. The reaction mixture was allowed to stir at 500 rpm on a stir plate and illuminated with one Kessil LED lamp (525 nm, 25% power output, 45 W) for 12 h. A representative experimental setup is shown below. The distance between the Kessil LED lamp and the reaction tube is 24 cm (Note: maintaining this LED lamp–reaction tube distance was critical to ensure optimal photobiocatalytic reaction performance).

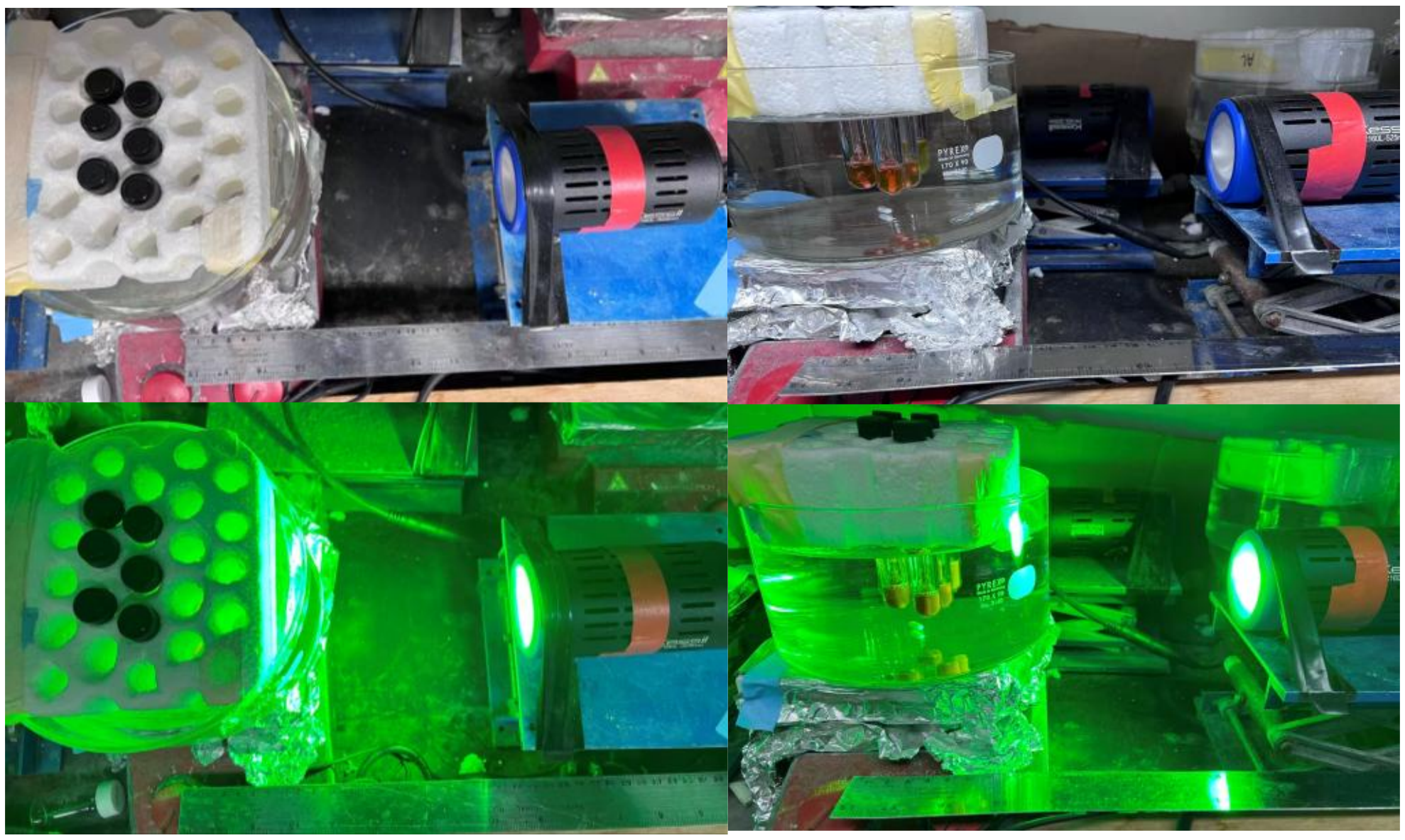

Figure S3. Photometallobiocatalytic reaction setup.

Upon completion, the reaction mixture was quenched by the addition of 0.6 mL EtOAc with mesitylene (1.0 mM) as the internal standard. After vigorous mixing, the reaction mixture was transferred into a 2 mL microcentrifuge tube and centrifuged at 15,000 rpm using an Eppendorf microcentrifuge 5424R for 5 min. The organic layer (ca. 300 µL) was transferred to a 2 mL glass vial with a 500 µL insert and analyzed by GC-MS and chiral HPLC. The yield was determined by GC-MS using mesitylene as a standard with the aid of calibration curves. The enantioselectivity was determined by chiral HPLC.

III. Selected results for reaction development.

Table S1. Evaluation of reaction conditions for photometallobiocatalytic C–C coupling.[a]

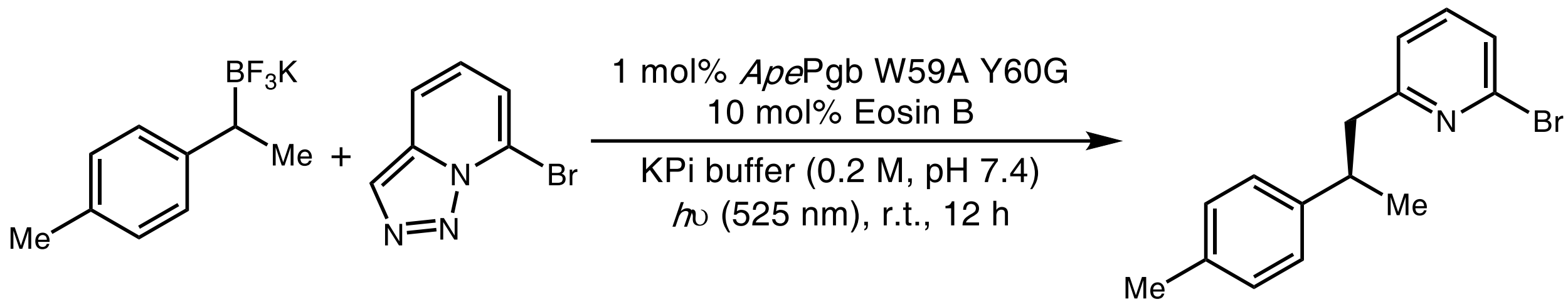

1a (1.0 equiv) **2a** (2.0 equiv) **3a**

entry	concentration of **1a** (mM)	lamp	Distance (cm)	yield of **3a** (%)	e.r. of **3a**
1	4	2	12	42	80:20
2	2	2	12	71	83:17
3	4	1	24	51	80:20
4	2	1	24	88	83:17

[a]Reaction conditions: DMSO (5% v/v). 1 mol% *Ape*Pgb W59A Y60G (20 μM), Kessil LED lamp (45 W, 25%), *hν* (525 nm), KPi buffer (0.2 M, pH 7.4), room temperature, 12 h.

Table S2. Summary of directed evolution of *Ape*Pgb^{LGGTS} from *Ape*Pgb W59A Y60G.

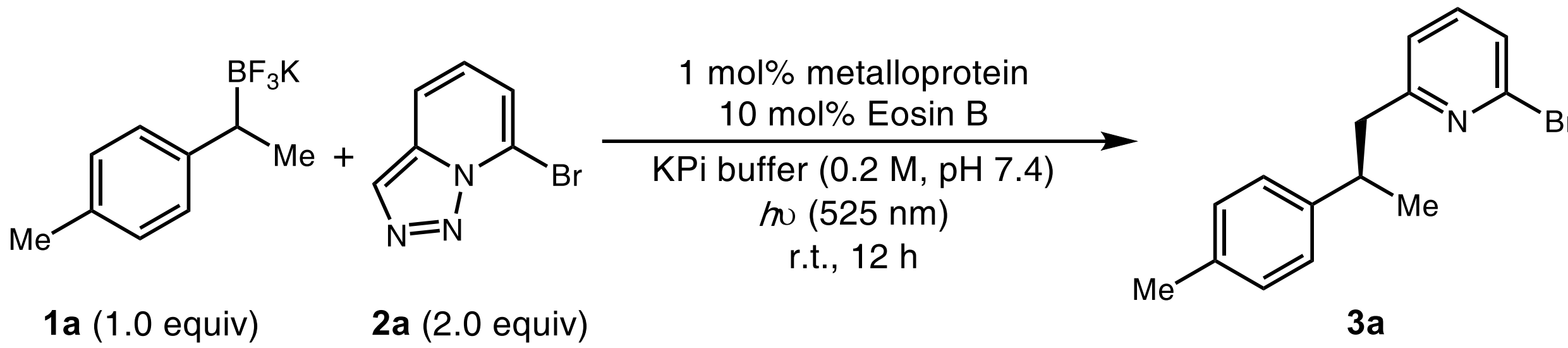

Parent	Sites Targeted by SSM	Selection Criteria	Beneficial Mutation(s)
*Ape*Pgb W59A Y60G	A59X, Y60X	Enantioselectivity	A59L
*Ape*Pgb W59A Y60G A59L	L69X, W96X and F145X	Enantioselectivity	F145G
*Ape*Pgb W59A Y60G A59L F145G	W62X, R90X, F93X and I149X	Enantioselectivity	F93T
*Ape*Pgb W59A Y60G A59L F145G F93T	F73X, L86X and P148X	Enantioselectivity and activity	P148S
*Ape*Pgb W59A Y60G A59L F145G F93T P148S (***Ape*Pgb^{LGGTS}**)	-	-	-

Table S3. Directed evolution of *Ape*Pgb^{LGGTS}: summary of activity and enantioselectivity.[a]

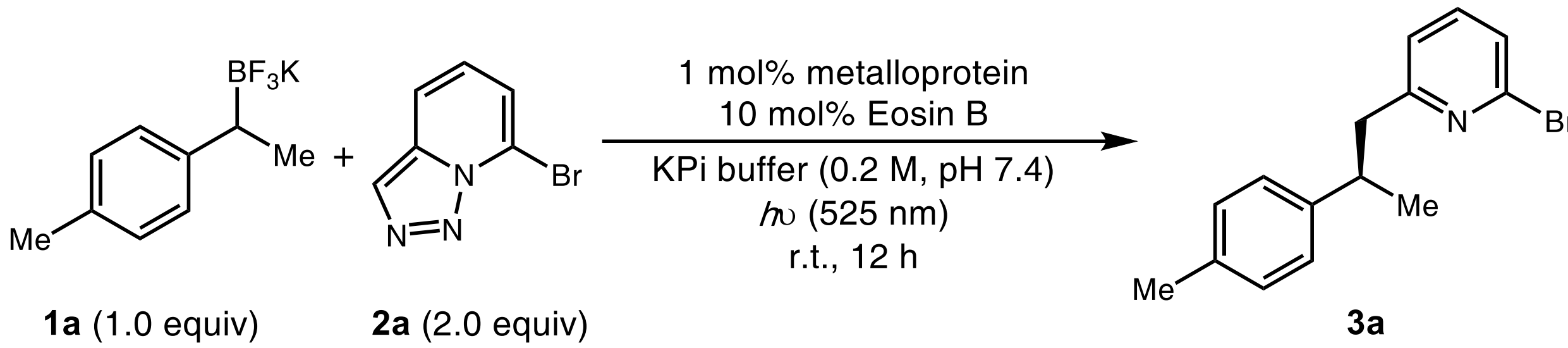

metalloenzyme	yield of **3a** (%)	e.r. of **3a**
*Ape*Pgb W59A Y60G	88±1	83:17
*Ape*Pgb W59A Y60G A59L	81±3	85:15
*Ape*Pgb W59A Y60G A59L F145G (***Ape*Pgb**$^{\mathbf{LGG}}$)	82±4	92:8
*Ape*Pgb W59A Y60G A59L F145G F93T	80±6	94:6
*Ape*Pgb W59A Y60G A59L F145G F93T P148S (***Ape*Pgb**$^{\mathbf{LGGTS}}$)	86±1	97:3

[a]Reaction conditions: **1a** (2.0 mM), **2a** (4.0 mM), DMSO (5% v/v). 1 mol% metalloenzyme (20 μM), Kessil LED lamp (45 W, 25%), *hν* (525 nm), KPi buffer (0.2 M, pH 7.4), room temperature, 12 h.

Table S4. Control experiments shedding light on each reaction component. [a]

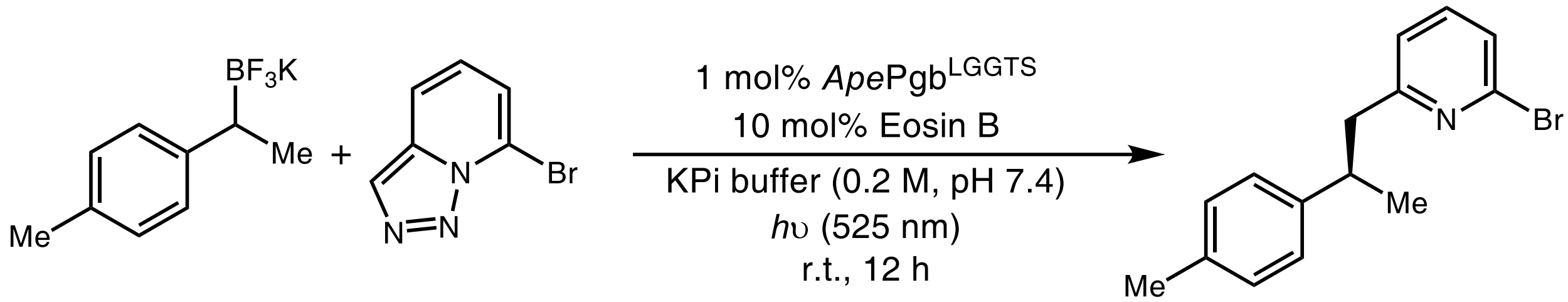

1a (1.0 equiv) **2a** (2.0 equiv) **3a**

entry	Variation from standard conditions	yield of **3a** (%)	e.r. of **3a**
1	-	86 ± 1	97:3
2	w/o *Ape*Pgb^{LGGTS}	0	-
3	1.5 equiv **2a**	77 ± 2	97:3
4	open to air	0	-
5	w/o eosin B	0	-
6	w/o light	0	-

[a]Reaction conditions: **1a** (2.0 mM), **2a** (4.0 mM), DMSO (5% v/v). 1 mol% *Ape*Pgb^{LGGTS} (20 μM), Kessil LED lamp (45 W, 25%), $h\nu$ (525 nm), KPi buffer (0.2 M, pH 7.4), room temperature, 12 h.

Table S5. Effect of BSA and Surfactant additives on the photobiocatalytic reaction. [a]

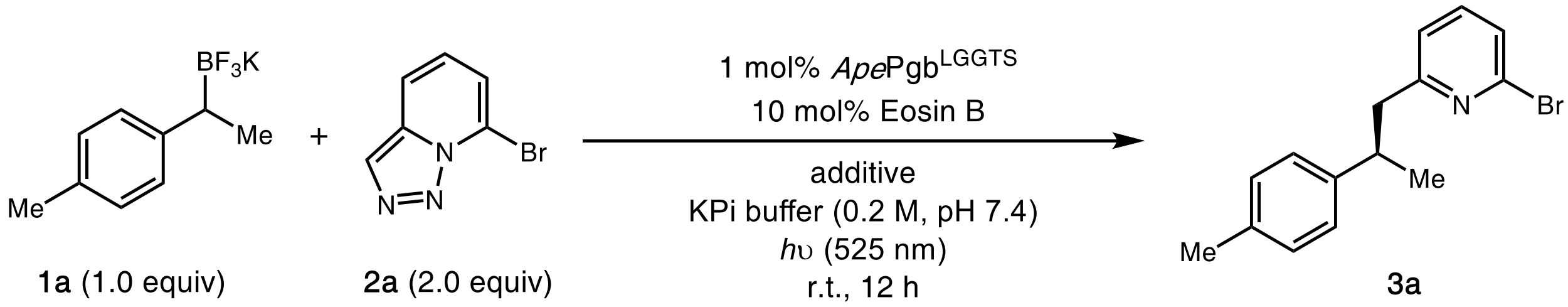

entry	additive	yield of **3a** (%)	e.r. of **3a**
1	2 wt% BSA	11	85:15
2	2 wt% TPGS-750-M	14	87:13
3	2 wt% β-cyclodextrin	16	54:46
4	2 wt% Brij 35	56	56:44
5	2 wt% Triton X-100	43	61:39

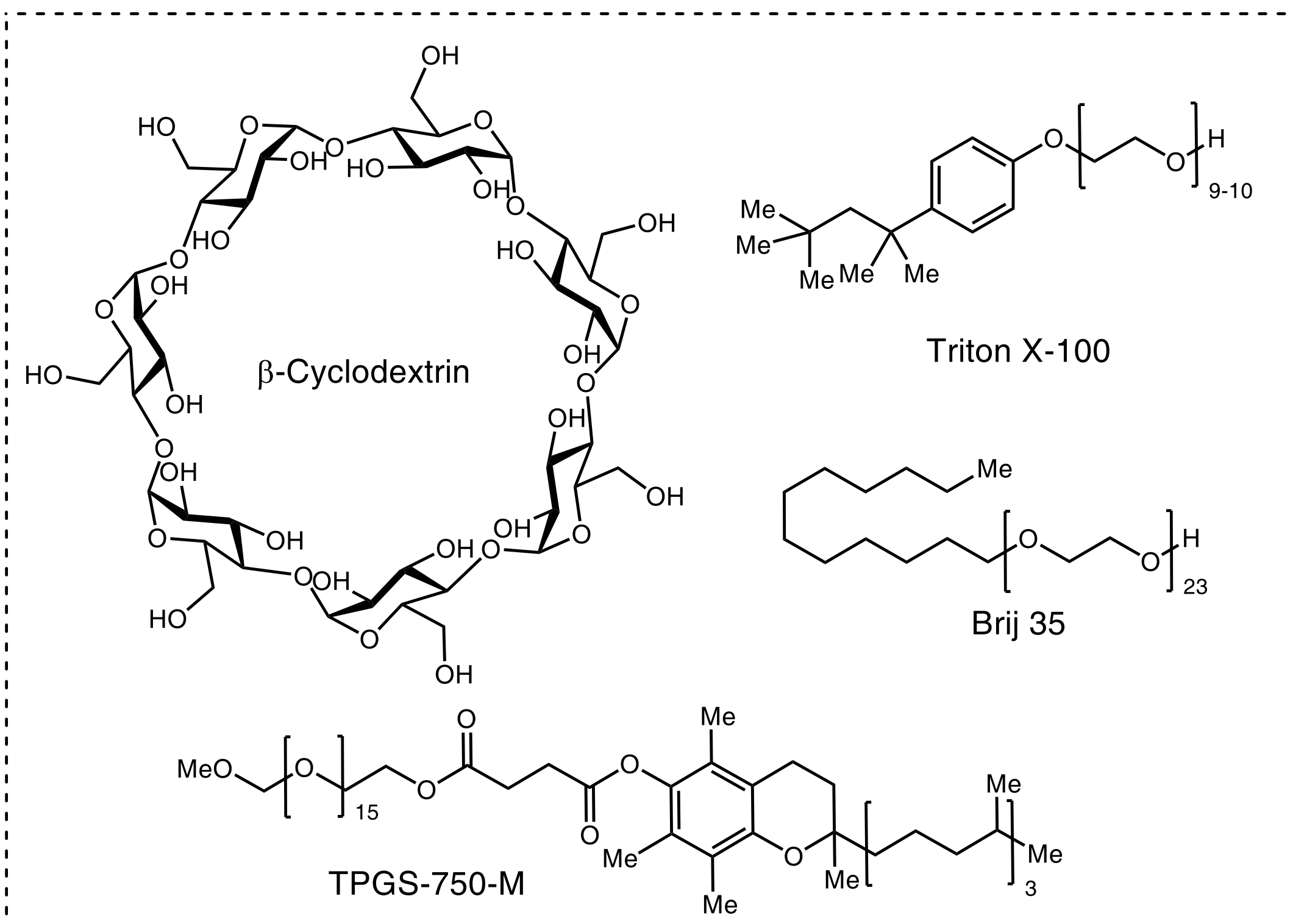

[a]Reaction conditions: **1a** (2.0 mM), **2a** (4.0 mM), DMSO (5% v/v). 1 mol% *Ape*Pgb^{LGGTS} (20 μM), additive (2 wt%), Kessil LED lamp (45 W, 25%), *hν* (525 nm), KPi buffer (0.2 M, pH 7.4), room temperature, 12 h.

IV. DNA and protein sequences

*Ape*Pgb W59A Y60G

DNA sequence

ATGACTCCCTCGGACATCCCGGGATATGATTATGGGCGTGTCGAGAAGTCACCCATCACGGACC
TTGAGTTTGACCTTCTGAAGAAGACTGTCATGTTAGGTGAAAAGGACGTAATGTACTTGAAAAA
GGCGTGTGACGTTCTGAAAGATCAAGTTGATGAGATCCTTGACTTGGCGGGTGGTTGGGTAGCA
TCAAATGAGCATTTGATTTATTACTTCTCCAATCCGGATACAGGAGAGCCTATTAAGGAATACC
TGGAACGTGTACGCGCTCGCTTTGGAGCCTGGATTCTGGACACTACCTGCCGCGACTATAACCG
TGAATGGTTAGACTACCAGTACGAAGTTGGGCTTCGTCATCACCGTTCAAAGAAAGGGGTCACA
GACGGAGTACGCACCGTGCCCCATATCCCACTTCGTTATCTTATCGCATTTATCTATCCTATCA
CCGCCACTATCAAGCCATTTTTGGCTAAGAAAGGTGGCTCTCCGGAAGACATCGAAGGGATGTA
CAACGCTTGGTTCAAGTCTGTAGTTTTACAAGTTGCCATCTGGTCACACCCTTATACTAAGGAG
AATGACTGGCTCGAGCACCACCACCACCACCACTGA

Protein sequence

MTPSDIPGYDYGRVEKSPITDLEFDLLKKTVMLGEKDVMYLKKACDVLKDQVDEILDLWYGWVA
SNEHLIYYFSNPDTGEPIKEYLERVRARFGAWILDTTCRDYNREWLDYQYEVGLRHHRSKKGVT
DGVRTVPHIPLRYLIAFIYPITATIKPFLAKKGGSPEDIEGMYNAWFKSVVLQVAIWSHPYTKE
NDWLEHHHHHH*

*Ape*Pgb W59A Y60G A59L

DNA sequence

ATGACTCCCTCGGACATCCCGGGATATGATTATGGGCGTGTCGAGAAGTCACCCATCACGGACC
TTGAGTTTGACCTTCTGAAGAAGACTGTCATGTTAGGTGAAAAGGACGTAATGTACTTGAAAAA
GGCGTGTGACGTTCTGAAAGATCAAGTTGATGAGATCCTTGACTTG**CTG**GGTGGTTGGGTAGCA
TCAAATGAGCATTTGATTTATTACTTCTCCAATCCGGATACAGGAGAGCCTATTAAGGAATACC
TGGAACGTGTACGCGCTCGCTTTGGAGCCTGGATTCTGGACACTACCTGCCGCGACTATAACCG
TGAATGGTTAGACTACCAGTACGAAGTTGGGCTTCGTCATCACCGTTCAAAGAAAGGGGTCACA
GACGGAGTACGCACCGTGCCCCATATCCCACTTCGTTATCTTATCGCATTTATCTATCCTATCA
CCGCCACTATCAAGCCATTTTTGGCTAAGAAAGGTGGCTCTCCGGAAGACATCGAAGGGATGTA

CAACGCTTGGTTCAAGTCTGTAGTTTTACAAGTTGCCATCTGGTCACACCCTTATACTAAGGAG

AATGACTGGCTCGAGCACCACCACCACCACCACTGA

Protein sequence

MTPSDIPGYDYGRVEKSPITDLEFDLLKKTVMLGEKDVMYLKKACDVLKDQVDEILDL**L**GGWVA

SNEHLIYYFSNPDTGEPIKEYLERVRARFGAWILDTTCRDYNREWLDYQYEVGLRHHRSKKGVT

DGVRTVPHIPLRYLIAGIYPITATIKPFLAKKGGSPEDIEGMYNAWFKSVVLQVAIWSHPYTKE

NDWLEHHHHHH*

***Ape*Pgb W59A Y60G A59L F145G**

(*Ape*Pgb^{LGG})

DNA sequence

ATGACTCCCTCGGACATCCCGGGATATGATTATGGGCGTGTCGAGAAGTCACCCATCACGGACC

TTGAGTTTGACCTTCTGAAGAAGACTGTCATGTTAGGTGAAAAGGACGTAATGTACTTGAAAAA

GGCGTGTGACGTTCTGAAAGATCAAGTTGATGAGATCCTTGACTTG**CTG**GGTGGTTGGGTAGCA

TCAAATGAGCATTTGATTTATTACTTCTCCAATCCGGATACAGGAGAGCCTATTAAGGAATACC

TGGAACGTGTACGCGCTCGCTTTGGAGCCTGGATTCTGGACACTACCTGCCGCGACTATAACCG

TGAATGGTTAGACTACCAGTACGAAGTTGGGCTTCGTCATCACCGTTCAAAGAAAGGGGTCACA

GACGGAGTACGCACCGTGCCCCATATCCCACTTCGTTATCTTATCGCA**GGT**ATCTATCCTATCA

CCGCCACTATCAAGCCATTTTTGGCTAAGAAAGGTGGCTCTCCGGAAGACATCGAAGGGATGTA

CAACGCTTGGTTCAAGTCTGTAGTTTTACAAGTTGCCATCTGGTCACACCCTTATACTAAGGAG

AATGACTGGCTCGAGCACCACCACCACCACCACTGA

Protein sequence

MTPSDIPGYDYGRVEKSPITDLEFDLLKKTVMLGEKDVMYLKKACDVLKDQVDEILDL**L**GGWVA

SNEHLIYYFSNPDTGEPIKEYLERVRARFGAWILDTTCRDYNREWLDYQYEVGLRHHRSKKGVT

DGVRTVPHIPLRYLIA**G**IYPITATIKPFLAKKGGSPEDIEGMYNAWFKSVVLQVAIWSHPYTKE

NDWLEHHHHHH*

***Ape*Pgb W59A Y60G A59L F145G F93T**

DNA sequence

ATGACTCCCTCGGACATCCCGGGATATGATTATGGGCGTGTCGAGAAGTCACCCATCACGGACC
TTGAGTTTGACCTTCTGAAGAAGACTGTCATGTTAGGTGAAAAGGACGTAATGTACTTGAAAAA
GGCGTGTGACGTTCTGAAAGATCAAGTTGATGAGATCCTTGACTTG**CTG**GGTGGTTGGGTAGCA
TCAAATGAGCATTTGATTTATTACTTCTCCAATCCGGATACAGGAGAGCCTATTAAGGAATACC
TGGAACGTGTACGCGCTCGC**ACG**GGAGCCTGGATTCTGGACACTACCTGCCGCGACTATAACCG
TGAATGGTTAGACTACCAGTACGAAGTTGGGCTTCGTCATCACCGTTCAAAGAAAGGGGTCACA
GACGGAGTACGCACCGTGCCCCATATCCCACTTCGTTATCTTATCGCA**GGT**ATCTATCCTATCA
CCGCCACTATCAAGCCATTTTTGGCTAAGAAAGGTGGCTCTCCGGAAGACATCGAAGGGATGTA
CAACGCTTGGTTCAAGTCTGTAGTTTTACAAGTTGCCATCTGGTCACACCCTTATACTAAGGAG
AATGACTGGCTCGAGCACCACCACCACCACCACTGA

Protein sequence

MTPSDIPGYDYGRVEKSPITDLEFDLLKKTVMLGEKDVMYLKKACDVLKDQVDEILDL**L**GGWVA
SNEHLIYYFSNPDTGEPIKEYLERVRAR**T**GAWILDTTCRDYNREWLDYQYEVGLRHHRSKKGVT
DGVRTVPHIPLRYLIA**G**IYPITATIKPFLAKKGGSPEDIEGMYNAWFKSVVLQVAIWSHPYTKE
NDWLEHHHHHH*

_Ape_Pgb W59A Y60G A59L F145G F93T P148S

(_Ape_Pgb^{LGGTS})

DNA sequence

ATGACTCCCTCGGACATCCCGGGATATGATTATGGGCGTGTCGAGAAGTCACCCATCACGGACC
TTGAGTTTGACCTTCTGAAGAAGACTGTCATGTTAGGTGAAAAGGACGTAATGTACTTGAAAAA
GGCGTGTGACGTTCTGAAAGATCAAGTTGATGAGATCCTTGACTTG**CTG**GGTGGTTGGGTAGCA
TCAAATGAGCATTTGATTTATTACTTCTCCAATCCGGATACAGGAGAGCCTATTAAGGAATACC
TGGAACGTGTACGCGCTCGC**ACG**GGAGCCTGGATTCTGGACACTACCTGCCGCGACTATAACCG
TGAATGGTTAGACTACCAGTACGAAGTTGGGCTTCGTCATCACCGTTCAAAGAAAGGGGTCACA
GACGGAGTACGCACCGTGCCCCATATCCCACTTCGTTATCTTATCGCA**GGT**ATCTAT**AGT**ATCA
CCGCCACTATCAAGCCATTTTTGGCTAAGAAAGGTGGCTCTCCGGAAGACATCGAAGGGATGTA
CAACGCTTGGTTCAAGTCTGTAGTTTTACAAGTTGCCATCTGGTCACACCCTTATACTAAGGAG
AATGACTGGCTCGAGCACCACCACCACCACCACTGA

Protein sequence

MTPSDIPGYDYGRVEKSPITDLEFDLLKKTVMLGEKDVMYLKKACDVLKDQVDEILDL**L**GGWVA
SNEHLIYYFSNPDTGEPIKEYLERVRAR**T**GAWILDTTCRDYNREWLDYQYEVGLRHHRSKKGVT
DGVRTVPHIPLRYLIA**G**IY**S**ITATIKPFLAKKGGSPEDIEGMYNAWFKSVVLQVAIWSHPYTKE
NDWLEHHHHHH*

Table S6. Primers used for site-saturation mutagenesis using the 22codon trick method.

DNA Template	Primer name	Primer sequence
*Ape*Pgb W59A Y60G	A59X_fwd_NDT	GATGAGATCCTTGACTTG**NDT**GGTGGTTGGGTAGCATC
	A59X_fwd_TGG	GATGAGATCCTTGACTTG**TGG**GGTGGTTGGGTAGCATC
	A59X_fwd_VHG	GATGAGATCCTTGACTTG**VHG**GGTGGTTGGGTAGCATC
	A59X_rev	CAAGTCAAGGATCTCATCAACTTGATC
*Ape*Pgb W59A Y60G A59L	F145X_fwd_NDT	CTTCGTTATCTTATCGCA**NDT**ATCTATAGTATC
	F145X_fwd_TGG	CTTCGTTATCTTATCGCA**TGG**ATCTATAGTATC
	F145X_fwd_VHG	CTTCGTTATCTTATCGCA**VHG**ATCTATAGTATC
	F145X_rev	TGCGATAAGATAACGAAGTGGGATA
*Ape*Pgb W59A Y60G A59L F145G	F93X_fwd_NDT	ACGTGTACGCGCTCGC**NDT**GGAGCCTGGATTCTGGAC
	F93X_fwd_TGG	ACGTGTACGCGCTCGC**TGG**GGAGCCTGGATTCTGGAC
	F93X_fwd_VHG	ACGTGTACGCGCTCGC**VHG**GGAGCCTGGATTCTGGAC
	F93X_rev	GCGAGCGCGTACACGTTCCAG
*Ape*Pgb W59A Y60G A59L F145G F93T	P148X_fwd_NDT	CTTATCGCAGGTATCTAT**NDT**ATCACCGCCACTATC
	P148X_fwd_TGG	CTTATCGCAGGTATCTAT**TGG**ATCACCGCCACTATC
	P148X_fwd_VHG	CTTATCGCAGGTATCTAT**VHG**ATCACCGCCACTATC
	P148X_rev	ATAGATACCTGCGATAAGATAACGAAG

V. Kinetic and fluorescence anisotropy titration data

Table S7. Time course of the photobiocatalytic transformation of (*rac*)-1a showed no kinetic resolution. [a]

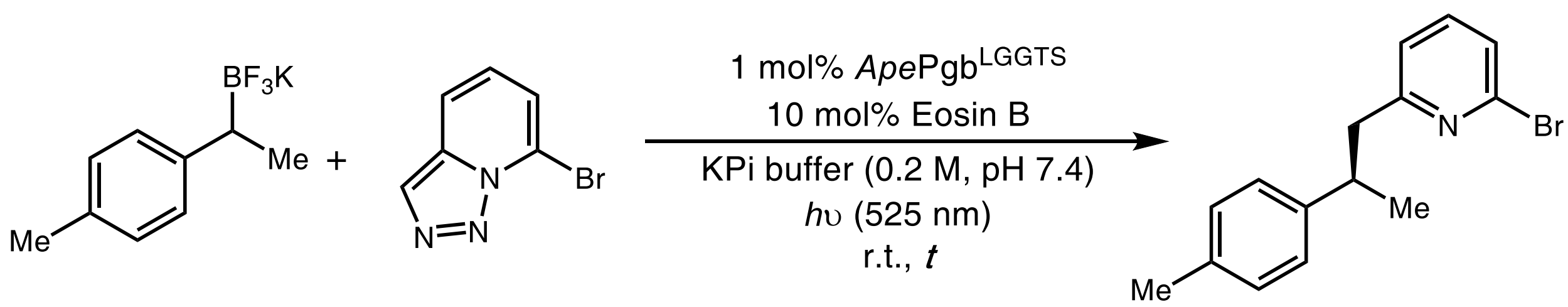

(*rac*)-**1a** 1.0 equiv   **2a** 2.0 equiv   **3a**

Time	yield of **3a**	e.r. of **3a**	e.r. of recovered **1a**
5 min	24%	97 : 3	50 : 50
10 min	27%	97 : 3	50 : 50
20 min	31%	97 : 3	50 : 50
40 min	40%	97 : 3	50 : 50
1 h	47%	97 : 3	50 : 50
2 h	53%	97 : 3	50 : 50
3 h	65%	97 : 3	50 : 50
5 h	76%	97 : 3	50 : 50
8 h	85%	97 : 3	n.d.
12 h	86%	97 : 3	n.d.

[a]Reaction conditions: (*rac*)-**1a** (2.0 mM), **2a** (4.0 mM), DMSO (5% v/v). 1 mol% *Ape*Pgb^{LGGTS}, Kessil LED (45 W, 25%), *hν* (525 nm), KPi buffer (pH 7.4), room temperature. n.d. = not detected.

Experimental procedure: Following the general procedure detailed above, the biocatalytic transformation of (*rac*)-**1a** was quenched at 5 min and 15 min and analyzed. The yields of the product **3a** were determined by normal phase HPLC. The enantioselectivity of the recovered starting material **1a** was determined using normal phase HPLC after oxidation with Oxone to the corresponding alcohol, a known stereoretentive process[6]. The alcohol was extracted into EtOAc/hexanes and analyzed by chiral HPLC. HPLC analysis conditions: Chiralpak IG column

(4.6 mm × 25 cm, 5 micron), 98 : 2 hexanes : *i*-PrOH, 1.2 mL/min, 40 °C. t_R = 10.3 min and 11.0 min, respectively.

5. Fluorescence anisotropy titration.

Fluorescence anisotropy (FA) titration was performed to determine the dissociation constant (K_d) of different photocatalysts and metalloenzyme *Ape*Pgb W59A Y60G. Fluorescence anisotropy is a photophysical tool to study the interactions of protein and fluorophore. By monitoring the changes in the rotational mobility of photocatalysts upon binding to proteins, fluorescence anisotropy allows the quantification of PC–*Ape*Pgb W59A Y60G binding strength.

Experimental procedure. In a 2 mL centrifuge tube, a 500 nM stock solution of PC in 200 mM KPi buffer (pH = 7.4) was prepared. Using a storage solution of *Ape*Pgb W59A Y60G (200 mM KPi buffer, pH = 7.4), a series of *Ape*Pgb W59A Y60G solutions with a concentration of 2.0 µM, 4.0 µM, 8.0 µM, 16.0 µM, 32.0 µM, 64.0 µM, 128.0 µM, 256.0 µM, and 512.0 µM were next prepared using 200 mM KPi buffer (pH = 7.4) as the diluent. All fluorescence anisotropy readings are reported as an average of triplicates. Fluorescence anisotropy measurements were carried out using a Tecan Spark Microplate Reader (Part No. 30124664).

To each well of a black 96-well microplate (Greiner Bio-One FLUOTRAC™ 200), were added 50 µL PC stock solution (500 nM in 200 mM KPi buffer, pH = 7.4). 50 µL *Ape*Pgb W59A Y60G solution of varying concentrations as detailed above was added to each well. The resulting solutions were mixed gently using a multichannel pipette. The final concentration of PC is 250 nM. In this measurement, each data point was measured in triplicated.

Fitting formula: $FA = A + B_{max}[E]/(K_d + [E])$, [E]: enzyme concentration.

Anisotropy (vs. single point) is taken with excitation at 440 nm and emission at 600 nm.

Table S8. Fluorescence anisotropy titration with *Ape*Pgb W59A Y60G–eosin B.

entry	enzyme concentration (μM)	FA (1st run)	FA (2nd run)	FA (3rd run)	average fluorescence anisotropy (FA)	CV
1	1	0.045	0.05	0.044	0.046	0.0026
2	2	0.05	0.049	0.051	0.05	0.00082
3	4	0.054	0.057	0.056	0.056	0.0012
4	8	0.063	0.064	0.064	0.064	0.00047
5	16	0.068	0.066	0.067	0.067	0.00082
6	32	0.071	0.07	0.07	0.07	0.00047
7	64	0.073	0.072	0.072	0.072	0.00047
8	128	0.074	0.075	0.073	0.074	0.00082
9	256	0.73	0.074	0.073	0.073	0.00047

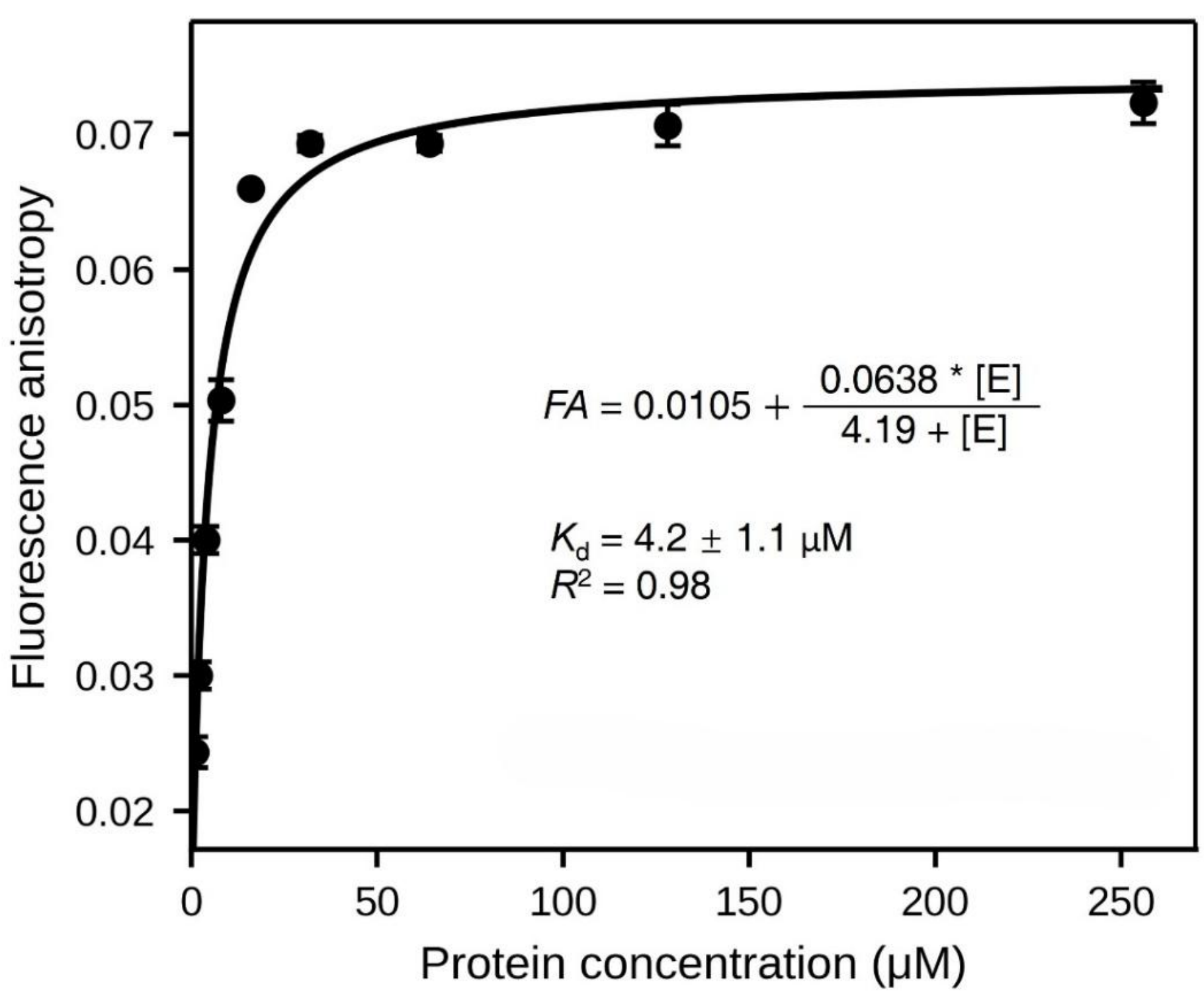

Figure S4. Fluorescence anisotropy titration of the *Ape*Pgb W59A Y60G–eosin B.

Table S9. Fluorescence anisotropy titration with *Ape*Pgb W59A Y60G–eosin Y.

entry	enzyme concentration (μM)	FA (1st run)	FA (2nd run)	FA (3rd run)	average fluorescence anisotropy (FA)	CV
1	1	0.046	0.048	0.049	0.048	0.0012
2	2	0.049	0.055	0.053	0.052	0.0025
3	4	0.057	0.062	0.06	0.06	0.0021
4	8	0.063	0.068	0.064	0.065	0.0022
5	16	0.07	0.069	0.068	0.069	0.00082
6	32	0.072	0.073	0.073	0.073	0.00047
7	64	0.074	0.075	0.074	0.074	0.00047
8	128	0.075	0.075	0.073	0.074	0.00094
9	256	0.076	0.075	0.074	0.075	0.00082

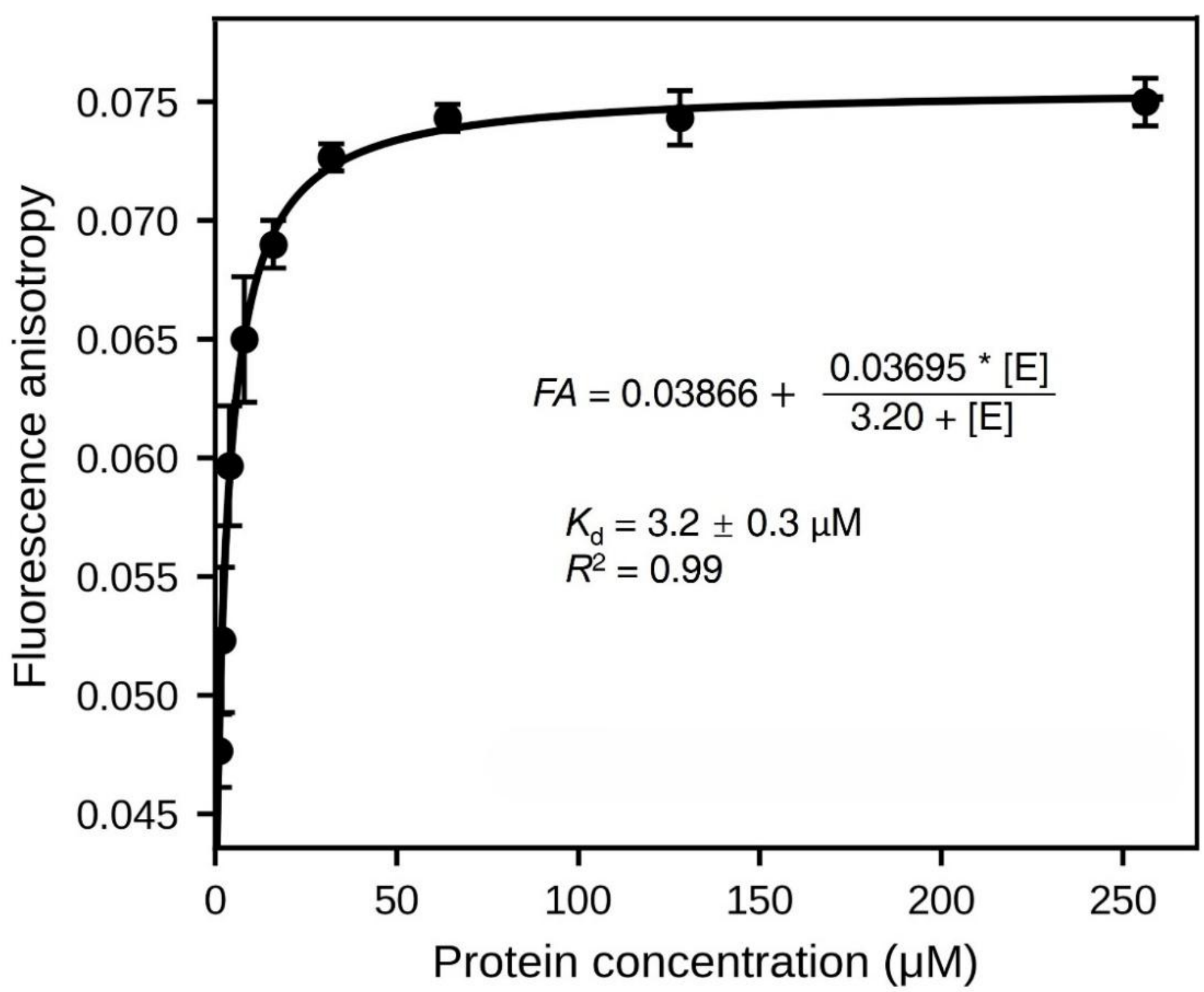

Figure S5. Fluorescence anisotropy titration with *Ape*Pgb W59A Y60G–eosin Y.

Table S10. Fluorescence anisotropy titration with *Ape*Pgb W59A Y60G–rhodamine B.

entry	enzyme concentration (μM)	FA (1st run)	FA (2nd run)	FA (3rd run)	average fluorescence anisotropy (FA)	CV
1	1	0.004	0.006	0.007	0.0057	0.0012
2	2	0.01	0.012	0.01	0.011	0.00094
3	4	0.02	0.021	0.02	0.02	0.00047
4	8	0.031	0.029	0.029	0.03	0.00094
5	16	0.039	0.041	0.039	0.040	0.00094
6	32	0.044	0.046	0.046	0.045	0.00094
7	64	0.051	0.052	0.05	0.051	0.00082
8	128	0.053	0.051	0.054	0.053	0.0012
9	256	0.055	0.055	0.054	0.055	0.00047

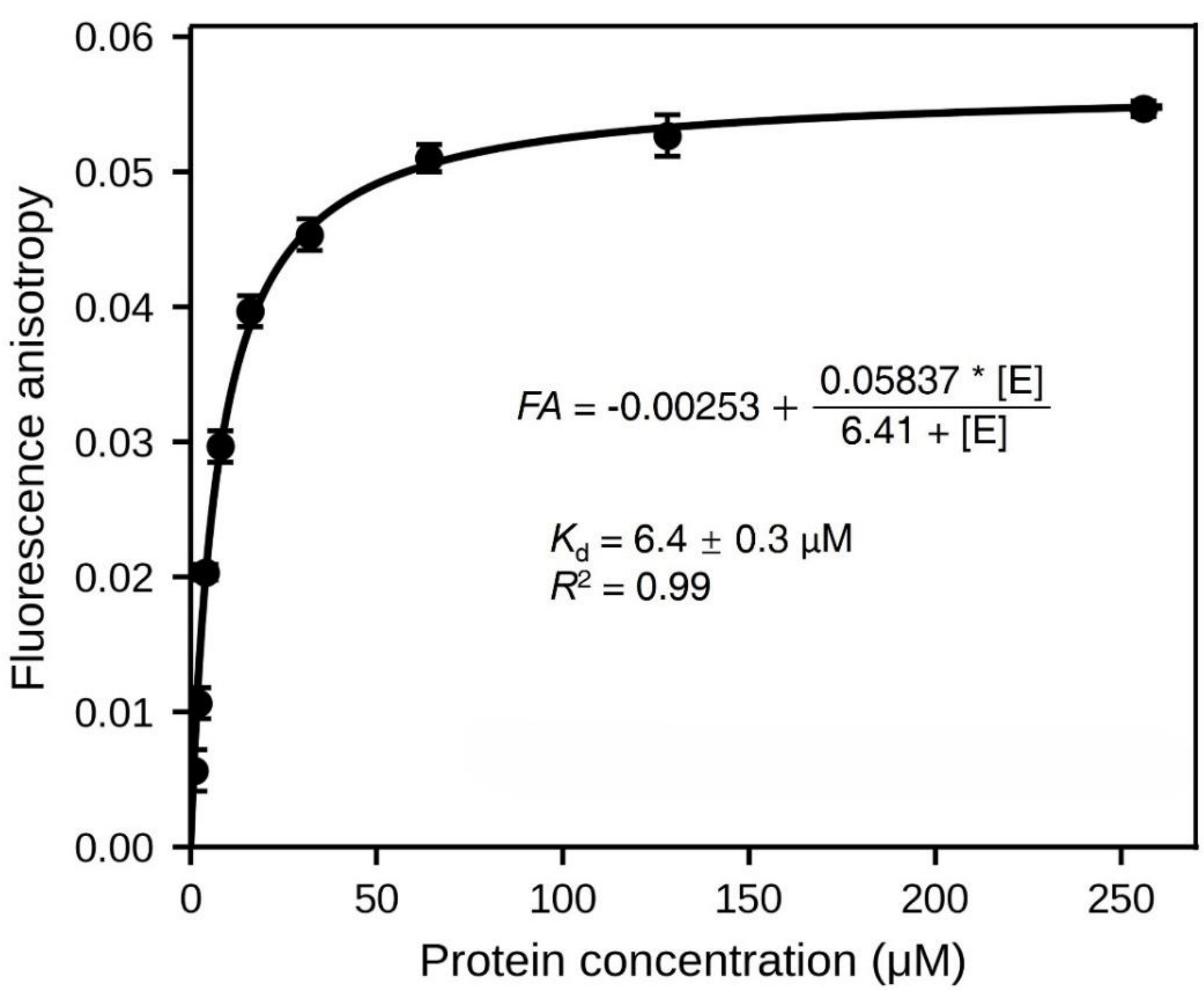

Figure S6. Fluorescence anisotropy titration with *Ape*Pgb W59A Y60G–rhodamine B.

Table S11. Fluorescence anisotropy titration with *Ape*Pgb W59A Y60G–fluorescein.

entry	enzyme concentration (μM)	FA (1st run)	FA (2nd run)	FA (3rd run)	average fluorescence anisotropy (FA)	CV
1	1	0.014	0.018	0.016	0.016	0.0012
2	2	0.019	0.019	0.018	0.019	0.0025
3	4	0.024	0.024	0.024	0.024	0.0021
4	8	0.03	0.031	0.029	0.03	0.0022
5	16	0.038	0.04	0.039	0.039	0.00082
6	32	0.049	0.05	0.049	0.049	0.00047
7	64	0.057	0.058	0.057	0.057	0.00047
8	128	0.063	0.064	0.062	0.063	0.00094
9	256	0.068	0.069	0.069	0.069	0.00082

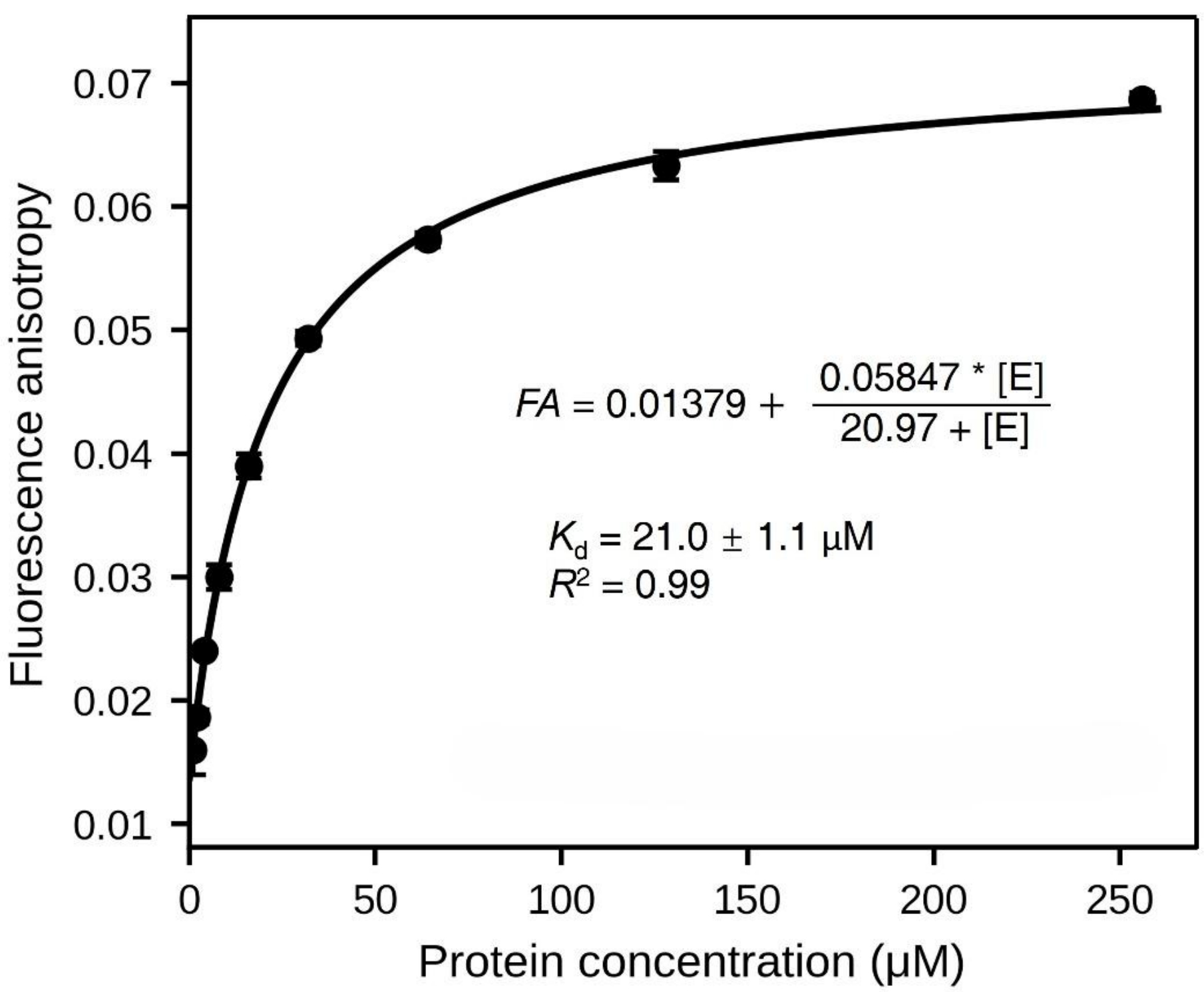

Figure S7. Fluorescence anisotropy titration with *Ape*Pgb W59A Y60G–fluorescein.

Table S12. Fluorescence anisotropy titration with *Ape*Pgb W59A Y60G–rose bengal.

entry	enzyme concentration (μM)	FA (1st run)	FA (2nd run)	FA (3rd run)	average fluorescence anisotropy (FA)	CV
1	1	0.149	0.145	0.147	0.147	0.0016
2	2	0.148	0.155	0.152	0.152	0.0029
3	4	0.159	0.159	0.157	0.158	0.00097
4	8	0.172	0.173	0.169	0.171	0.0017
5	16	0.188	0.185	0.188	0.187	0.0014
6	32	0.209	0.204	0.206	0.206	0.0021
7	64	0.219	0.22	0.219	0.219	0.00047
8	128	0.232	0.233	0.234	0.233	0.00082
9	256	0.24	0.24	0.242	0.24	0.00094

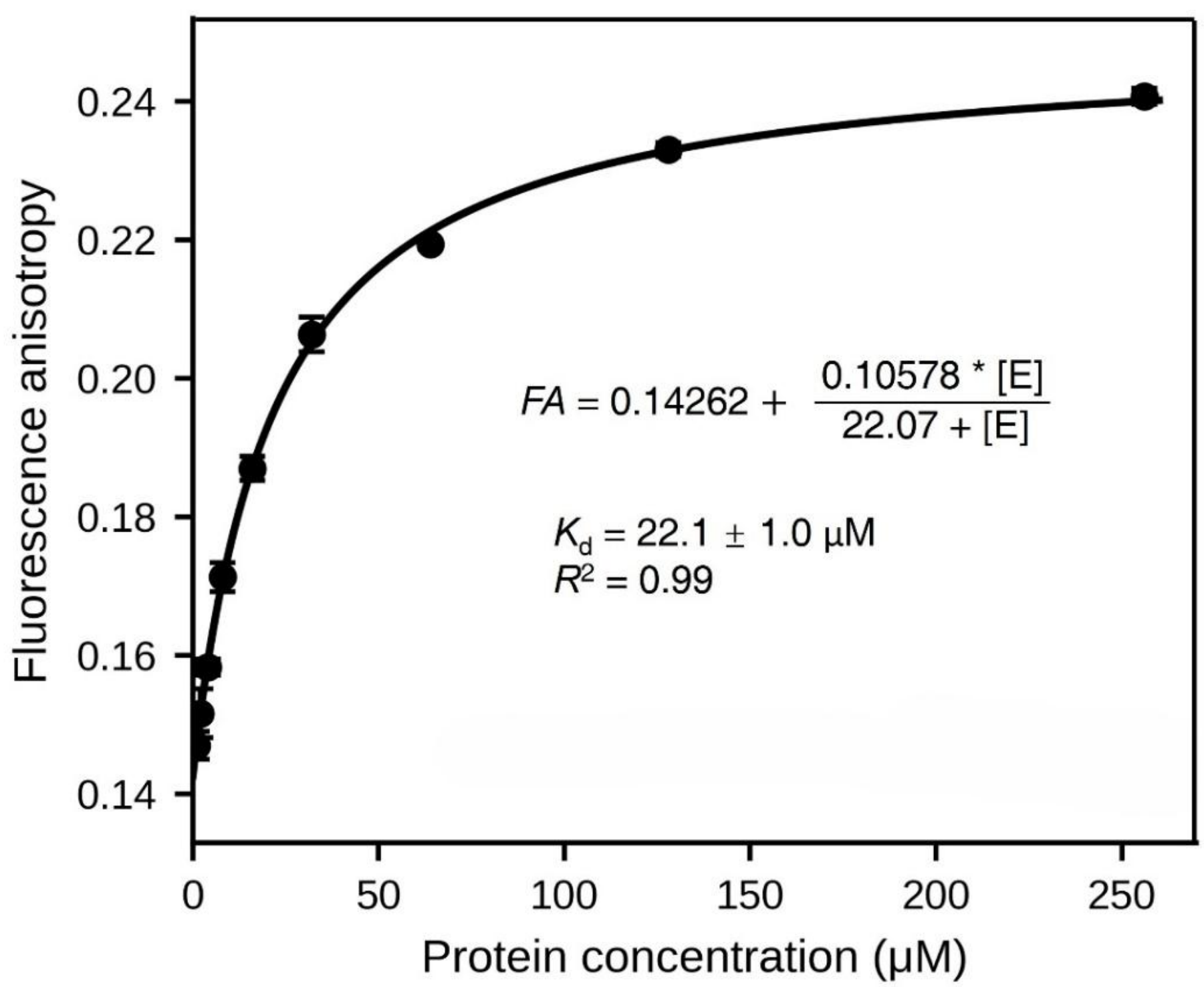

Figure S8. Fluorescence anisotropy titration with *Ape*Pgb W59A Y60G–rose bengal.

Fluorescence anisotropy titration of *Ape*Pgb^{LGGTS}

Experimental procedure. In a 2 mL centrifuge tube, a 500 nM stock solution of Eosin B in 200 mM KPi buffer (pH = 7.4) was prepared. Using a storage solution of *Ape*Pgb^{LGGTS} (200 mM KPi buffer, pH = 7.4), a series of *Ape*Pgb^{LGGTS} solutions with a concentration of 2.0 μM, 4.0 μM, 8.0 μM, 16.0 μM, 32.0 μM, 64.0 μM, 128.0 μM, 256.0 μM, and 512.0 μM were next prepared using 200 mM KPi buffer (pH = 7.4) as the diluent. Anisotropy (vs. single point) is taken with excitation at 515 nm and emission at 560 nm for Eosin B. All fluorescence anisotropy readings are reported as an average of triplicates. Fluorescence anisotropy measurements were carried out using a Tecan Spark Microplate Reader (Part No. 30124664).

To each well of a black 96-well microplate (Greiner Bio-One FLUOTRAC™ 200), were added 50 μL Eosin B stock solution (500 nM in 200 mM KPi buffer, pH = 7.4). 50 μL *Ape*Pgb^{LGGTS} solution of varying concentrations as detailed above was added to each well. The resulting solutions were mixed gently using a multichannel pipette. The final concentration of Eosin B is 250 nM. In this measurement, each data point was measured in triplicated.

Fitting formula: $FA = A + B_{max}[E]/(K_d + [E])$, [E]: enzyme concentration.

Table S13. Data of fluorescence anisotropy titration of *Ape*Pgb^{LGGTS}–eosin B.

entry	enzyme concentration (μM)	FA (1st run)	FA (2nd run)	FA (3rd run)	average fluorescence anisotropy (FA)	CV
1	1	0.078	0.078	0.078	0.078	0
2	2	0.109	0.109	0.106	0.108	0.0014
3	4	0.156	0.157	0.154	0.156	0.0012
4	8	0.217	0.215	0.216	0.216	0.00082
5	16	0.271	0.269	0.266	0.269	0.0020
6	32	0.309	0.308	0.305	0.307	0.0017
7	64	0.331	0.330	0.329	0.330	0.00082
8	128	0.343	0.341	0.344	0.343	0.0012
9	256	0.345	0.343	0.344	0.344	0.00082

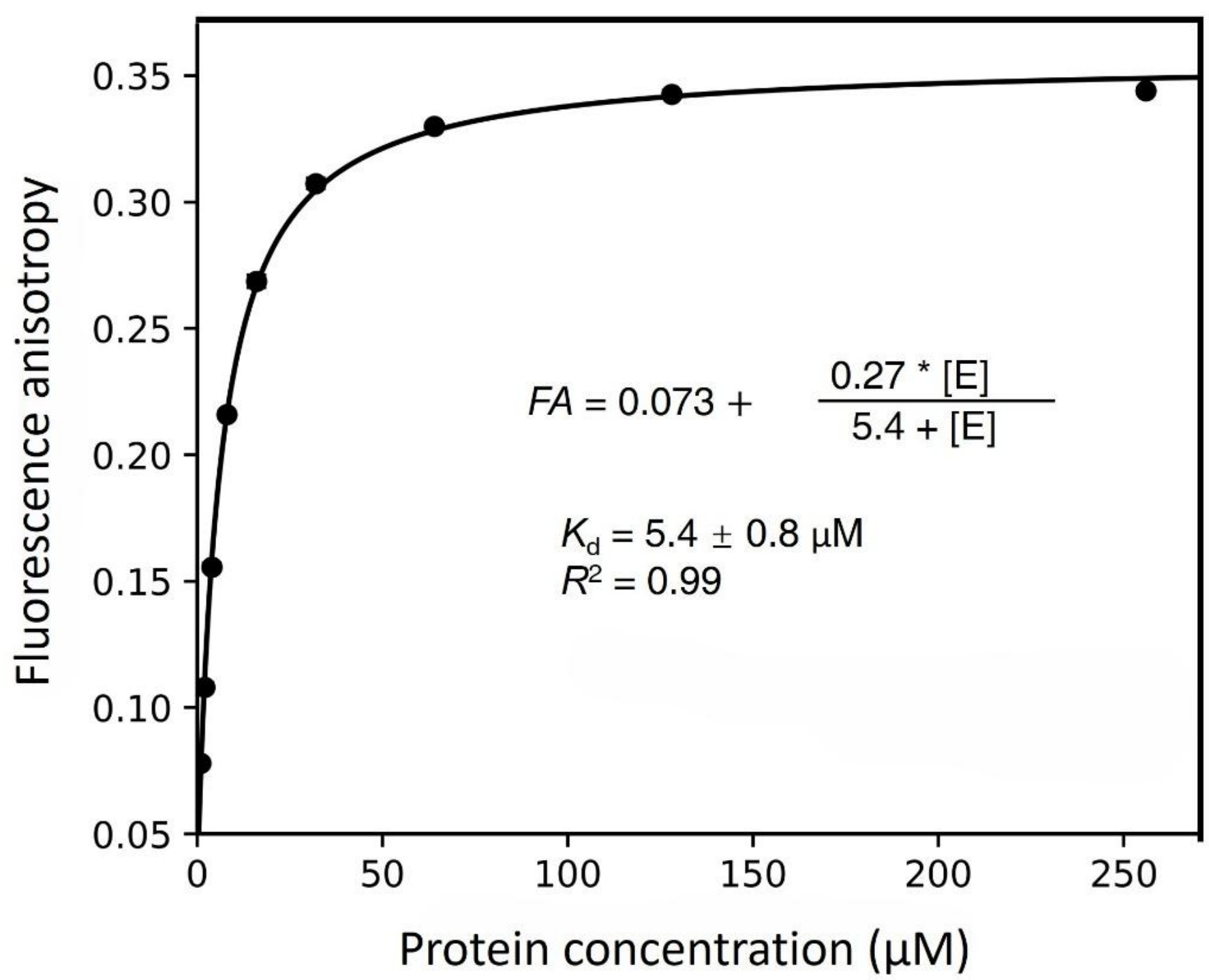

Figure S9. Fluorescence anisotropy titration of *Ape*Pgb^{LGGTS}–eosin B.

Table S14. Data of fluorescence anisotropy titration studies of *Ape*Pgb^{LGGTS}–eosin Y.

entry	enzyme concentration (μM)	FA (1st run)	FA (2nd run)	FA (3rd run)	average fluorescence anisotropy (FA)	CV
1	1	0.045	0.05	0.044	0.046	0.0032
2	2	0.05	0.049	0.051	0.050	0.0010
3	4	0.054	0.057	0.056	0.0557	0.0015
4	8	0.063	0.064	0.064	0.0637	0.0006
5	16	0.068	0.066	0.067	0.0670	0.0010
6	32	0.073	0.072	0.072	0.0703	0.0006
7	64	0.074	0.075	0.073	0.0723	0.0006
8	128	0.074	0.075	0.073	0.0740	0.0010
9	256	0.073	0.074	0.073	0.0733	0.0006

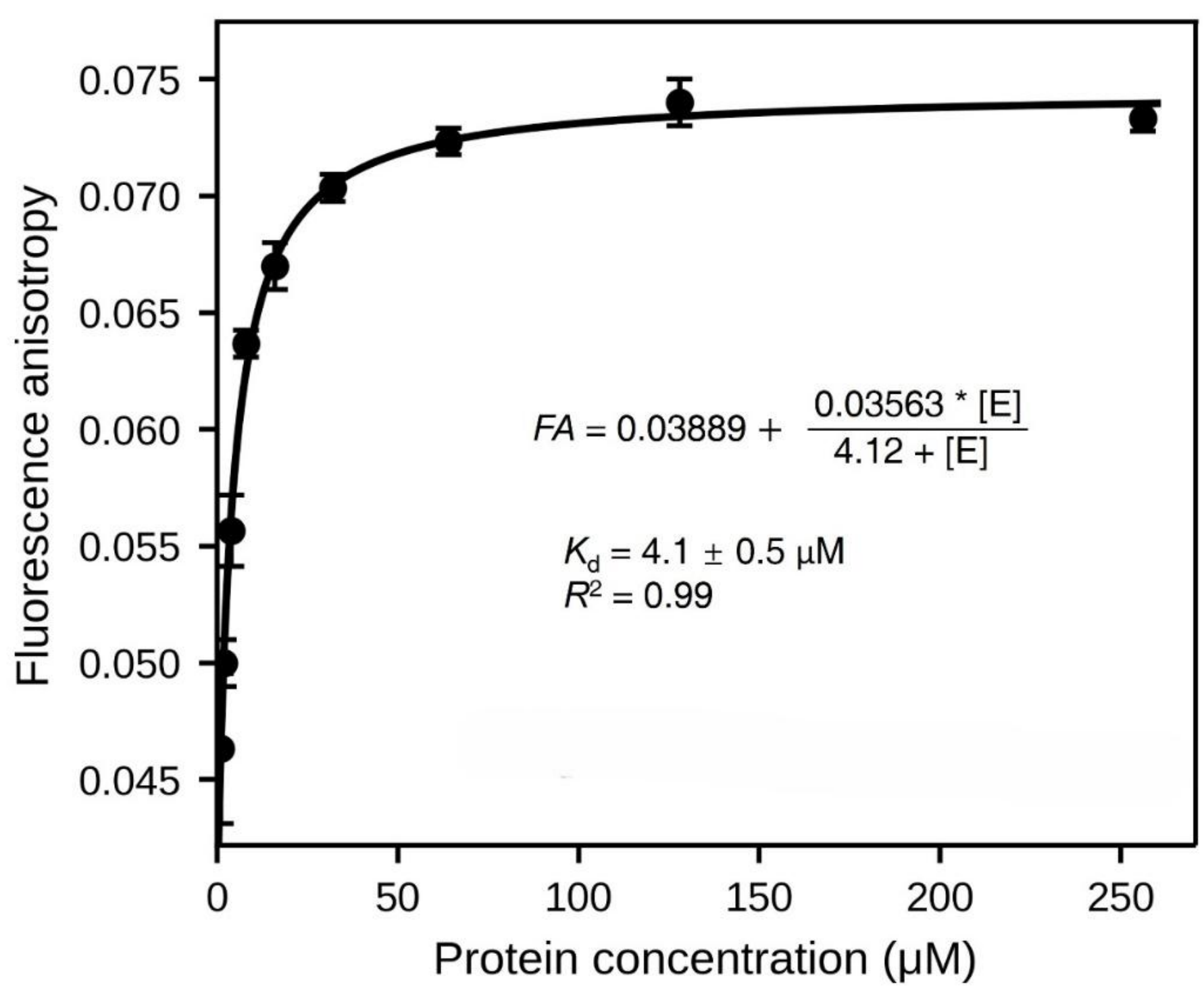

Figure S10. Fluorescence anisotropy titration of *Ape*Pgb^{LGGTS}–eosin Y.

Table S15. Data of fluorescence anisotropy titration studies of *Ape*Pgb^{LGGTS}–rhodamine.

entry	enzyme concentration (μM)	FA (1st run)	FA (2nd run)	FA (3rd run)	average fluorescence anisotropy (FA)	CV
1	1	0.046	0.047	0.046	0.046	0.0006
2	2	0.058	0.054	0.053	0.055	0.0026
3	4	0.067	0.063	0.067	0.0657	0.0023
4	8	0.076	0.076	0.078	0.0767	0.0012
5	16	0.088	0.087	0.089	0.0880	0.0010
6	32	0.104	0.105	0.104	0.1043	0.0006
7	64	0.111	0.112	0.113	0.1120	0.0010
8	128	0.113	0.114	0.114	0.1137	0.0006
9	256	0.113	0.112	0.114	0.1130	0.0010

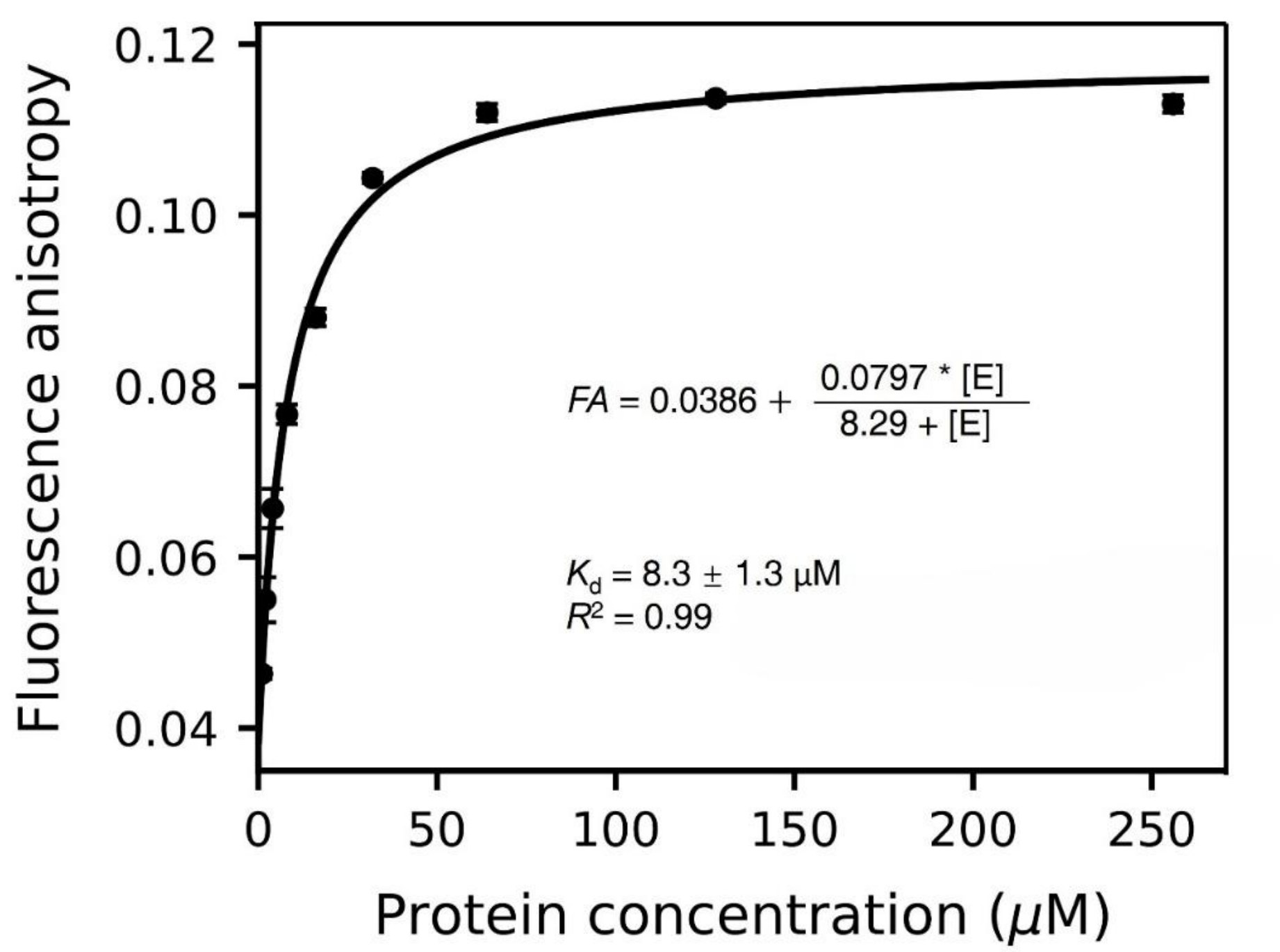

Figure S11. Fluorescence anisotropy titration of *Ape*Pgb^{LGGTS}–rhodamine.

Table S16. Data of fluorescence anisotropy titration studies of of *Ape*Pgb^{LGGTS}–rose bengal.

entry	enzyme concentration (μM)	FA (1st run)	FA (2nd run)	FA (3rd run)	average fluorescence anisotropy (FA)	CV
1	1	0.017	0.017	0.017	0.017	0.0000
2	2	0.019	0.02	0.019	0.019	0.0006
3	4	0.022	0.022	0.025	0.023	0.0017
4	8	0.029	0.029	0.029	0.029	0.0000
5	16	0.034	0.033	0.034	0.0337	0.0006
6	32	0.039	0.039	0.04	0.0393	0.0006
7	64	0.045	0.044	0.043	0.0440	0.0010
8	128	0.047	0.047	0.047	0.0470	0.0000
9	256	0.049	0.048	0.048	0.0483	0.0006

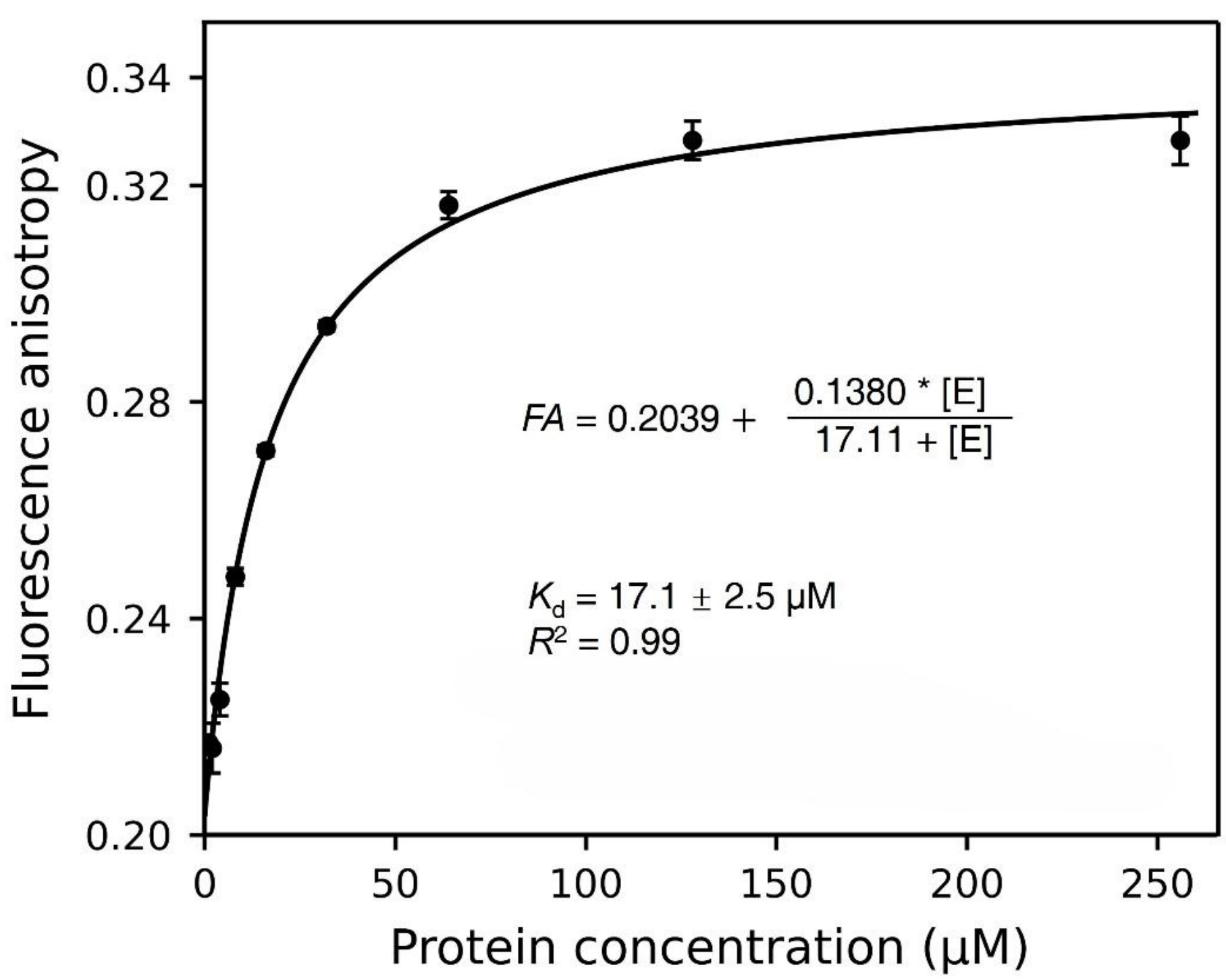

Figure S12. Fluorescence anisotropy titration of *Ape*Pgb^{LGGTS}–rose bengal.

Table S17. Data of fluorescence anisotropy titration studies of of *Ape*Pgb^{LGGTS}–fluorescein.

entry	enzyme concentration (μM)	FA (1st run)	FA (2nd run)	FA (3rd run)	average fluorescence anisotropy (FA)	CV
1	1	0.017	0.017	0.017	0.017	0.0000
2	2	0.019	0.02	0.019	0.019	0.0006
3	4	0.022	0.022	0.025	0.023	0.0017
4	8	0.029	0.029	0.029	0.029	0.0000
5	16	0.034	0.033	0.034	0.0337	0.0006
6	32	0.039	0.039	0.04	0.0393	0.0006
7	64	0.045	0.044	0.043	0.0440	0.0010
8	128	0.047	0.047	0.047	0.0470	0.0000
9	256	0.049	0.048	0.048	0.0483	0.0006

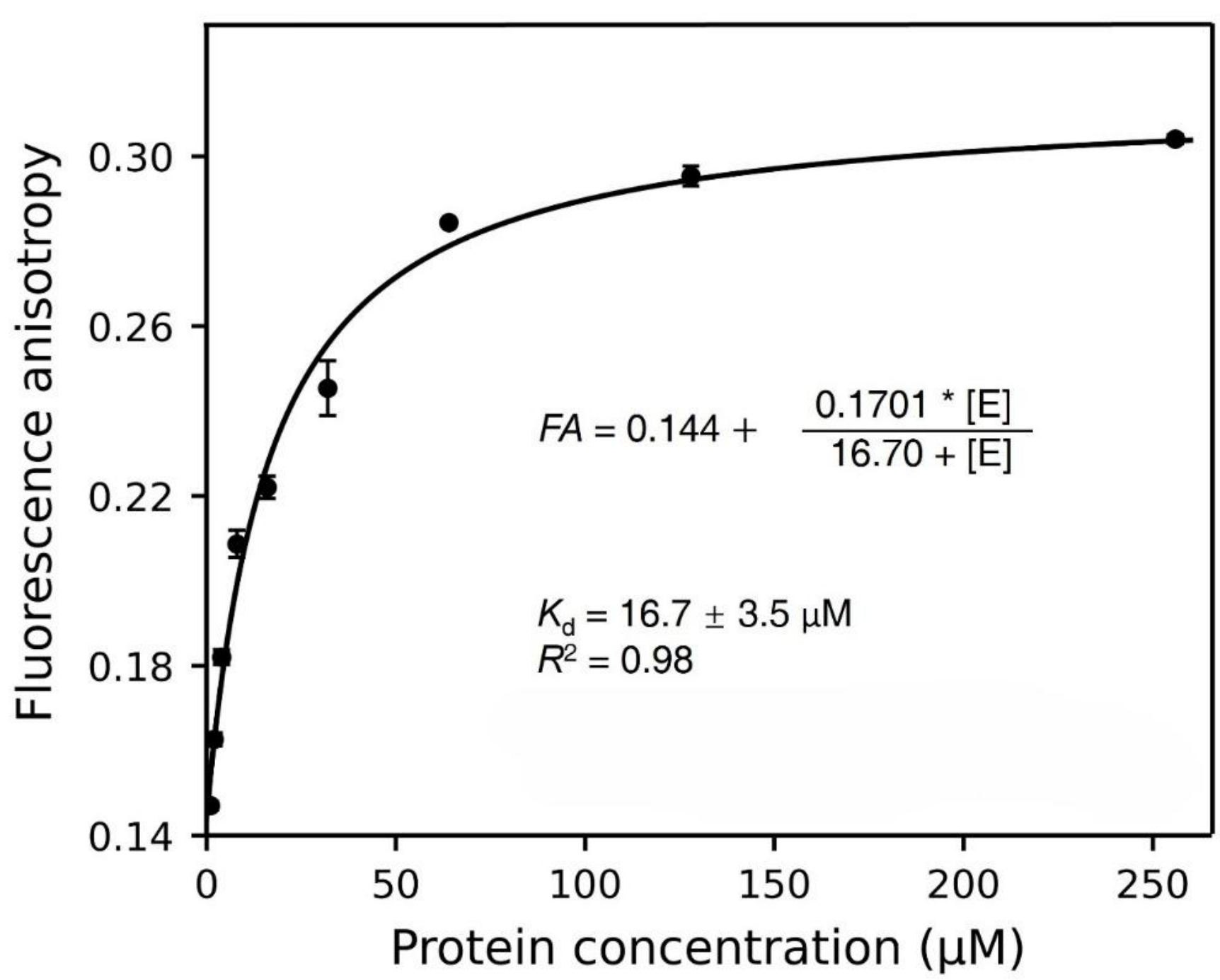

Figure S13. Fluorescence anisotropy titration of *Ape*Pgb^{LGGTS}–fluorescein.

VI. Synthesis and characterization of substrates

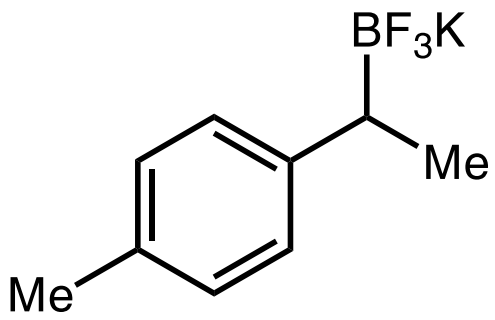

1a

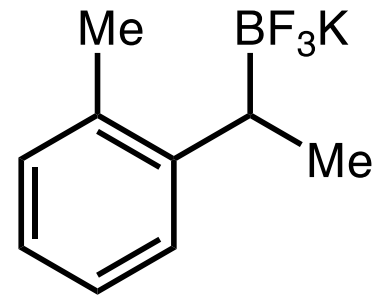

1b

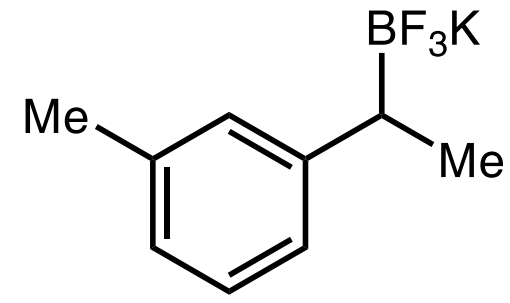

1c

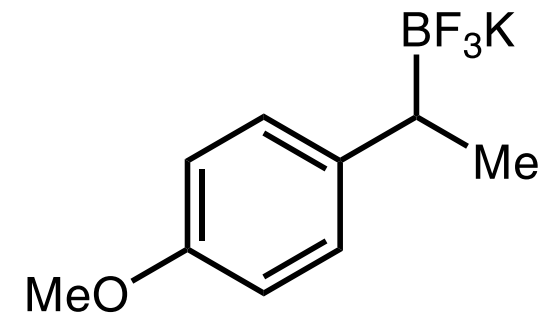

1d

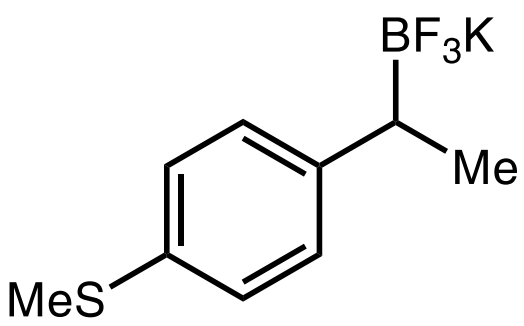

1e

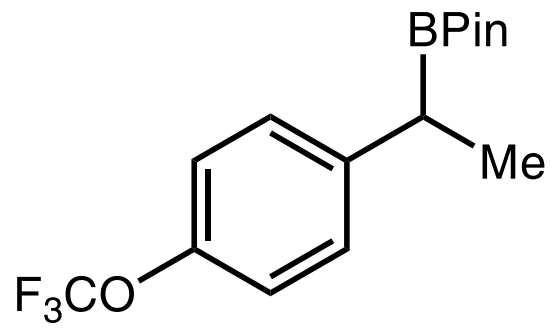

1f

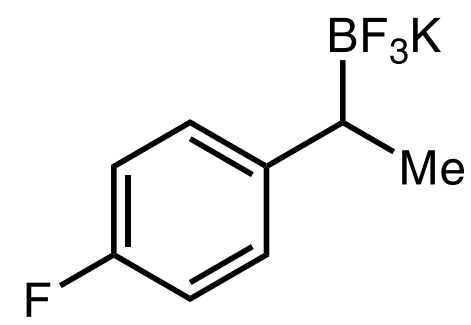

1g

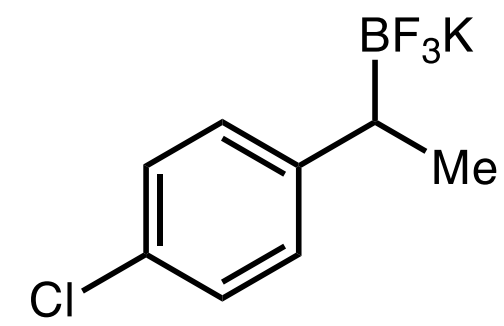

1h

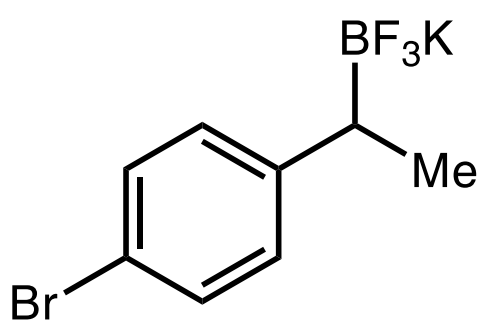

1i

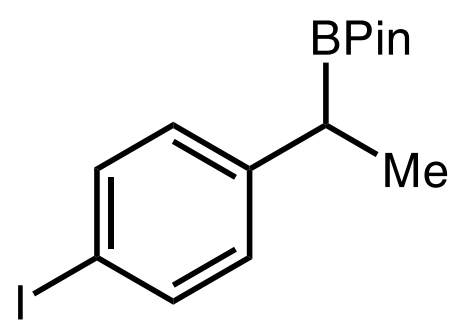

1j

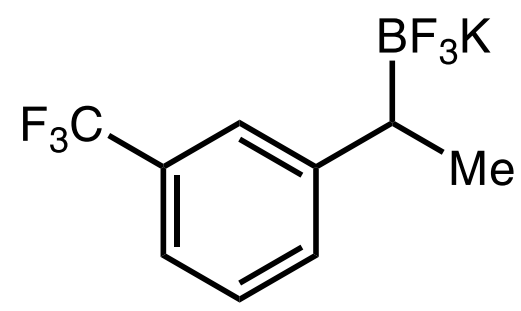

1k

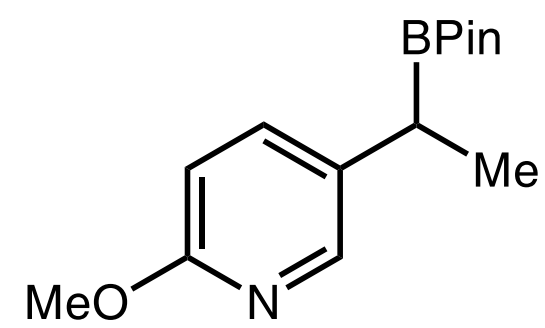

1l

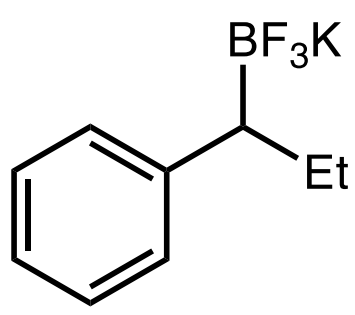

1m

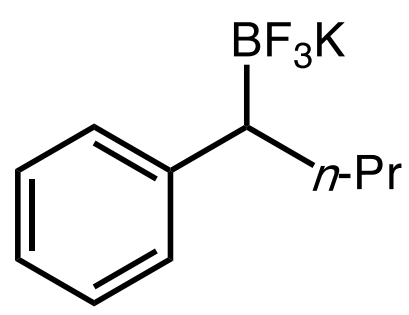

1n

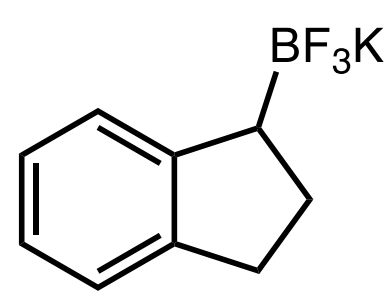

1o

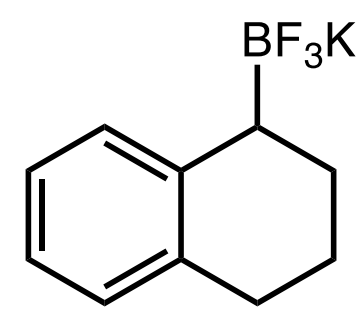

1p

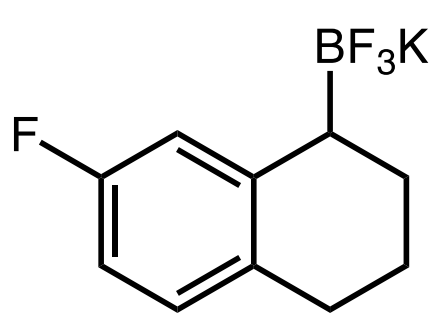

1q

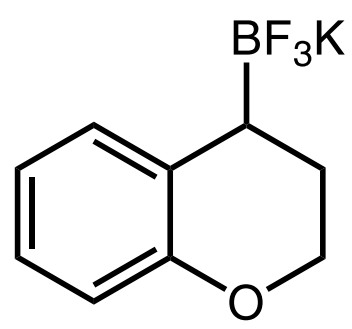

1r

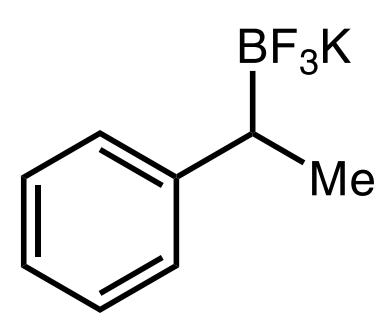

1s

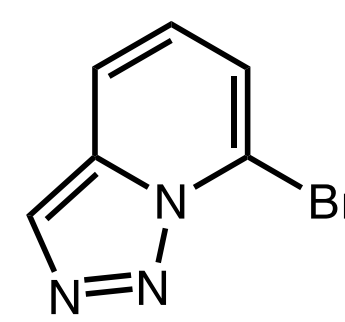

2a

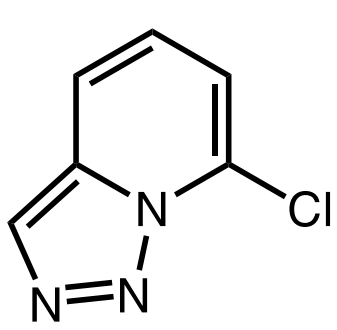

2b

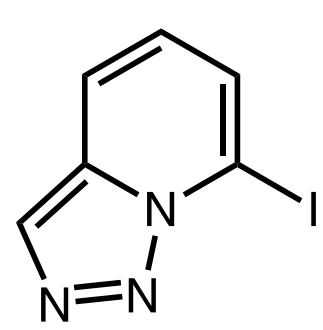

2c

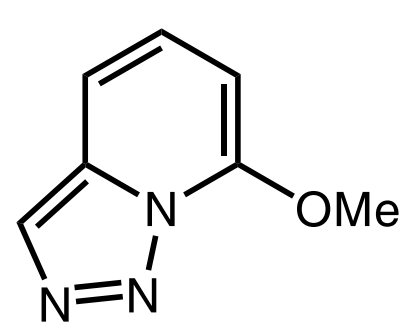

2d

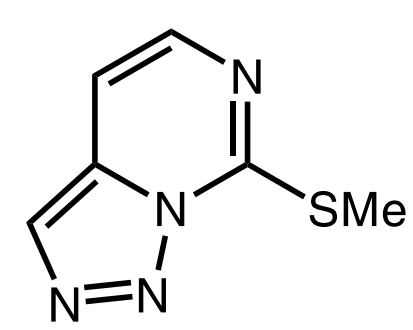

2e

Compounds **1a–1s** and **2a–2d** are known compounds and they were prepared using literature procedures.[7,8] Compounds **2e** is a new compound. This substrate was synthesized according to General procedures **A** as detailed below.

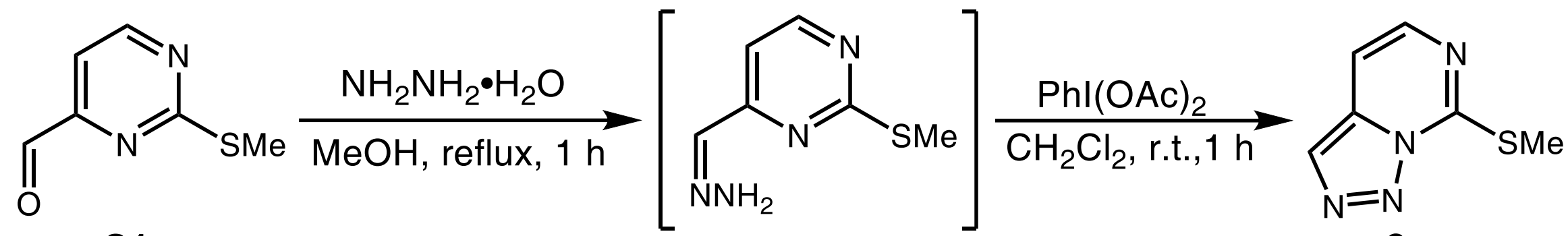

S1 **2e**

General Procedure A: To a 100 mL round bottom flask were added the aldehyde **S1** (6.0 mmol, 0.99 g, 1.0 equiv), NH_2NH_2•H_2O (18.0 mmol, 1.16 g, 3.0 equiv) and MeOH (20 mL). The mixture was heated to reflux at 75 ℃ for 1 h. After cooling to room temperature, the reaction was quenched with sat. NH_4Cl (aq.) and extracted with EtOAc (20 mL × 3). The combined organic layers were dried over $MgSO_4$, filtered through a celite plug, and concentrated *in vacuo* with the aid of a rotary evaporator to afford the crude hydrazone product as an oil. The residue was dissolved in CH_2Cl_2 (20 mL) and cooled to 0 ℃. PhI(OAc)$_2$ (6.6 mmol, 2.2 g, 1.1 equiv) was added slowly, and the mixture was stirred at room temperature for 1 h. The reaction was quenched by the addition of water and extracted with CH_2Cl_2 (20 mL × 3). The combined organic layers were dried over $MgSO_4$ and concentrated *in vacuo* with the aid of a rotary evaporator. The crude product was purified by column chromatography with the aid of Biotage Isolera (100 g Sfär cartridge, 100% hexanes for 2 CV, 0–70% EtOAc in hexanes for 10 CV, then 70% EtOAc/hexanes for 2 CV) to afford the desired product **2e** as a pale yellow solid (0.31 g, 31% yield).

7-(Methylthio)-[1,2,3]triazolo[1,5-c]pyrimidine (2e)

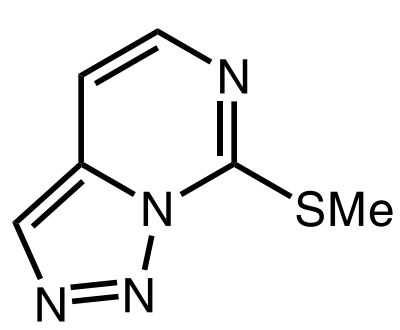

This compound was prepared by following General Procedure A. Purified by Biotage (100 g Sfär cartridge, 100% hexanes for 2 CV, 0–70% EtOAc/hexanes for 10 CV, then 70% EtOAc/hexanes for 2 CV) to afford the product as a pale yellow solid. **^{1}H NMR** (400 MHz, $CDCl_3$) δ: 8.07 (s, 1H), 7.87 (d, J = 8.0 Hz, 1H), 7.38 (d, J = 8.0 Hz, 1H), 2.77(s, 3H) ppm. **^{13}C NMR** (100 MHz, $CDCl_3$) δ: 151.7, 139.3, 134.1, 126.1, 107.3, 13.3 ppm. **HRMS** (GC-MS, EI TOF) calculated for $[C_6H_6N_4S]^+$: 166.0313, found: 166.0317.

VII. Synthesis and characterization of products

General procedure for the synthesis of racemic products

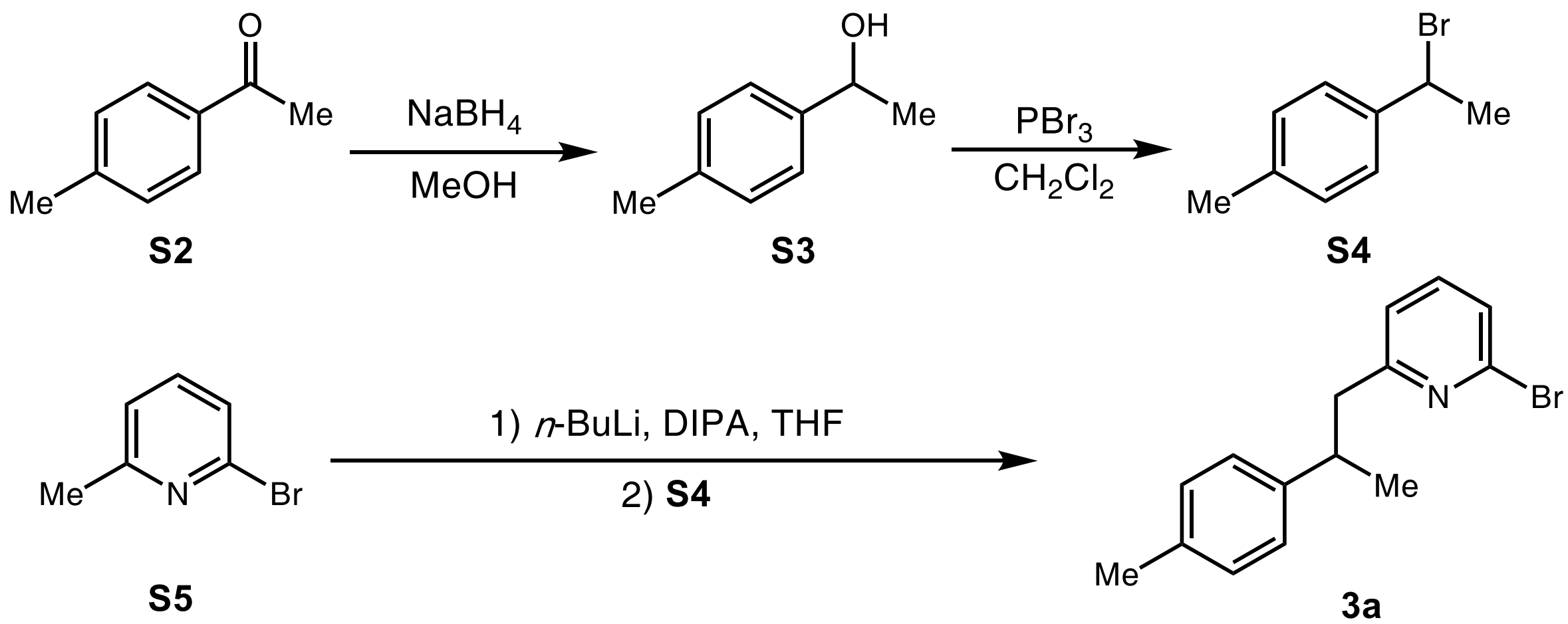

General Procedure B: This procedure was modified from a previously described procedure.[9] In a 100 mL-round bottom flask, ketone **S2** (20 mmol, 2.68 g, 1.0 equiv) was dissolved in MeOH. $NaBH_4$ (22 mmol, 0.83 g, 1.1 equiv) was added slowly at 0 °C. The mixture was stirred at 0 °C for 1 h until TLC analysis indicated complete consumption of **S2**. The reaction was quenched with H_2O and extracted with EtOAc (100 mL × 3). The combined organic layers were washed with brine, dried over $MgSO_4$, and concentrated *in vacuo* with the aid of a rotary evaporator to afford alcohol **S3** as a colorless oil.

To a 100 mL round bottom flask were added **S3** (20 mmol, 1.0 equiv) and CH_2Cl_2 (30 mL). PBr_3 (22 mmol, 6.0 g/2.1 mL, 1.1 equiv) was added to this mixture at 0 °C under a N_2 atmosphere. The mixture was stirred at room temperature for 2 h, then quenched with H_2O and extracted with CH_2Cl_2 (20 mL × 3). The combined organic layers were dried over $MgSO_4$ and concentrated *in vacuo* with the aid of a rotary evaporator to provide the crude product, which was purified by column chromatography with the aid of a Biotage Isolera to afford **S4** as a colorless oil.

A separate 100 mL-round bottom flask was evacuated and backfilled with N_2 three times. Diisopropylamine (5.5 mmol, 0.557 g/0.77 mL, 1.1 equiv) and THF (20 mL) were added. At 0 °C, *n*-BuLi (5.5 mmol, 2.5 M in hexanes, 2.2 mL, 1.1 equiv) was slowly added and the mixture was allowed to stir at 0 °C for 15 min. To this freshly prepared LDA (5.5 mmol, 1.1 equiv), the **S5** (5.0 mmol, 0.86 g, 1.0 equiv) was dissolved in THF (5 mL) was added slowly at –78 °C under a N_2 atmosphere. The resulting mixture was allowed to stir at –78 °C for 2 h. A solution of **S4** (5.5 mmol, 1.1 g, 1.1 equiv) in THF (5 mL) was then added dropwise to the solution at –78 °C. The mixture was warmed to room temperature and allowed to stir for 12 h. The reaction was quenched

with H_2O and extracted with EtOAc (100 mL × 3). The combined organic layers were washed with brine, dried over $MgSO_4$, and concentrated *in vacuo* with the aid of a rotary evaporator. The crude product was purified by column chromatography with the aid of a Biotage Isolera to afford the desired product **3a**.

2-Bromo-6-(2-(*p*-tolyl)propyl)pyridine (3a)

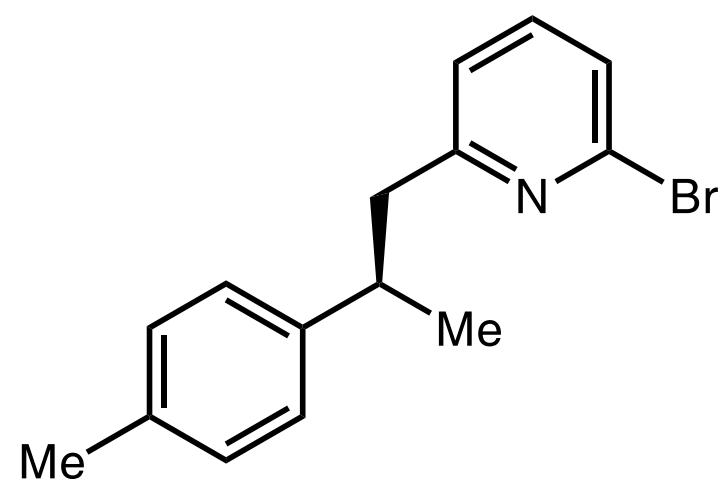

This compound was prepared by following General Procedure B. Purified by Biotage (50 g Sfär cartridge, 100% hexanes for 2 CV, 0–20% EtOAc/hexanes for 10 CV, then 20% EtOAc/hexanes for 2 CV) to afford the product as a colorless oil. **^{1}H NMR** (400 MHz, $CDCl_3$) δ: 7.35 – 7.31 (m, 1H), 7.28 – 7.25 (m, 1H), 7.07 (s, 3H), 6.84 (d, J = 4.0 Hz, 1H), 3.29 – 3.19 (m, 1H), 3.04 – 2.93 (m, 2H), 2.30 (s, 3H), 1.25 (d, J = 8.0 Hz, 3H) ppm. **^{13}C NMR** (100 MHz, $CDCl_3$) δ: 162.6, 143.3, 141.6, 138.4, 135.8, 129.2, 127.0, 125.5,122.6, 46.7, 39.9, 21.8, 21.1 ppm. **HRMS** (GC-MS, EI TOF) calculated for $[C_{15}H_{16}BrN]^+$: 289.0466, found: 289.0459.

HPLC analysis conditions: OJ-H column, 95:5 hexanes: *i*-PrOH, 1.2 mL/min, 40 °C, t_R (major) = 5.4 min, t_R (minor) = 5.1 min.

Yield: run 1: 86%; run 2: 86%; run 3: 85%; average yield: 86%.

Enantioselectivity: run 1: 97 : 3 e.r.; run 2: 97 : 3 e.r.; run 3: 97 : 3 e.r.; average e.r.: 97 : 3.

2-Bromo-6-(2-(*o*-tolyl)propyl)pyridine (3b)

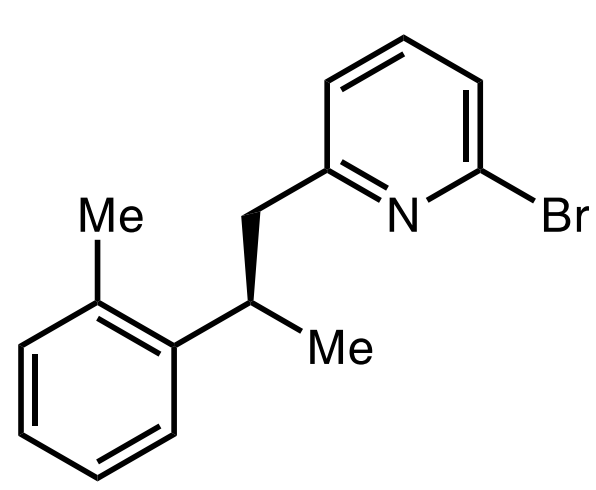

This compound was prepared by following General Procedure B. Purified by Biotage (50 g Sfär cartridge, 100% hexanes for 2 CV, 0–20% EtOAc/hexanes for 10 CV, then 20% EtOAc/hexanes for 2 CV) to afford the product as a colorless oil. **^{1}H NMR** (400 MHz, $CDCl_3$) δ: 7.33 – 7.23 (m, 3H), 7.21 – 7.14 (m, 1H), 7.09 – 7.03 (m, 2H), 6.81 – 6.79 (m, 1H), 3.61 – 3.50 (m, 1H), 3.01 (d, J = 8.0 Hz, 2H), 2.21 (s, 3H), 1.27 (d, J = 4.0 Hz, 3H) ppm. **^{13}C NMR** (100 MHz, $CDCl_3$) δ: 162.5, 144.3, 141.6, 138.4, 135.7, 130.4, 126.3, 125.9, 125.49, 125.48, 122.4, 46.2, 35.1, 21.4, 19.6 ppm. **HRMS** (GC-MS, EI TOF) calculated for $[C_{15}H_{16}BrN]^+$: 289.0466, found: 289.0479.

HPLC analysis conditions: OJ-H column, hexanes, 1.2 mL/min, 40 °C, t_R (major) = 7.6 min, t_R (minor) = 8.1 min.

Yield: run 1: 40%; run 2: 42%; run 3: 41%; average yield: 41%.

Enantioselectivity: run 1: 73 : 27 e.r.; run 2: 73 : 27 e.r.; run 3: 73 : 27 e.r.; average e.r.: 73 : 27.

2-Bromo-6-(2-(*m*-tolyl)propyl)pyridine (3c)

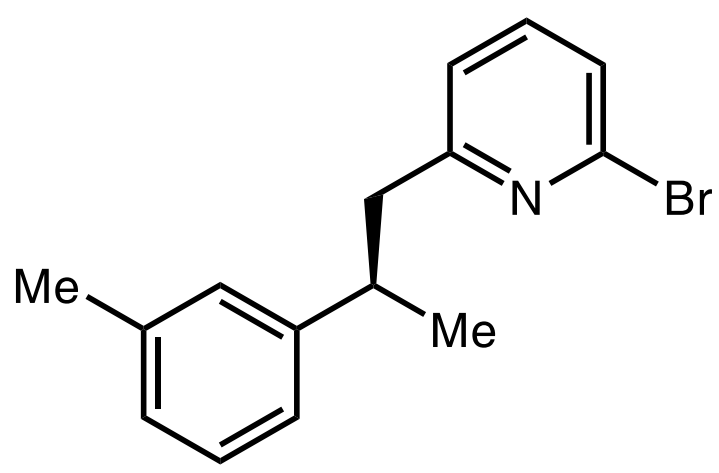

This compound was prepared by following General Procedure B. Purified by Biotage (50 g Sfär cartridge, 100% hexanes for 2 CV, 0–20% EtOAc/hexanes for 10 CV, then 20% EtOAc/hexanes for 2 CV) to afford the product as a colorless oil. **^{1}H NMR** (400 MHz, $CDCl_3$) δ: 7.37 – 7.30 (m, 1H), 7.29 – 7.24 (m, 1H), 7.19 – 7.11 (m, 1H), 7.03 – 6.93 (m, 3H), 6.85 (dd, J_1 = 8.0 Hz, J_2 = 4.0 Hz, 1H), 3.29 – 3.18 (m, 1H), 3.08-2.90 (m, 2H), 2.31 (s, 3H), 1.26 (d, J = 8.0 Hz, 3H) ppm. **^{13}C NMR** (100 MHz, $CDCl_3$) δ: 162.5, 146.3, 141.6, 138.4, 138.0, 128.4, 128.0, 127.0, 125.5, 124.1, 122.6, 46.7, 40.2, 21.62, 21.59 ppm. **HRMS** (GC-MS, EI TOF) calculated for $[C_{15}H_{16}BrN]^+$: 289.0466, found: 289.0466.

HPLC analysis conditions: OJ-H column, hexanes, 1.2 mL/min, 40 °C, t_R (major) = 10.1 min, t_R (minor) = 11.4 min.

Yield: run 1: 67%; run 2: 66%; run 3: 67%; average yield: 67%.

Enantioselectivity: run 1: 85 : 15 e.r.; run 2: 85 : 15 e.r.; run 3: 85 : 15 e.r.; average e.r.: 85 : 15.

2-Bromo-6-(2-(4-methoxyphenyl)propyl)pyridine (3d)

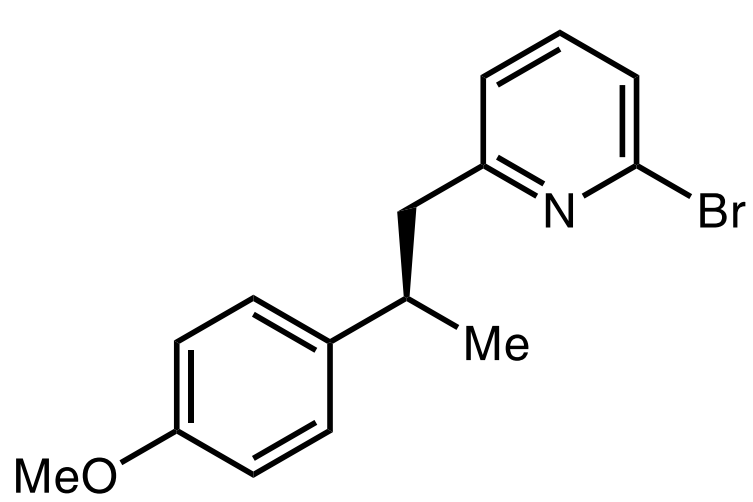

This compound was prepared by following General Procedure B. Purified by Biotage (50 g Sfär cartridge, 100% hexanes for 2 CV, 0–20% EtOAc/hexanes for 10 CV, then 20% EtOAc/hexanes for 2 CV) to afford the product as a colorless oil. **^{1}H NMR** (400 MHz, $CDCl_3$) δ: 7.35 – 7.29 (m, 1H), 7.28 – 7.22 (m, 1H), 7.10 – 7.04 (m, 2H), 6.83 – 6.76 (m, 3H), 3.77 (s, 3H), 3.28 – 3.15 (m, 1H), 2.97 (d, J = 8.0 Hz, 2H), 1.25 (d, J = 8.0 Hz, 3H) ppm. **^{13}C NMR** (100 MHz, $CDCl_3$) δ: 162.6, 158.0, 141.6, 138.4, 138.3, 128.1, 125.5, 122.6, 113.8, 55.4, 47.0, 39.5, 21.9 ppm. **HRMS** (GC-MS, EI TOF) calculated for $[C_{15}H_{16}BrNO]^+$: 305.0415, found: 305.0420.

HPLC analysis conditions: OJ-H column, 99.5:0.5 hexanes: *i*-PrOH, 0.5 mL/min, 25 °C, t_R (major) = 63.3 min, t_R (minor) = 61.7 min.

Yield: run 1: 84%; run 2: 84%; run 3: 83%; average yield: 84%.

Enantioselectivity: run 1: 97 : 3 e.r.; run 2: 97 : 3 e.r.; run 3: 97 : 3 e.r.; average e.r.: 97 : 3.

2-Bromo-6-(2-(4-(methylthio)phenyl)propyl)pyridine (3e)

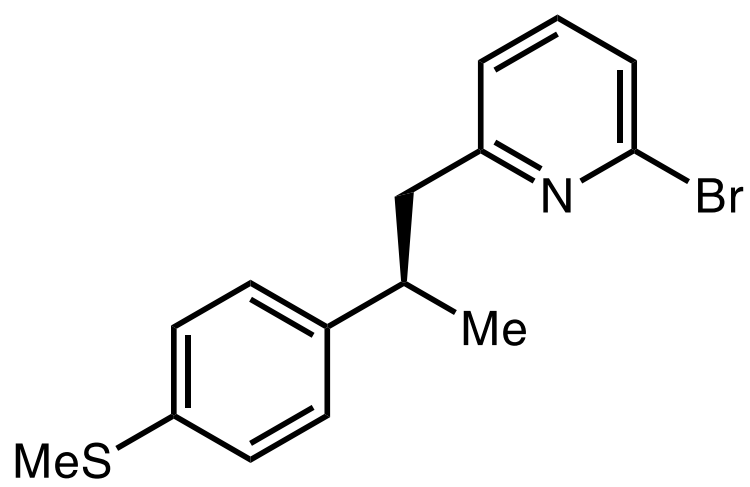

This compound was prepared by following General Procedure B. Purified by Biotage (50 g Sfär cartridge, 100% hexanes for 2 CV, 0–20% EtOAc/hexanes for 10 CV, then 20% EtOAc/hexanes for 2 CV) to afford the product as a colorless oil. **^{1}H NMR** (400 MHz, $CDCl_3$) δ: 7.36 – 7.28 (m, 1H), 7.28 – 7.23 (m, 1H), 7.19 – 7.12 (m, 2H), 7.12 – 7.05 (m, 2H), 6.82 (d, *J* = 8.0 Hz, 1H), 3.32 – 3.19 (m, 1H), 2.98 (d, *J* = 8.0 Hz, 2H), 2.45 (s, 3H), 1.26 (d, *J* = 8.0 Hz, 3H) ppm. **^{13}C NMR** (100 MHz, $CDCl_3$) δ: 162.3, 143.3, 141.6, 138.5, 135.8, 127.7, 127.1, 125.6, 122.6, 46.6, 39.8, 21.7, 16.3 ppm. **HRMS** (GC-MS, EI TOF) calculated for $[C_{15}H_{16}BrNS]^+$: 321.0187, found: 321.0185.

HPLC analysis conditions: OJ-H column, 99.5:0.5 hexanes: *i*-PrOH, 0.5 mL/min, 25 °C, t_R (major) = 68.8 min, t_R (minor) = 65.7 min.

Yield: run 1: 44%; run 2: 47%; run 3: 46%; average yield: 46%.

Enantioselectivity: run 1: 93 : 7 e.r.; run 2: 93 : 7 e.r.; run 3: 93 : 7 e.r.; average e.r.: 93 : 7.

2-Bromo-6-(2-(4-(trifluoromethoxy)phenyl)propyl)pyridine (3f)

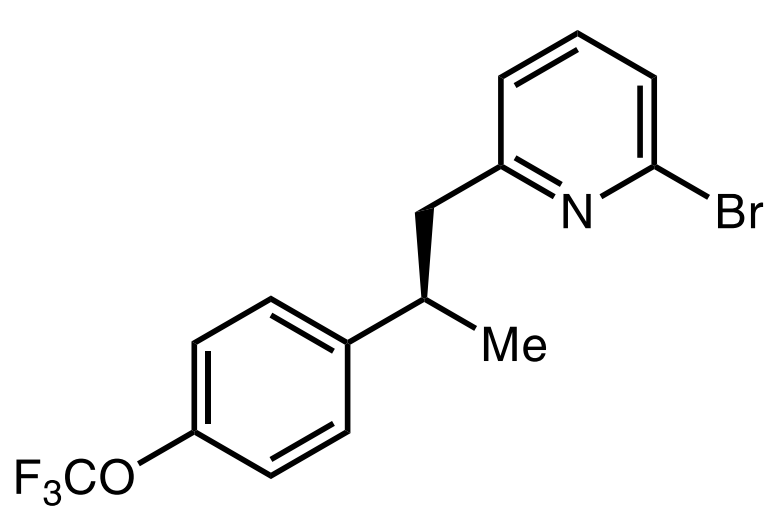

This compound was prepared by following General Procedure B. Purified by Biotage (50 g Sfär cartridge, 100% hexanes for 2 CV, 0–20% EtOAc/hexanes for 10 CV, then 20% EtOAc/hexanes for 2 CV) to afford the product as a colorless oil. **^{1}H NMR** (400 MHz, $CDCl_3$) δ: 7.36 – 7.30 (m, 1H), 7.29 – 7.25 (m, 1H), 7.21 – 7.15 (m, 2H), 7.13 – 7.07 (m, 2H), 6.82 (d, *J* = 8.0 Hz, 1H), 3.38 – 3.26 (m, 1H), 2.98 (d, *J* = 8.0 Hz, 2H), 1.27 (d, *J* = 8.0 Hz, 3H) ppm. **^{13}C NMR** (100 MHz, $CDCl_3$) δ: 161.9, 147.7 (d, *J* = 2.0 Hz), 144.9, 141.7, 138.5, 128.4, 125.7, 122.6, 121.0, 120.6 (q, *J* = 255.0 Hz), 46.6, 39.7, 21.6 ppm. **^{19}F**

NMR (376 MHz, $CDCl_3$) δ: –57.9 ppm. **HRMS** (GC-MS, EI TOF) calculated for $[C_{15}H_{13}BrF_3NO]^+$: 359.0133, found: 359.0139.

HPLC analysis conditions: IA column, hexanes, 1.2 mL/min, 25 °C, t_R (major) = 11.0 min, t_R (minor) = 9.9 min.

Yield: run 1: 78%; run 2: 76%; run 3: 75%; average yield: 76%.

Enantioselectivity: run 1: 98 : 2 e.r.; run 2: 98 : 2 e.r.; run 3: 98 : 2 e.r.; average e.r.: 98 : 2.

2-Bromo-6-(2-(4-fluorophenyl)propyl)pyridine (3g)

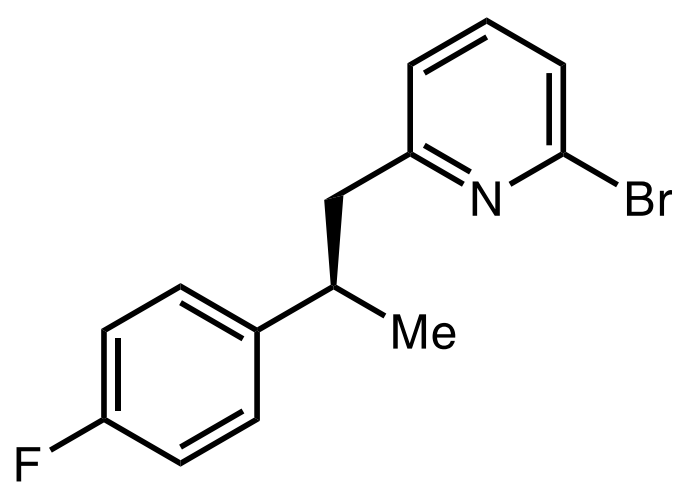

This compound was prepared by following General Procedure B. Purified by Biotage (50 g Sfär cartridge, 100% hexanes for 2 CV, 0–20% EtOAc/hexanes for 10 CV, then 20% EtOAc/hexanes for 2 CV) to afford the product as a colorless oil. **^{1}H NMR** (400 MHz, $CDCl_3$) δ: 7.35 – 7.28 (m, 1H), 7.25 (d, *J* = 8.0 Hz, 1H), 7.14 – 7.05 (m, 2H), 6.97 – 6.87 (m, 2H), 6.79 (d, *J* = 8.0 Hz, 1H), 3.34 – 3.20 (m, 1H), 3.03 – 2.89 (m, 2H), 1.26 (d, *J* = 8.0 Hz, 3H) ppm. **^{13}C NMR** (100 MHz, $CDCl_3$) δ: 162.1, 161.4 (d, *J* = 242.0 Hz), 141.8 (d, *J* = 3.0 Hz), 141.6, 138.4, 128.5 (d, *J* = 7.0 Hz), 125.6, 122.6, 115.2 (d, *J* = 21.0 Hz), 46.8, 39.6, 21.8 ppm. **^{19}F NMR** (376 MHz, $CDCl_3$) δ: –117.2 ppm. **HRMS** (GC-MS, EI TOF) calculated for $[C_{14}H_{13}BrFN]^+$: 293.0215, found: 293.0228.

HPLC analysis conditions: OD-H column, 99.5:0.5 hexanes: *i*-PrOH, 0.5 mL/min, 25 °C, t_R (major) = 22.8 min, t_R (minor) = 22.2 min.

Yield: run 1: 83%; run 2: 84%; run 3: 86%; average yield: 84%.

Enantioselectivity: run 1: 90 : 10 e.r.; run 2: 90 : 10 e.r.; run 3: 90 : 10 e.r.; average e.r.: 90 : 10.

(*R*)-2-Bromo-6-(2-(4-fluorophenyl)propyl)pyridine (3h)

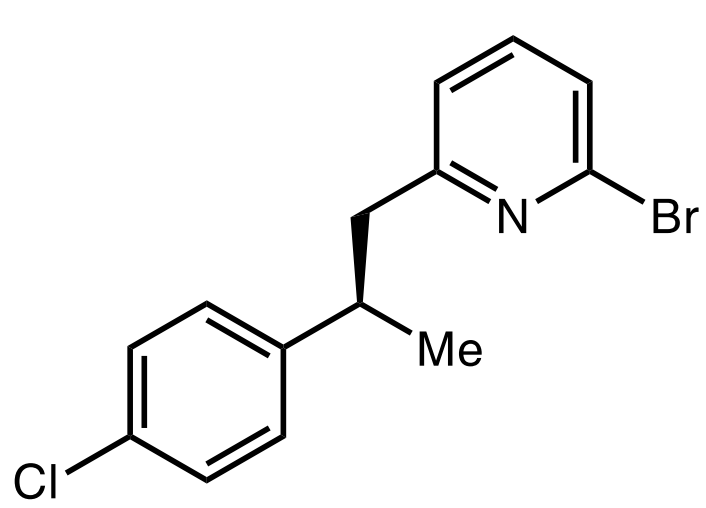

This compound was prepared by following General Procedure B. Purified by Biotage (50 g Sfär cartridge, 100% hexanes for 2 CV, 0–20% EtOAc/hexanes for 10 CV, then 20% EtOAc/hexanes for 2 CV) to afford the product as a colorless oil. **^{1}H NMR** (400 MHz, $CDCl_3$) δ: 7.37 – 7.30 (m, 1H), 7.27 (d, *J* = 4.0 Hz, 1H), 7.24 – 7.18 (m, 2H), 7.12 – 7.05 (m, 2H), 6.80 (d, *J* = 8.0 Hz, 1H), 3.35 – 3.21 (m, 1H), 3.04 – 2.89 (m, 2H), 1.27 (d, *J*

= 4.0 Hz, 3H) ppm. **^{13}C NMR** (100 MHz, $CDCl_3$) δ: 162.0, 144.7, 141.7, 138.5, 131.9, 128.6, 128.55, 125.7, 122.6, 46.6, 39.7, 21.7 ppm. **HRMS** (GC-MS, EI TOF) calculated for $[C_{14}H_{13}BrClN]^+$: 308.9920, found: 308.9918.

HPLC analysis conditions: OJ-H column, hexanes, 0.5 mL/min, 25 °C, t_R (major) = 84.1 min, t_R (minor) = 90.6 min.

Yield: run 1: 89%; run 2: 90%; run 3: 92%; average yield: 90%. **Isolated yield**: 86%, 123.7 mg colorless oil.

Enantioselectivity: run 1: 97 : 3 e.r.; run 2: 97 : 3 e.r.; run 3: 97 : 3 e.r.; average e.r.: 97 : 3.

2-Bromo-6-(2-(4-bromophenyl)propyl)pyridine (3i)

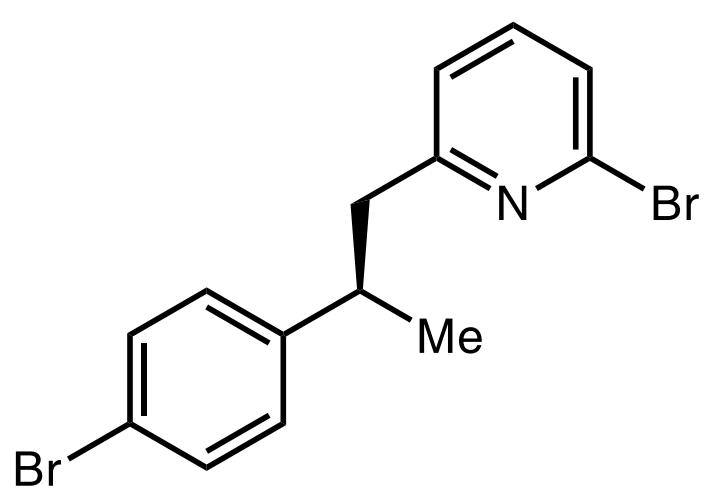

This compound was prepared by following General Procedure B. Purified by Biotage (50 g Sfär cartridge, 100% hexanes for 2 CV, 0–20% EtOAc/hexanes for 10 CV, then 20% EtOAc/hexanes for 2 CV) to afford the product as a colorless oil. **^{1}H NMR** (400 MHz, $CDCl_3$) δ: 7.39 – 7.25 (m, 4H), 7.07 – 6.99 (m, 2H), 6.81 (dd, J_1 = 8.0 Hz, J_2 = 4.0 Hz, 1H), 3.33 – 3.21 (m, 1H), 3.04 – 2.91 (m, 2H), 1.26 (d, J = 8.0 Hz, 3H) ppm. **^{13}C NMR** (100 MHz, $CDCl_3$) δ: 161.9, 145.2, 141.7, 138.5, 131.6, 129.0, 125.7, 122.6, 120.0, 46.5, 39.8, 21.6 ppm. **HRMS** (GC-MS, EI TOF) calculated for $[C_{14}H_{13}Br_2N]^+$: 352.9415, found: 352.9407.

HPLC analysis conditions: OD-H column, 99.5: 0.5: 0.1 hexanes: *i*-PrOH: DEA, 0.5 mL/min, 25 °C, t_R (major) = 21.6 min, t_R (minor) = 21.1 min.

Yield: run 1: 59%; run 2: 64%; run 3: 60%; average yield: 61%.

Enantioselectivity: run 1: 98 : 2 e.r.; run 2: 98 : 2 e.r.; run 3: 98 : 2 e.r.; average e.r.: 98 : 2.

2-Bromo-6-(2-(4-iodophenyl)propyl)pyridine (3j)

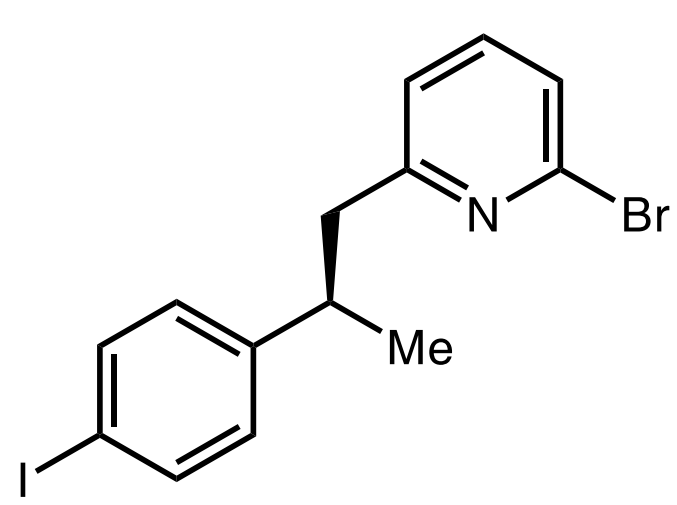

This compound was prepared by following General Procedure B. Purified by Biotage (50 g Sfär cartridge, 100% hexanes for 2 CV, 0–20% EtOAc/hexanes for 10 CV, then 20% EtOAc/hexanes for 2 CV) to afford the product as a colorless oil. **^{1}H NMR** (400 MHz, $CDCl_3$) δ: 7.60 – 7.53 (m, 2H), 7.38 – 7.30 (m, 1H), 7.29 – 7.24 (m, 1H), 6.96 – 6.88 (m, 2H), 6.82 (d, J = 4.0 Hz, 1H), 3.33 – 3.19 (m, 1H), 2.97 (d, J = 4.0 Hz, 2H), 1.25 (d, J

= 8.0 Hz, 3H) ppm. **^{13}C NMR** (100 MHz, $CDCl_3$) δ: 161.9, 145.9, 141.7, 138.6, 137.5, 129.3, 125.7, 122.6, 91.4, 46.4, 39.9, 21.6 ppm. **HRMS** (GC-MS, CI TOF) calculated for $[C_{14}H_{13}BrI]^+$: 400.9276, found:400.9290.

HPLC analysis conditions: OJ-H column, 99: 1: 0.1 hexanes: EtOH: DEA, 1.2 mL/min, 25 °C, t_R (major) = 8.9 min, t_R (minor) = 8.6 min.

Yield: run 1: 44%; run 2: 44%; run 3: 47%; average yield: 45%.

Enantioselectivity: run 1: 97 : 3 e.r.; run 2: 97 : 3 e.r.; run 3: 97 : 3 e.r.; average e.r.: 97 : 3.

2-Bromo-6-(2-(3-(trifluoromethyl)phenyl)propyl)pyridine (3k)

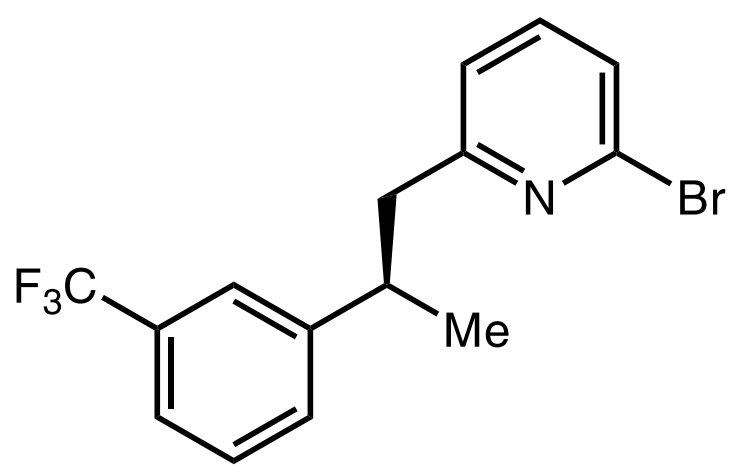

This compound was prepared by following General Procedure B. Purified by Biotage (50 g Sfär cartridge, 100% hexanes for 2 CV, 0–20% EtOAc/hexanes for 10 CV, then 20% EtOAc/hexanes for 2 CV) to afford the product as a colorless oil. **^{1}H NMR** (400 MHz, $CDCl_3$) δ: 7.47 – 7.30 (m, 5H), 7.27 (d, *J* = 8.0 Hz, 1H), 6.82 (d, *J* = 8.0 Hz, 1H), 3.45 – 3.31 (m, 1H), 3.00 (d, *J* = 8.0 Hz, 2H), 1.30 (d, *J* = 8.0 Hz, 3H) ppm. **^{13}C NMR** (100 MHz, $CDCl_3$) δ: 161.7, 147.2, 141.8, 138.5, 130.8 (q, *J* = 32.0 Hz), 130.7, 128.9, 125.8, 124.4 (q, *J* = 271.0 Hz), 123.9 (q, *J* = 5.0 Hz), 123.2 (q, *J* = 4.0 Hz), 122.6, 46.5, 40.1, 21.4 ppm. **^{19}F NMR** (376 MHz, $CDCl_3$) δ: –62.5 ppm. **HRMS** (GC-MS, EI TOF) calculated for $[C_{15}H_{13}BrF_3N]^+$: 343.0183, found: 343.0183.

HPLC analysis conditions: OJ-H column, 99.5: 0.5: 0.1 hexanes: *i*-PrOH: DEA, 0.5 mL/min, 25 °C, t_R (major) = 17.1 min, t_R (minor) = 116.4 min.

Yield: run 1: 61%; run 2: 60%; run 3: 63%; average yield: 61%.

Enantioselectivity: run 1: 71 : 29 e.r.; run 2: 71 : 29 e.r.; run 3: 71 : 29 e.r.; average e.r.: 71 : 29.

5-(1-(6-bromopyridin-2-yl)propan-2-yl)-2-methoxypyridine (3l)

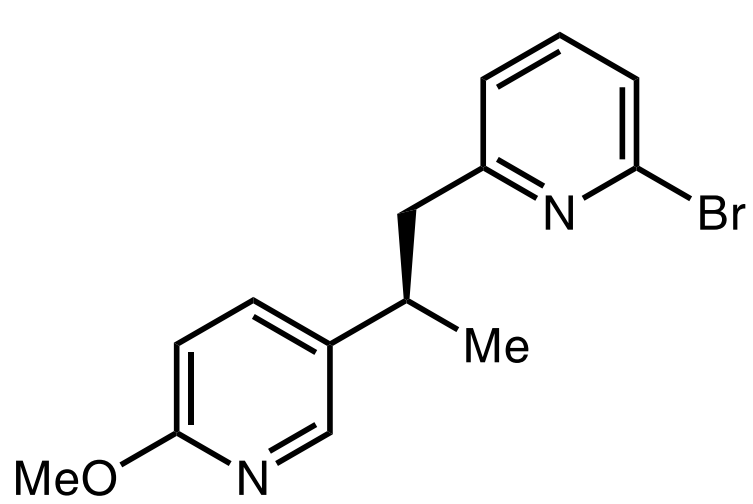

This compound was prepared by following General Procedure B. Purified by Biotage (50 g Sfär cartridge, 100% hexanes for 2 CV, 0–20% EtOAc/hexanes for 10 CV, then 20% EtOAc/hexanes for 2 CV) to afford the product as a colorless oil. **^{1}H NMR** (400 MHz, $CDCl_3$) δ: 7.91 (d, *J* = 4.0 Hz, 1H), 7.41 (dd, J_1 = 8.0 Hz, J_2 = 4.0

Hz, 1H), 7.37 – 7.30 (m, 2H), 7.27 (d, *J* = 4.0 Hz, 1H), 3.88 (s, 3H), 3.33 – 3.21 (m, 1H), 3.03 – 2.89 (m, 2H), 1.27 (d, *J* = 4.0 Hz, 3H) ppm. **^{13}C NMR** (100 MHz, $CDCl_3$) δ: 162.9, 161.8, 145.3, 141.8, 138.5, 137.6, 134.0, 125.7, 122.6, 110.7, 53.5, 46.5, 37.0, 21.7 ppm. **HRMS** (GC-MS, CI TOF) calculated for $[C_{14}H_{15}BrN_2O]^+$: 306.0368, found: 306.0357.

HPLC analysis conditions: OD-H column, 99.5: 0.5 hexanes: *i*-PrOH, 0.5 mL/min, 40 °C, t_R (major) = 14.0 min, t_R (minor) = 13.5 min.

Yield: run 1: 80%; run 2: 80%; run 3: 81%; average yield: 80%.

Enantioselectivity: run 1: 95 : 5 e.r.; run 2: 95 : 5 e.r.; run 3: 95 : 5 e.r.; average e.r.: 95 : 45

2-Bromo-6-(2-(4-bromophenyl)propyl)pyridine (3m)

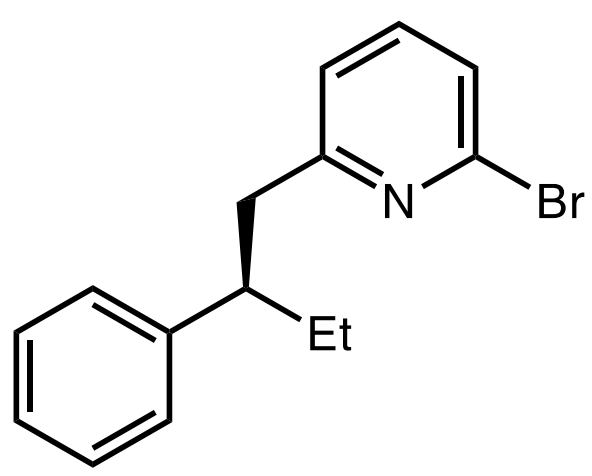

This compound was prepared by following General Procedure B. Purified by Biotage (50 g Sfär cartridge, 100% hexanes for 2 CV, 0–20% EtOAc/hexanes for 10 CV, then 20% EtOAc/hexanes for 2 CV) to afford the product as a colorless oil. **^{1}H NMR** (400 MHz, $CDCl_3$) δ: 7.31 – 7.18 (m, 4H), 7.18 – 7.06 (m, 3H), 6.73 (dd, J_1 = 8.0 Hz, J_2 = 0.31 Hz, 1H), 3.18 – 3.05 (m, 1H), 3.05 – 2.90 (m, 2H), 1.84 – 1.58 (m, 2H), 0.77 (t, *J* = 8.0 Hz, 3H) ppm. **^{13}C NMR** (100 MHz, $CDCl_3$) δ: 162.5, 144.2, 141.5, 138.3, 128.4, 128.0, 126.3, 125.4, 122.5, 48.0, 45.4, 29.0, 12.1 ppm. **HRMS** (GC-MS, EI TOF) calculated for $[C_{15}H_{16}BrN]^+$: 289.0466, found: 289.0468.

HPLC analysis conditions: OJ-H column, 90:10 hexanes: *i*-PrOH, 1.2 mL/min, 25 °C, t_R (major) = 6.1 min, t_R (minor) = 7.8 min.

Yield: run 1: 83%; run 2: 86%; run 3: 85%; average yield: 85%.

Enantioselectivity: run 1: 91 : 9 e.r.; run 2: 91 : 9 e.r.; run 3: 91 : 9 e.r.; average e.r.: 91 : 9.

2-Bromo-6-(2-phenylpentyl)pyridine (3n)

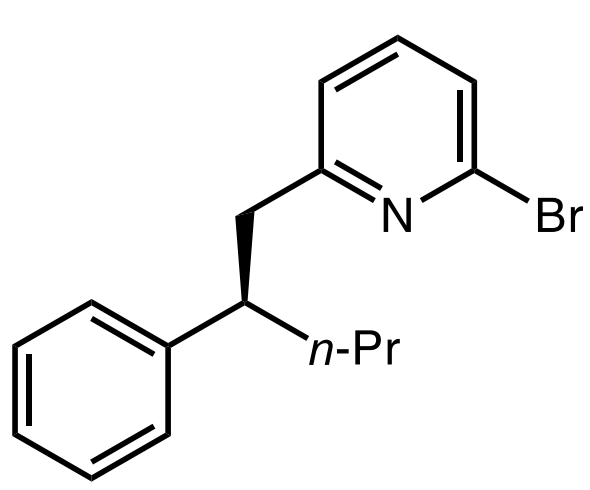

This compound was prepared by following General Procedure B. Purified by Biotage (50 g Sfär cartridge, 100% hexanes for 2 CV, 0–20% EtOAc/hexanes for 10 CV, then 20% EtOAc/hexanes for 2 CV) to afford the product as a colorless oil. **^{1}H NMR** (400 MHz, $CDCl_3$) δ: 7.29 – 7.18 (m, 4H), 7.17 – 7.05 (m, 3H), 6.73 (dd, J_1 = 8.0 Hz, J_2 = 0.31 Hz, 1H),

3.18 – 3.05 (m, 1H), 3.05 – 2.90 (m, 2H), 1.84 – 1.58 (m, 2H), 0.77 (t, J = 8.0 Hz, 3H) ppm. **^{13}C NMR** (100 MHz, $CDCl_3$) δ: 162.5, 144.2, 141.5, 138.3, 128.4, 128.0, 126.3, 125.4, 122.5, 48.0, 45.4, 29.0, 12.1 ppm. **HRMS** (GC-MS, EI TOF) calculated for $[C_{16}H_{18}BrN]^+$: 303.0623, found: 303.0626.

HPLC analysis conditions: OJ-H column, 90:10 hexanes: *i*-PrOH, 1.2 mL/min, 25 °C, t_R (major) = 5.2 min, t_R (minor) = 8.6 min.

Yield: run 1: 66%; run 2: 66%; run 3: 67%; average yield: 66%.

Enantioselectivity: run 1: 82 : 18 e.r.; run 2: 82 : 18 e.r.; run 3: 82 : 18 e.r.; average e.r.: 82 : 18.

2-Bromo-6-((2,3-dihydro-1H-inden-1-yl)methyl)pyridine (3o)

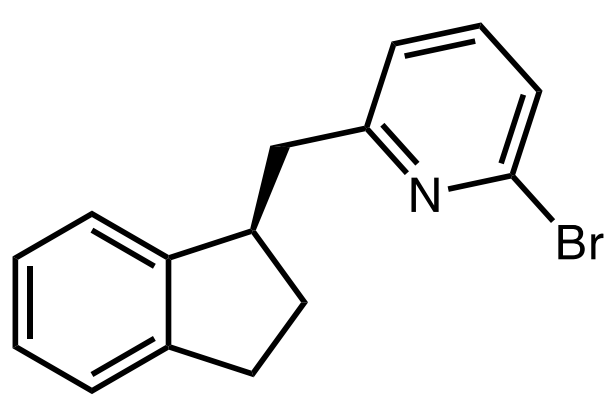

This compound was prepared by following General Procedure B. Purified by Biotage (50 g Sfär cartridge, 100% hexanes for 2 CV, 0–20% EtOAc/hexanes for 10 CV, then 20% EtOAc/hexanes for 2 CV) to afford the product as a pale yellow solid. **^{1}H NMR** (400 MHz, $CDCl_3$) δ: 7.48 – 7.41 (m, 1H), 7.35 (d, J = 4.0 Hz, 1H), 7.23 (d, J = 8.0 Hz, 1H), 7.19 – 3.09 (m, 2H), 7.09 – 7.02 (m, 2H), 3.74 – 3.63 (m, 1H), 3.27 (dd, J_1 = 16.0 Hz, J_2 = 8.0 Hz, 1H), 2.98 – 2.77 (m, 3H), 2.24 – 2.10 (m, 1H), 1.85 – 1.72 (m, 1H) ppm. **^{13}C NMR** (100 MHz, $CDCl_3$) δ: 162.6, 146.5, 144.1, 141.9, 138.6, 126.7, 126.2, 125.7, 124.7, 123.9, 122.6, 45.0, 43.5, 32.0, 31.3 ppm. **HRMS** (GC-MS, EI TOF) calculated for $[C_{15}H_{14}BrN]^+$: 287.0310, found: 287.0308.

HPLC analysis conditions: OJ-H column, 99.5:0.5:0.1 hexanes: *i*-PrOH: DEA, 1.2 mL/min, 25 °C, t_R (major) = 10.2 min, t_R (minor) = 10.9 min.

Yield: run 1: 89%; run 2: 89%; run 3: 88%; average yield: 89%.

Enantioselectivity: run 1: 82 : 18 e.r.; run 2: 82 : 18 e.r.; run 3: 82 : 18 e.r.; average e.r.: 82 : 18.

2-Bromo-6-((1,2,3,4-tetrahydronaphthalen-1-yl)methyl)pyridine (3p)

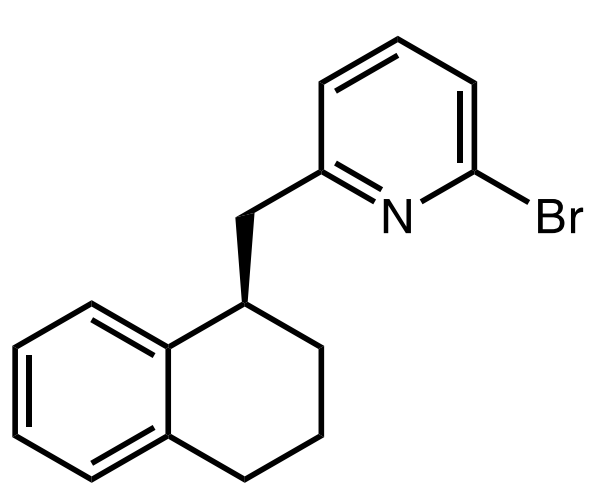

This compound was prepared by following General Procedure B. Purified by Biotage (50 g Sfär cartridge, 100% hexanes for 2 CV, 0–20% EtOAc/hexanes for 10 CV, then 20% EtOAc/hexanes for 2 CV) to afford the product as a pale yellow solid. **^{1}H NMR** (400 MHz, $CDCl_3$) δ: 7.47 –

7.41 (m, 1H), 7.34 (d, J = 8.0 Hz, 1H), 7.17 – 7.06 (m, 4H), 7.03 (d, J = 4.0 Hz, 1H), 3.43 – 3.33 (m, 1H), 3.22 (dd, J_1 = 16.0 Hz, J_2 = 8.0 Hz, 1H), 2.96 – 2.86 (m, 1H), 2.86 – 2.69 (m, 2H), 1.92 – 1.84 (m, 1H), 1.79 – 1.67 (m, 2H), 1.67 – 1.53 (m, 1H), ppm. **^{13}C NMR** (100 MHz, $CDCl_3$) δ: 162.7, 141.9, 140.0, 138.5, 137.2, 129.3, 128.9, 126.0, 125.7, 122.9, 45.3, 38.0, 29.7, 27.4, 19.5 ppm. **HRMS** (GC-MS, EI TOF) calculated for $[C_{16}H_{16}BrN]^+$: 301.0466, found: 301.0462.

HPLC analysis conditions: OJ-H column, 90:10 hexanes: *i*-PrOH, 1.2 mL/min, 25 °C, t_R (major) = 4.8 min, t_R (minor) = 5.3 min.

Yield: run 1: 84%; run 2: 83%; run 3: 83%; average yield: 83%.

Enantioselectivity: run 1: 97 : 3 e.r.; run 2: 97 : 3 e.r.; run 3: 97 : 3 e.r.; average e.r.: 97 : 3.

2-Bromo-6-((7-fluoro-1,2,3,4-tetrahydronaphthalen-1-yl)methyl)pyridine (3q)

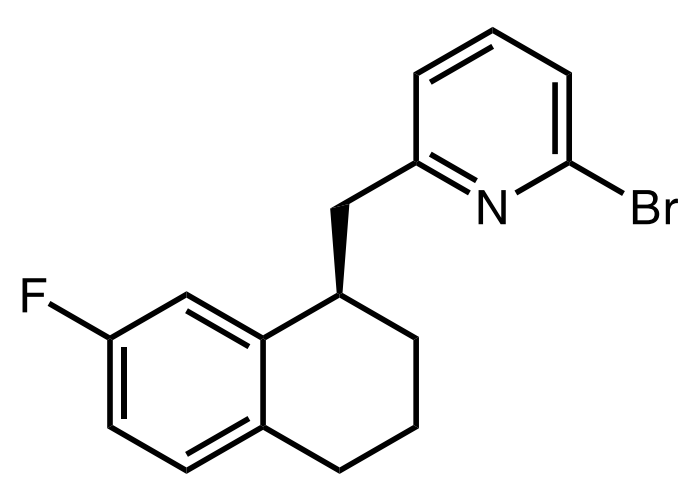

This compound was prepared by following General Procedure B. Purified by Biotage (50 g Sfär cartridge, 100% hexanes for 2 CV, 0–20% EtOAc/hexanes for 10 CV, then 20% EtOAc/hexanes for 2 CV) to afford the product as a colorless oil. **^{1}H NMR** (400 MHz, $CDCl_3$) δ: 7.48 – 7.41 (m, 1H), 7.34 (d, J = 8.0 Hz, 1H), 7.07 – 7.00 (m, 2H), 6.85 – 6.76 (m, 2H), 3.40 – 3.30 (m, 1H), 3.18 (dd, J_1 = 12.0 Hz, J_2 = 4.0 Hz, 1H), 2.87 (dd, J_1 = 12.0 Hz, J_2 = 8.0 Hz, 1H), 2.81 – 2.65 (m, 2H), 1.93 – 1.79 (m, 1H), 1.77 – 1.65 (m, 2H), 1.62 – 1.51 (m, 1H) ppm. **^{13}C NMR** (100 MHz, $CDCl_3$) δ: 162.2, 159.8, 142.0, 141.9, 138.7, 132.7 (d, J = 2.0 Hz), 130.5 (d, J = 8.0 Hz), 125.8, 122.9, 114.9 (d, J = 21.0 Hz), 113.1 (d, J = 21.0 Hz), 45.1, 38.1 (d, J = 1.0 Hz), 29.1, 27.2, 19.7 ppm. **HRMS** (GC-MS, EI TOF) calculated for $[C_{16}H_{15}BrFN]^+$: 319.0372, found: 319.0374.

HPLC analysis conditions: OJ-H column, 99.5: 0.5: 0.1 hexanes: *i*-PrOH: DEA, 1.2 mL/min, 25 °C, t_R (major) = 9.3 min, t_R (minor) = 10.9 min.

Yield: run 1: 81%; run 2: 81%; run 3: 80%; average yield: 81%.

Enantioselectivity: run 1: 92 : 8 e.r.; run 2: 92 : 8 e.r.; run 3: 92 : 8 e.r.; average e.r.: 92 : 8.

2-Bromo-6-(chroman-4-ylmethyl)pyridine (3r)

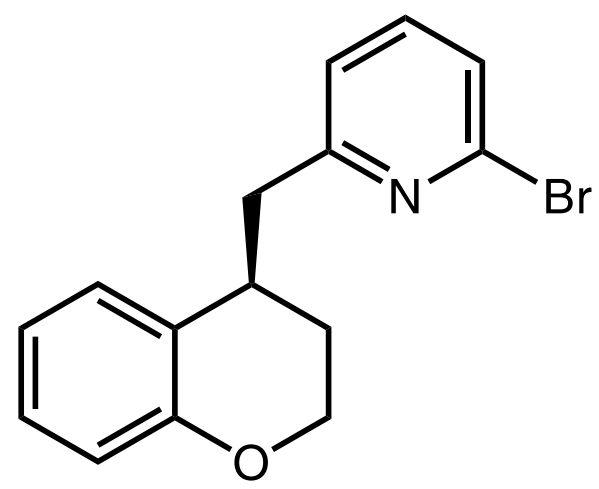

This compound was prepared by following General Procedure B. Purified by Biotage (50 g Sfär cartridge, 100% hexanes for 2 CV, 0–10% EtOAc/hexanes for 10 CV, then 10% EtOAc/hexanes for 2 CV) to afford the product as a colorless oil. **^{1}H NMR** (400 MHz, $CDCl_3$) δ: 7.50 – 7.42 (m, 1H), 7.36 (d, J = 8.0 Hz, 1H), 7.14 – 7.07 (m, 1H), 7.07 – 7.01 (m, 2H), 6.86 – 6.79 (m, 2H), 4.27 – 4.13 (m, 2H), 3.46 – 3.34 (m, 1H), 3.29 (dd, J_1 = 16.0 Hz, J_2 = 8.0 Hz, 1H), 2.90 (dd, J_1 = 12.0 Hz, J_2 = 8.0 Hz, 1H), 2.03 – 1.91 (m, 1H), 1.84 – 1.72 (m, 1H) ppm. **^{13}C NMR** (100 MHz, $CDCl_3$) δ: 161.6, 154.7, 142.0, 138.7, 129.2, 127.8, 126.0, 125.4, 123.0, 120.3, 117.1, 63.4, 44.7, 33.9, 26.8 ppm. **HRMS** (GC-MS, EI TOF) calculated for $[C_{15}H_{14}BrNO]^+$: 303.0259, found: 303.0331.

HPLC analysis conditions: OJ-H column, 99.5:0.5:0.1 hexanes: *i*-PrOH: DEA, 1.2 mL/min, 25 °C, t_R (major) = 25.0 min, t_R (minor) = 27.5 min.

Yield: run 1: 89%; run 2: 88%; run 3: 91%; average yield: 89%.

Enantioselectivity: run 1: 98 : 2 e.r.; run 2: 98 : 2 e.r.; run 3: 98 : 2 e.r.; average e.r.: 98 : 2.

2-Chloro-6-(2-(*p*-tolyl)propyl)pyridine (3s)

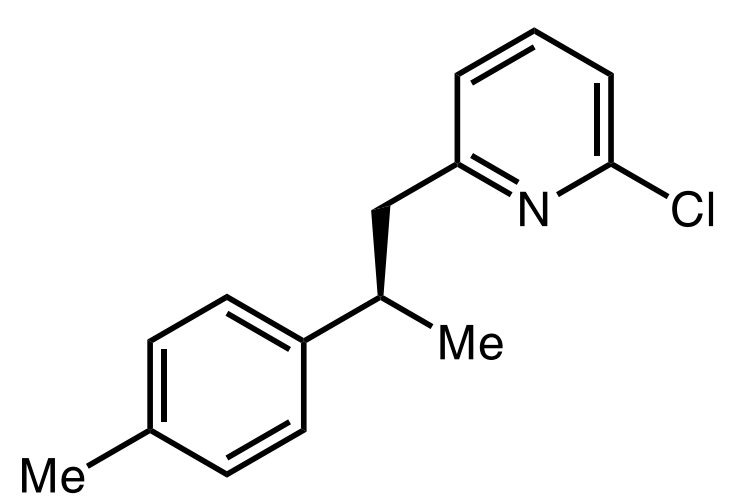

This compound was prepared by following General Procedure B. Purified by Biotage (50 g Sfär cartridge, 100% hexanes for 2 CV, 0–20% EtOAc/hexanes for 10 CV, then 20% EtOAc/hexanes for 2 CV) to afford the product as a colorless oil. **^{1}H NMR** (400 MHz, $CDCl_3$) δ: 7.47 – 7.41 (m, 1H), 7.14 – 7.02 (m, 5H), 6.85 – 6.78 (m, 1H), 3.32 – 3.18 (m, 1H), 3.06 – 2.92 (m, 2H), 2.30 (s, 3H), 1.25 (d, J = 8.0 Hz, 3H) ppm. **^{13}C NMR** (100 MHz, $CDCl_3$) δ: 162.0, 150.8, 143.3, 138.7, 135.7, 129.2, 127.0, 122.2, 121.6, 46.8, 39.9, 21.8, 21.1 ppm. **HRMS** (GC-MS, EI TOF) calculated for $[C_{15}H_{16}ClN]^+$: 245.0971, found: 245.0977.

HPLC analysis conditions: OJ-H column, 95:5 hexanes: *i*-PrOH, 1.2 mL/min, 40 °C, t_R (major) = 5.0 min, t_R (minor) = 4.8 min.

Yield: run 1: 81%; run 2: 80%; run 3: 81%; average yield: 81%.

Enantioselectivity: run 1: 98 : 2 e.r.; run 2: 98 : 2 e.r.; run 3: 98 : 2 e.r.; average e.r.: 98 : 2.

2-Iodo-6-(2-(*p*-tolyl)propyl)pyridine (3t)

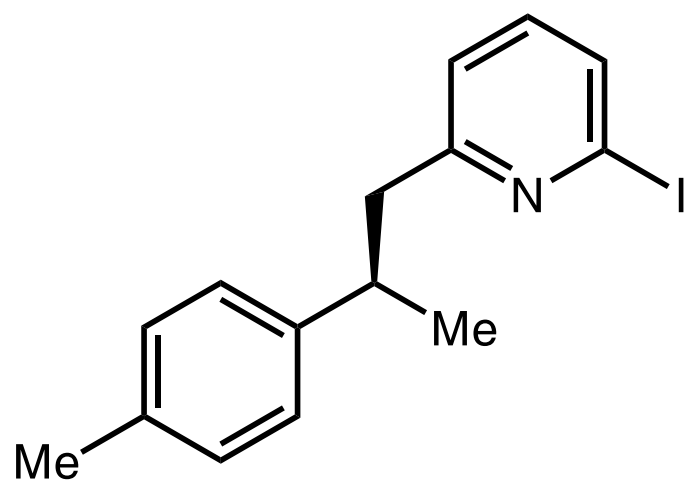

This compound was prepared by following General Procedure B. Purified by Biotage (50 g Sfär cartridge, 100% hexanes for 2 CV, 0–20% EtOAc/hexanes for 10 CV, then 20% EtOAc/hexanes for 2 CV) to afford the product as a colorless oil. **^{1}H NMR** (400 MHz, $CDCl_3$) δ: 7.49 (d, J = 8.0 Hz, 1H), 7.12 – 7.03 (m, 5H), 7.83 (d, J = 8.0 Hz, 1H), 3.28 – 3.16 (m, 1H), 3.03 – 2.91 (m, 2H), 2.30 (s, 3H), 1.25 (d, J = 8.0 Hz, 3H) ppm. **^{13}C NMR** (100 MHz, $CDCl_3$) δ: 163.2, 143.3, 137.5, 135.7, 132.4, 129.2, 127.1, 122.9, 117.7, 46.8, 39.9, 21.8, 21.1 ppm. **HRMS** (GC-MS, EI TOF) calculated for $[C_{15}H_{16}IN]^+$: 337.0322, found: 337.0312.

HPLC analysis conditions: OJ-H column, 95:5 hexanes: *i*-PrOH, 1.2 mL/min, 40 °C, t_R (major) = 5.9 min, t_R (minor) = 5.5 min.

Yield: run 1: 34%; run 2: 37%; run 3: 41%; average yield: 37%.

Enantioselectivity: run 1: 91 : 9 e.r.; run 2: 91 : 9 e.r.; run 3: 91 : 9 e.r.; average e.r.: 91 : 9.

2-Methoxy-6-(2-(*p*-tolyl)propyl)pyridine (3u)

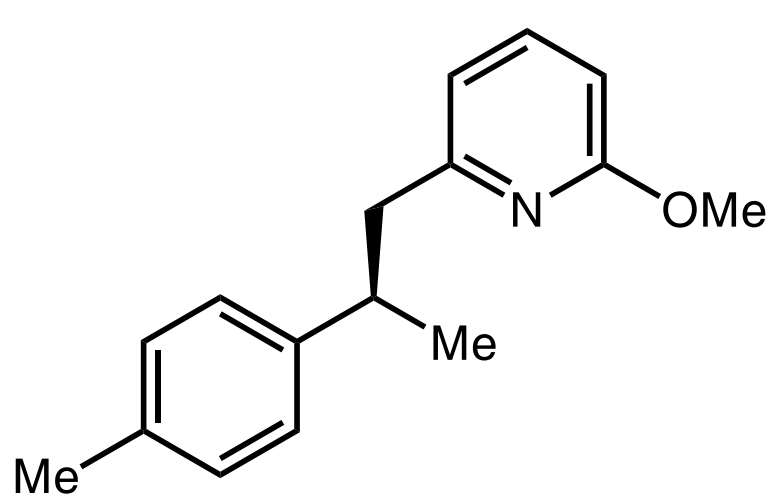

This compound was prepared by following General Procedure B. Purified by Biotage (50 g Sfär cartridge, 100% hexanes for 2 CV, 0–20% EtOAc/hexanes for 10 CV, then 20% EtOAc/hexanes for 2 CV) to afford the product as a colorless oil. **^{1}H NMR** (400 MHz, $CDCl_3$) δ: 7.44 – 7.35 (m, 1H), 7.17 – 7.03 (m, 4H), 6.60 – 6.48 (m, 2H), 3.92 (s, 3H), 3.38 – 3.24 (m, 1H), 3.04 – 2.81 (m, 2H), 2.32 (s, 3H), 1.26 (d, J = 8.0 Hz, 3H) ppm. **^{13}C NMR** (100 MHz, $CDCl_3$) δ: 163.7, 158.7, 144.2, 138.6, 135.4, 129.1, 127.0, 116.2, 107.4, 53.4, 46.7, 39.5, 21.8, 21.1 ppm. **HRMS** (GC-MS, EI TOF) calculated for $[C_{16}H_{19}NO]^+$: 241.1467, found: 251.1458.

HPLC analysis conditions: OJ-H column, hexanes, 0.5 mL/min, 40 °C, t_R (major) = 17.9 min, t_R (minor) = 19.3 min.

Yield: run 1: 71%; run 2: 70%; run 3: 73%; average yield: 71%.

Enantioselectivity: run 1: 98 : 2 e.r.; run 2: 98 : 2 e.r.; run 3: 98 : 2 e.r.; average e.r.: 98 : 2.

2-(Methylthio)-4-(2-(*p*-tolyl)propyl)pyrimidine (3v)

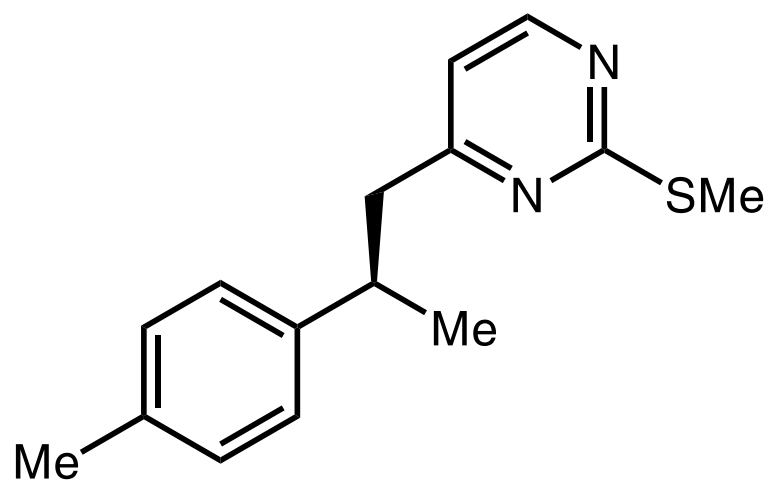

This compound was prepared by following General Procedure B. Purified by Biotage (50 g Sfär cartridge, 100% hexanes for 2 CV, 0–20% EtOAc/hexanes for 10 CV, then 20% EtOAc/hexanes for 2 CV) to afford the product as a colorless oil. **^{1}H NMR** (400 MHz, $CDCl_3$) δ: 8.29 (d, *J* = 4.0 Hz, 1H), 7.07 (s, 4H), 6.58 (d, *J* = 8.0 Hz, 1H), 3.34 – 3.20 (m, 1H), 3.00 – 2.82 (m, 2H), 2.55 (s, 3H), 2.30 (s, 3H), 1.28 (d, *J* = 4.0 Hz, 3H) ppm. **^{13}C NMR** (100 MHz, $CDCl_3$) δ: 172.3, 169.6, 156.7, 142.9, 135.9, 129.2, 126.9, 116.3, 46.4, 39.1, 22.0, 21.1, 14.2 ppm. **HRMS** (GC-MS, EI TOF) calculated for $[C_{15}H_{18}N_2S]^+$: 258.1191, found: 258.1190.

HPLC analysis conditions: OJ-H column, 99.5: 0.5: 0.1 hexanes: *i*-PrOH: DEA, 1.2 mL/min, 25 °C, t_R (major) = 12.9 min, t_R (minor) = 12.1 min.

Yield: run 1: 83%; run 2: 86%; run 3: 84%; average yield: 84%.

Enantioselectivity: run 1: 83 : 17 e.r.; run 2: 83 : 17 e.r.; run 3: 84 : 16 e.r.; average e.r.: 83 : 17.

(*R*)-2-Methoxy-6-(2-phenylpropyl)pyridine (3w)

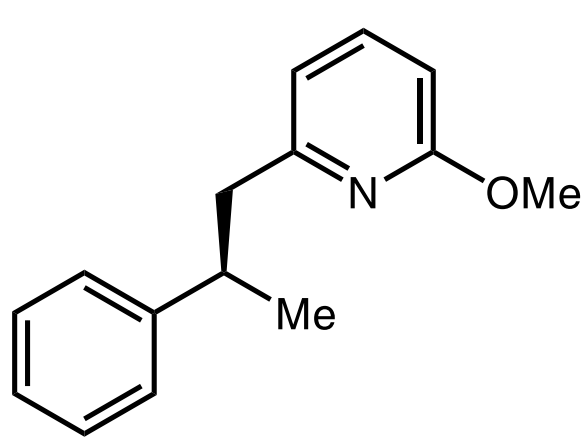

This compound was prepared by following General Procedure B. Purified by Biotage (50 g Sfär cartridge, 100% hexanes for 2 CV, 0–20% EtOAc/hexanes for 10 CV, then 20% EtOAc/hexanes for 2 CV) to afford the product as a colorless oil. **^{1}H NMR** (400 MHz, $CDCl_3$) δ: 7.39 (dd, J_1 = 12.0 Hz, J_2 = 8.0 Hz, 1H), 7.30 – 7.12 (m, 5H), 6.53 (dd, J_1 = 12.0 Hz, J_2 = 8.0 Hz, 2H), 3.91 (s, 3H), 3.42 – 3.27 (m, 1H), 3.05 – 2.81 (m, 2H), 1.28 (d, *J* = 4.0 Hz, 3H) ppm. **^{13}C NMR** (100 MHz, $CDCl_3$) δ: 163.7, 158.6, 147.2, 138.6, 128.4, 127.2, 126.0, 116.2, 107.5, 53.4, 46.6, 39.9, 21.7 ppm. Spectral data match those previously reported.[10]

HPLC analysis conditions: OJ-H column, hexanes, 1.2 mL/min, 40 °C, t_R (major) = 7.0 min, t_R (minor) = 8.7 min.

Yield: run 1: 51%; run 2: 50%; run 3: 53%; average yield: 51%. **Isolated yield**: 50%, 21.5 mg colorless oil.

Enantioselectivity: run 1: 93 : 7 e.r.; run 2: 93 : 7 e.r.; run 3: 93 : 7 e.r.; average e.r.: 93 : 7.

Specific rotation: $[\alpha]_D^{20}$ = –54.0 (*c* = 0.1, CH_2Cl_2).

VIII. GC-MS calibration curves of products

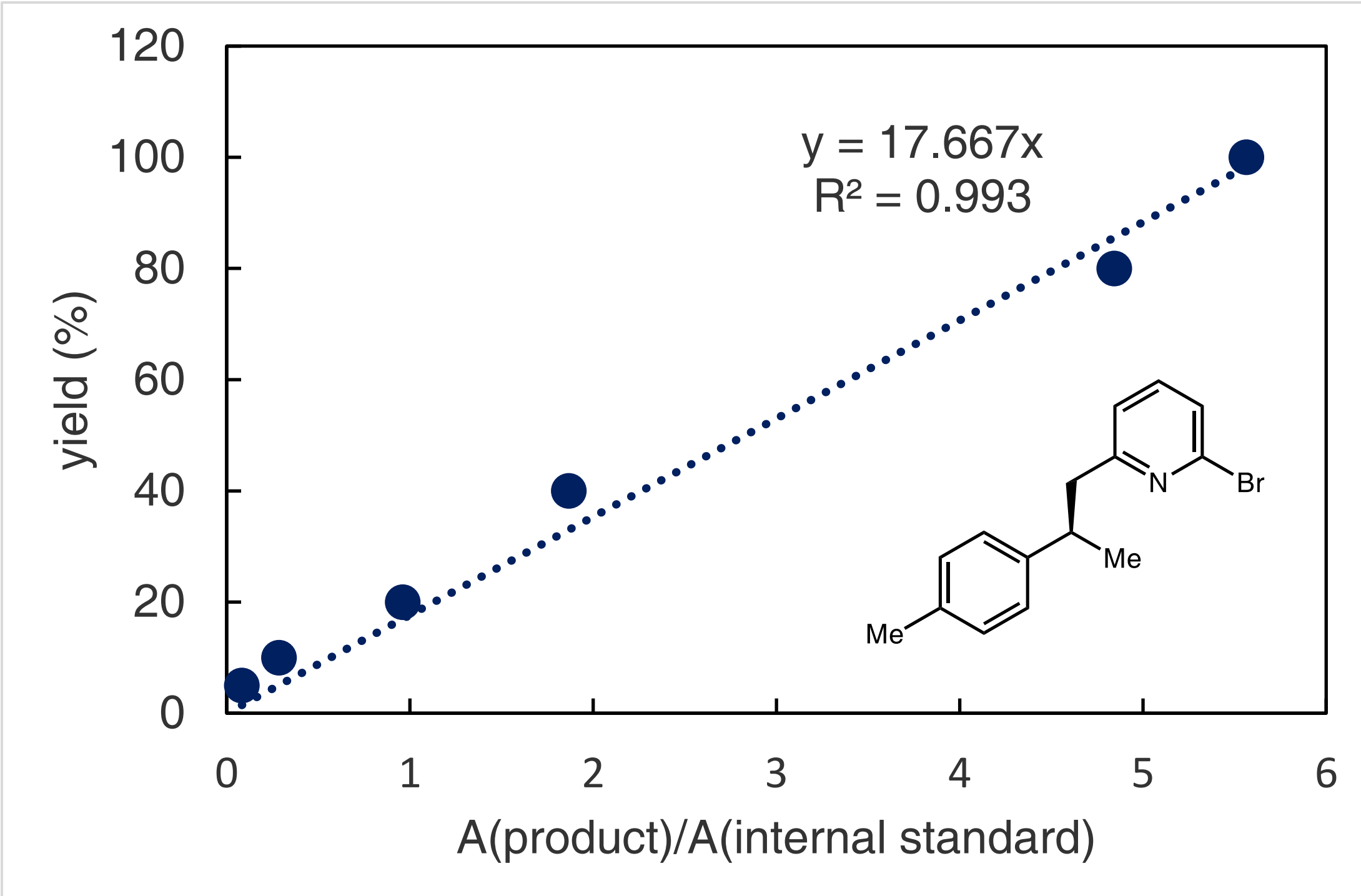

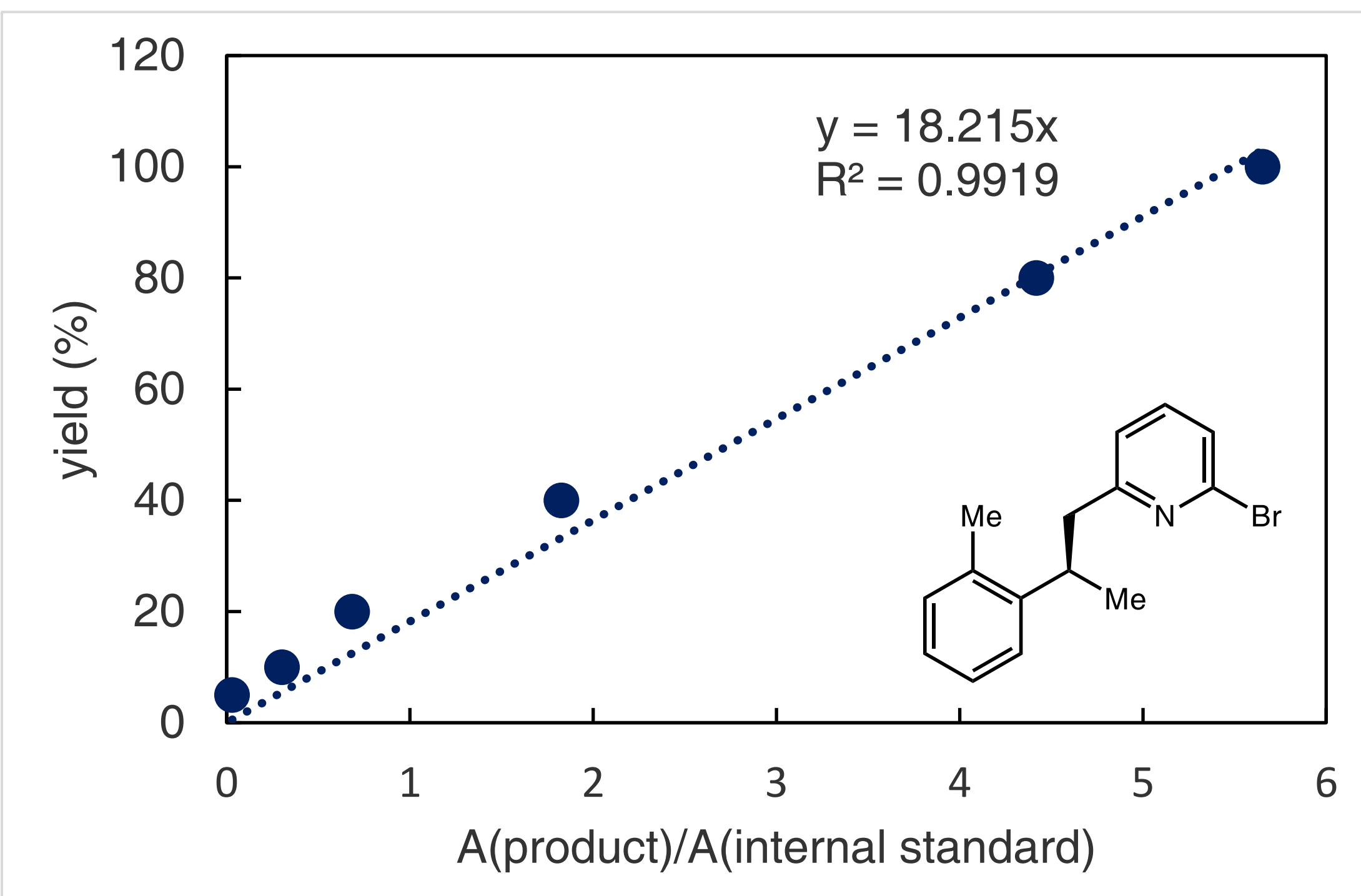

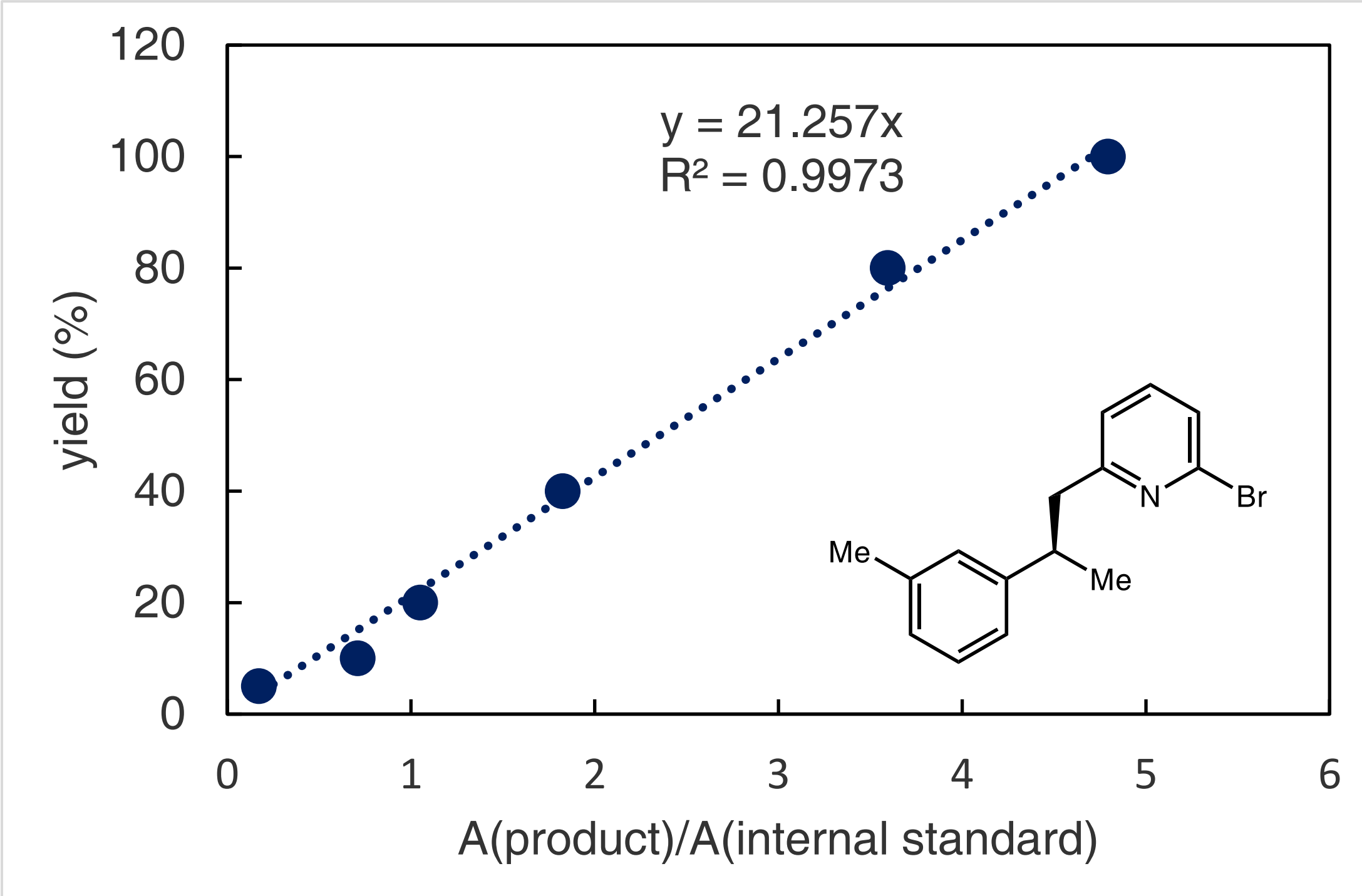
y = 21.257x
R² = 0.9973
yield (%)
A(product)/A(internal standard)
Me
N
Br
Me

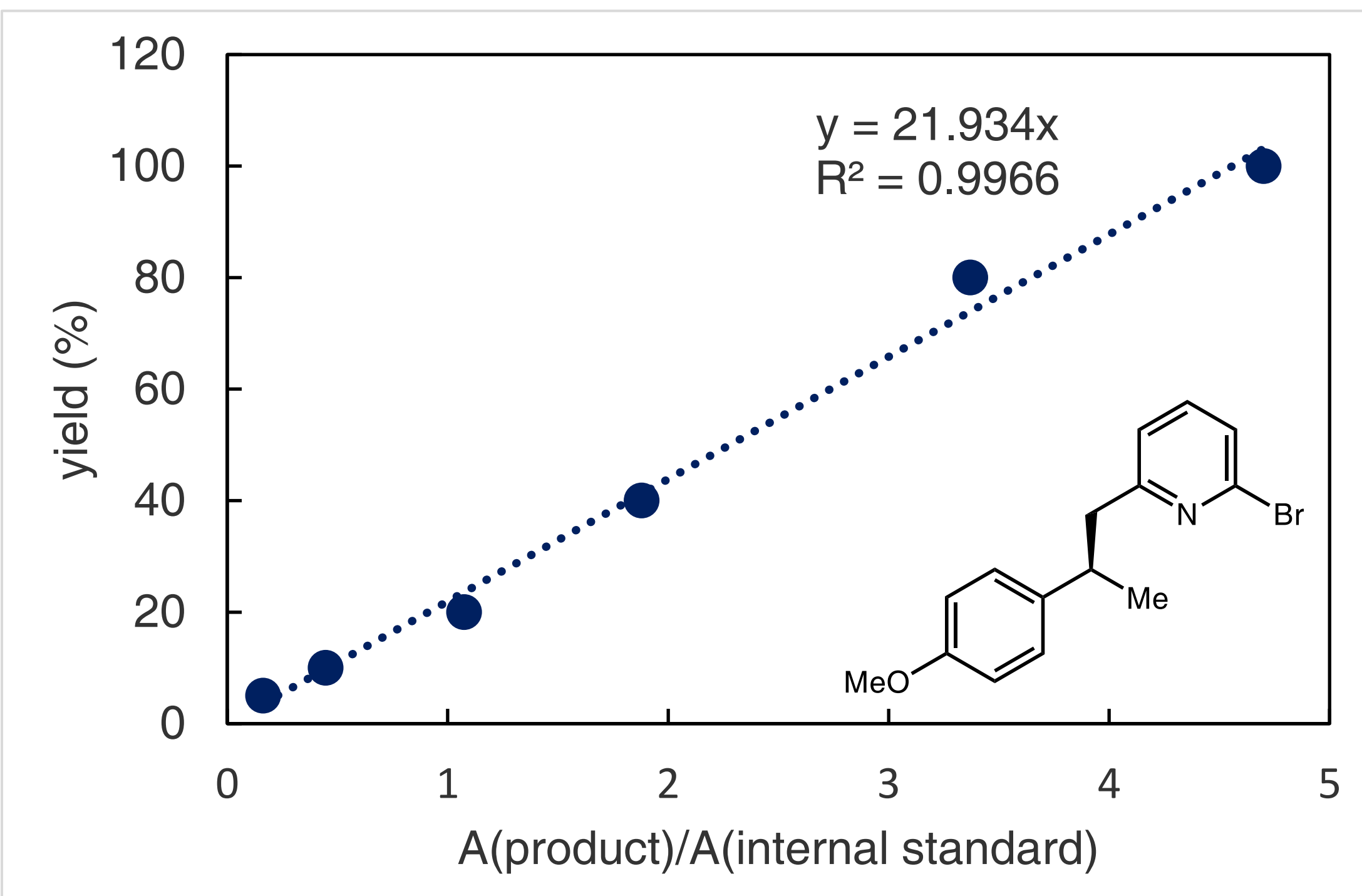
y = 21.934x
R² = 0.9966
yield (%)
A(product)/A(internal standard)
N
Br
Me
MeO

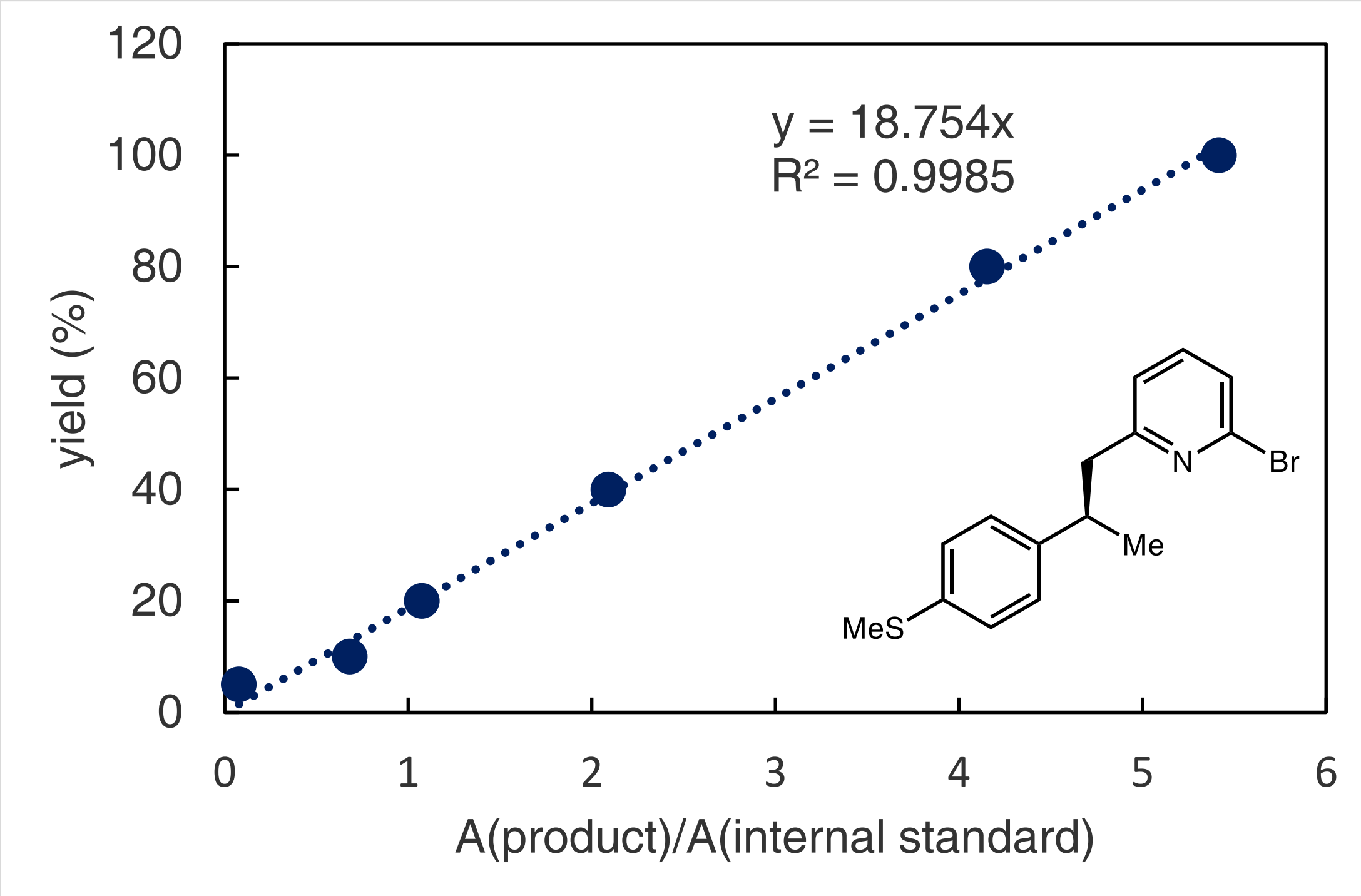
y = 18.754x
R² = 0.9985
yield (%)
A(product)/A(internal standard)
0
20
40
60
80
100
120
1
2
3
4
5
6
N
Br
Me
MeS

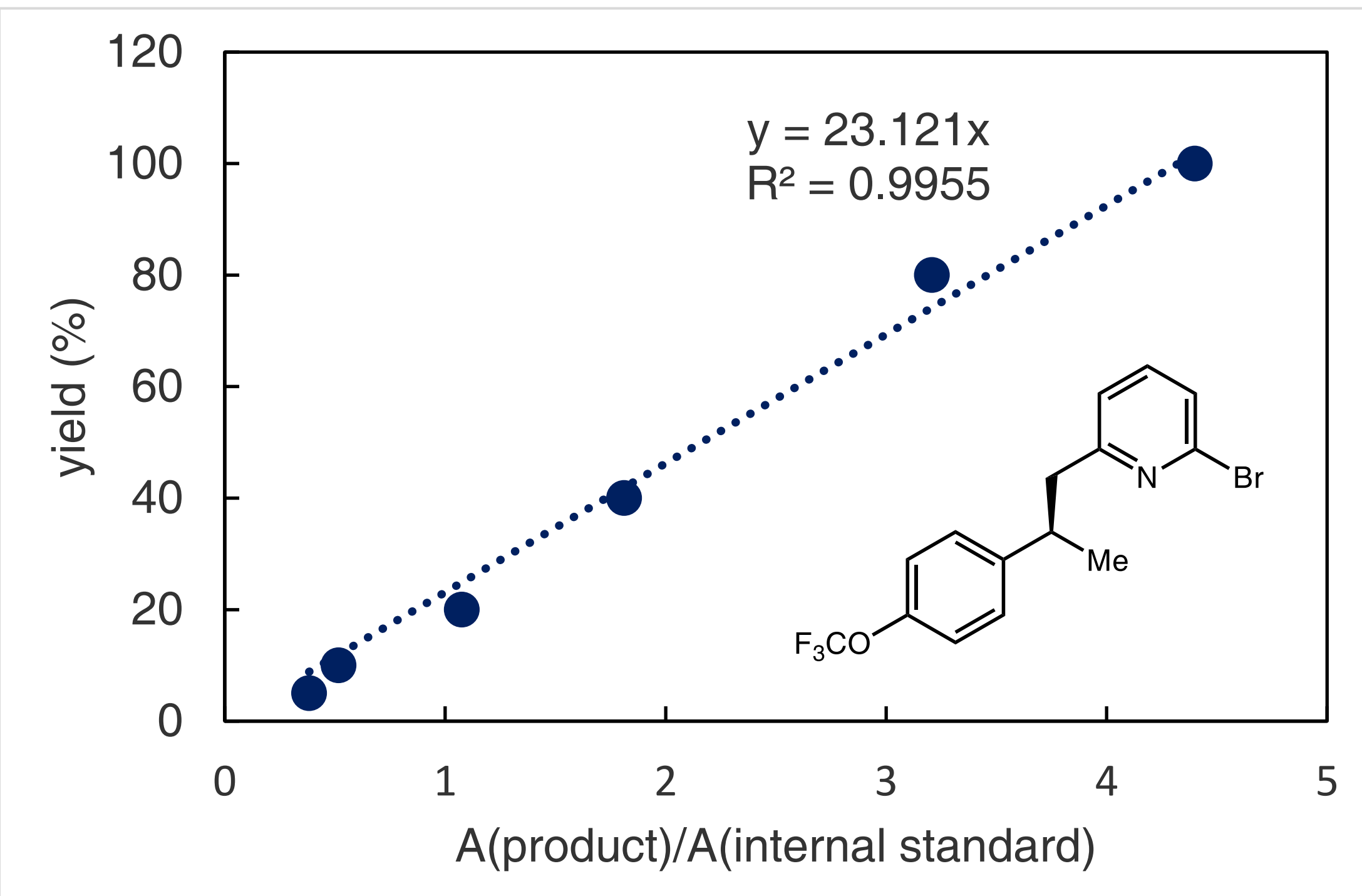
y = 23.121x
R² = 0.9955
yield (%)
A(product)/A(internal standard)
0
20
40
60
80
100
120
1
2
3
4
5
N
Br
Me
F3CO

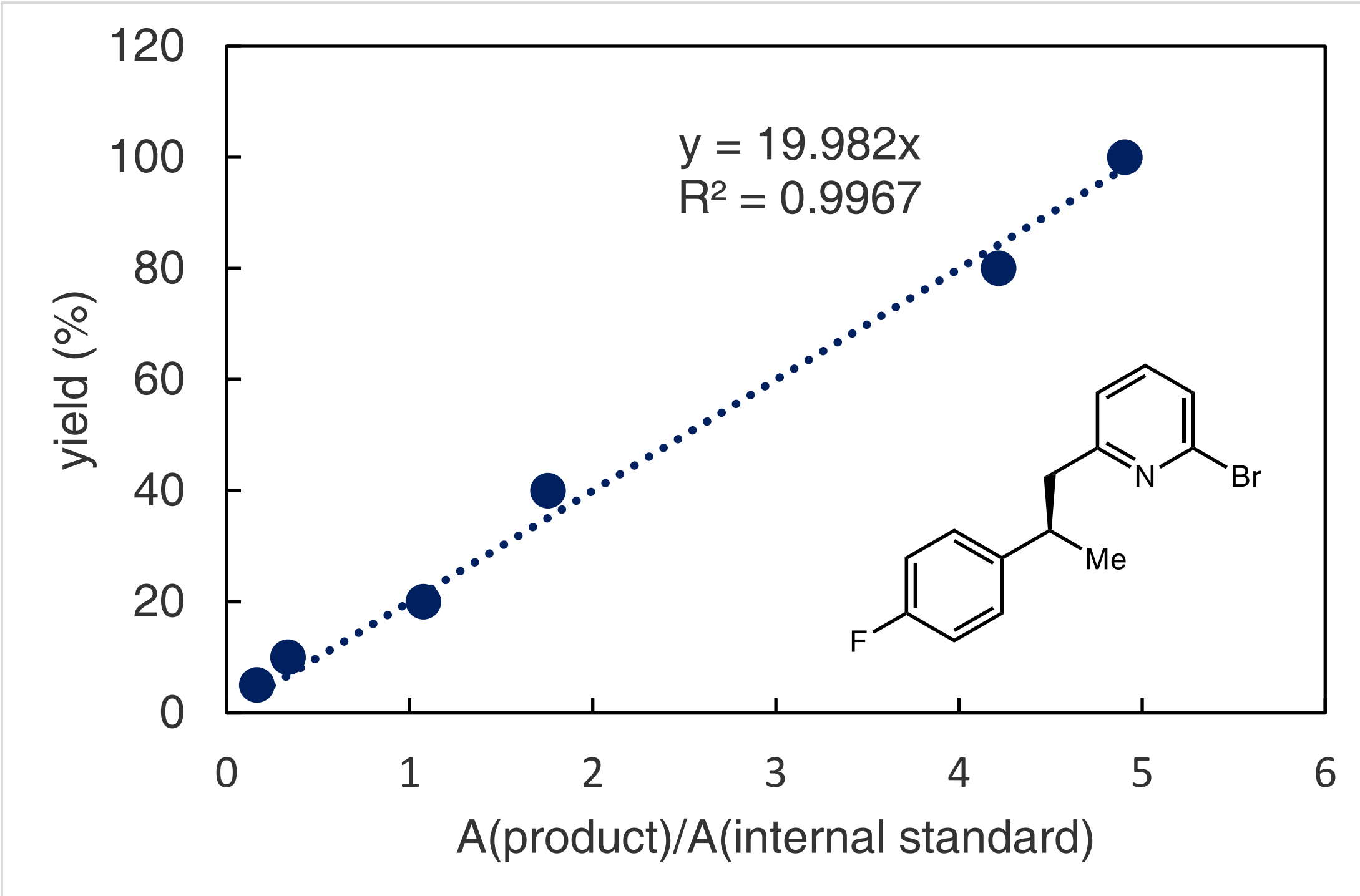

y = 19.982x
R² = 0.9967
yield (%)
A(product)/A(internal standard)
N
Br
Me
F

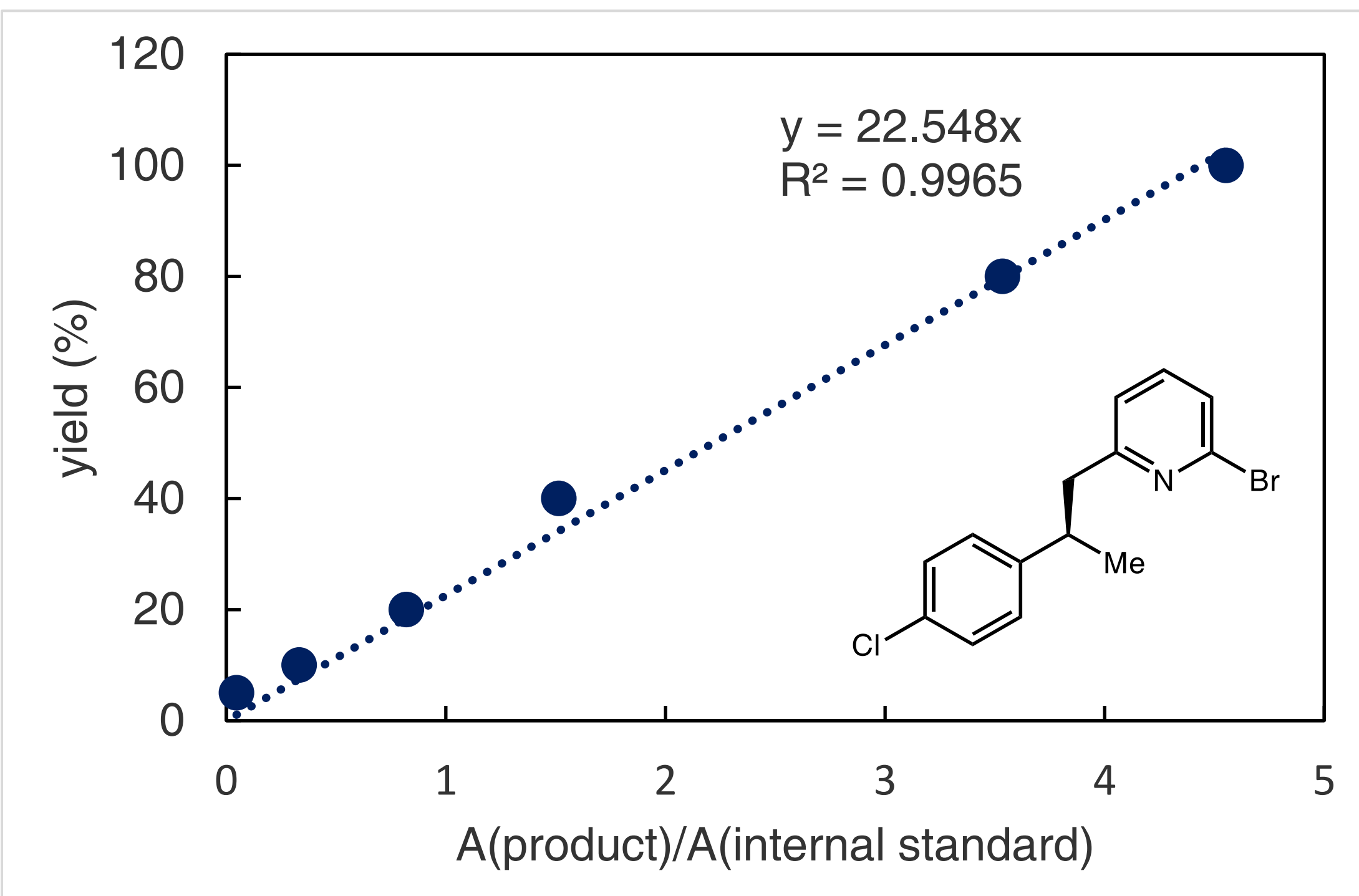

y = 22.548x
R² = 0.9965
yield (%)
A(product)/A(internal standard)
N
Br
Me
Cl

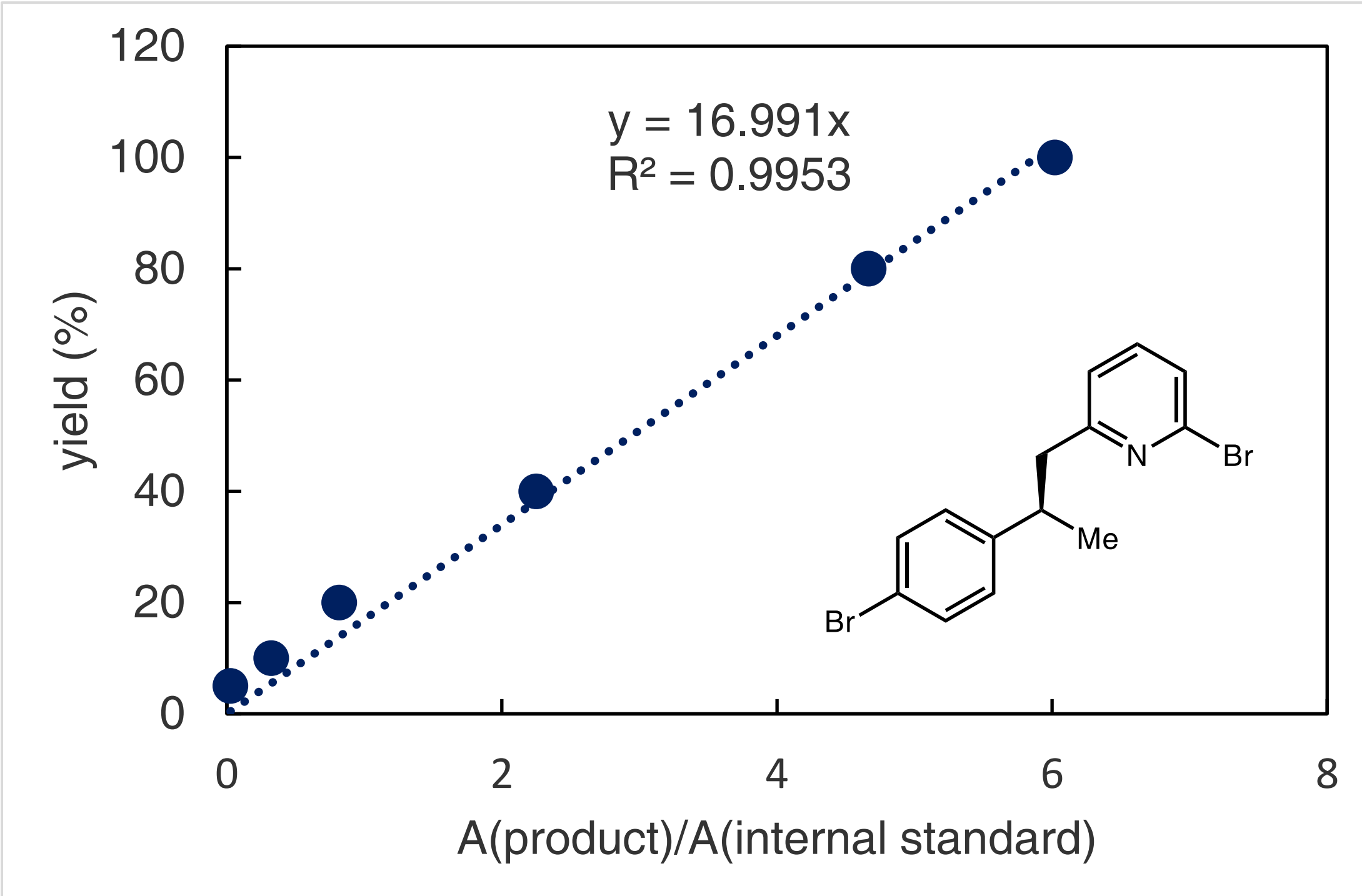
y = 16.991x
R^2 = 0.9953
yield (%)
A(product)/A(internal standard)
Br
N
Me
Br
0
20
40
60
80
100
120
0
2
4
6
8

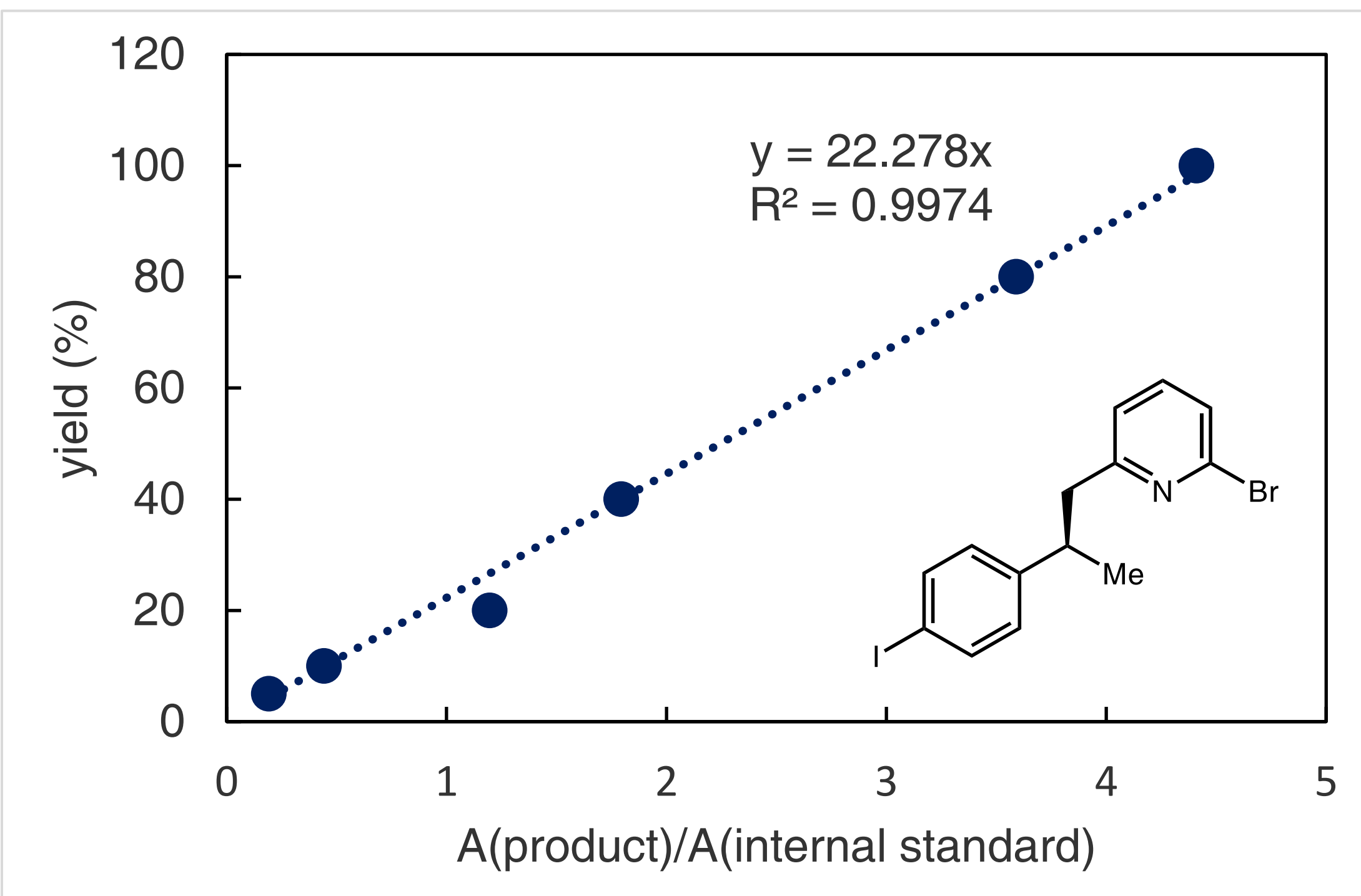
y = 22.278x
R^2 = 0.9974
yield (%)
A(product)/A(internal standard)
Br
N
Me
I
0
20
40
60
80
100
120
0
1
2
3
4
5

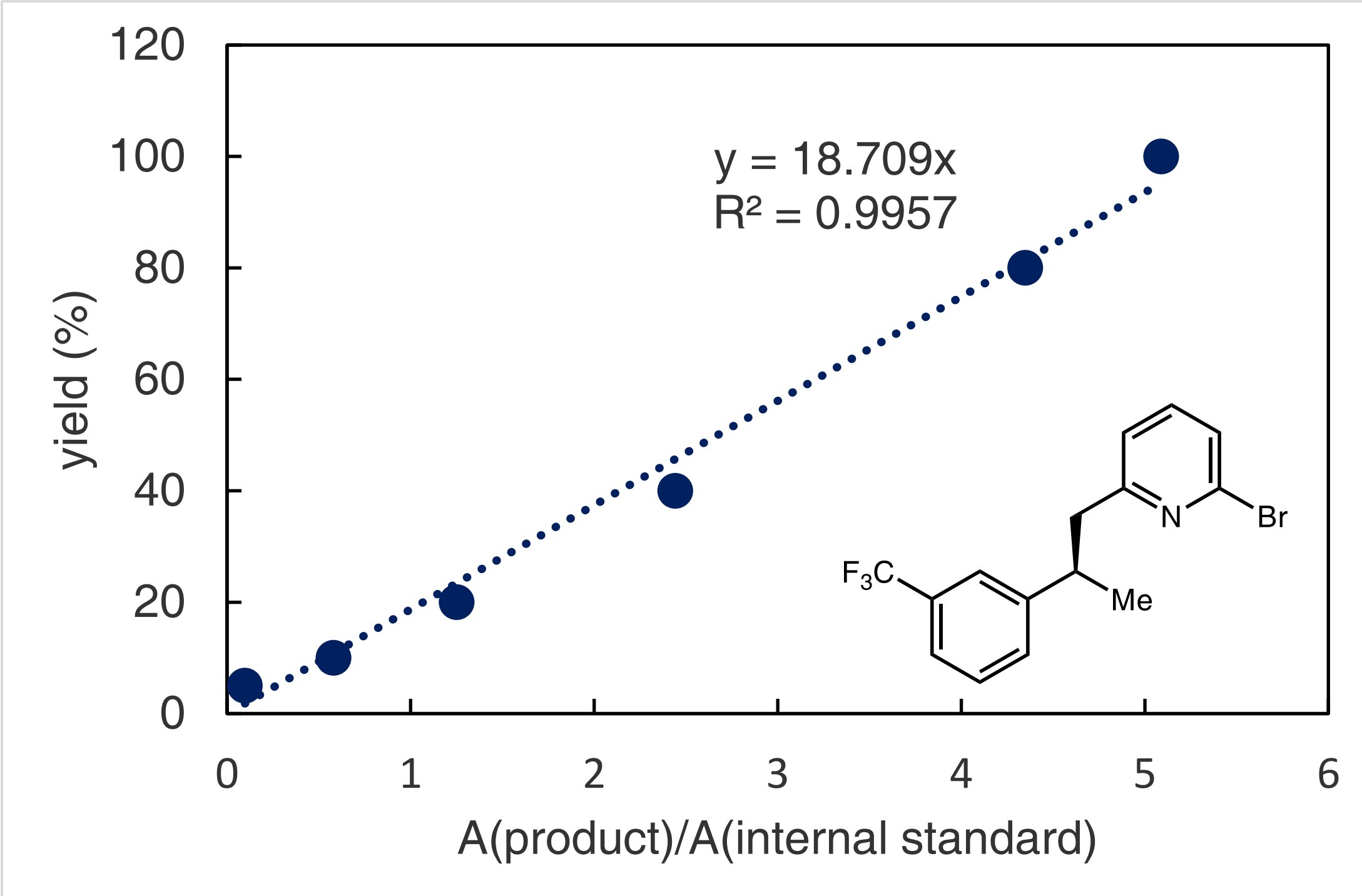

y = 18.709x
R² = 0.9957
yield (%)
A(product)/A(internal standard)
120
100
80
60
40
20
0
0
1
2
3
4
5
6
F3C
N
Br
Me

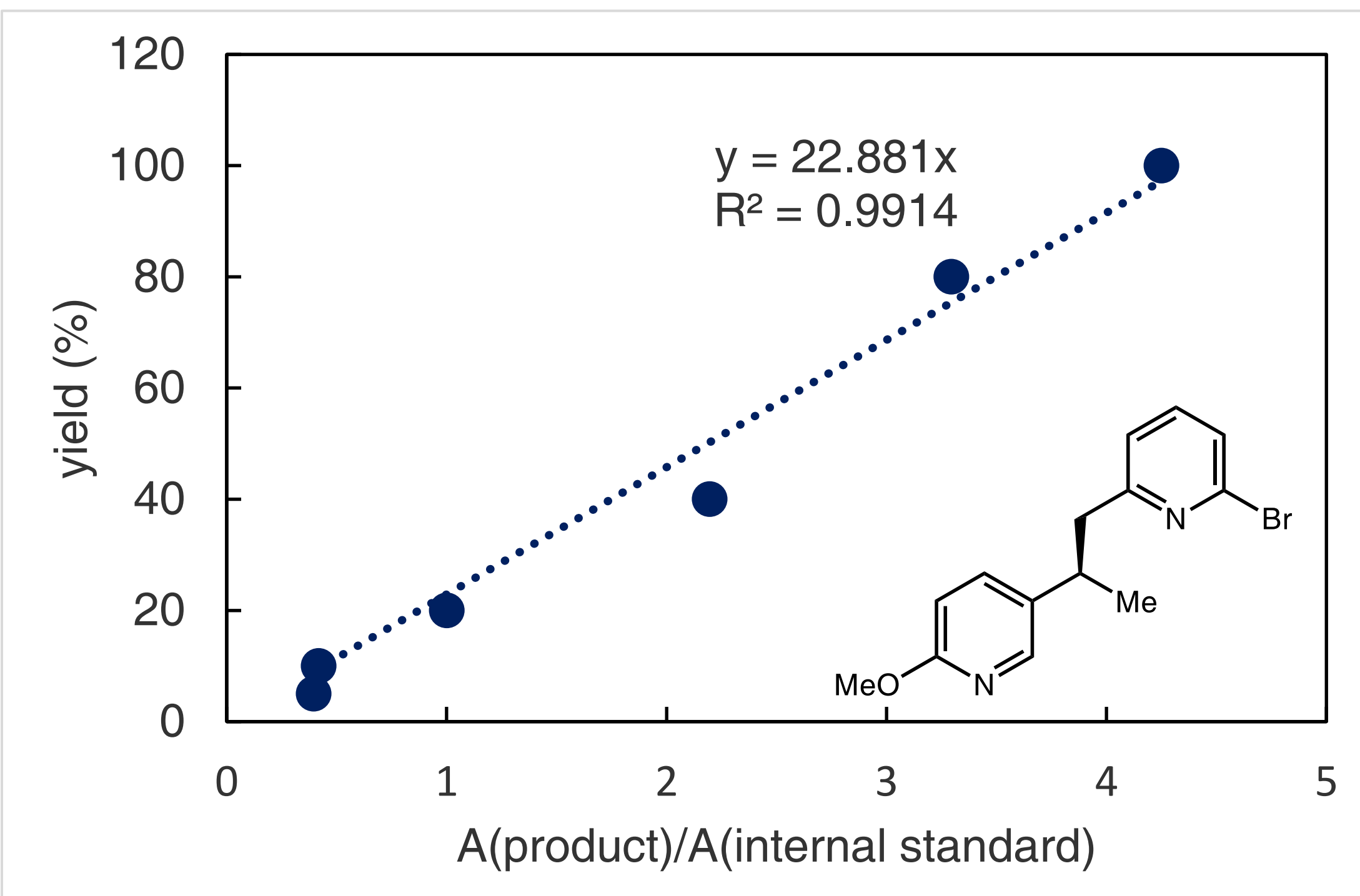

y = 22.881x
R² = 0.9914
yield (%)
A(product)/A(internal standard)
120
100
80
60
40
20
0
0
1
2
3
4
5
MeO
N
N
Br
Me

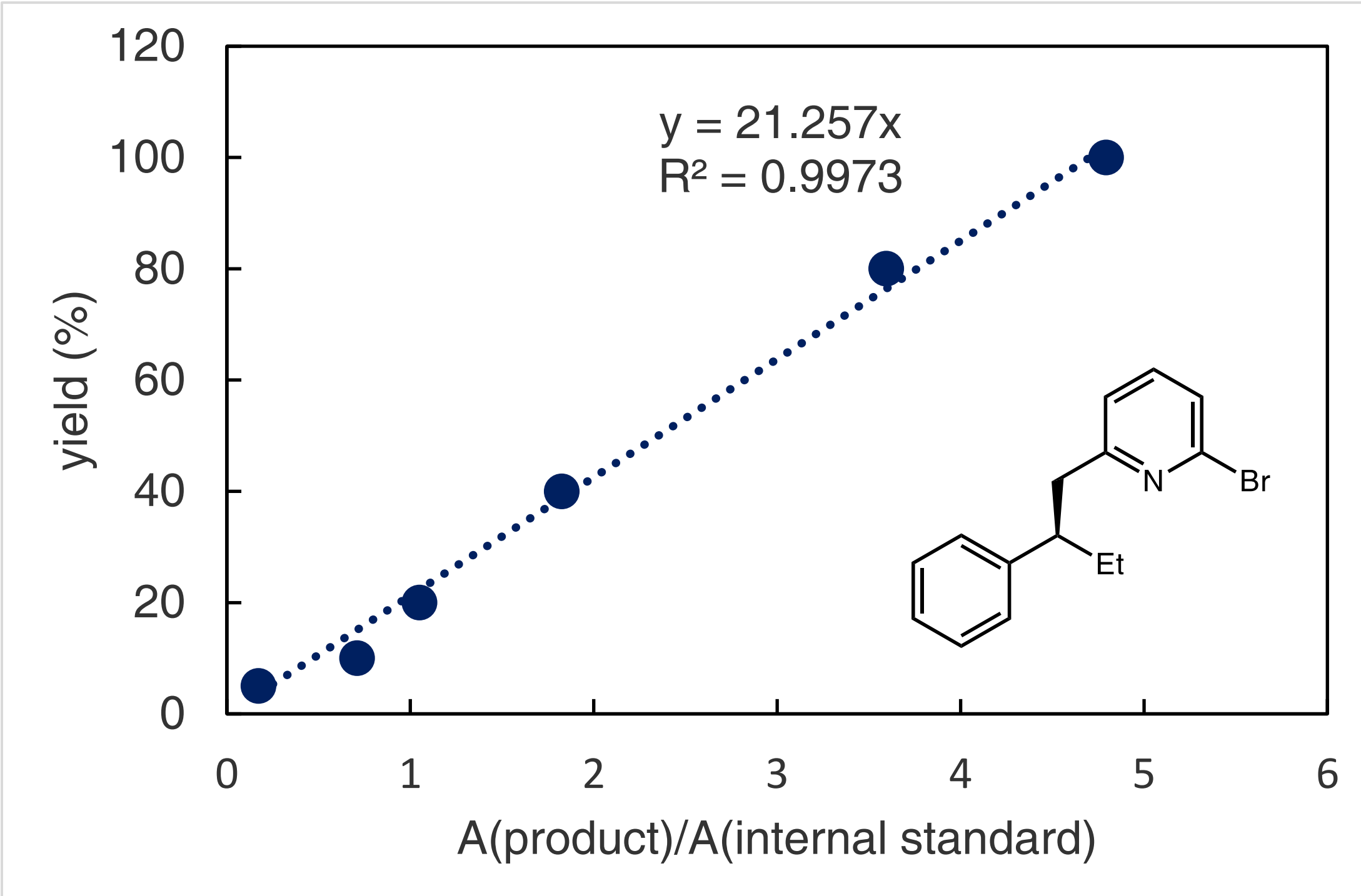
y = 21.257x
R² = 0.9973
yield (%)
A(product)/A(internal standard)
N
Br
Et
0
20
40
60
80
100
120
0
1
2
3
4
5
6

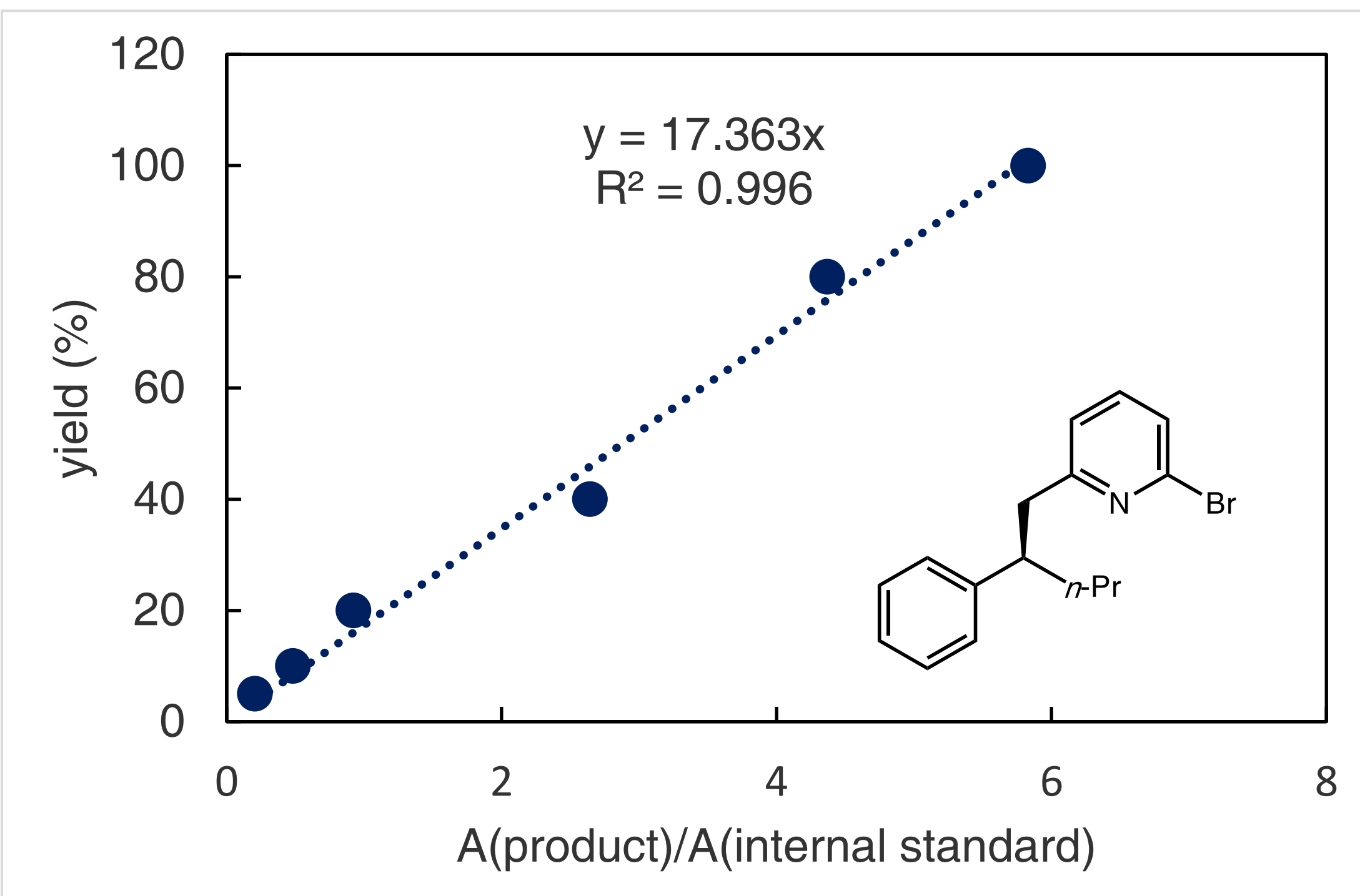
y = 17.363x
R² = 0.996
yield (%)
A(product)/A(internal standard)
N
Br
n-Pr
0
20
40
60
80
100
120
0
2
4
6
8

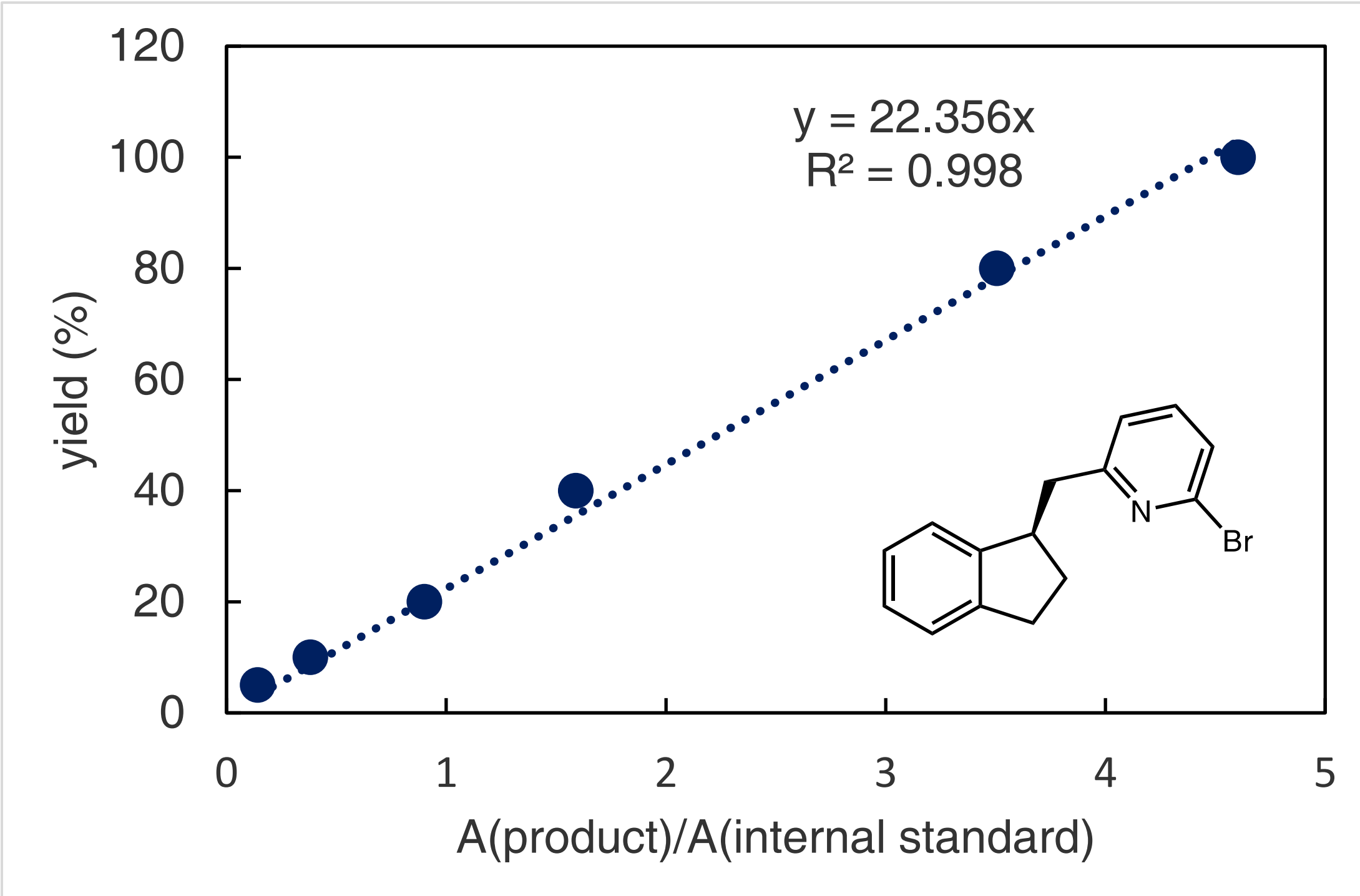
120
100
80
60
40
20
0
yield (%)
0
1
2
3
4
5
A(product)/A(internal standard)
y = 22.356x
R² = 0.998
N
Br

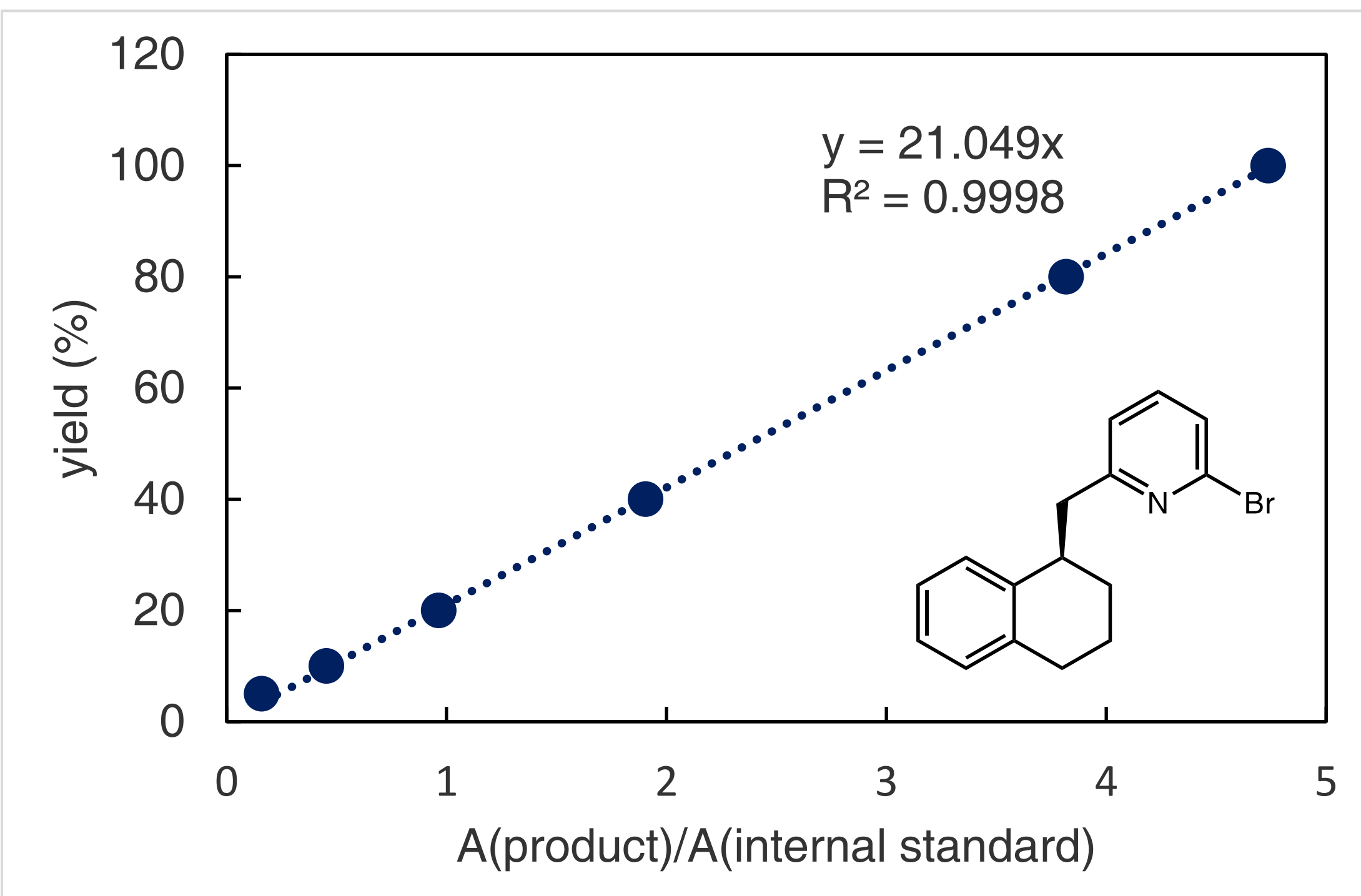
120
100
80
60
40
20
0
yield (%)
0
1
2
3
4
5
A(product)/A(internal standard)
y = 21.049x
R² = 0.9998
N
Br

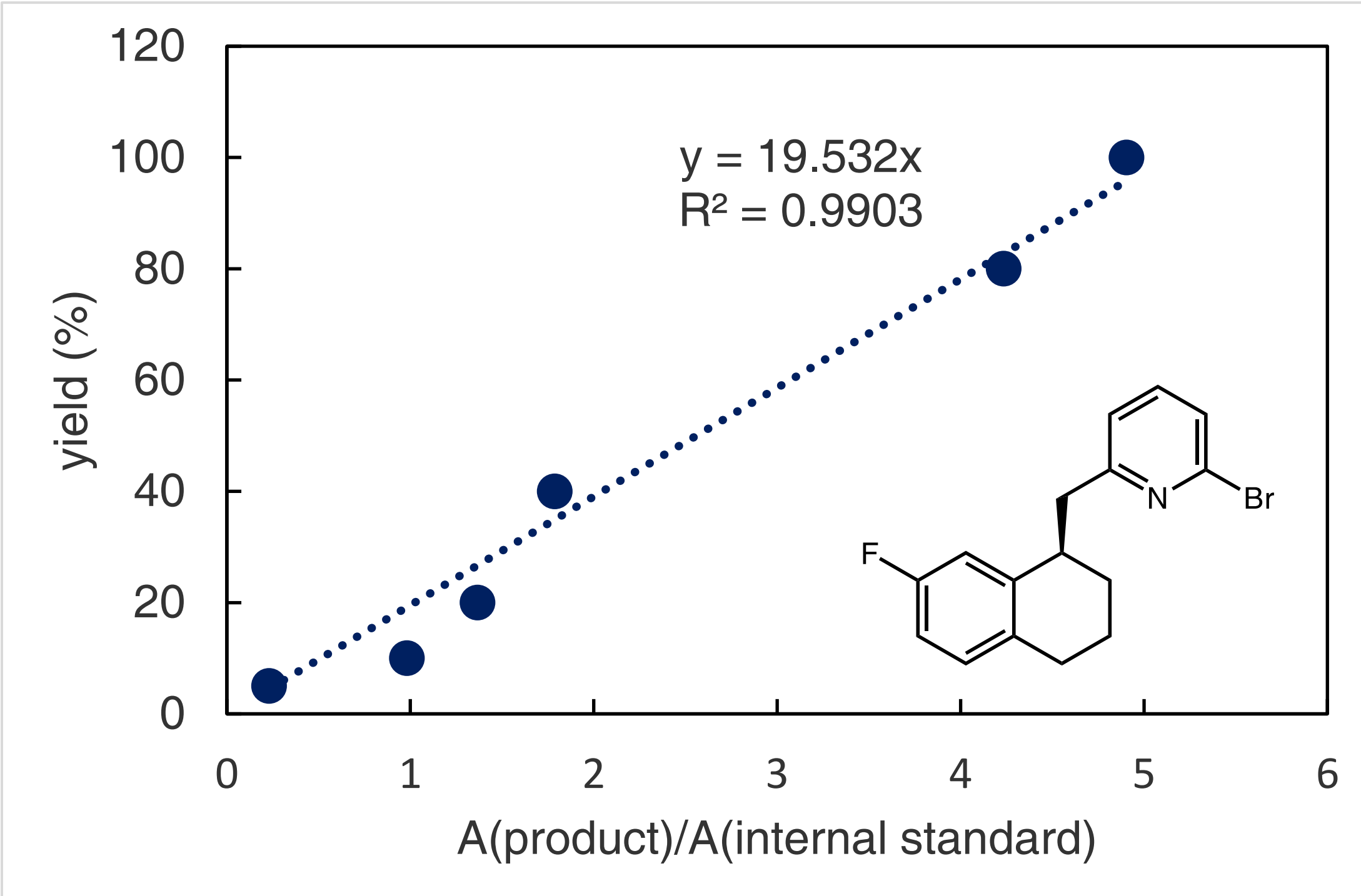

y = 19.532x
R² = 0.9903
yield (%)
A(product)/A(internal standard)
120
100
80
60
40
20
0
0
1
2
3
4
5
6
F
N
Br

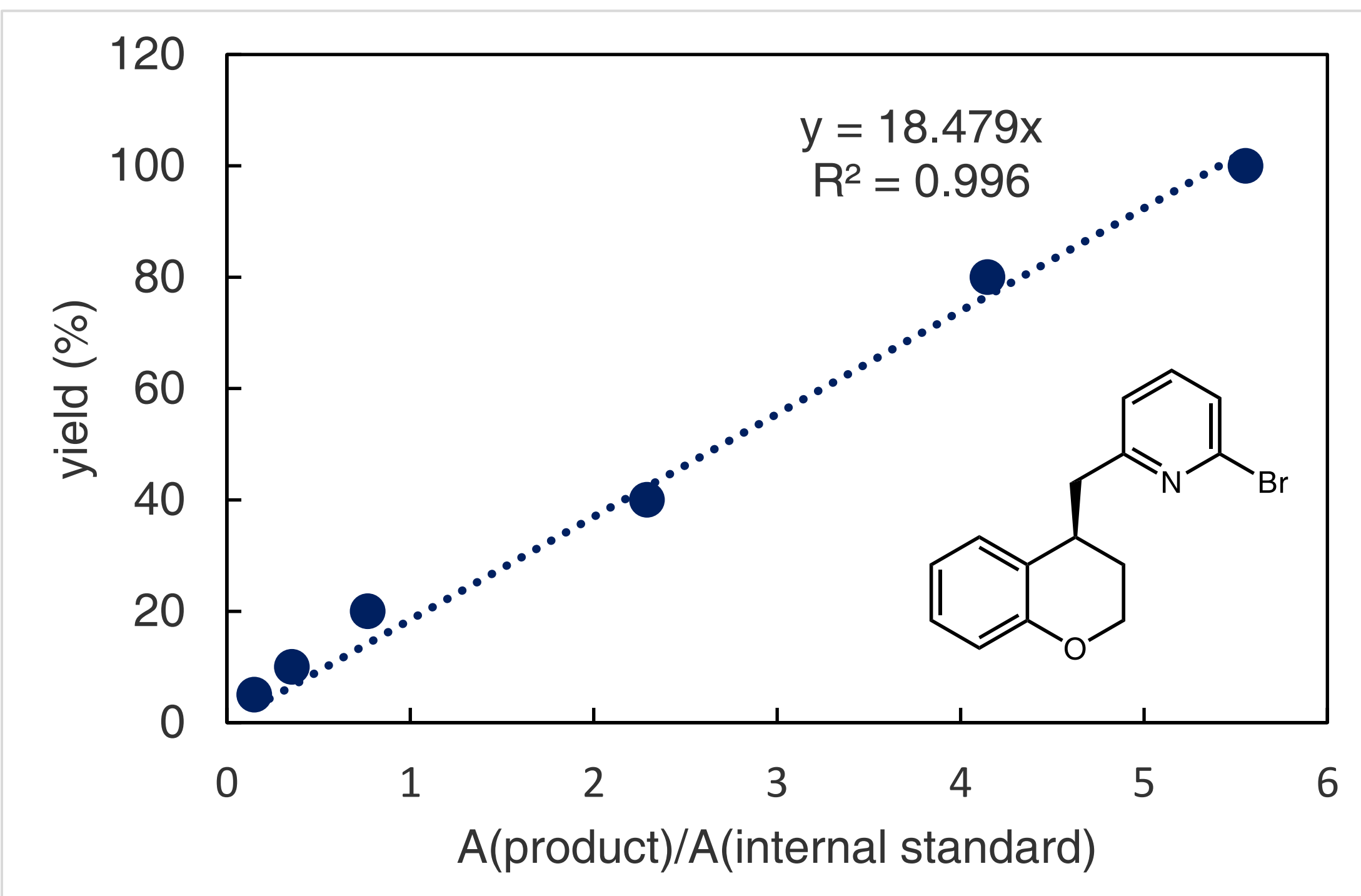

y = 18.479x
R² = 0.996
yield (%)
A(product)/A(internal standard)
120
100
80
60
40
20
0
0
1
2
3
4
5
6
N
Br
O

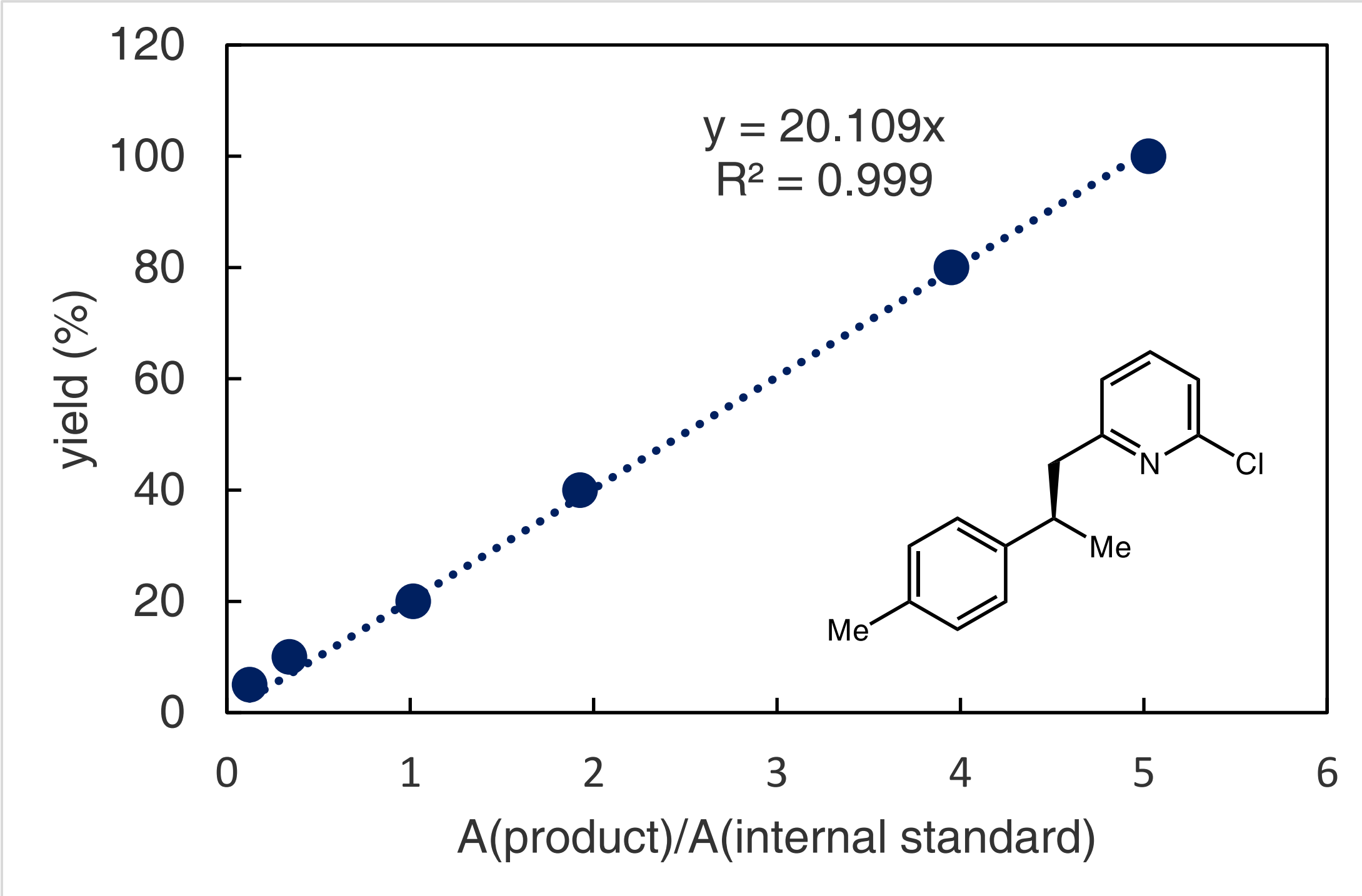
y = 20.109x
R² = 0.999
yield (%)
A(product)/A(internal standard)
N
Cl
Me
Me

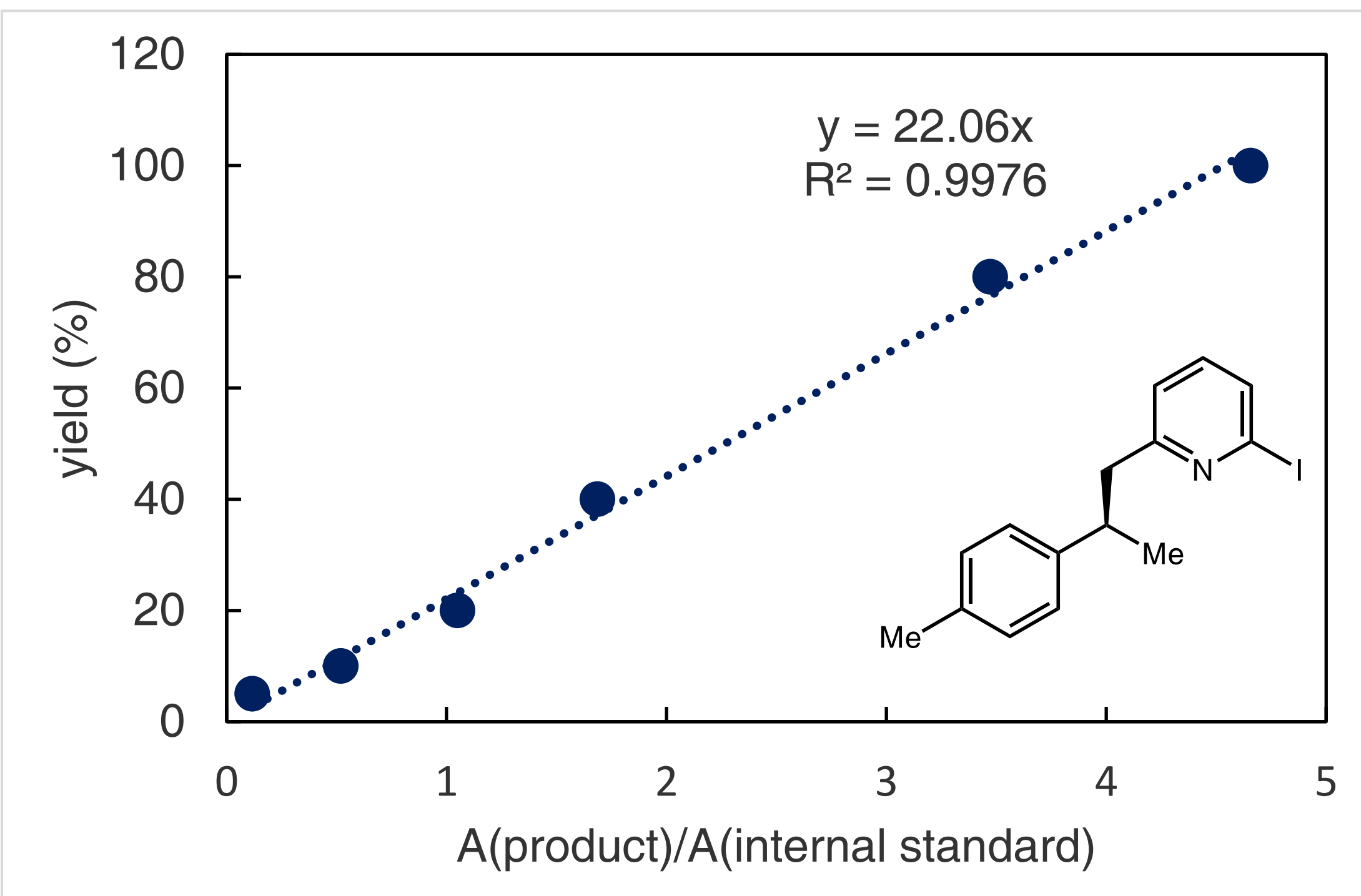
y = 22.06x
R² = 0.9976
yield (%)
A(product)/A(internal standard)
N
I
Me
Me

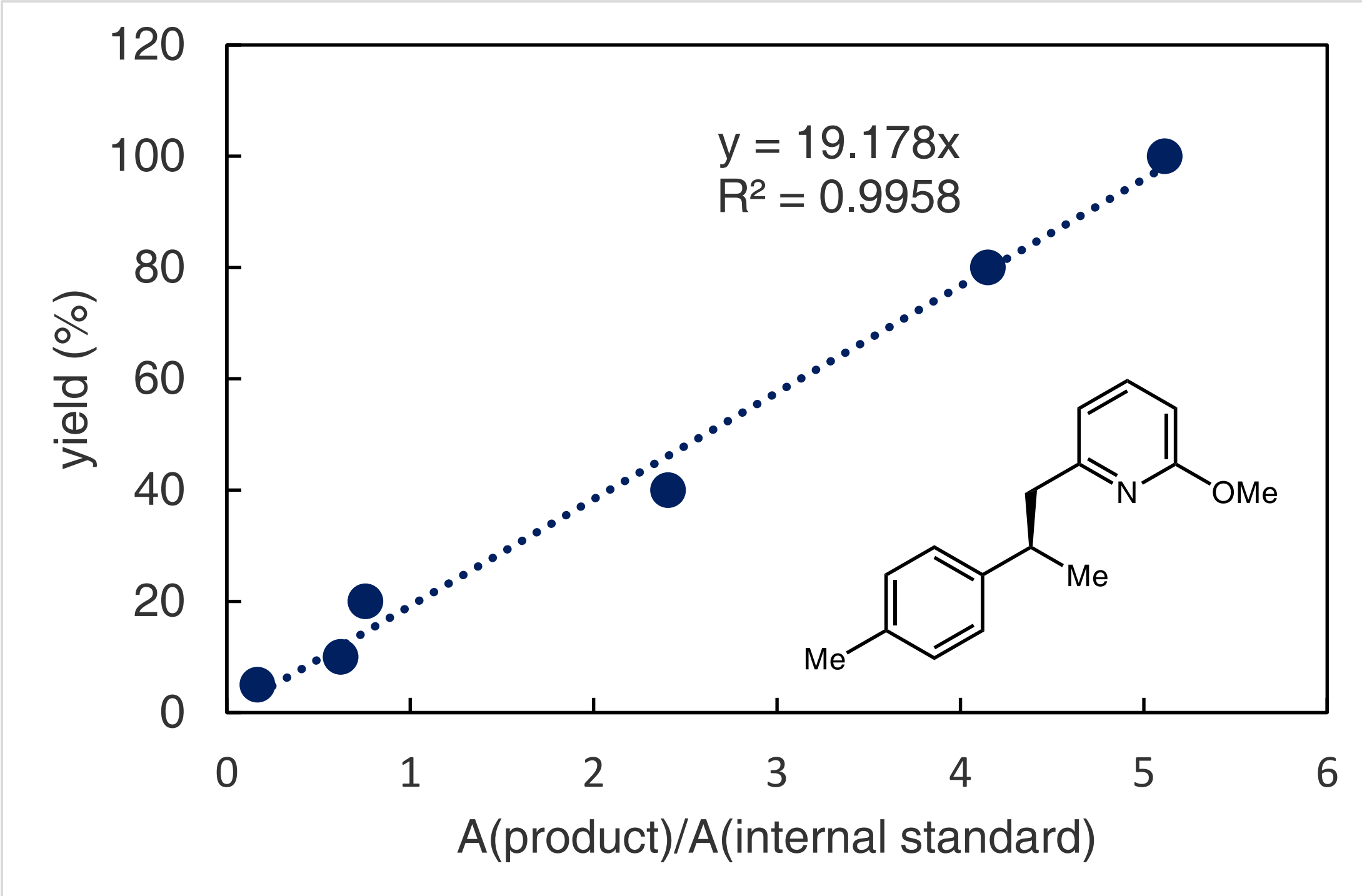
y = 19.178x
R² = 0.9958
120
100
80
60
40
20
0
yield (%)
0
1
2
3
4
5
6
A(product)/A(internal standard)
N
OMe
Me
Me

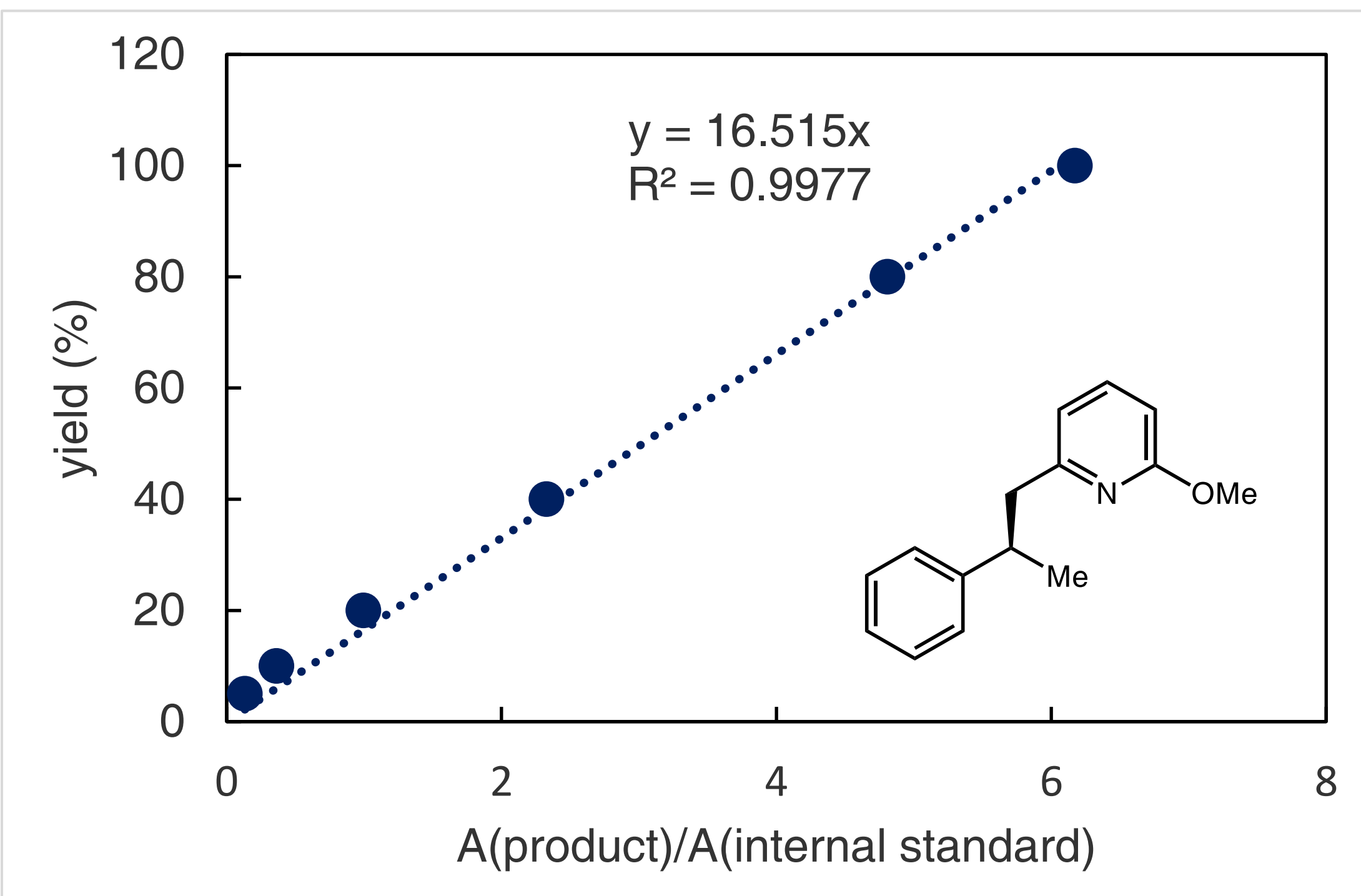
y = 16.515x
R² = 0.9977
120
100
80
60
40
20
0
yield (%)
0
2
4
6
8
A(product)/A(internal standard)
N
OMe
Me

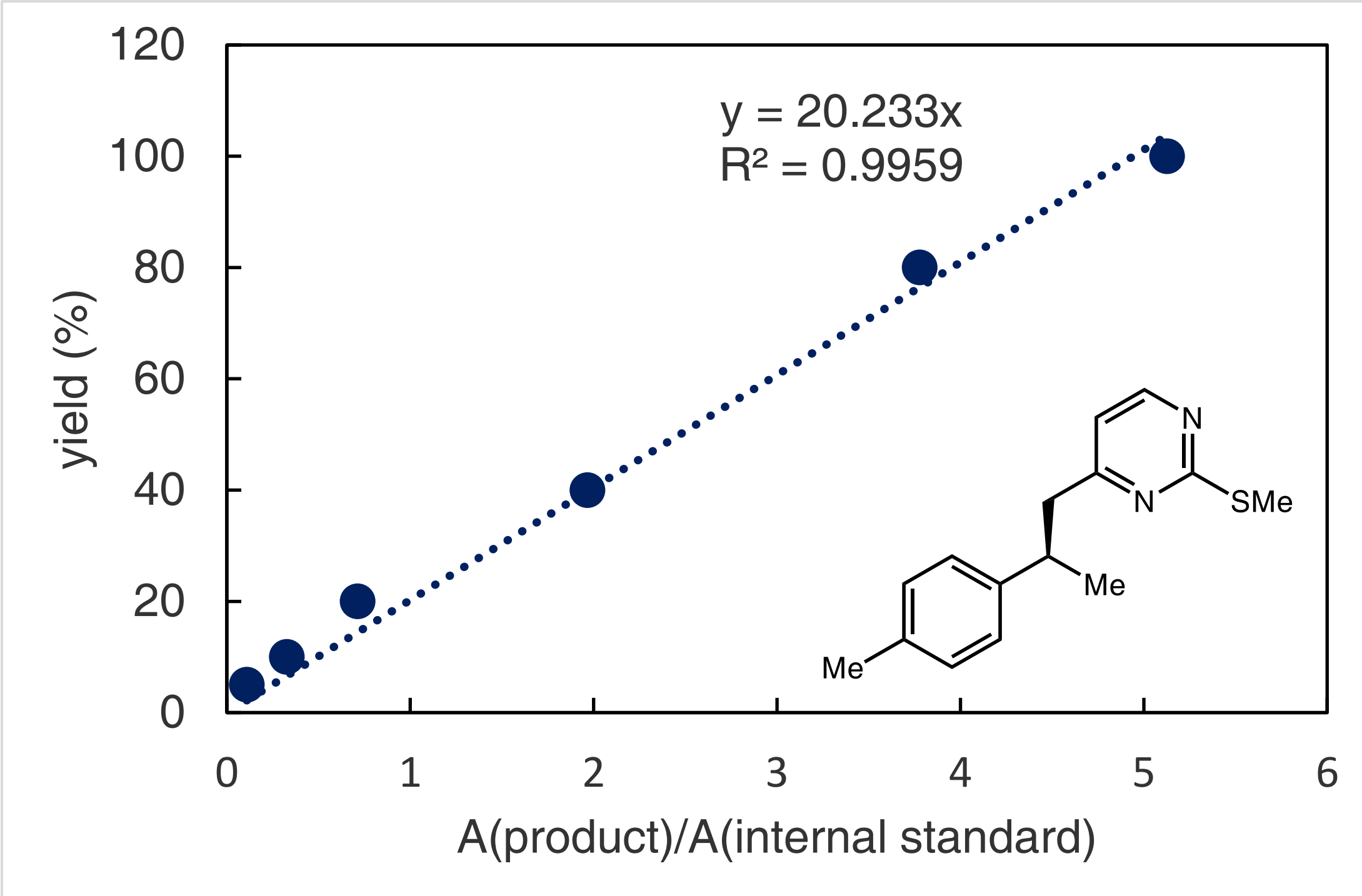
y = 20.233x
R² = 0.9959
yield (%)
A(product)/A(internal standard)
0
20
40
60
80
100
120
0
1
2
3
4
5
6
N
N
SMe
Me
Me

IX. HPLC traces of products

(*R*)-2-Bromo-6-(2-(*p*-tolyl)propyl)pyridine (3a)

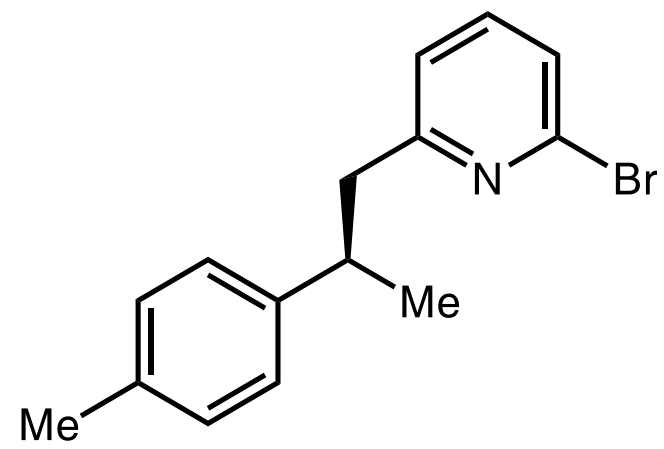

Racemic **3a** (HPLC analysis):

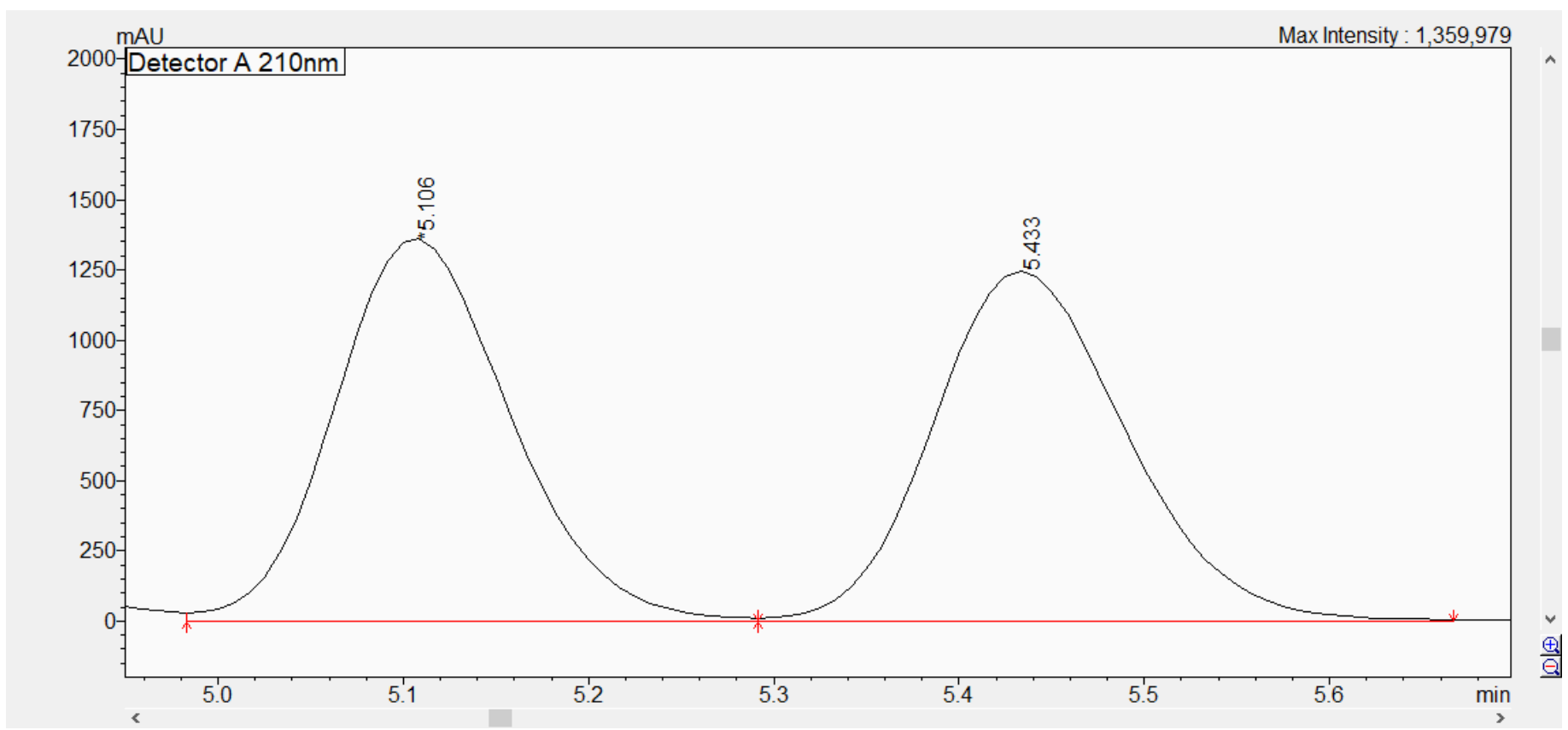

Peak#	Height	Ret. Time	Area	Area%
1	1359593	5.106	9008983	49.942
2	1246024	5.433	9029736	50.058
Total	2605617		18038720	100.000

Enantioenriched (*R*)-**3a** (HPLC analysis):

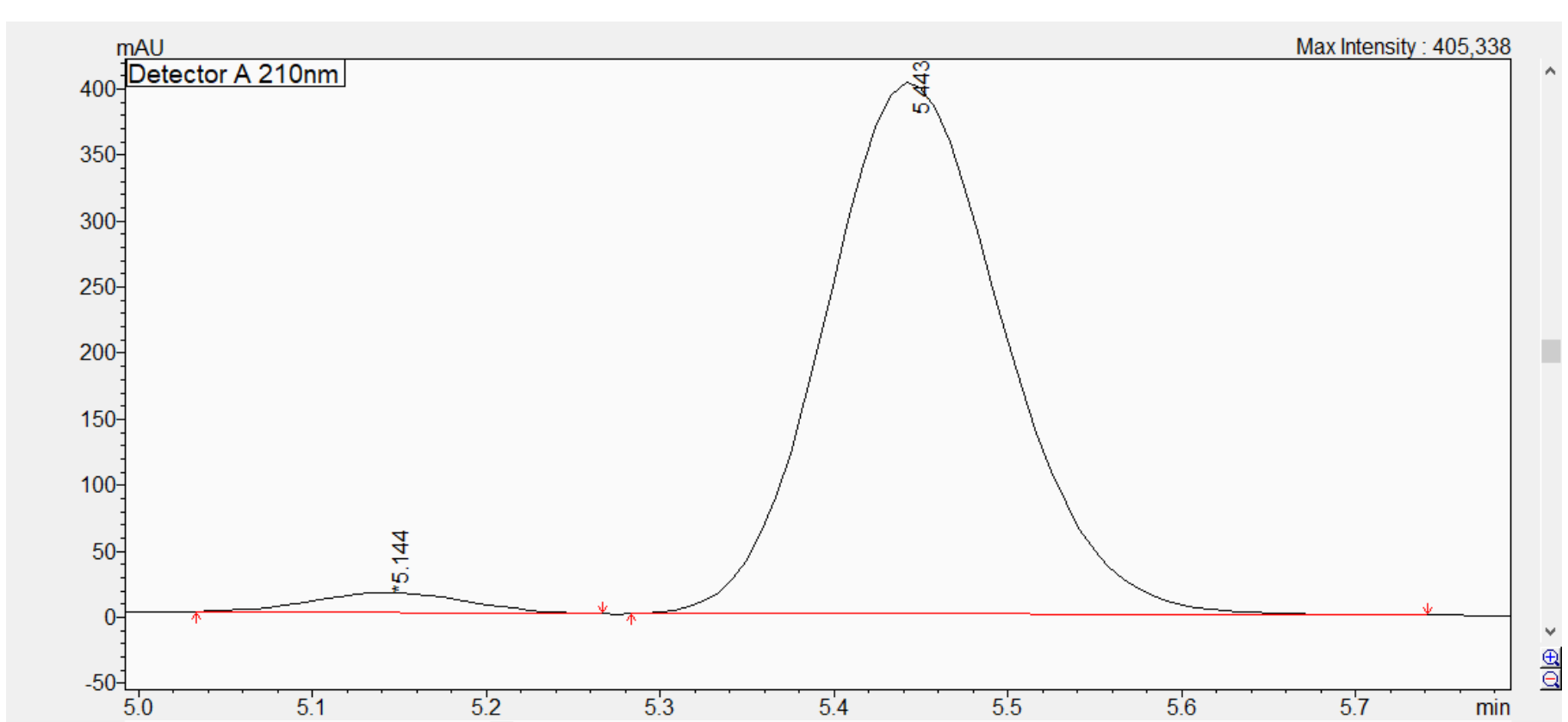

Peak#	Height	Ret. Time	Area	Area%
1	15037	5.144	93443	3.143
2	403113	5.443	2880073	96.857
Total	418150		2973516	100.000

(*R*)-2-Bromo-6-(2-(o-tolyl)propyl)pyridine (3b)

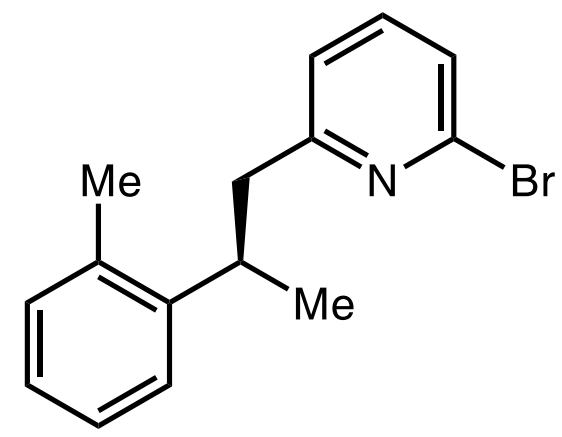

Racemic **3b** (HPLC analysis):

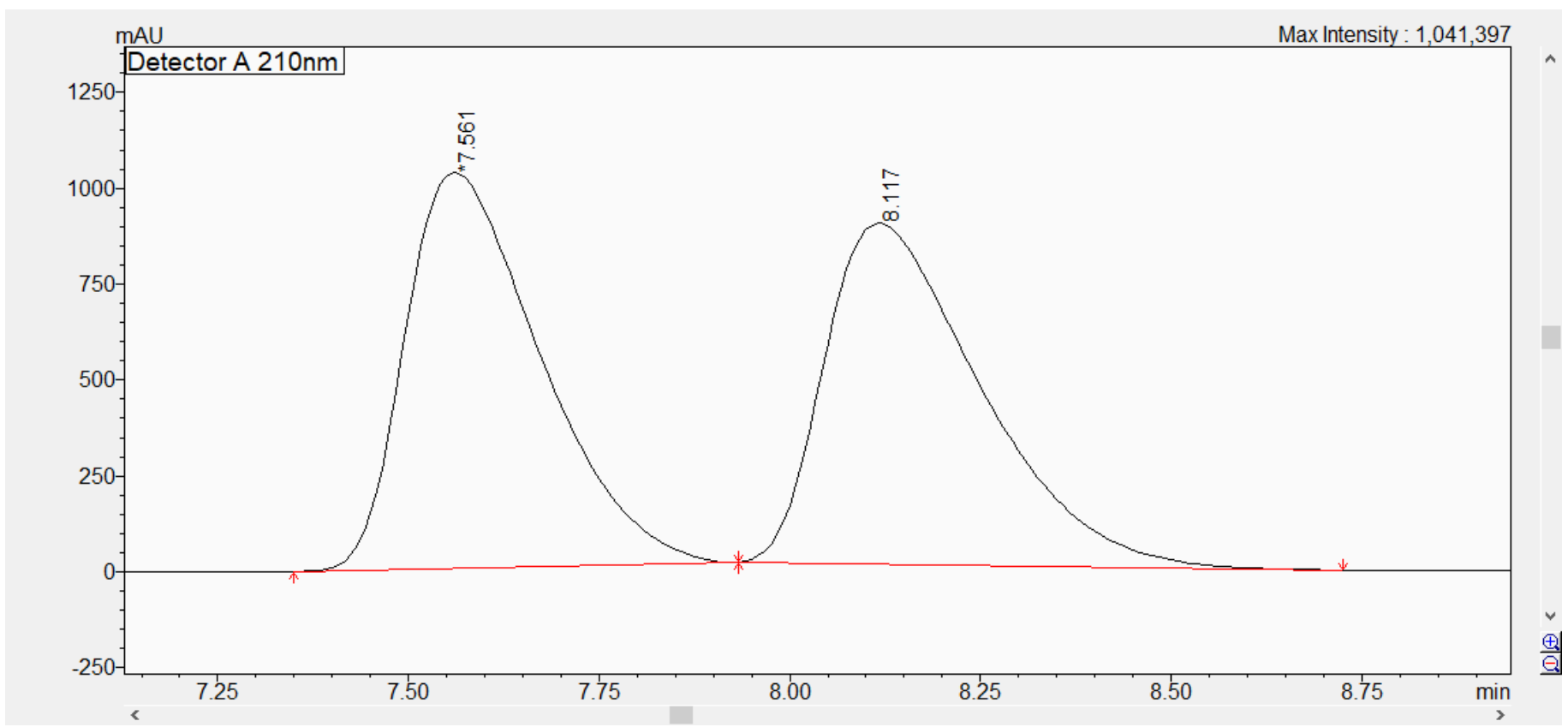

Peak#	Height	Ret. Time	Area	Area%
1	1031405	7.561	12700407	50.089
2	888093	8.117	12655363	49.911
Total	1919498		25355769	100.000

Enantioenriched (*R*)-**3b** (HPLC analysis):

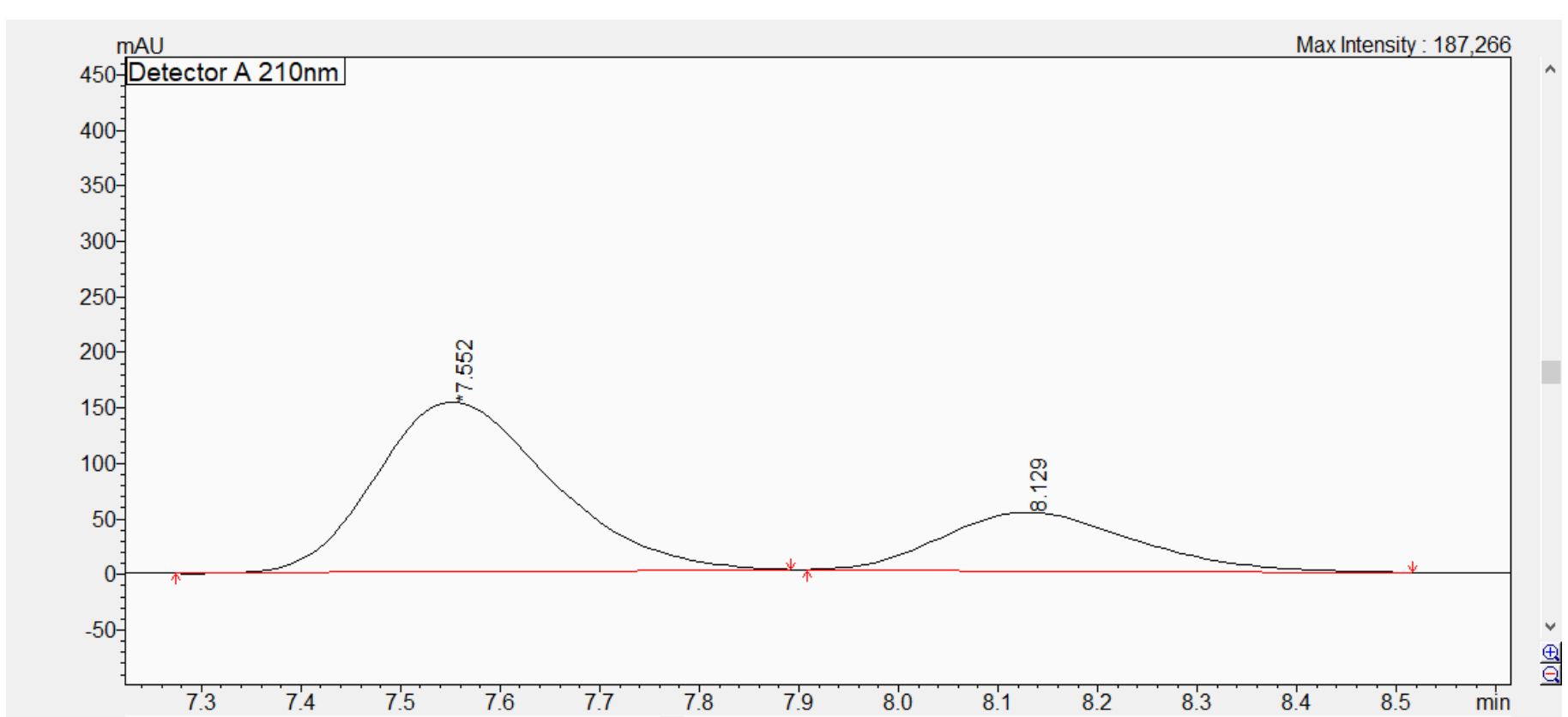

Peak#	Height	Ret. Time	Area	Area%
1	152967	7.552	1874337	72.781
2	52079	8.129	700976	27.219
Total	205046		2575313	100.000

(*R*)-2-Bromo-6-(2-(*m*-tolyl)propyl)pyridine (3c)

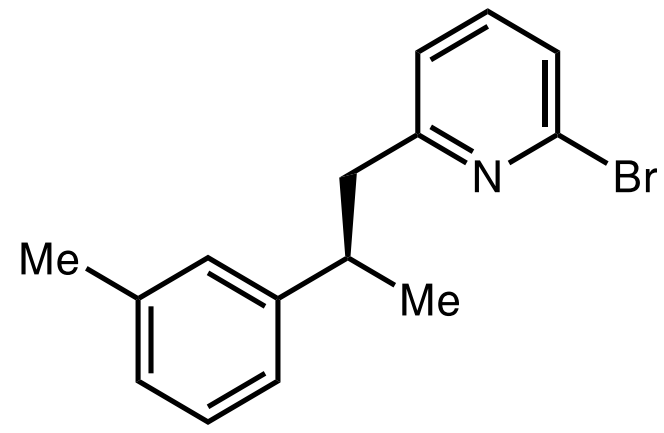

Racemic **3c** (HPLC analysis):

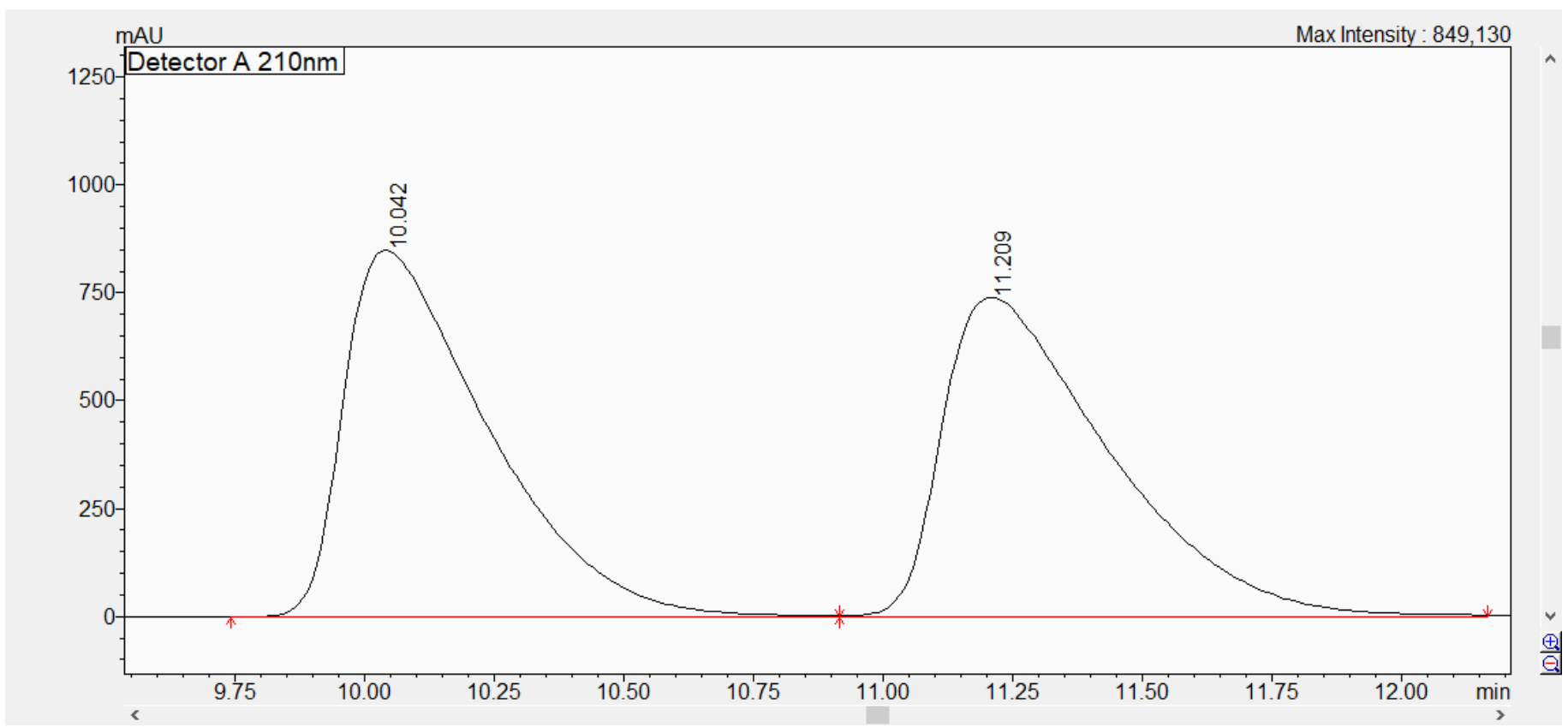

Peak#	Height	Ret. Time	Area	Area%
1	848888	10.042	16410026	49.810
2	739431	11.209	16535415	50.190
Total	1588319		32945440	100.000

Enantioenriched (*R*)-**3c** (HPLC analysis):

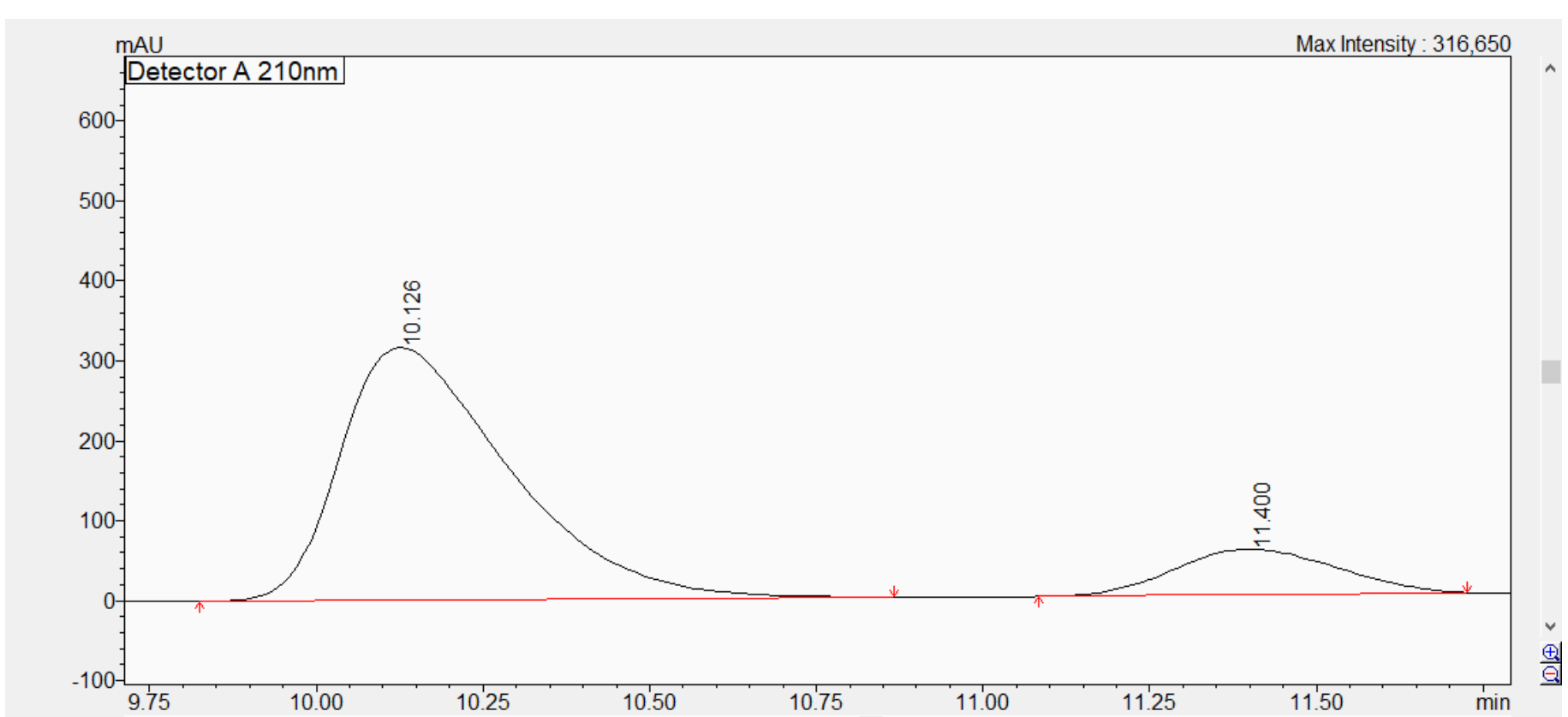

Peak#	Height	Ret. Time	Area	Area%
1	315778	10.126	5563122	85.308
2	56797	11.400	958111	14.692
Total	372575		6521233	100.000

(*R*)-2-Bromo-6-(2-(4-methoxyphenyl)propyl)pyridine (3d)

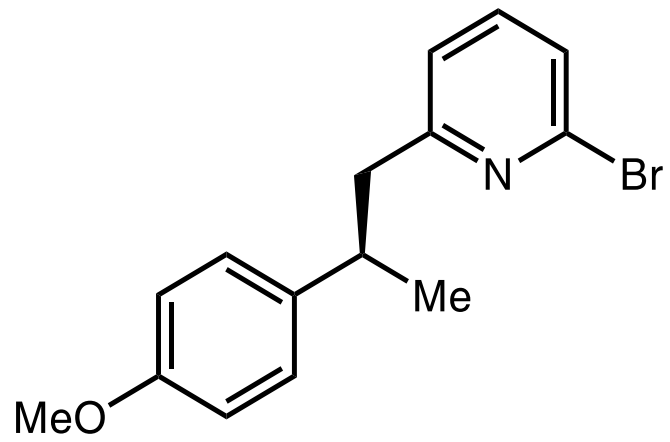

Racemic **3d** (HPLC analysis):

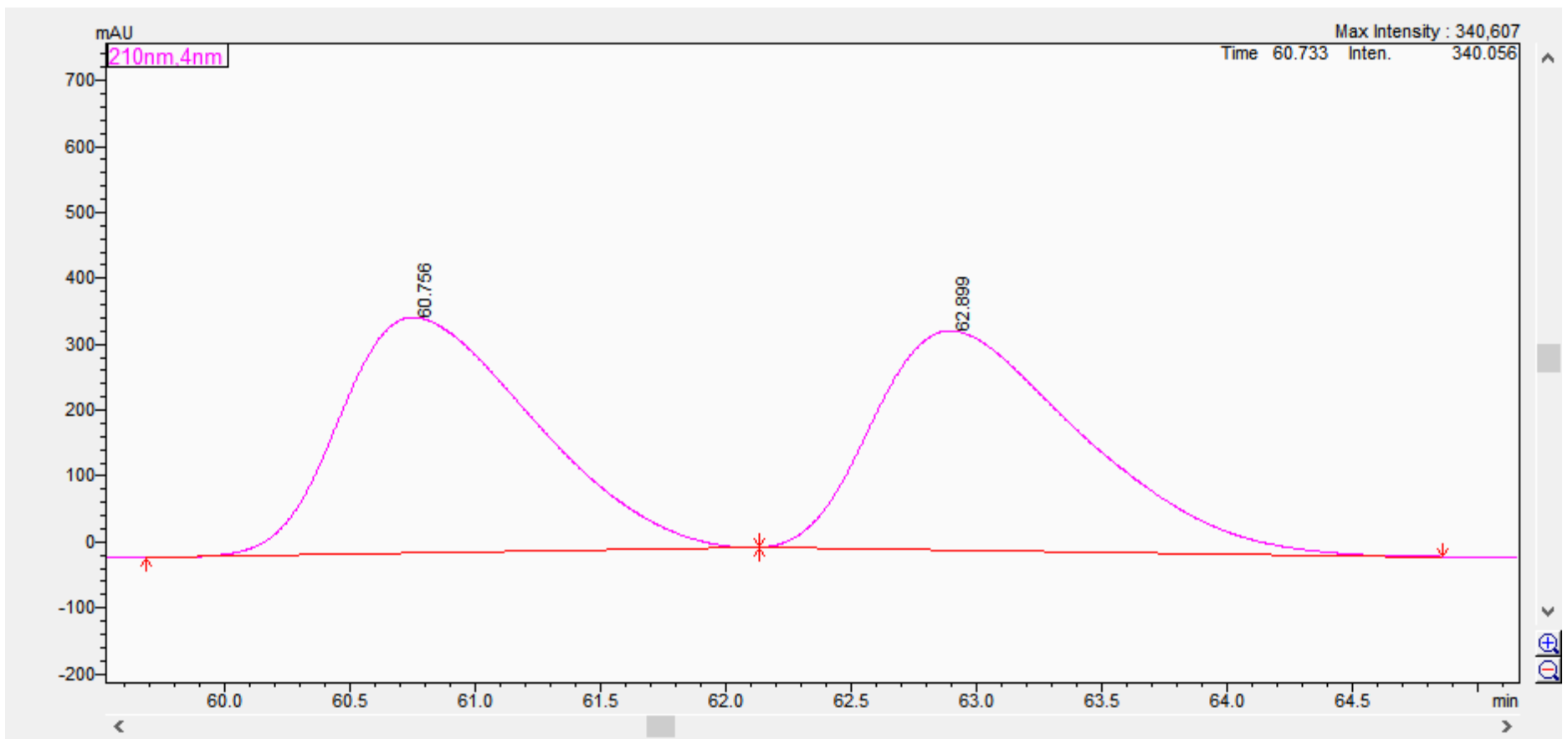

Peak#	Ret. Time	Area	Area%
1	60.756	19226598	50.043
2	62.899	19193452	49.957
Total		38420050	100.000

Enantioenriched (*R*)-**3d** (HPLC analysis):

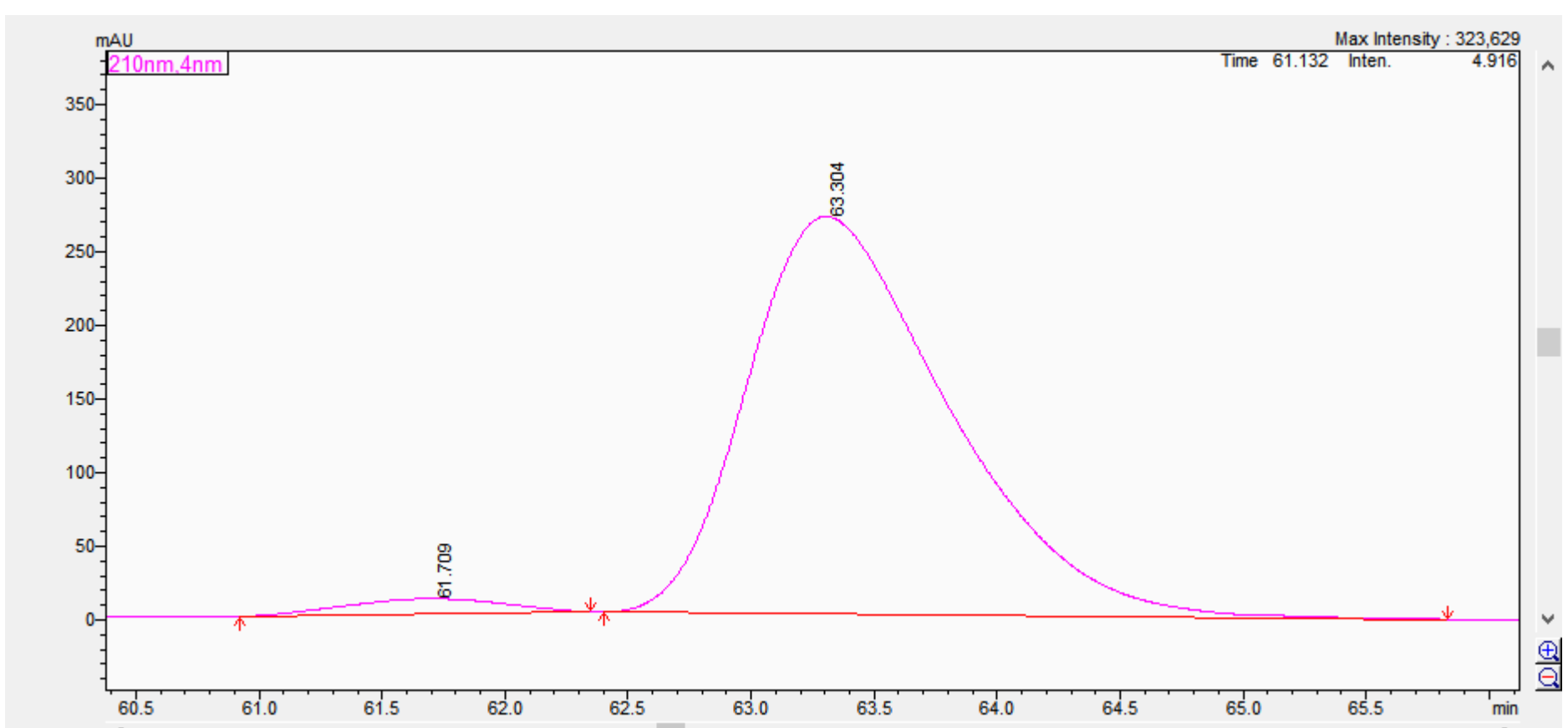

Peak#	Ret. Time	Area	Area%
1	61.709	468449	2.962
2	63.304	15348616	97.038
Total		15817065	100.000

(*R*)-2-Bromo-6-(2-(4-(methylthio)phenyl)propyl)pyridine (3e)

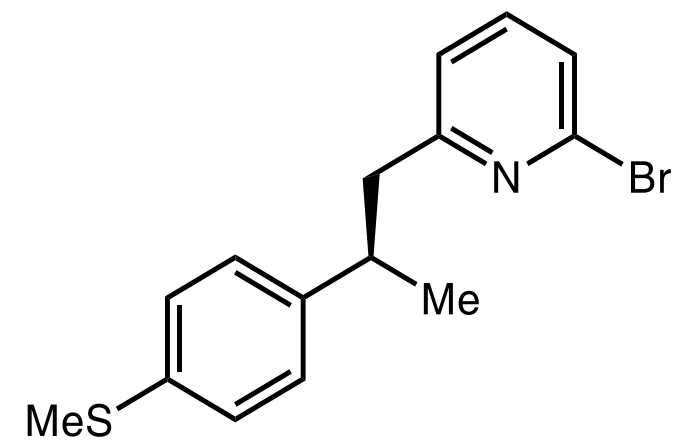

Racemic **3e** (HPLC analysis):

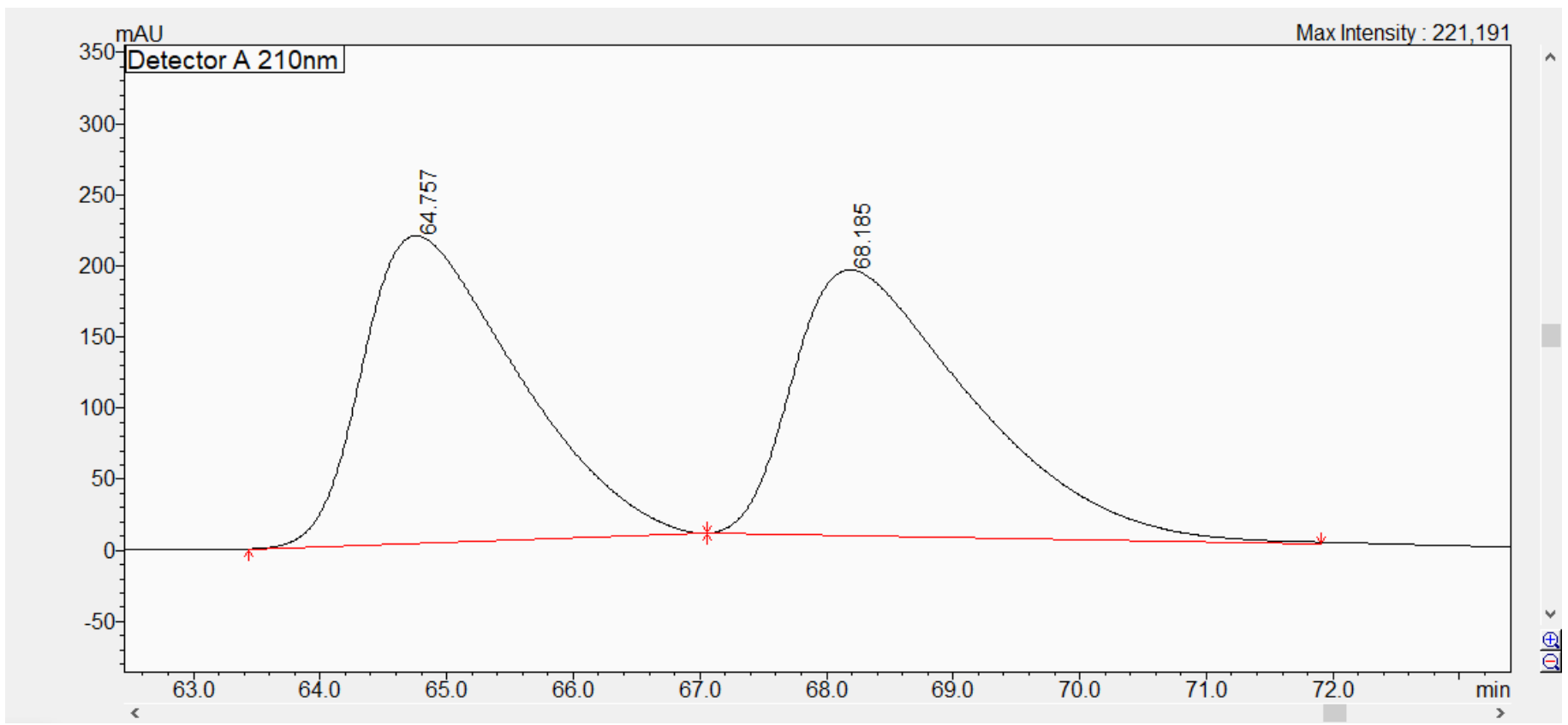

Peak#	Height	Ret. Time	Area	Area%
1	216151	64.757	18339022	49.938
2	186921	68.185	18384808	50.062
Total	403072		36723831	100.000

Enantioenriched (*R*)-**3e** (HPLC analysis):

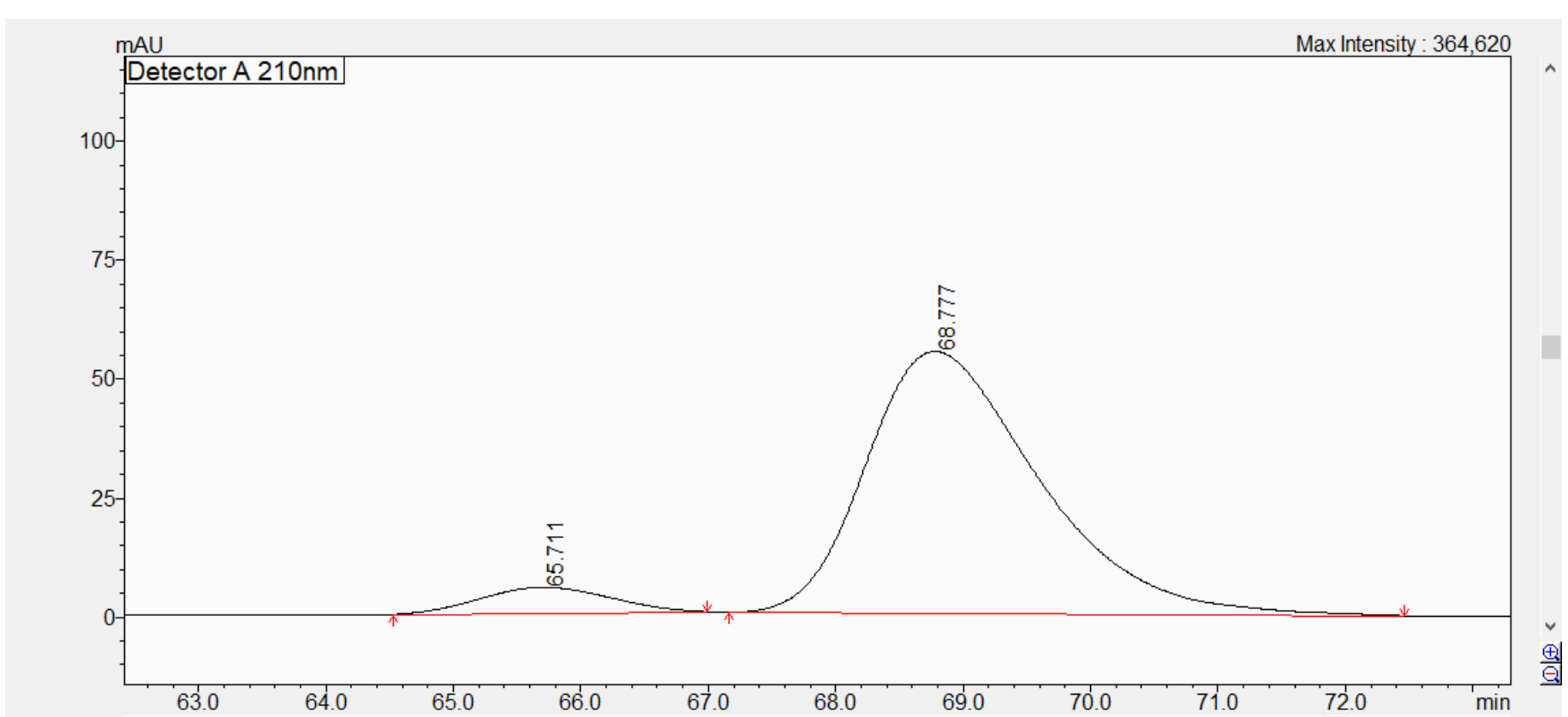

Peak#	Height	Ret. Time	Area	Area%
1	5443	65.711	385371	6.900
2	55073	68.777	5199560	93.100
Total	60516		5584931	100.000

(*R*)-2-Bromo-6-(2-(4-(trifluoromethoxy)phenyl)propyl)pyridine (3f)

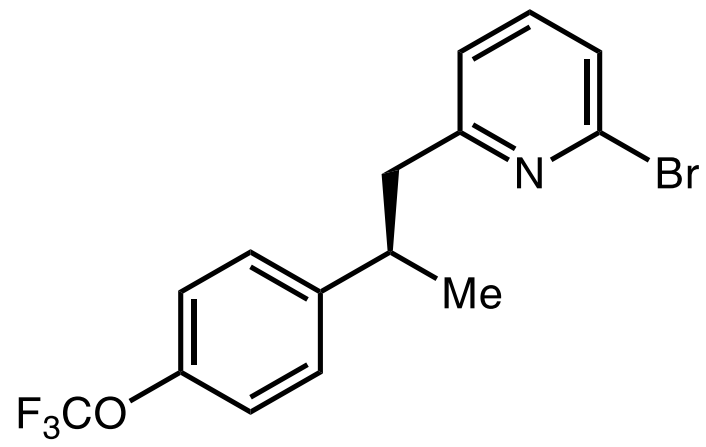

Racemic **3f** (HPLC analysis):

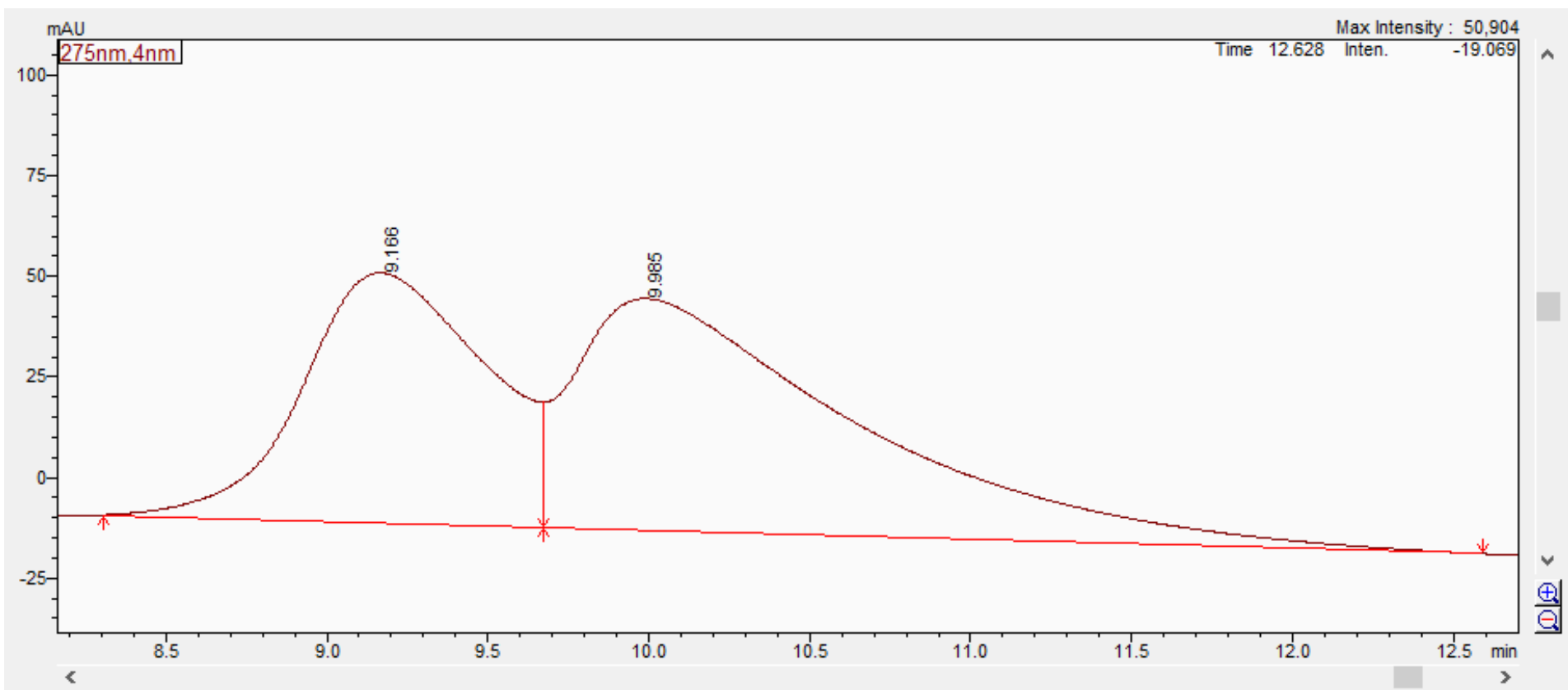

Peak#	Ret. Time	Area	Area%
1	9.166	2464613	41.290
2	9.985	3504370	58.710
Total		5968984	100.000

Enantioenriched (*R*)-**3f** (HPLC analysis):

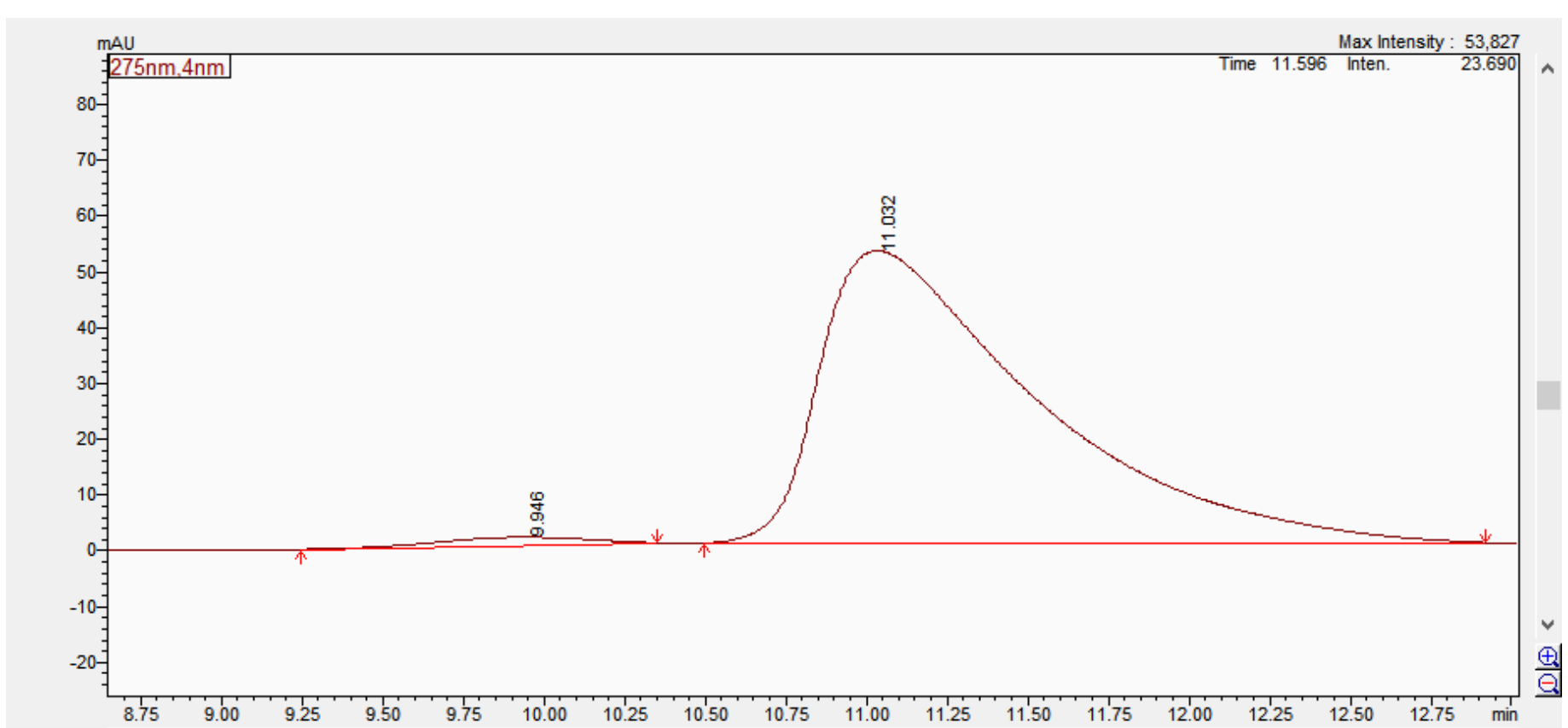

Peak#	Ret. Time	Area	Area%
1	9.946	47213	1.890
2	11.032	2451219	98.110
Total		2498432	100.000

(*R*)-2-bromo-6-(2-(4-fluorophenyl)propyl)pyridine (3g)

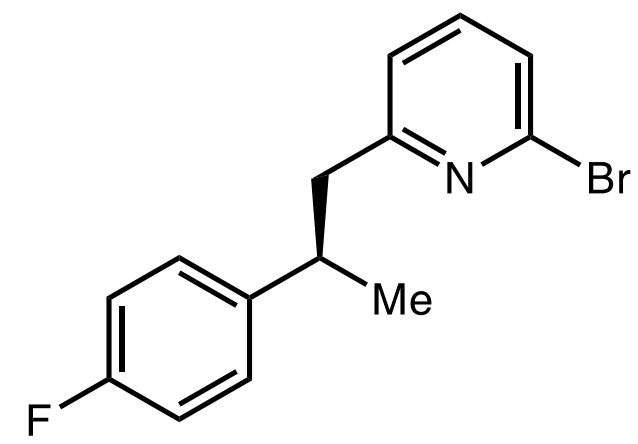

Racemic **3g** (HPLC analysis):

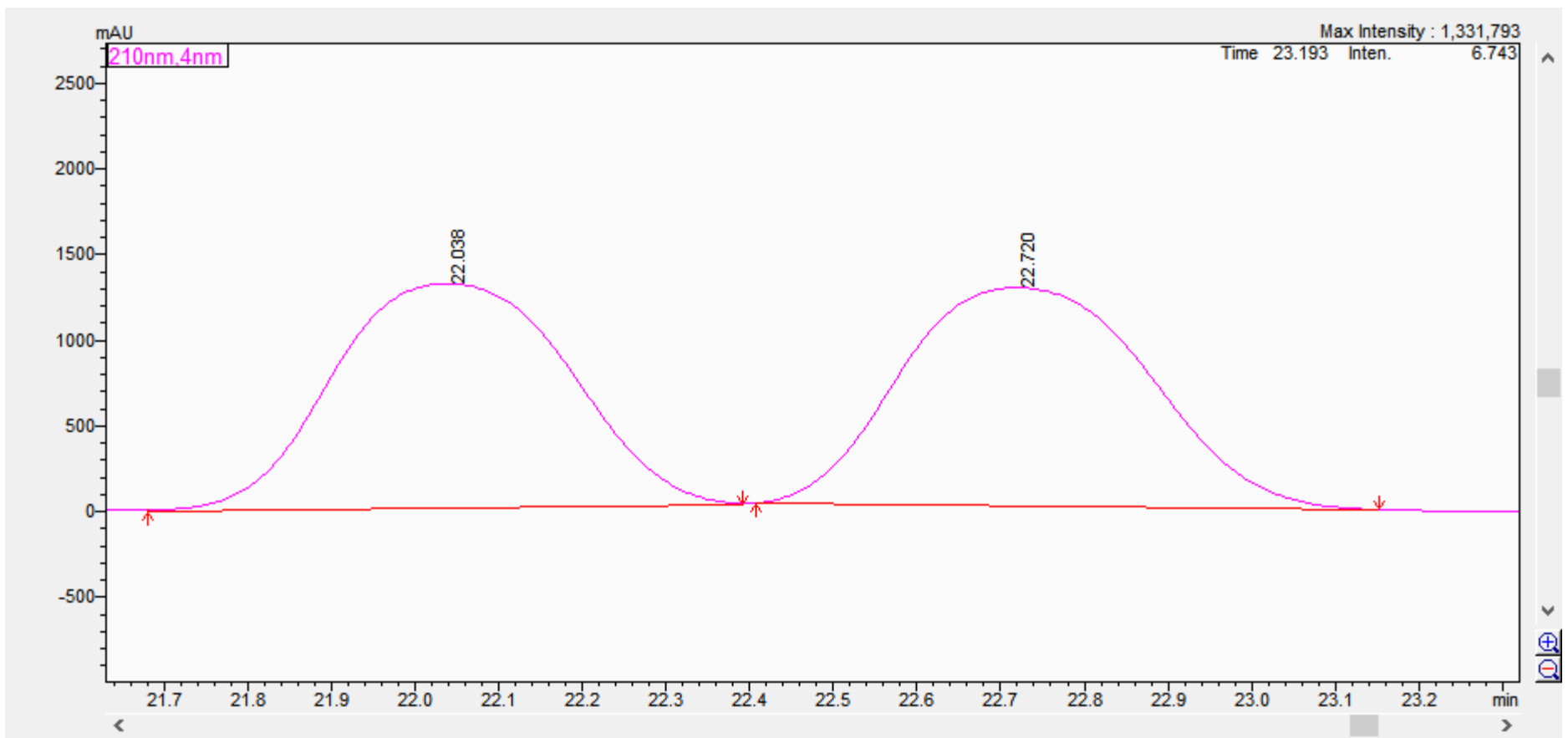

Peak#	Ret. Time	Area	Area%
1	22.038	25783000	49.769
2	22.720	26022284	50.231
Total		51805285	100.000

Enantioenriched (*R*)-**3g** (HPLC analysis):

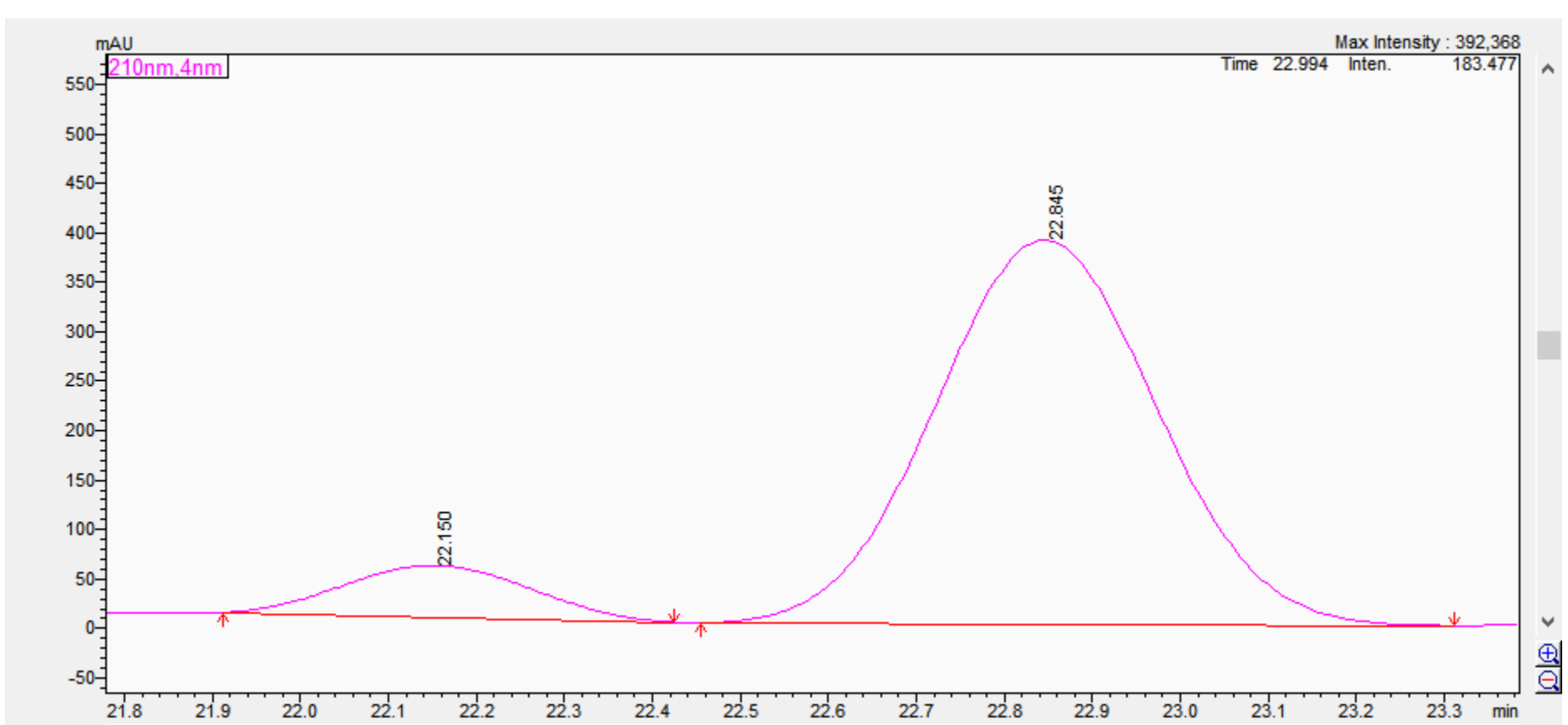

Peak#	Ret. Time	Area	Area%
1	22.150	762744	10.036
2	22.845	6837714	89.964
Total		7600458	100.000

(*R*)-2-Bromo-6-(2-(4-chlorophenyl)propyl)pyridine (3h)

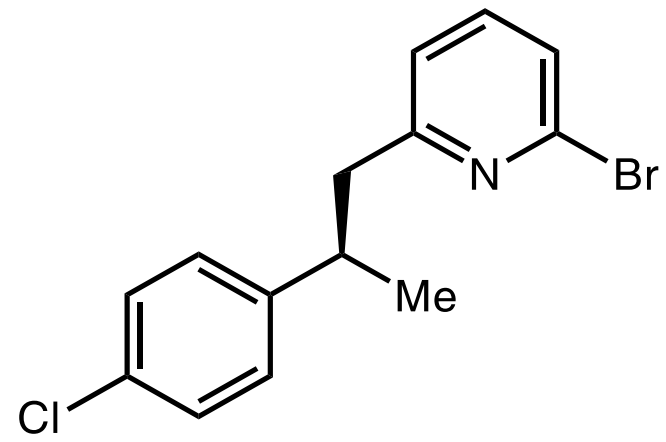

Racemic **3h** (HPLC analysis):

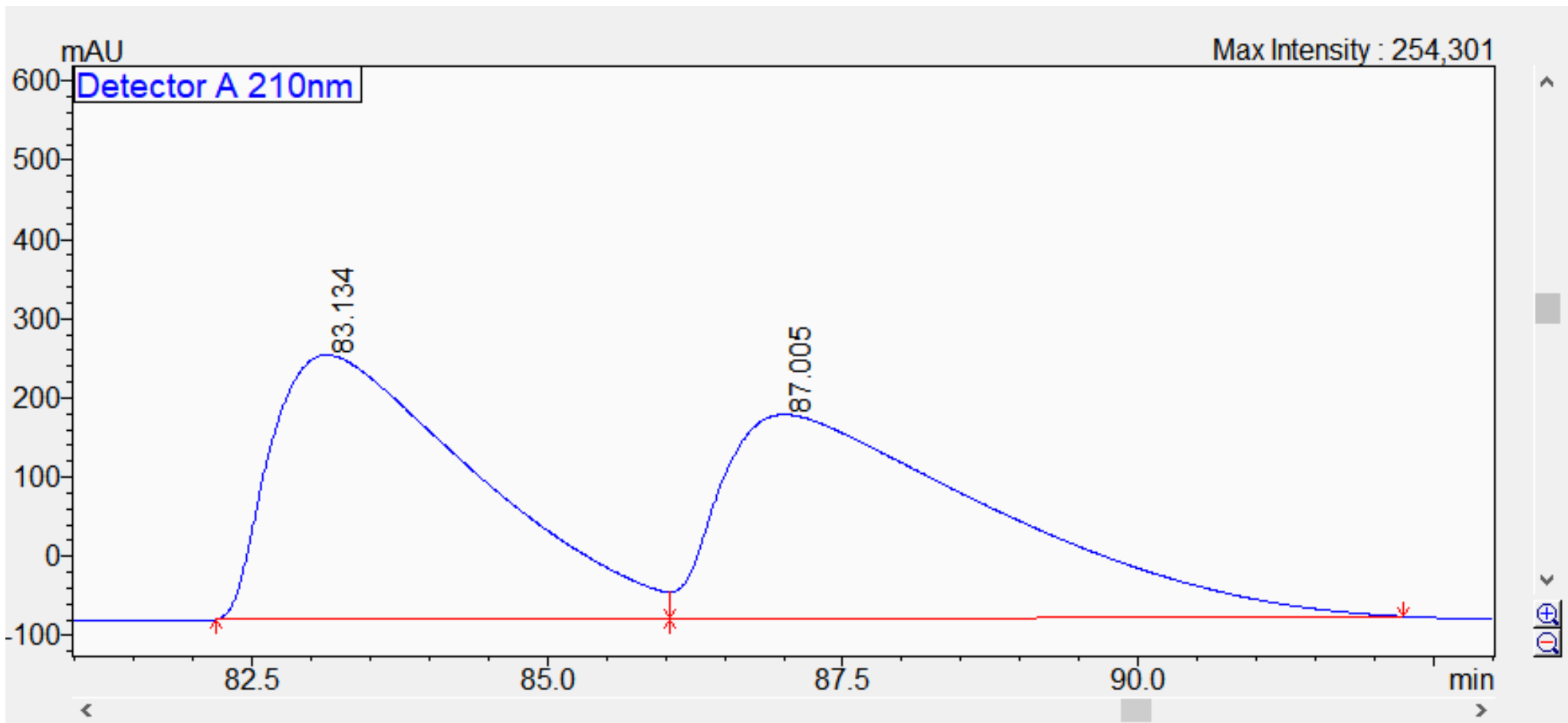

Peak#	Ret. Time	Area	Area%
1	83.134	40320824	49.461
2	87.005	41199337	50.539
Total		81520161	100.000

Enantioenriched (*R*)-**3h** (HPLC analysis):

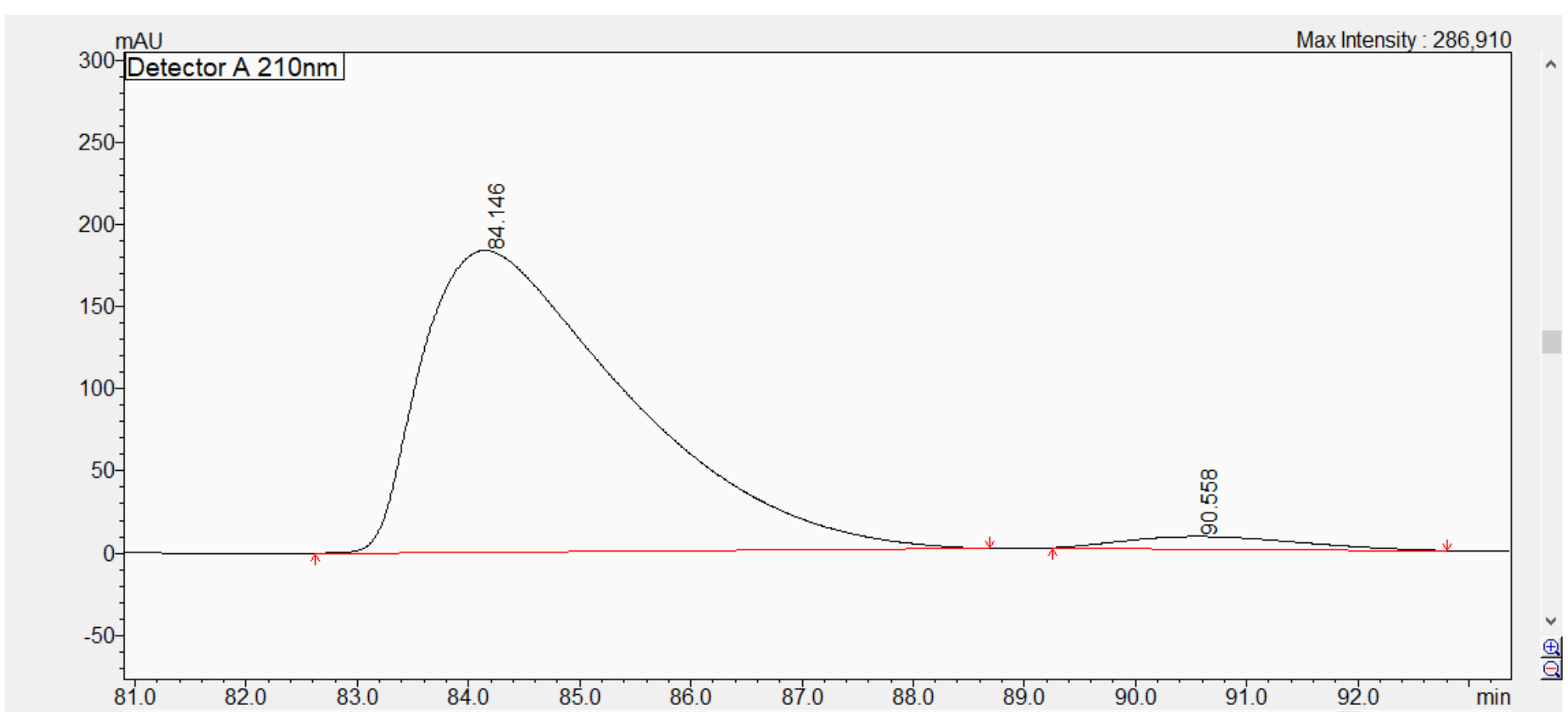

Peak#	Height	Ret. Time	Area	Area%
1	183449	84.146	23580947	96.659
2	7479	90.558	814993	3.341
Total	190928		24395940	100.000

(*R*)-2-bromo-6-(2-(4-bromophenyl)propyl)pyridine (3i)

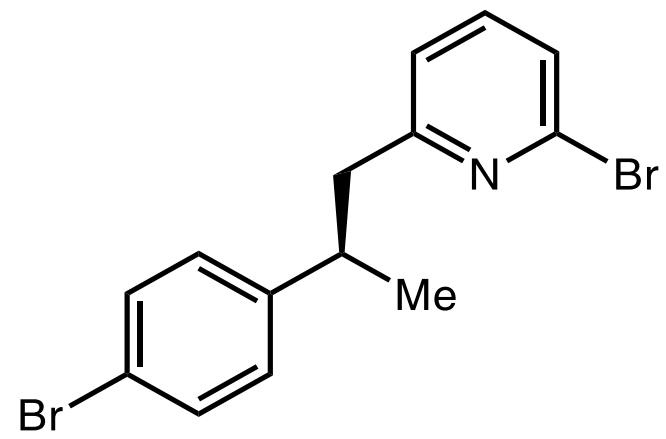

Racemic **3i** (HPLC analysis):

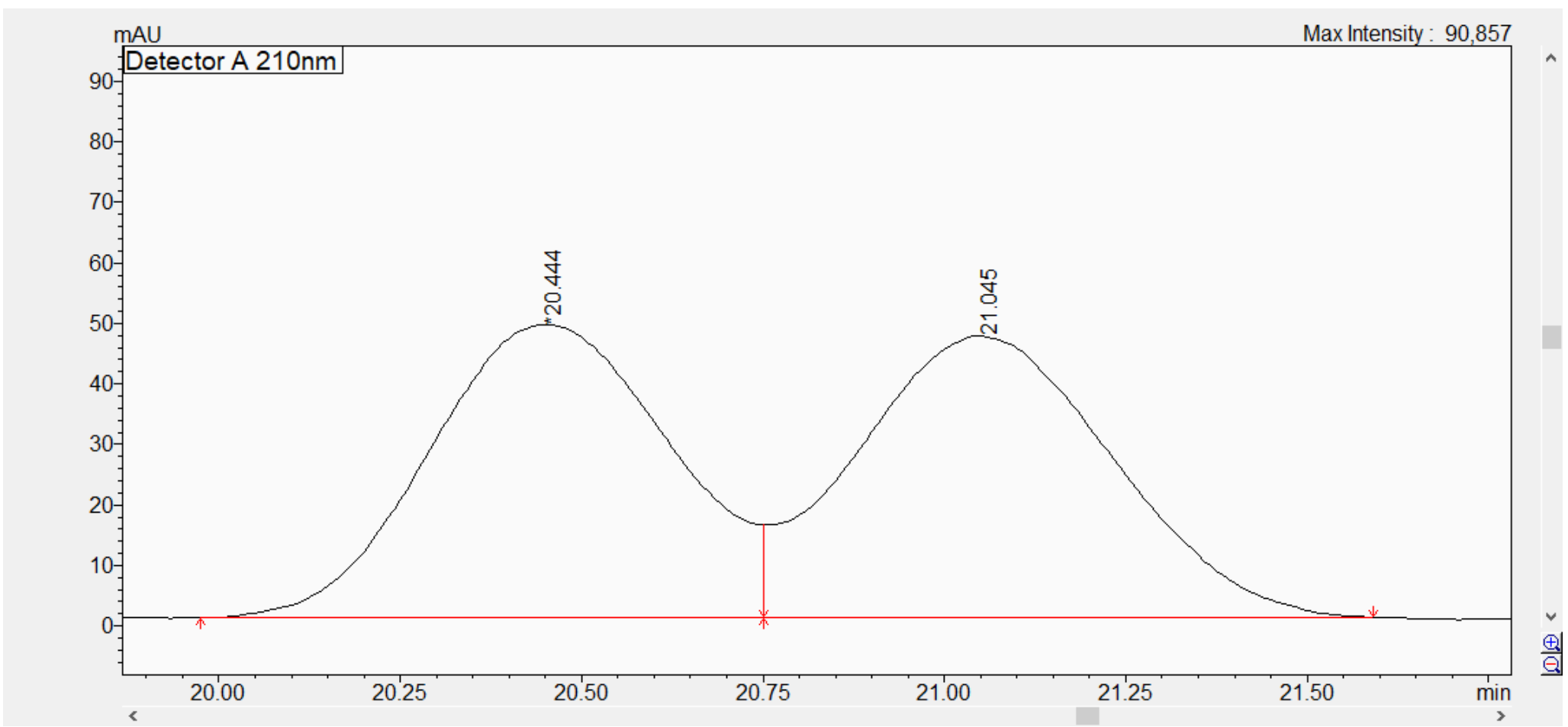

Peak#	Height	Ret. Time	Area	Area%
1	48364	20.444	1110962	49.384
2	46405	21.045	1138661	50.616
Total	94769		2249623	100.000

Enantioenriched (*R*)-**3i** (HPLC analysis):

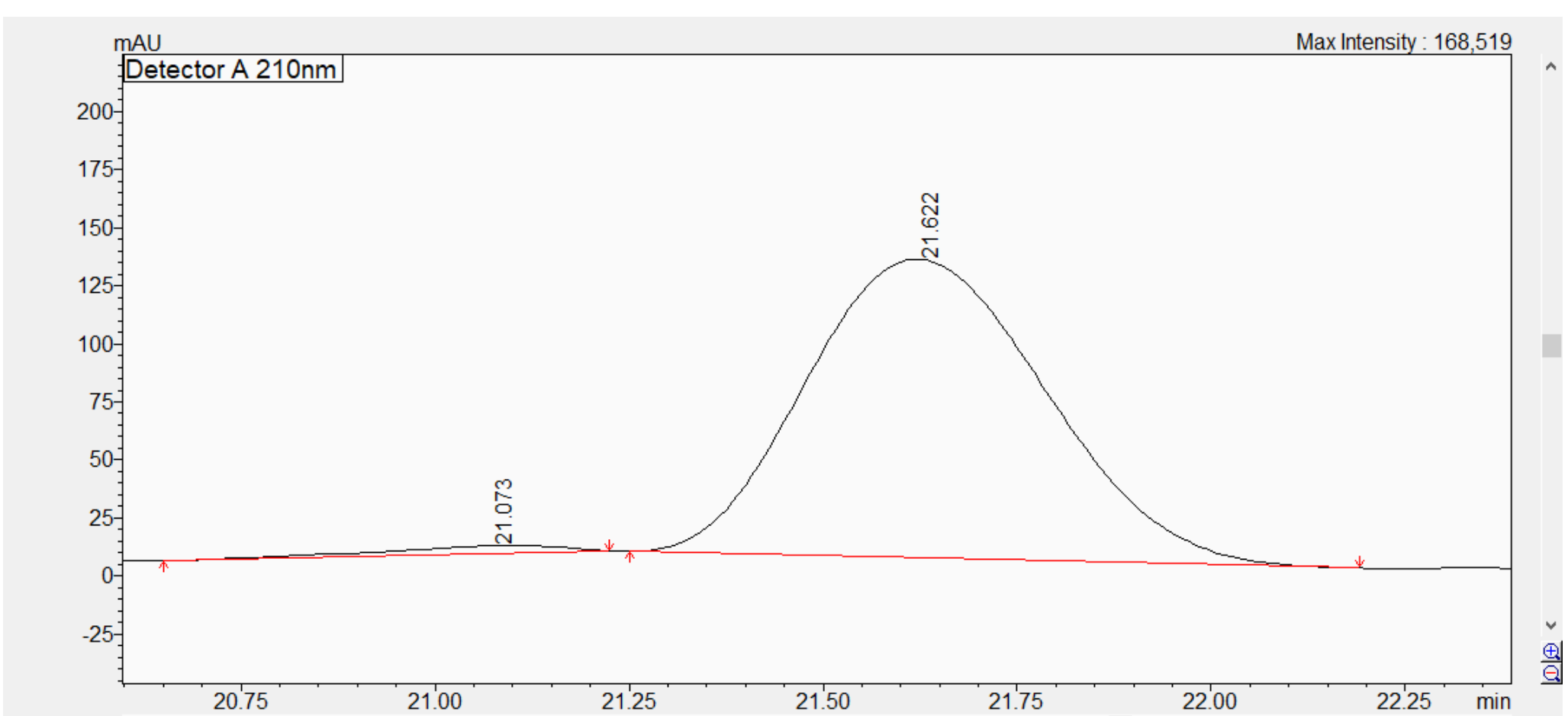

Peak#	Height	Ret. Time	Area	Area%
1	3341	21.073	54268	1.932
2	128467	21.622	2755024	98.068
Total	131808		2809292	100.000

(*R*)-2-bromo-6-(2-(4-iodophenyl)propyl)pyridine (3j)

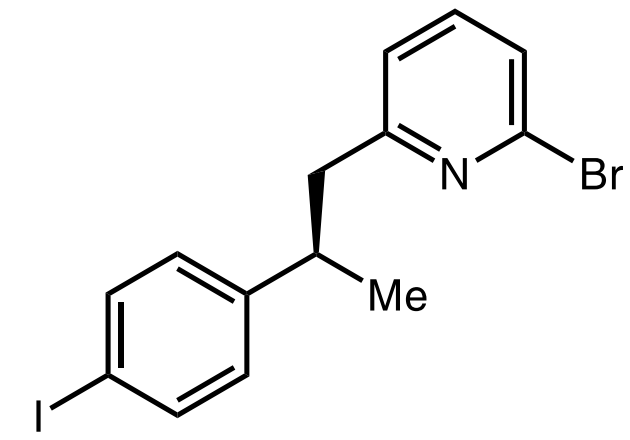

Racemic **3j** (HPLC analysis):

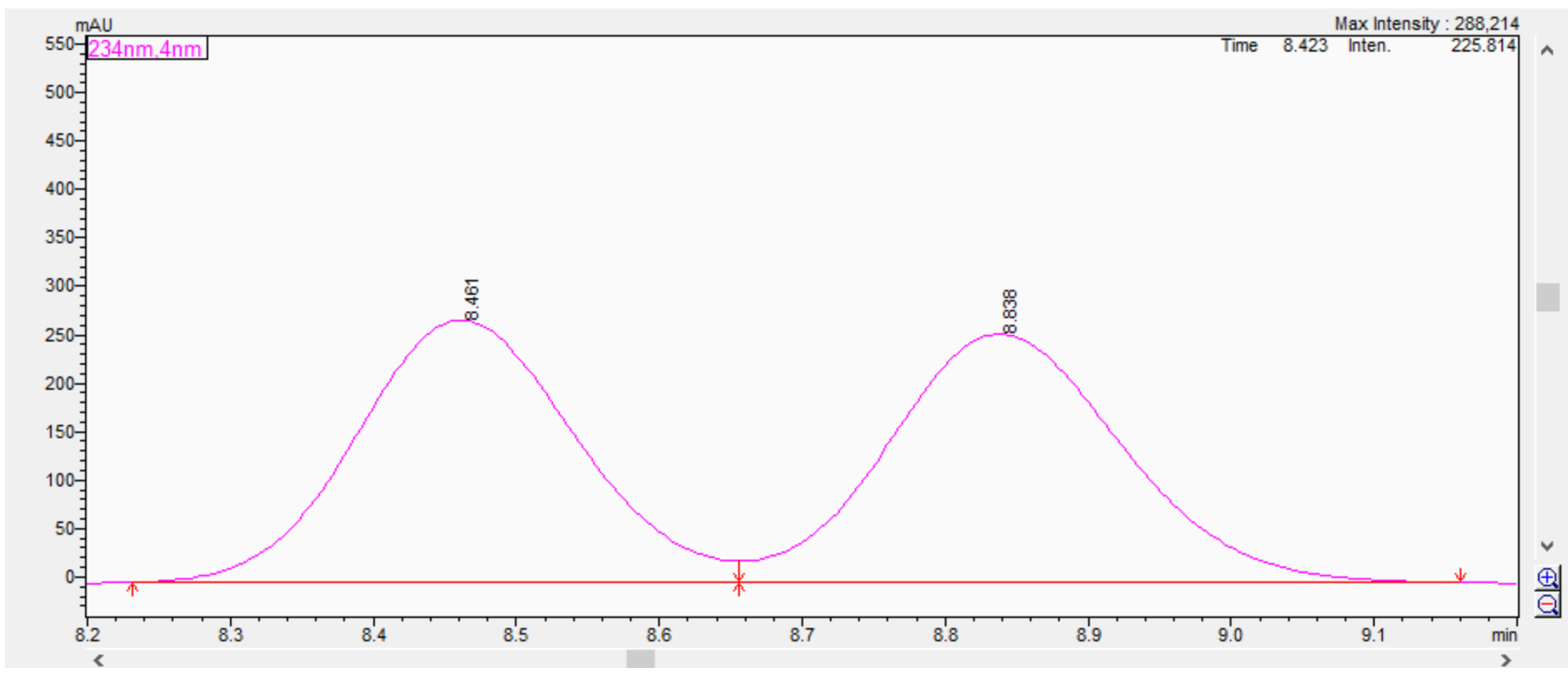

Peak#	Ret. Time	Area	Area%
1	8.461	2602507	50.069
2	8.838	2595378	49.931
Total		5197885	100.000

Enantioenriched (*R*)-**3j** (HPLC analysis):

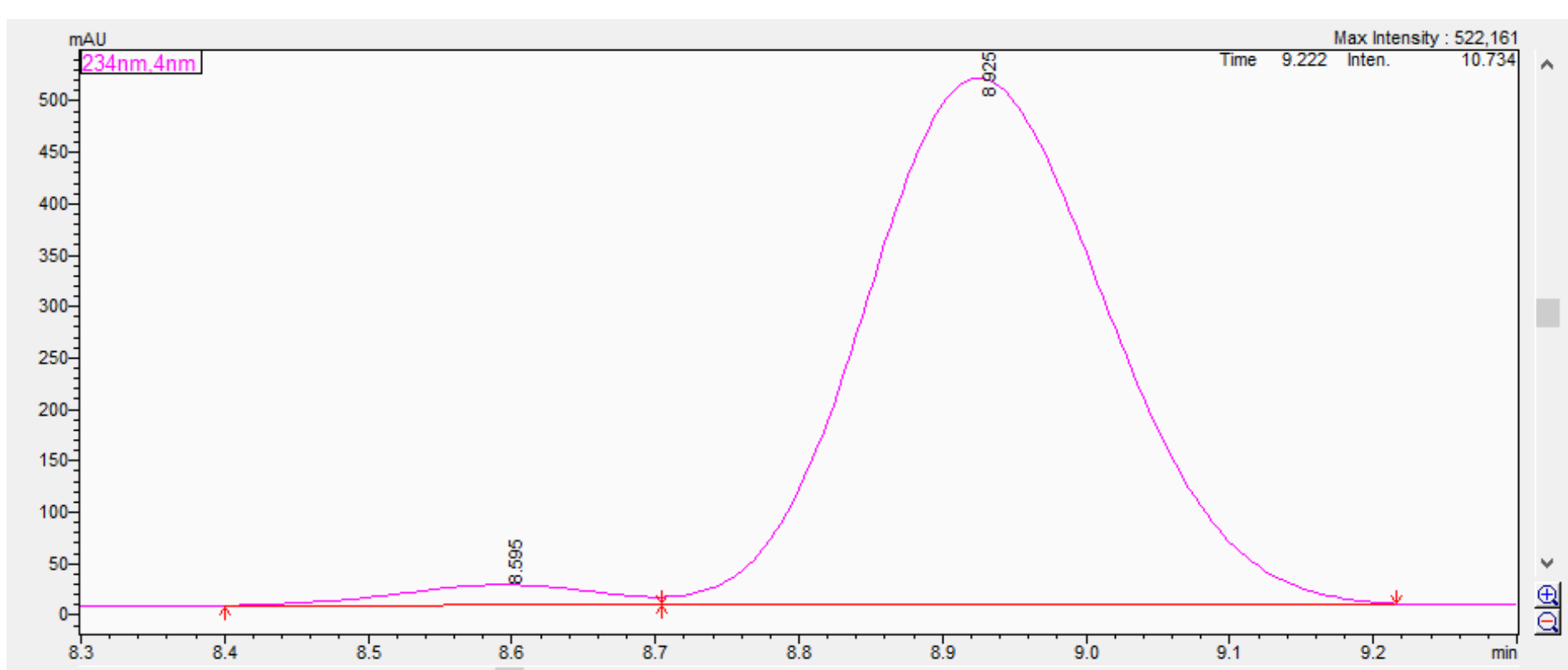

Peak#	Ret. Time	Area	Area%
1	8.595	194465	3.126
2	8.925	6025884	96.874
Total		6220349	100.000

(*R*)-2-Bromo-6-(2-(3-(trifluoromethyl)phenyl)propyl)pyridine (3k)

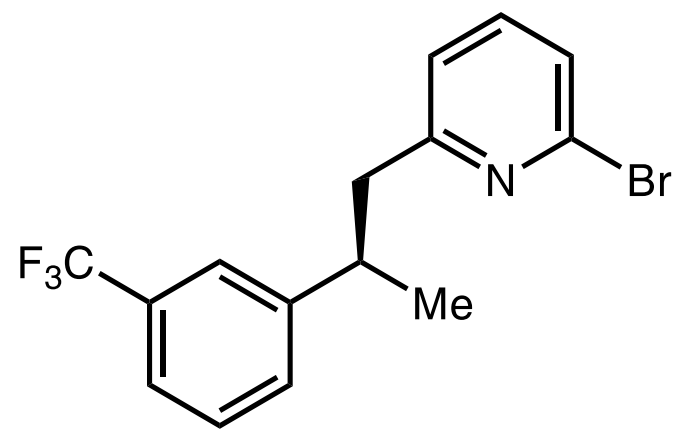

Racemic **3k** (HPLC analysis):

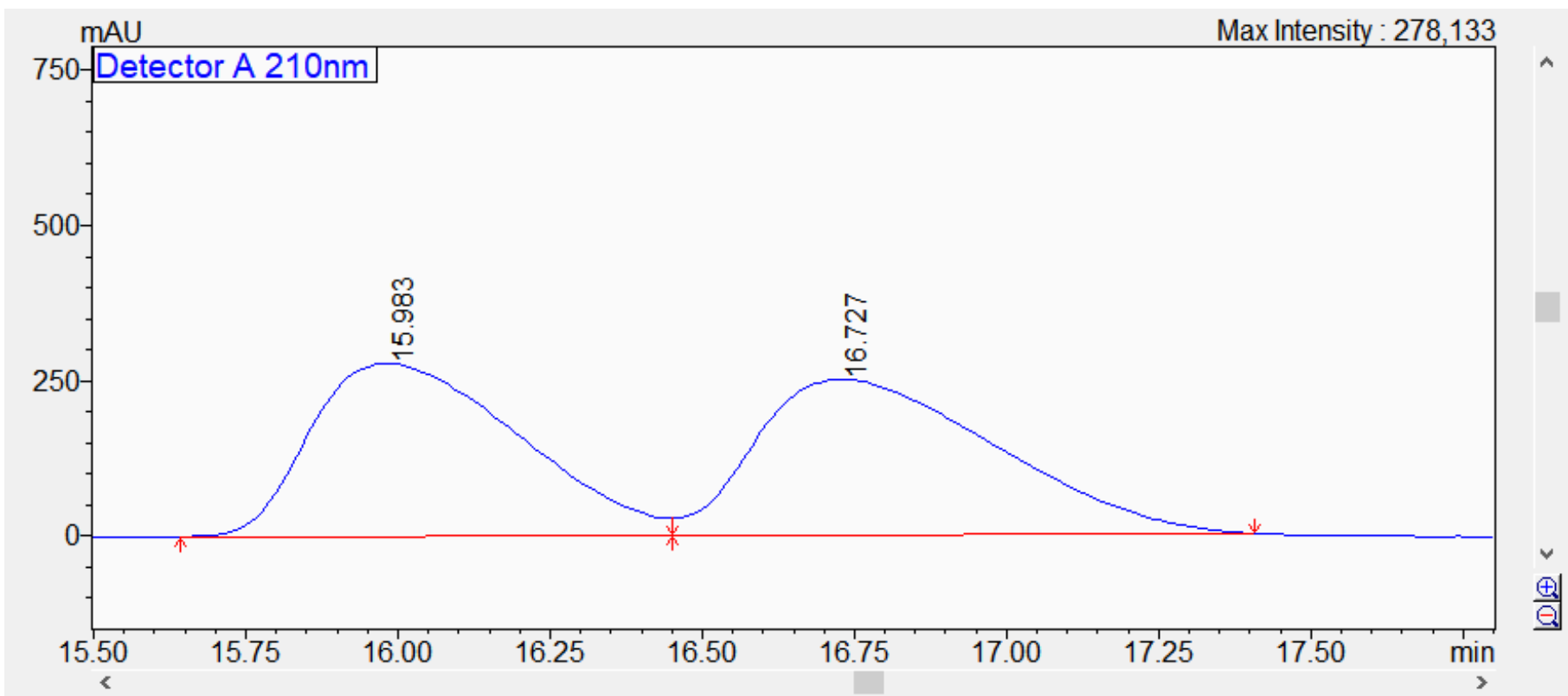

Peak#	Ret. Time	Area	Area%
1	15.983	6619299	49.121
2	16.727	6856151	50.879
Total		13475450	100.000

Enantioenriched (*R*)-**3k** (HPLC analysis):

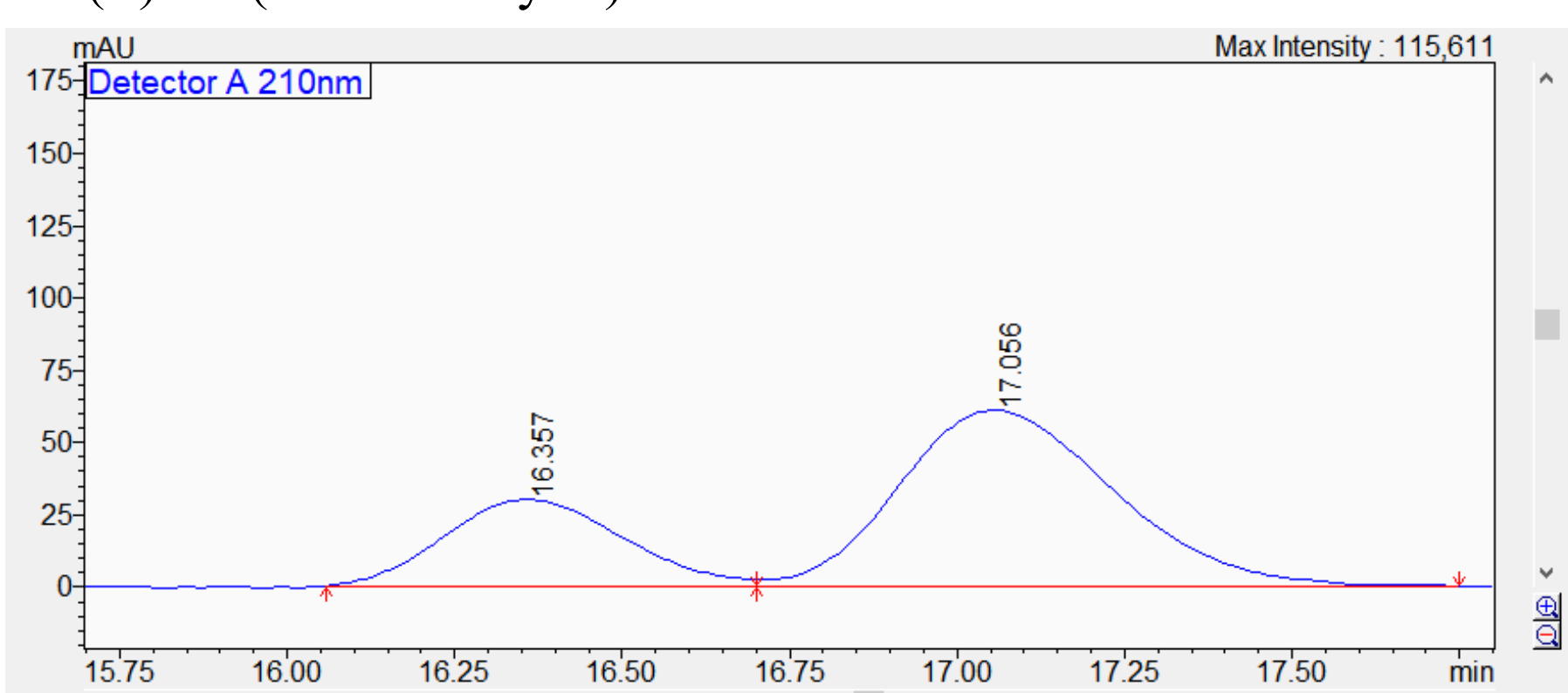

Peak#	Ret. Time	Area	Area%
1	16.357	552370	28.953
2	17.056	1355461	71.047
Total		1907831	100.000

(*R*)-5-(1-(6-bromopyridin-2-yl)propan-2-yl)-2-methoxypyridine (3l)

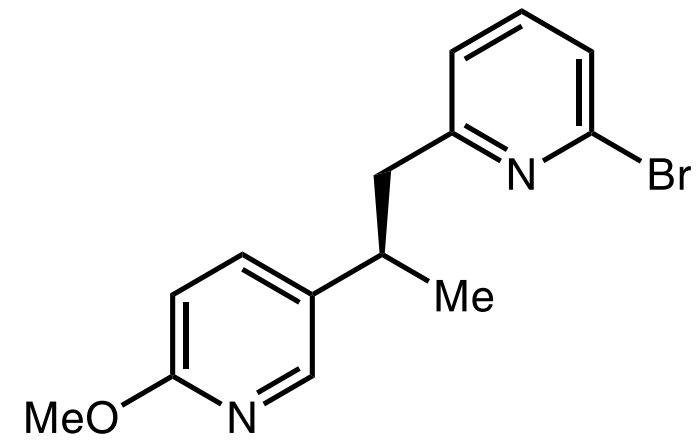

Racemic **3l** (HPLC analysis):

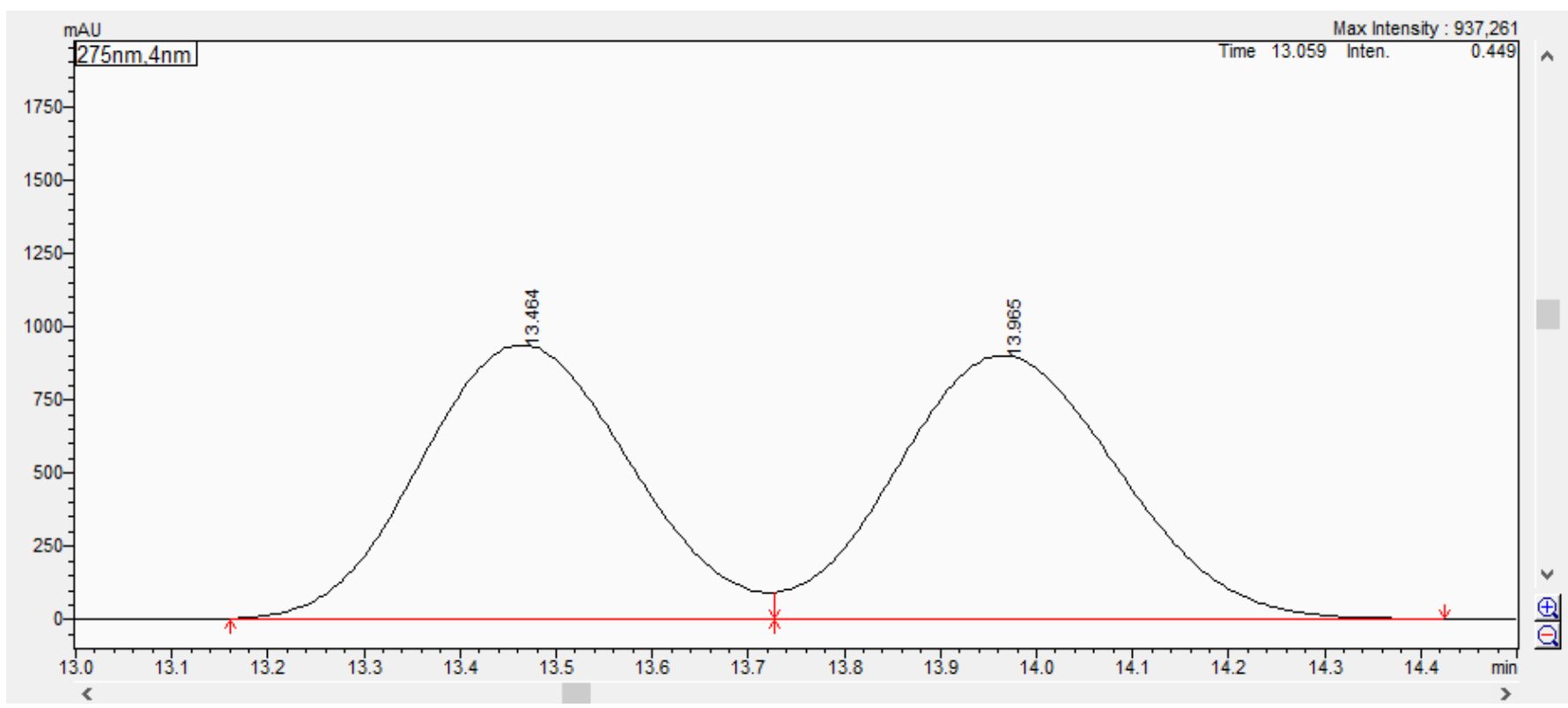

Peak#	Ret. Time	Area	Area%
1	13.464	14237739	49.644
2	13.965	14441718	50.356
Total		28679457	100.000

Enantioenriched (*R*)-**3l** (HPLC analysis):

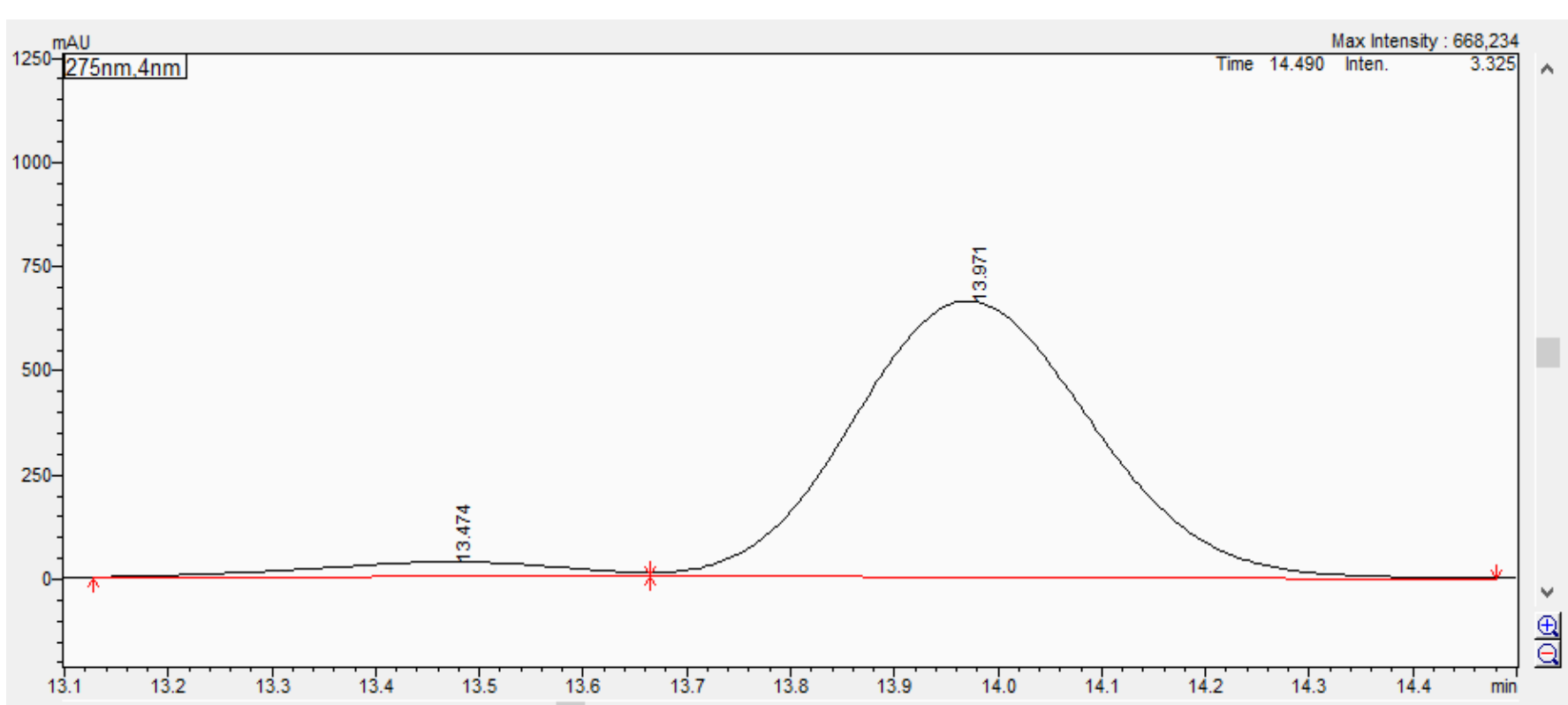

Peak#	Ret. Time	Area	Area%
1	13.474	568493	5.073
2	13.971	10637119	94.927
Total		11205612	100.000

(*R*)-2-Bromo-6-(2-phenylbutyl)pyridine (3m)

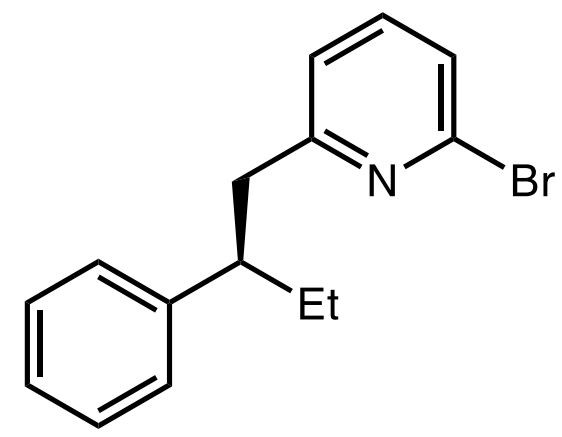

Racemic **3m** (HPLC analysis):

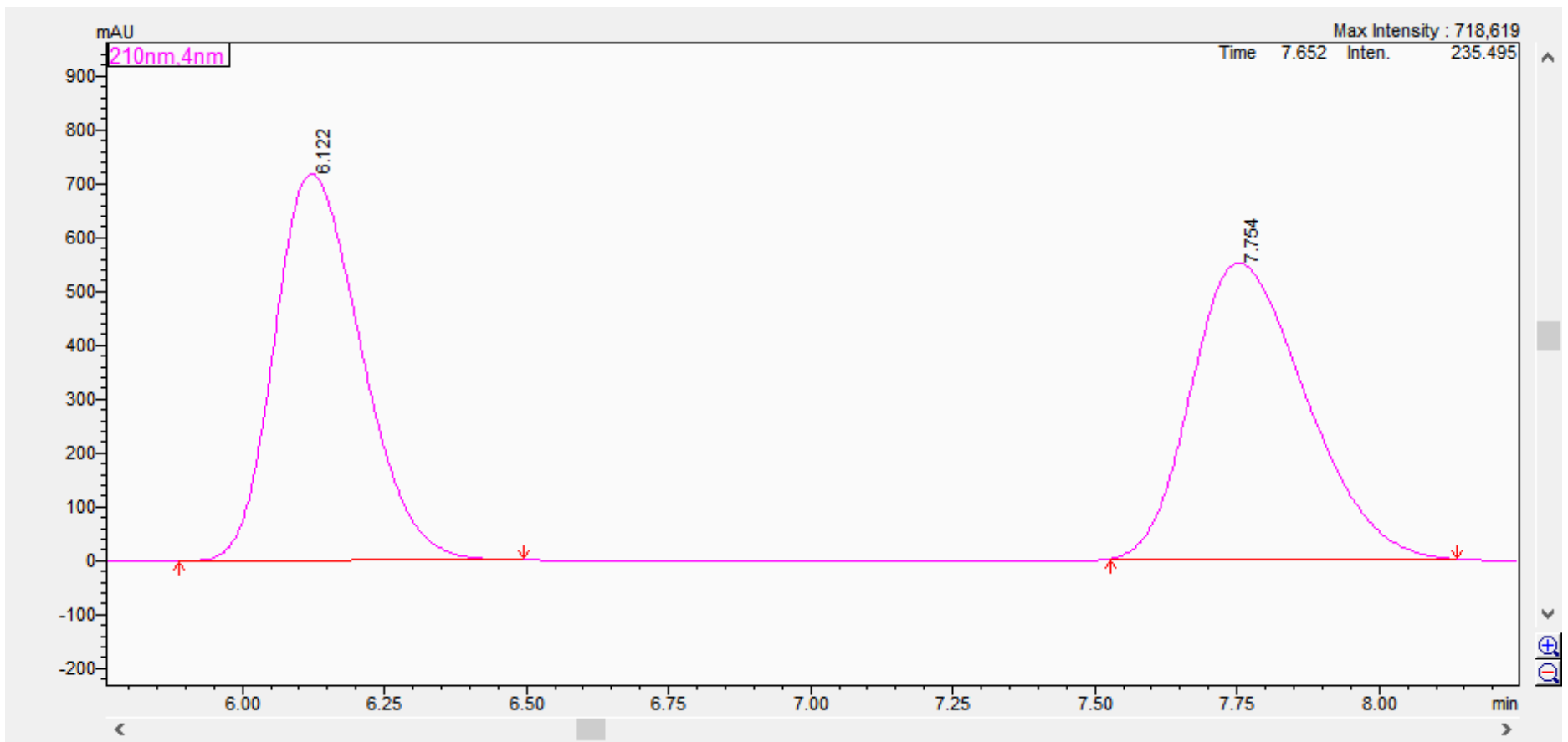

Peak#	Ret. Time	Area	Area%
1	6.122	7634337	49.771
2	7.754	7704591	50.229
Total		15338928	100.000

Enantioenriched (*R*)-**3m** (HPLC analysis):

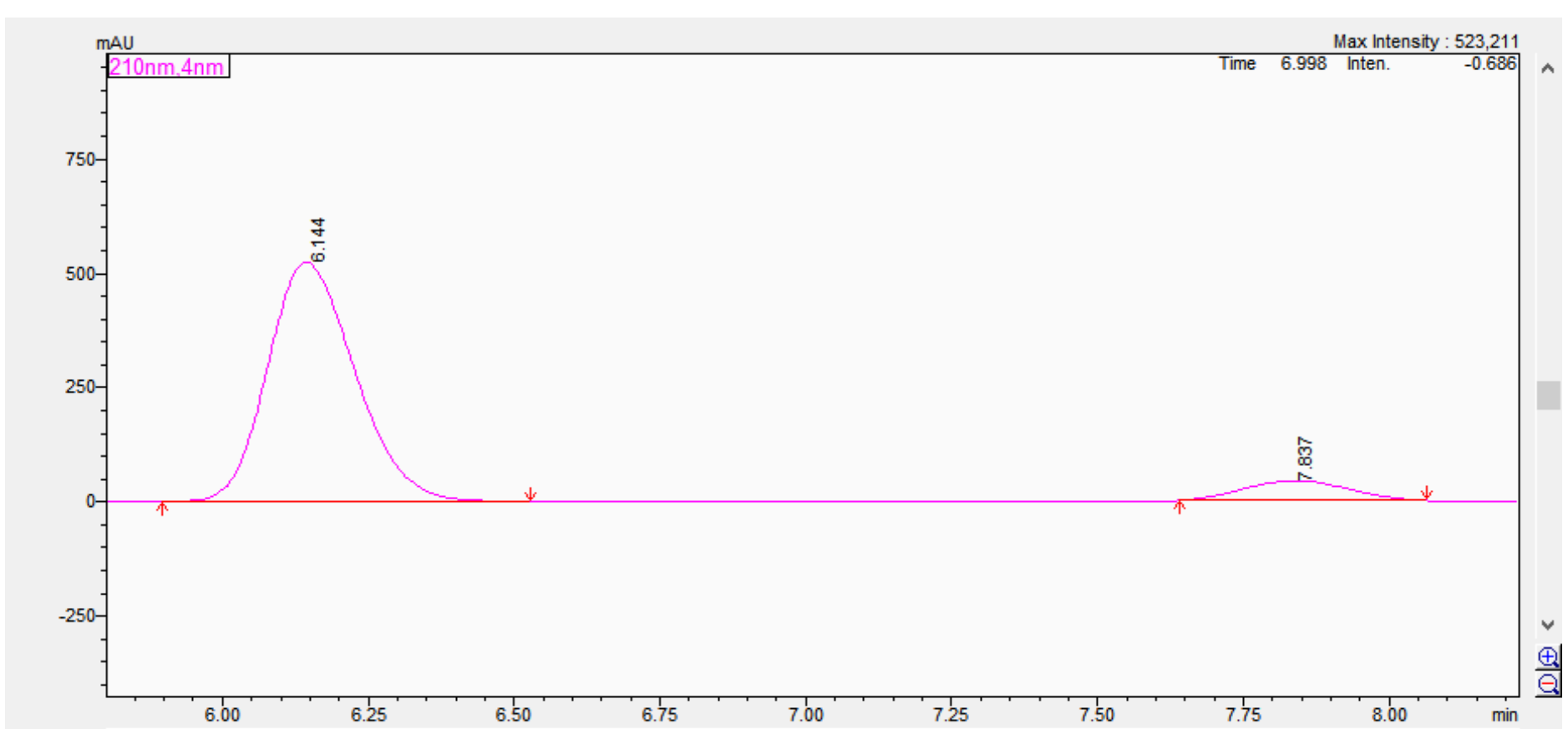

Peak#	Ret. Time	Area	Area%
1	6.144	5447947	90.941
2	7.837	542689	9.059
Total		5990636	100.000

(*R*)-2-bromo-6-(2-phenylpentyl)pyridine (3n)

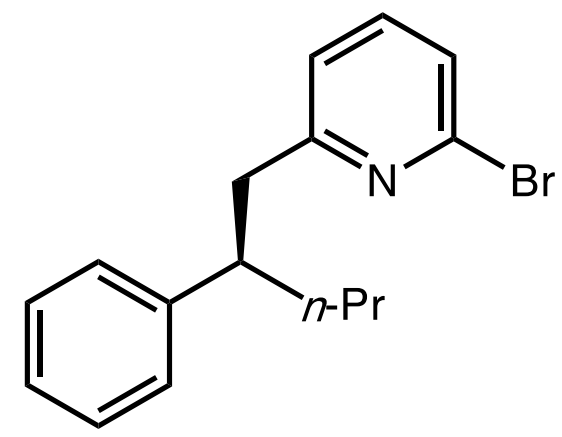

Racemic **3n** (HPLC analysis):

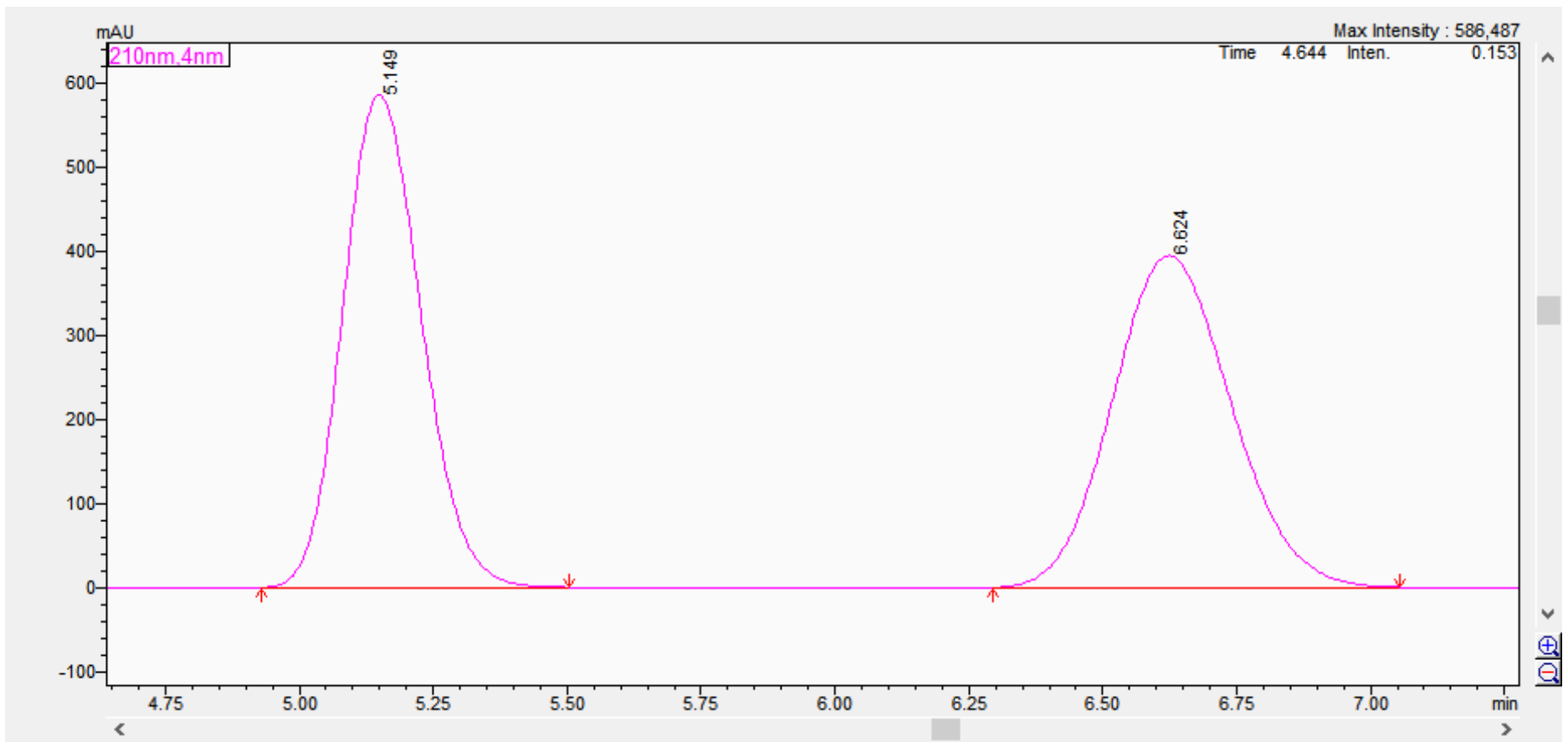

Peak#	Ret. Time	Area	Area%
1	5.149	5975239	49.502
2	6.624	6095548	50.498
Total		12070787	100.000

Enantioenriched (*R*)-**3n** (HPLC analysis):

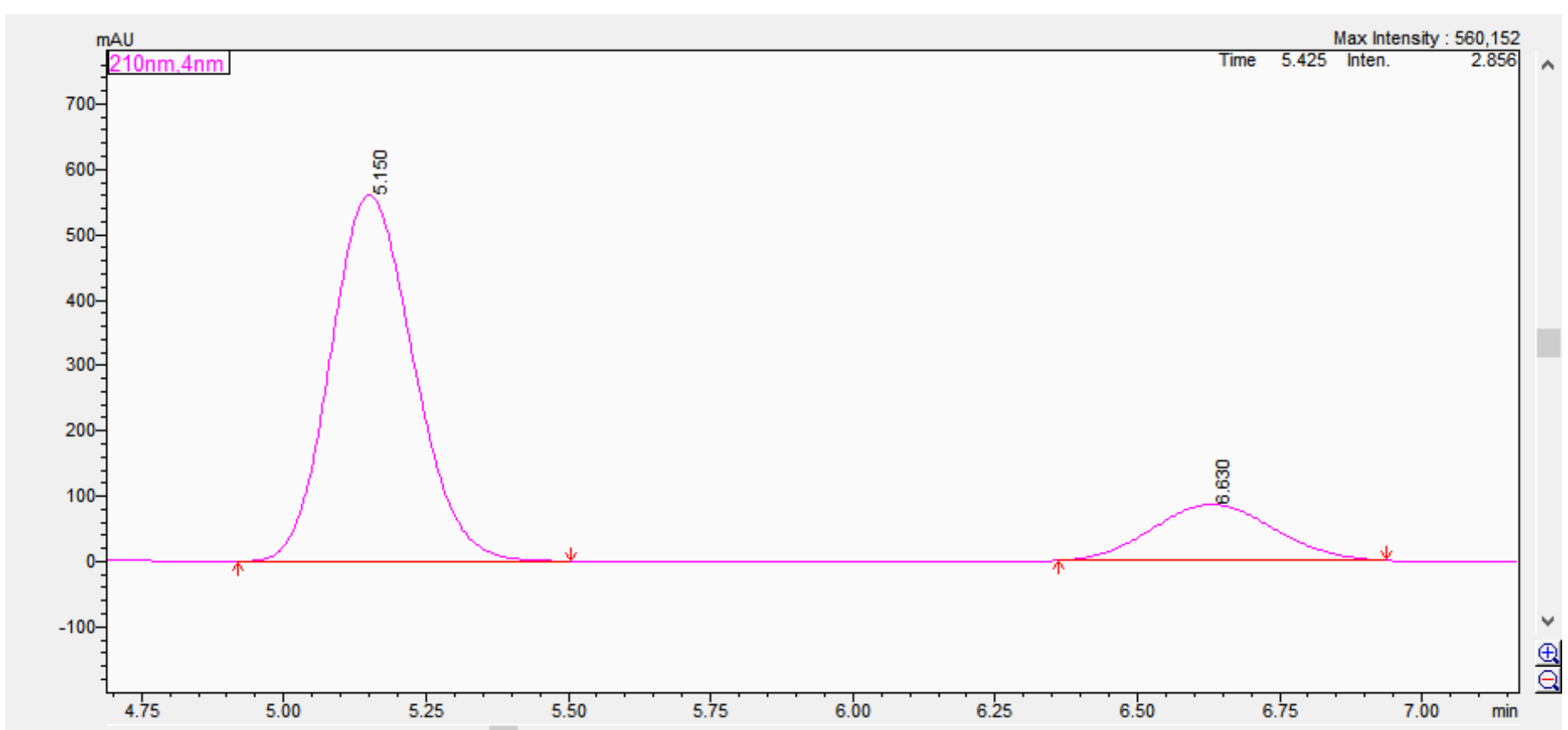

Peak#	Ret. Time	Area	Area%
1	5.150	5678078	81.649
2	6.630	1276200	18.351
Total		6954278	100.000

(*R*)-2-bromo-6-((2,3-dihydro-1*H*-inden-1-yl)methyl)pyridine (3o)

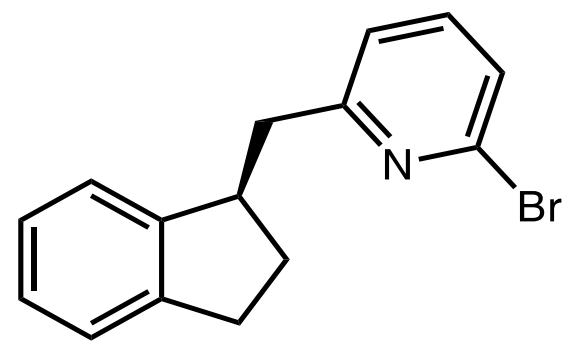

Racemic **3o** (HPLC analysis):

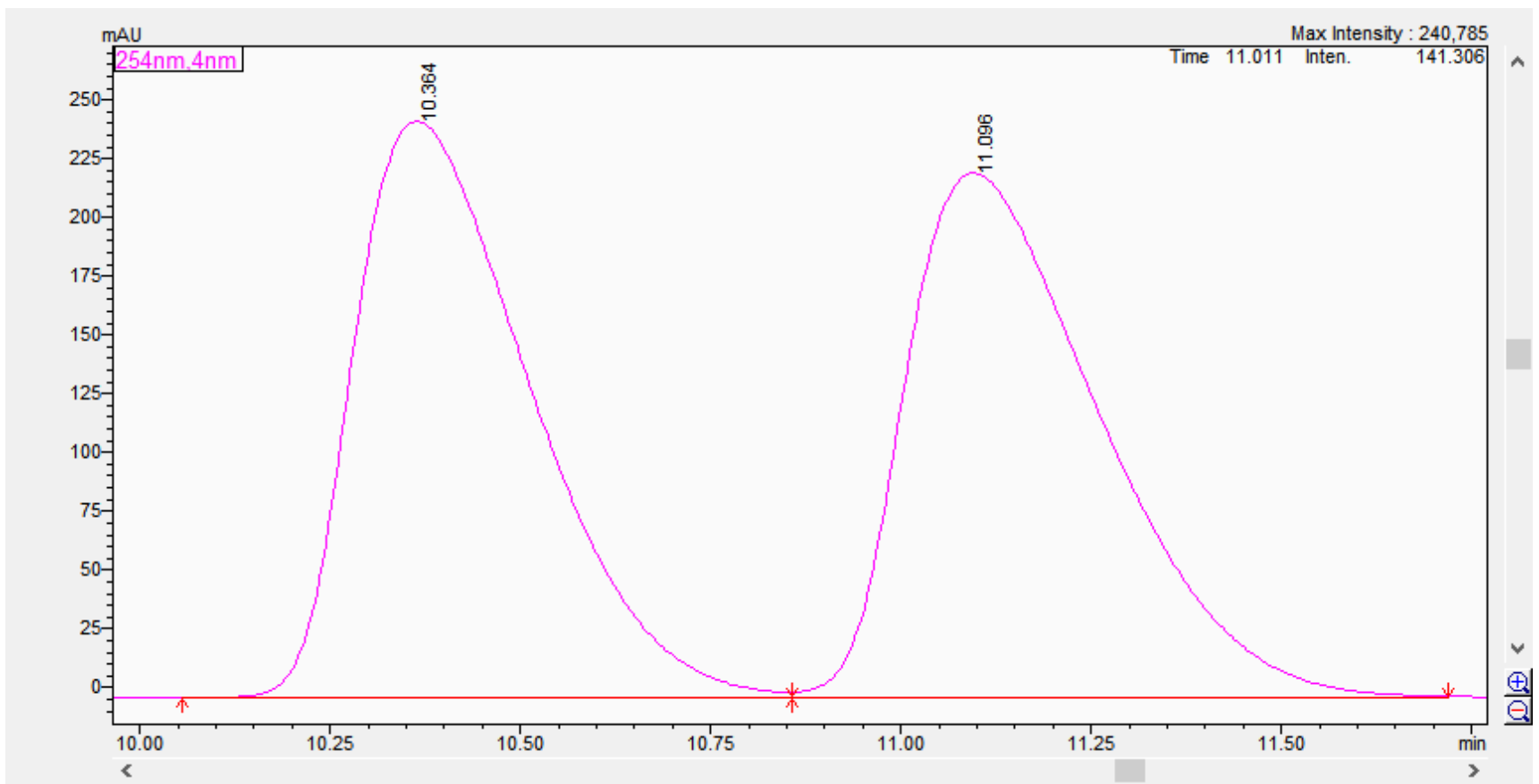

Peak#	Ret. Time	Area	Area%
1	10.364	3962942	49.880
2	11.096	3981992	50.120
Total		7944935	100.000

Enantioenriched (*R*)-**3o** (HPLC analysis):

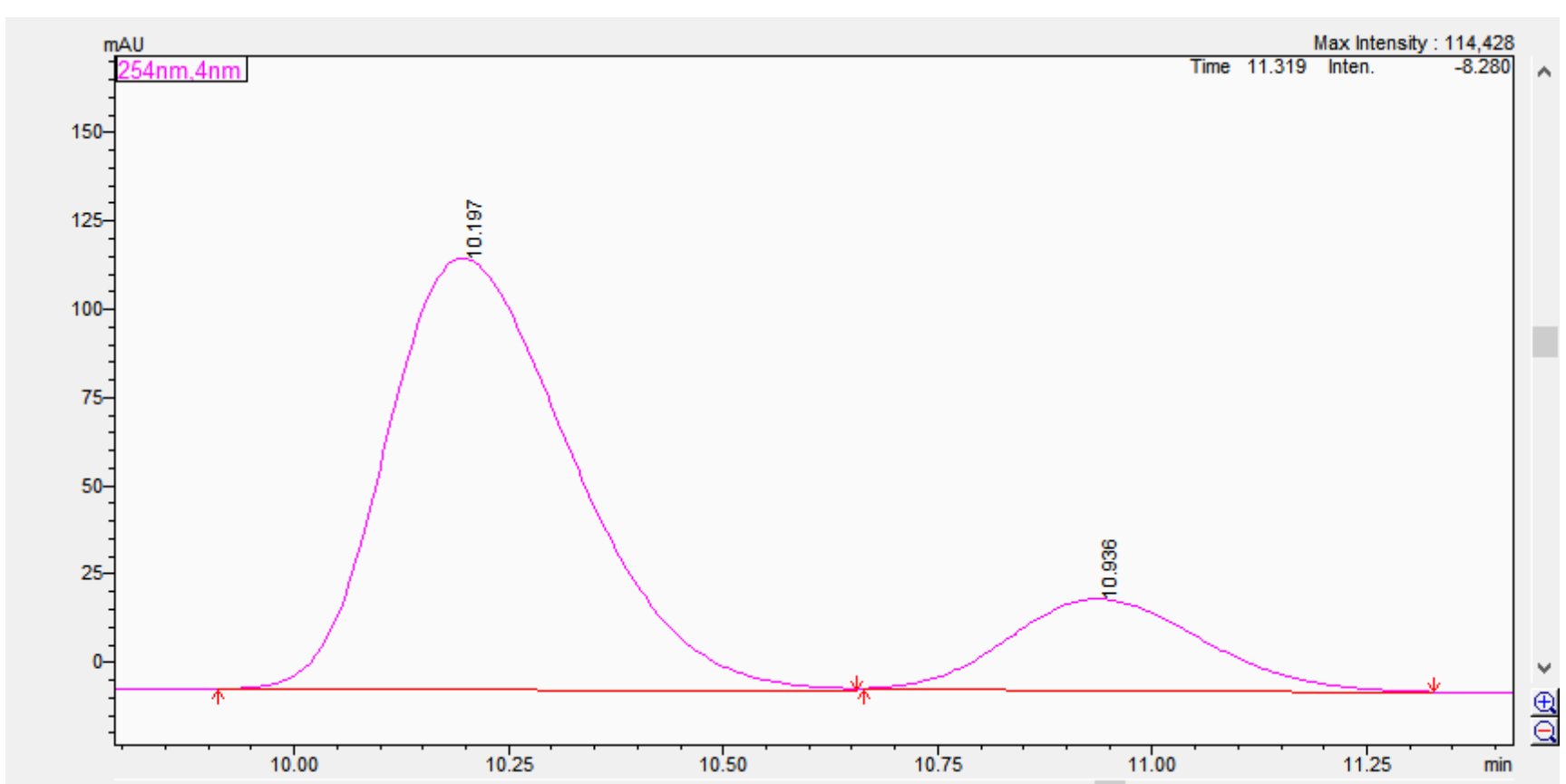

Peak#	Ret. Time	Area	Area%
1	10.197	1845006	82.219
2	10.936	399008	17.781
Total		2244014	100.000

(*R*)-2-Bromo-6-((1,2,3,4-tetrahydronaphthalen-1-yl)methyl)pyridine (3p)

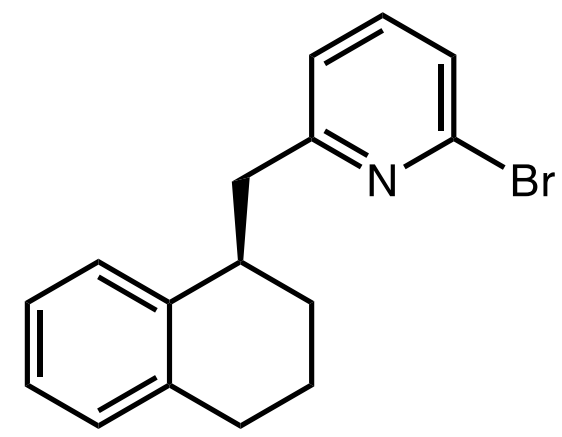

Racemic **3p** (HPLC analysis):

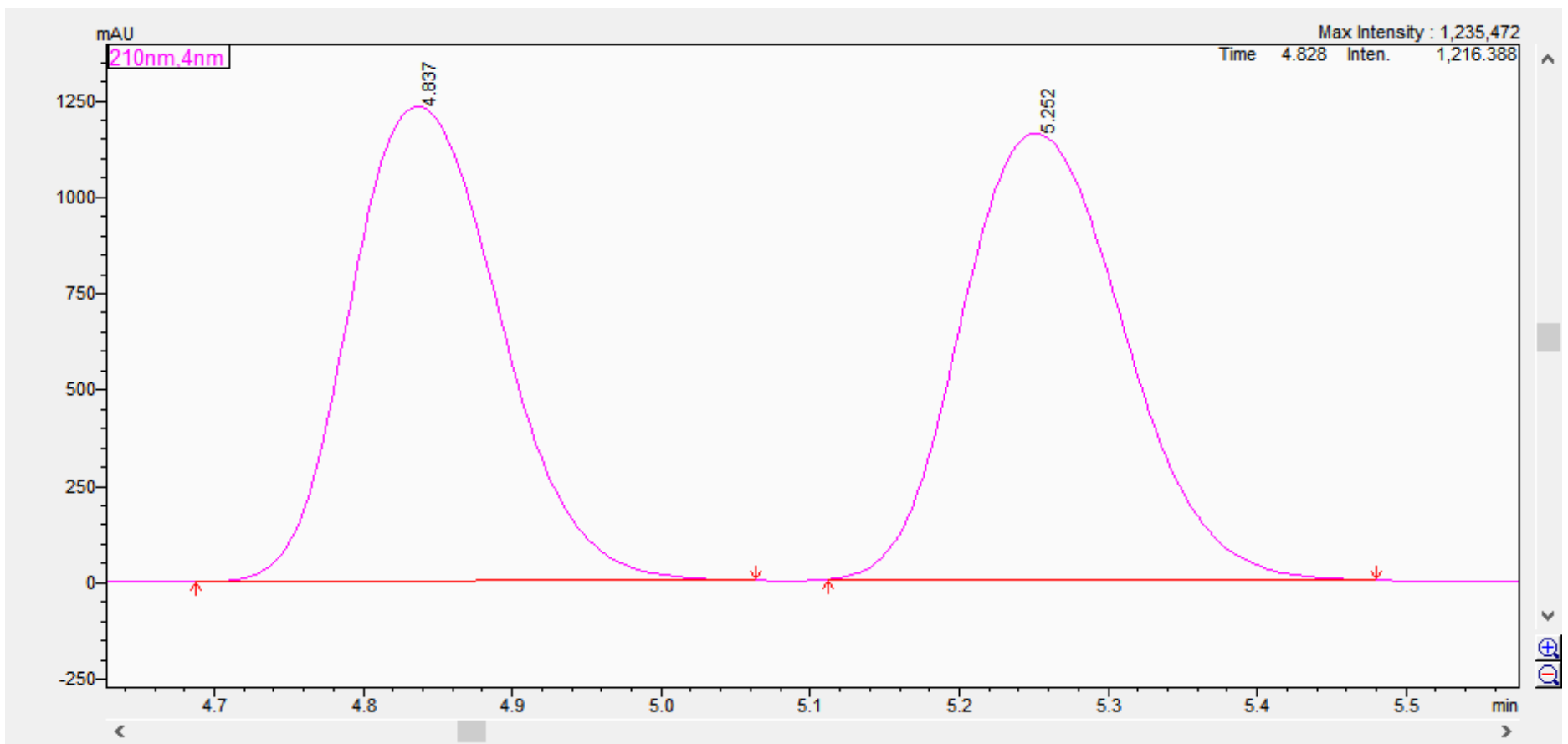

Peak#	Ret. Time	Area	Area%
1	4.837	8626452	49.512
2	5.252	8796654	50.488
Total		17423107	100.000

Enantioenriched (*R*)-**3p** (HPLC analysis):

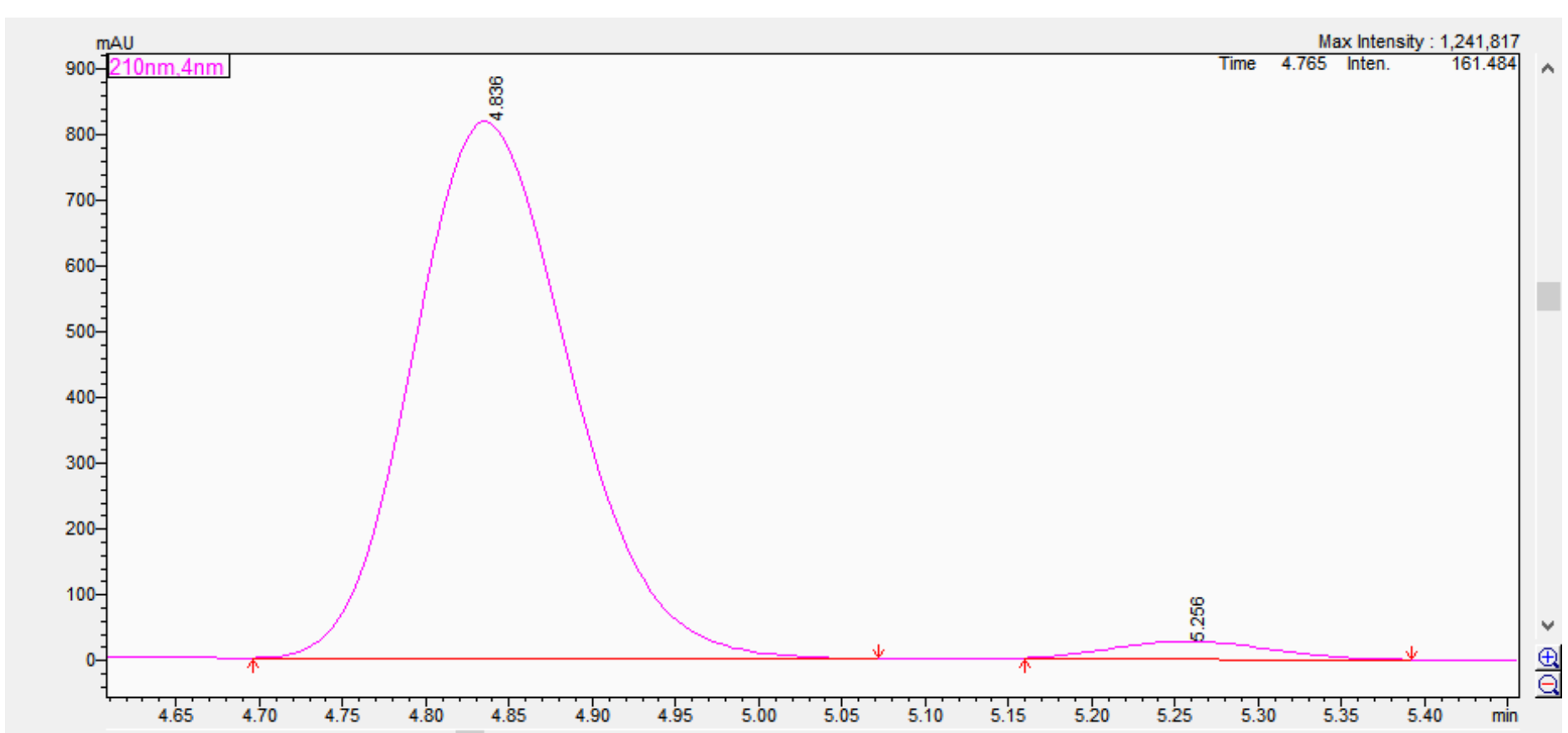

Peak#	Ret. Time	Area	Area%
1	4.836	5374711	96.935
2	5.256	169942	3.065
Total		5544653	100.000

(*R*)-2-bromo-6-((7-fluoro-1,2,3,4-tetrahydronaphthalen-1-yl)methyl)pyridine (3q)

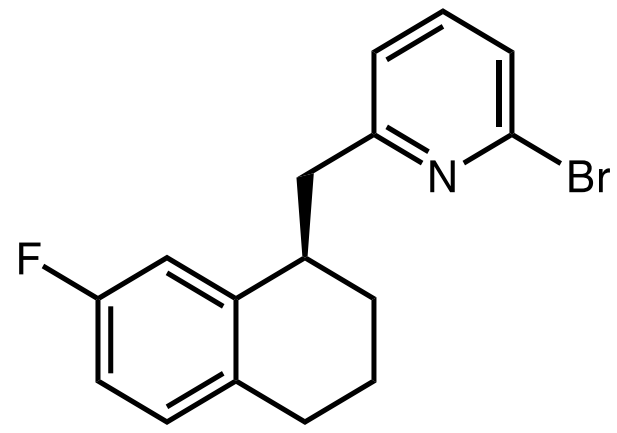

Racemic **3q** (HPLC analysis):

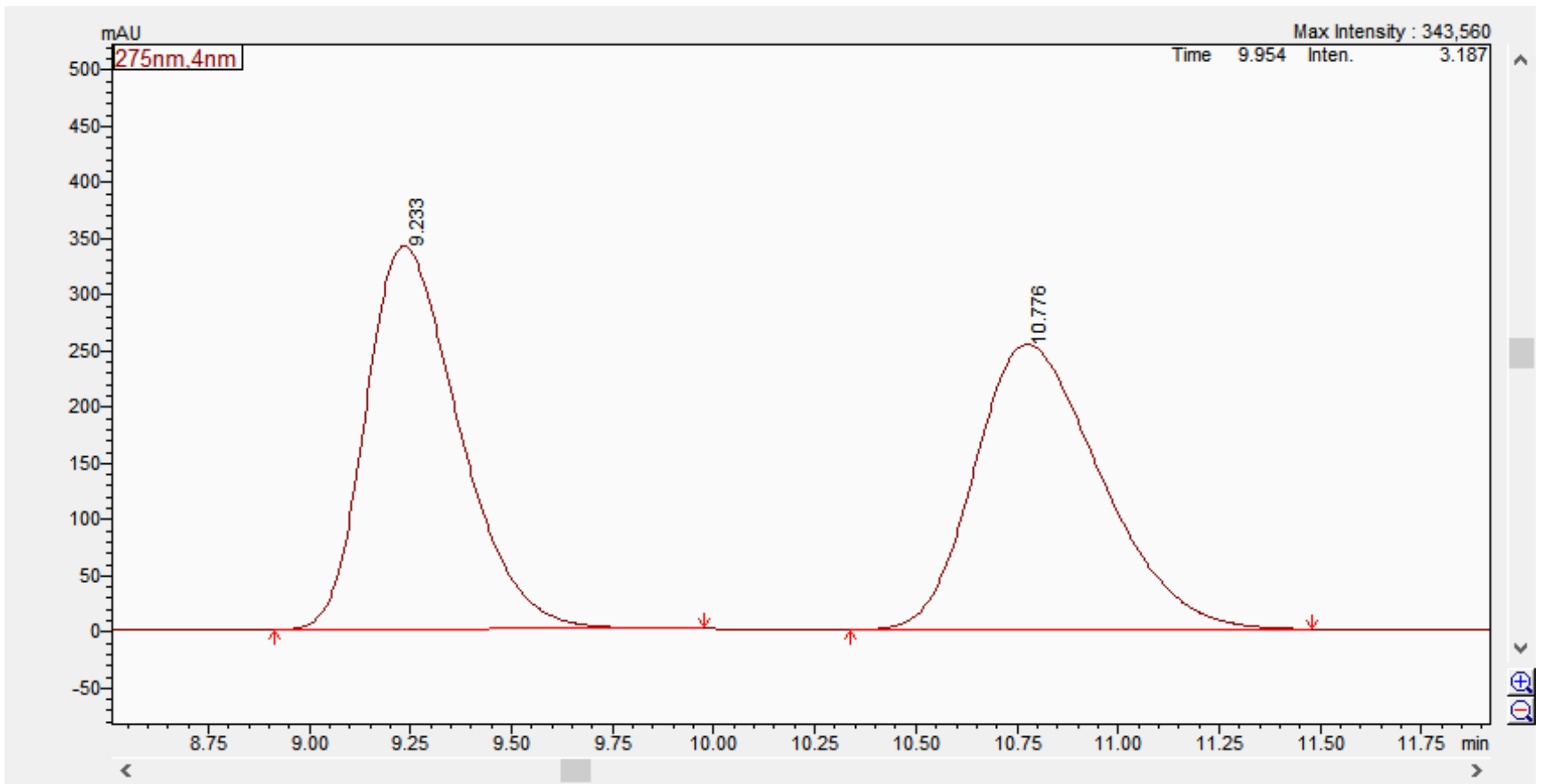

Peak#	Ret. Time	Area	Area%
1	9.233	5486989	49.904
2	10.776	5508017	50.096
Total		10995006	100.000

Enantioenriched (*R*)-**3q** (HPLC analysis):

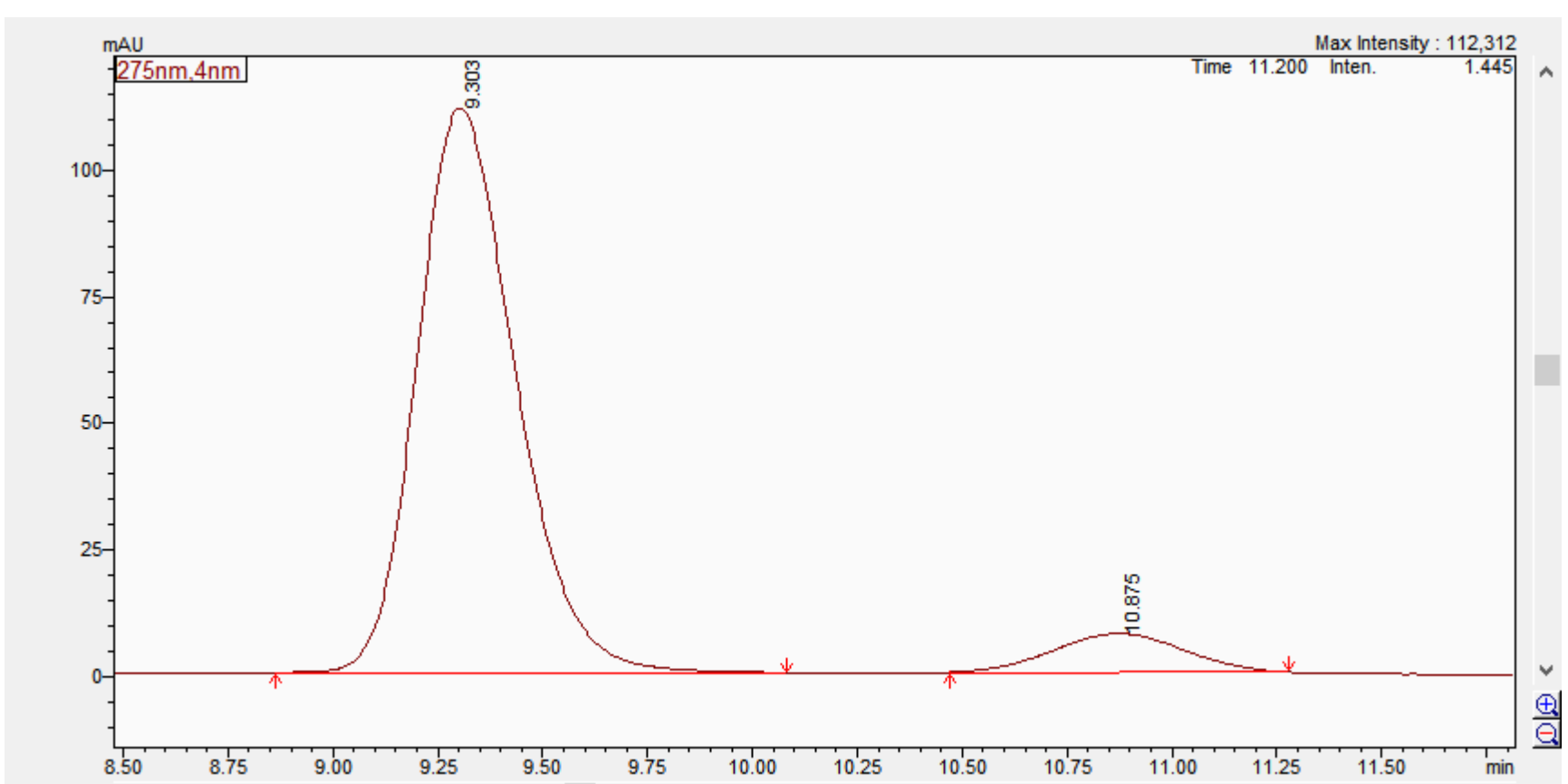

Peak#	Ret. Time	Area	Area%
1	9.303	1821137	91.668
2	10.875	165519	8.332
Total		1986656	100.000

(*S*)-2-bromo-6-(chroman-4-ylmethyl)pyridine (3r)

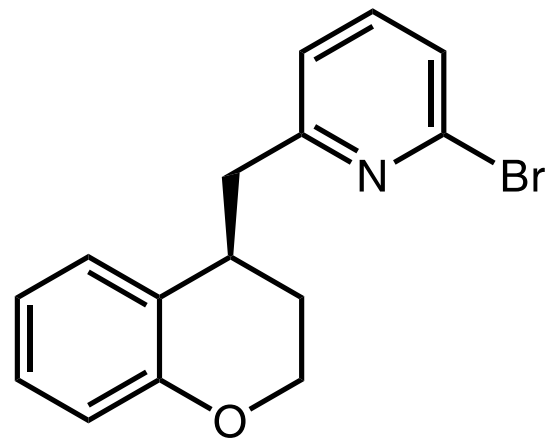

Racemic **3r** (HPLC analysis):

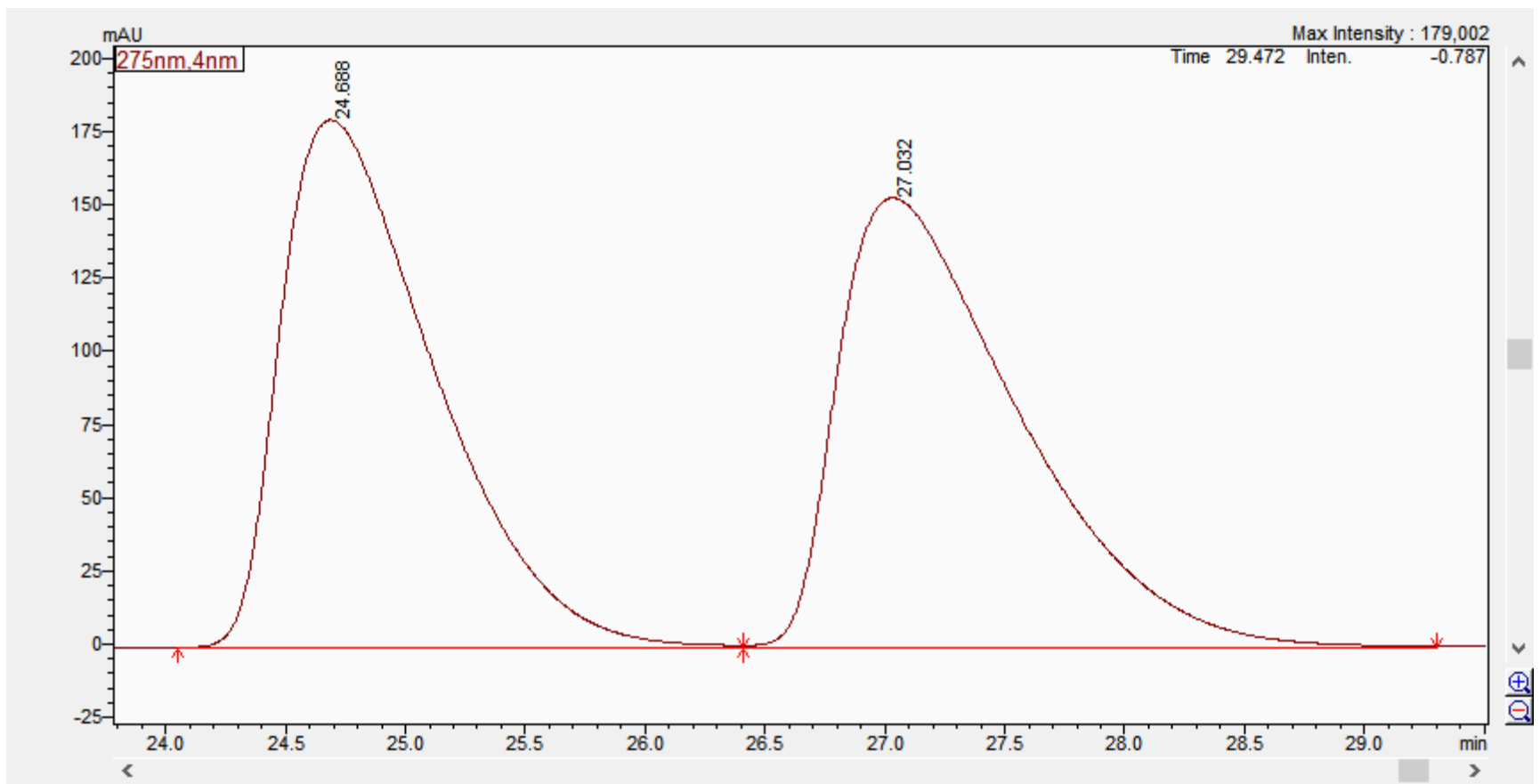

Peak#	Ret. Time	Area	Area%
1	24.688	8033308	49.925
2	27.032	8057318	50.075
Total		16090626	100.000

Enantioenriched (*S*)-**3r** (HPLC analysis):

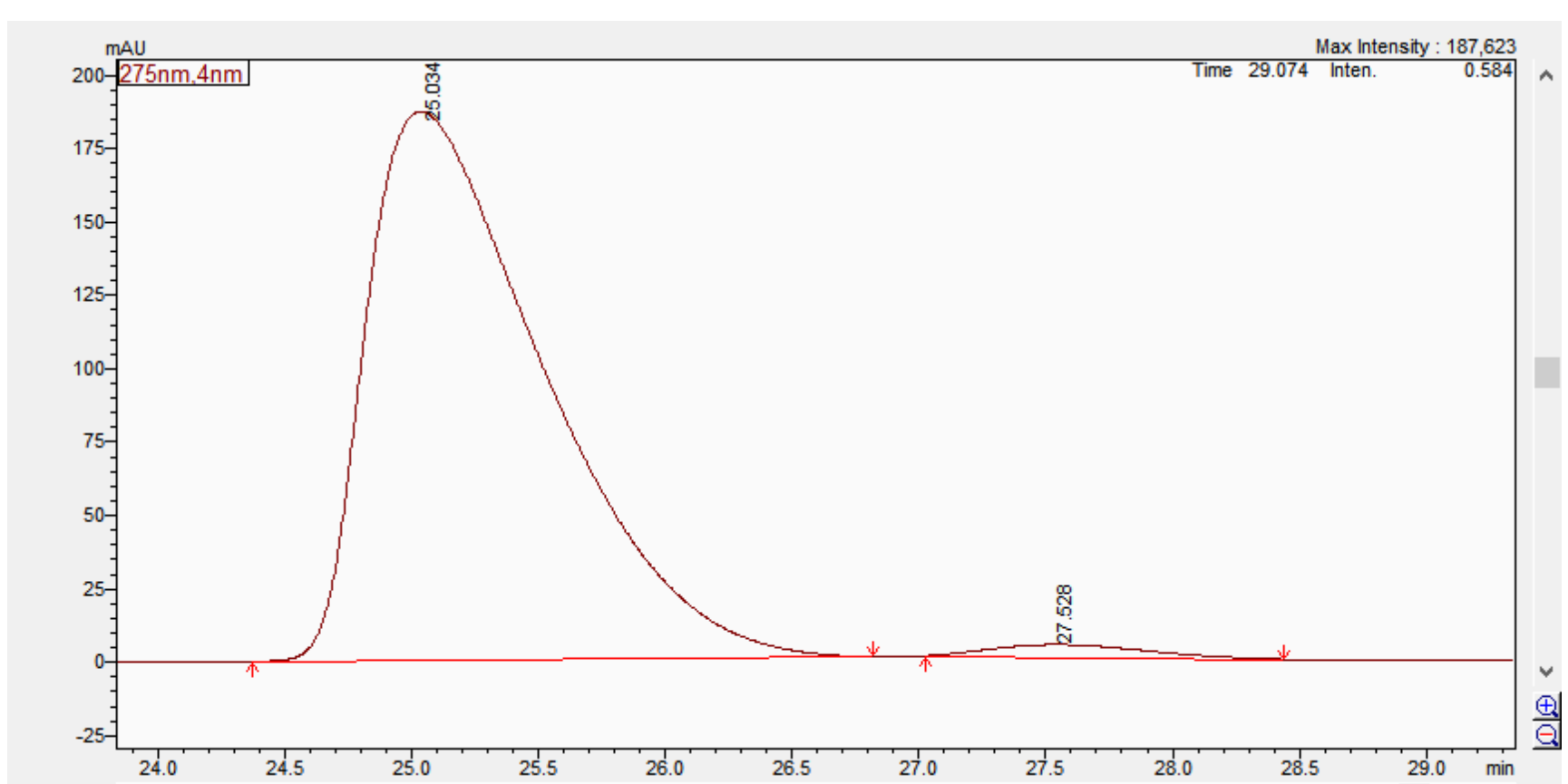

Peak#	Ret. Time	Area	Area%
1	25.034	9114954	98.058
2	27.528	180515	1.942
Total		9295469	100.000

(*R*)-2-chloro-6-(2-(p-tolyl)propyl)pyridine (3s)

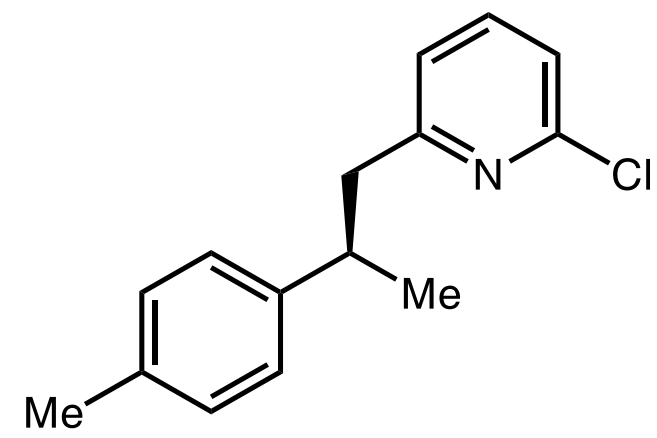

Racemic **3s** (HPLC analysis):

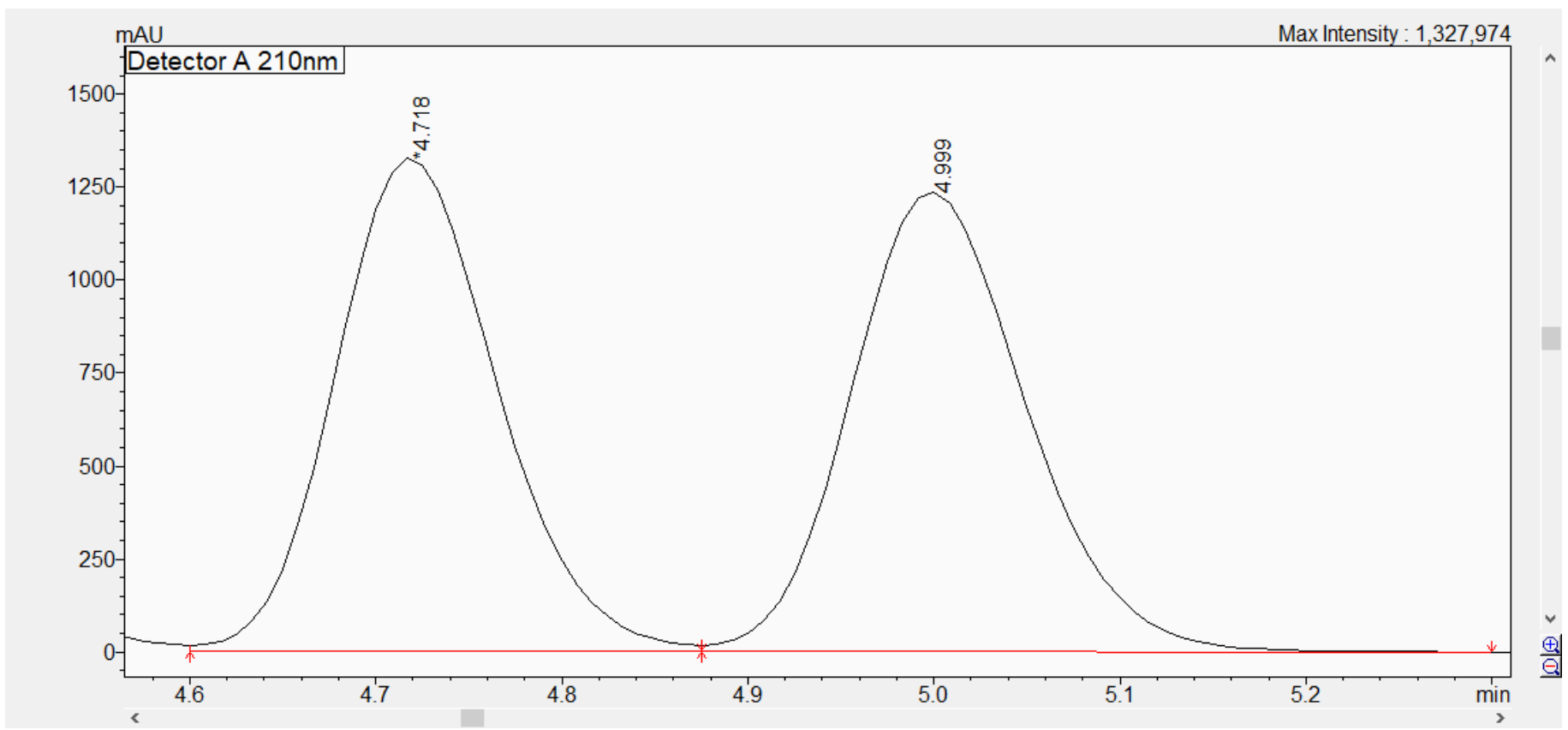

Peak#	Height	Ret. Time	Area	Area%
1	1326630	4.718	8050932	49.863
2	1234537	4.999	8095041	50.137
Total	2561167		16145974	100.000

Enantioenriched (*R*)-**3s** (HPLC analysis):

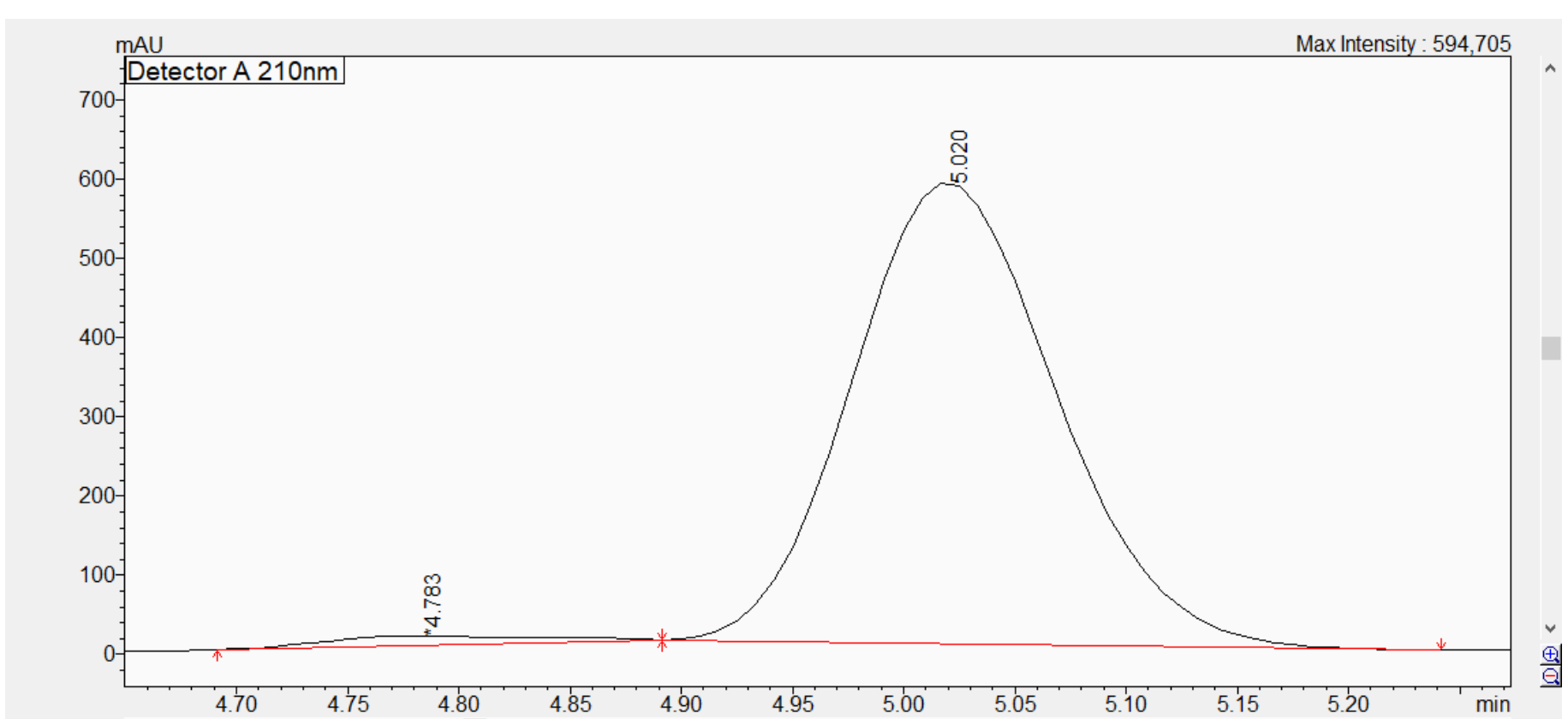

Peak#	Height	Ret. Time	Area	Area%
1	11735	4.783	80472	2.119
2	580898	5.020	3716654	97.881
Total	592632		3797126	100.000

(*R*)-2-Iodo-6-(2-(p-tolyl)propyl)pyridine (3t)

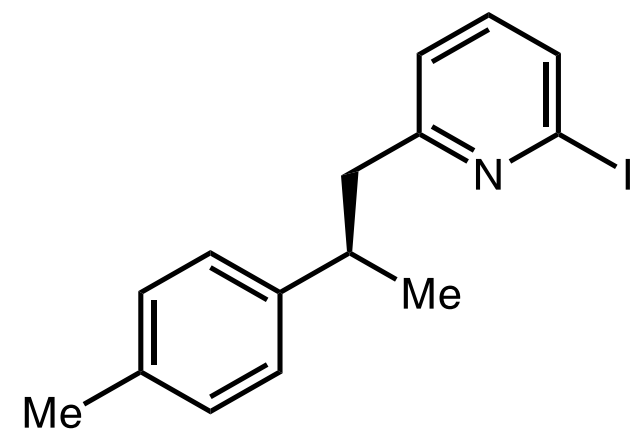

Racemic **3t** (HPLC analysis):

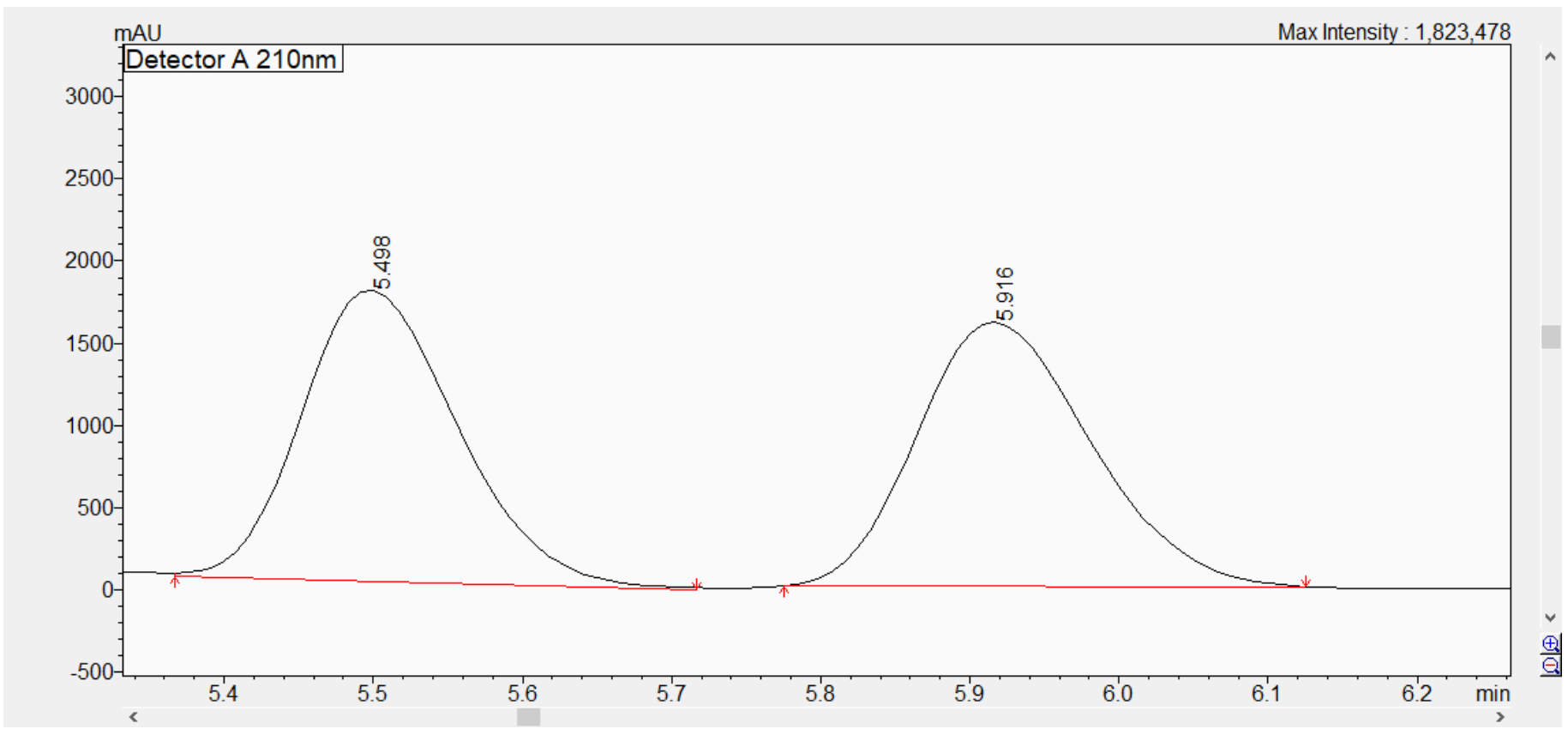

Peak#	Height	Ret. Time	Area	Area%
1	1774266	5.498	12908333	49.701
2	1611139	5.916	13063857	50.299
Total	3385405		25972191	100.000

Enantioenriched (*R*)-**3t** (HPLC analysis):

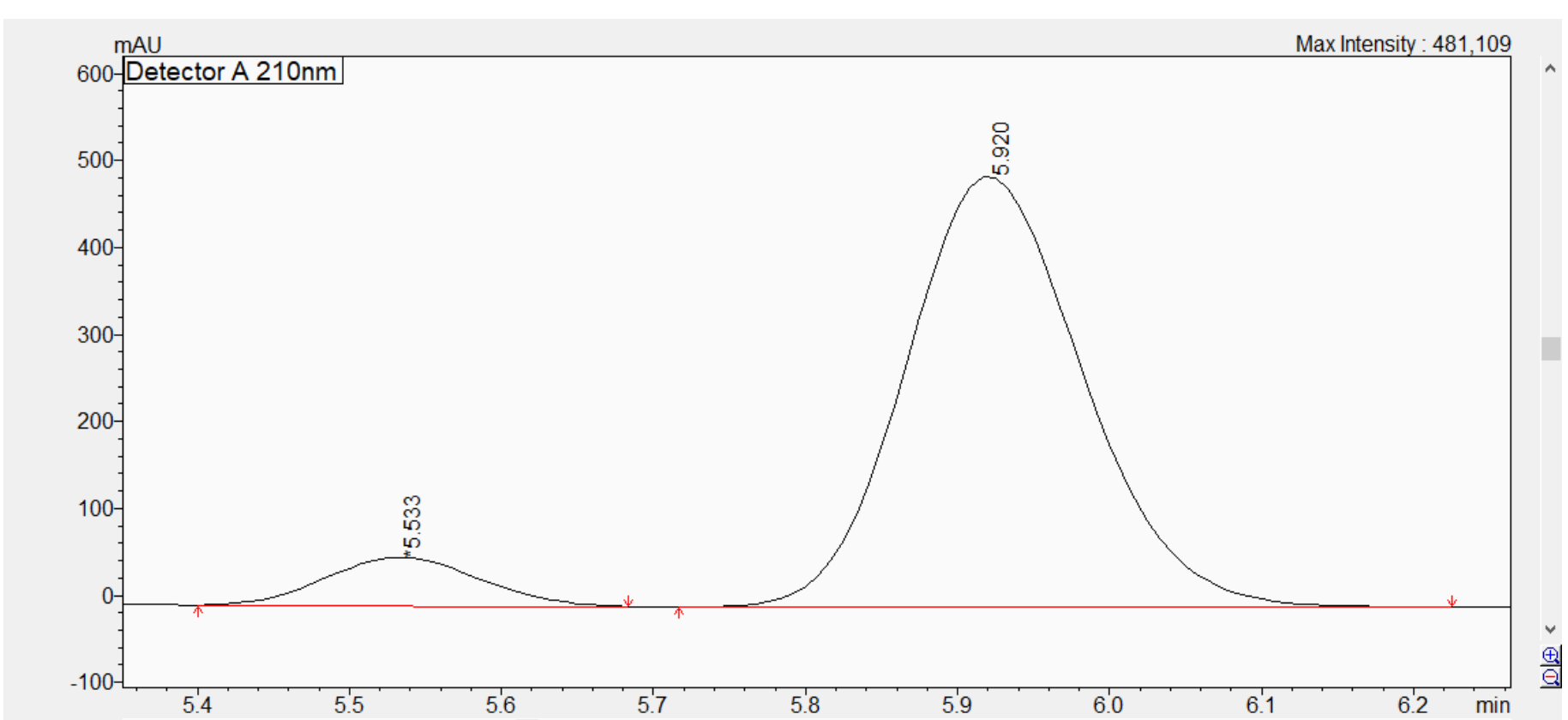

Peak#	Height	Ret. Time	Area	Area%
1	55471	5.533	389106	8.825
2	494558	5.920	4019857	91.175
Total	550029		4408963	100.000

(*R*)-2-methoxy-6-(2-(*p*-tolyl)propyl)pyridine (3u)

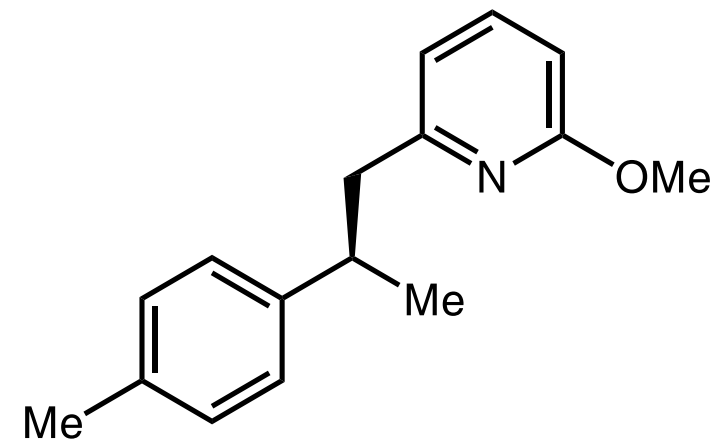

Racemic **3u** (HPLC analysis):

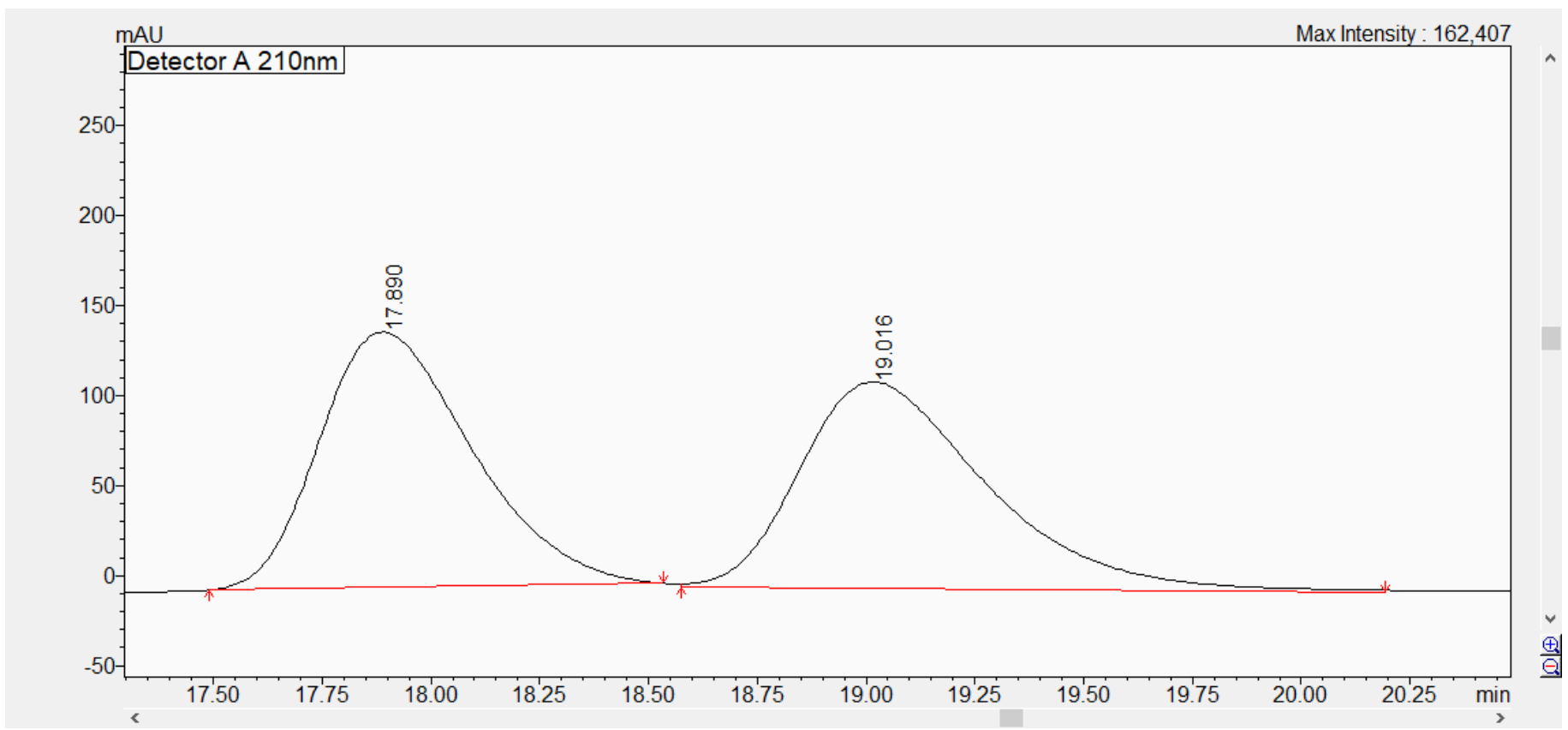

Peak#	Height	Ret. Time	Area	Area%
1	141962	17.890	3464419	50.115
2	114761	19.016	3448531	49.885
Total	256723		6912950	100.000

Enantioenriched (*R*)-**3u** (HPLC analysis):

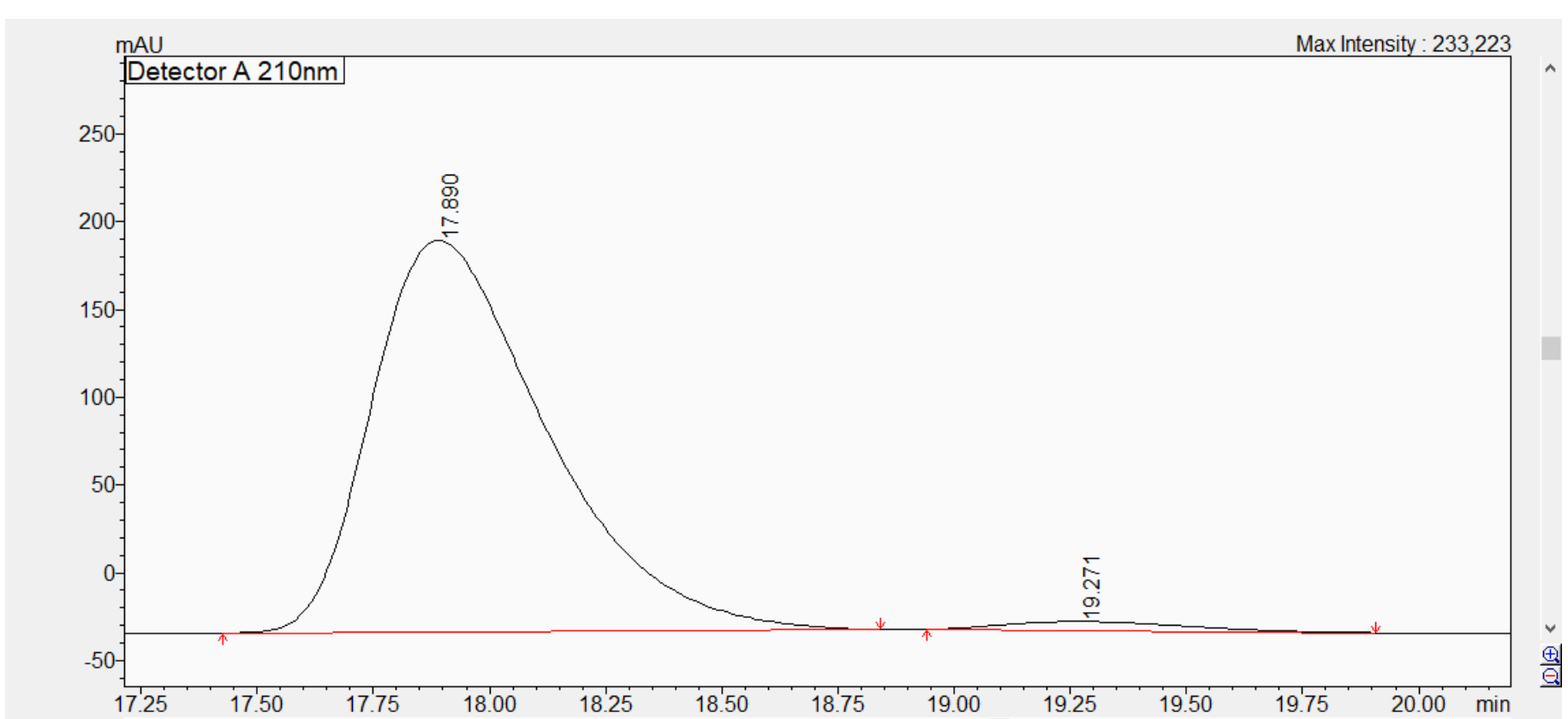

Peak#	Height	Ret. Time	Area	Area%
1	223069	17.890	5846140	97.693
2	5143	19.271	138074	2.307
Total	228213		5984214	100.000

(*R*)-2-(methylthio)-4-(2-(*p*-tolyl)propyl)pyrimidine (3v)

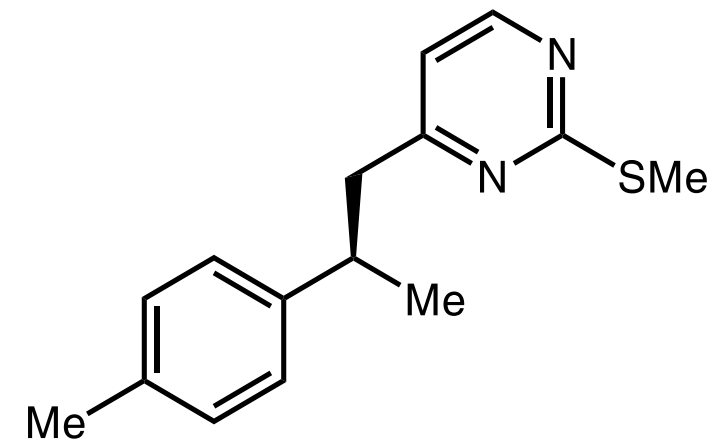

Racemic **3v** (HPLC analysis):

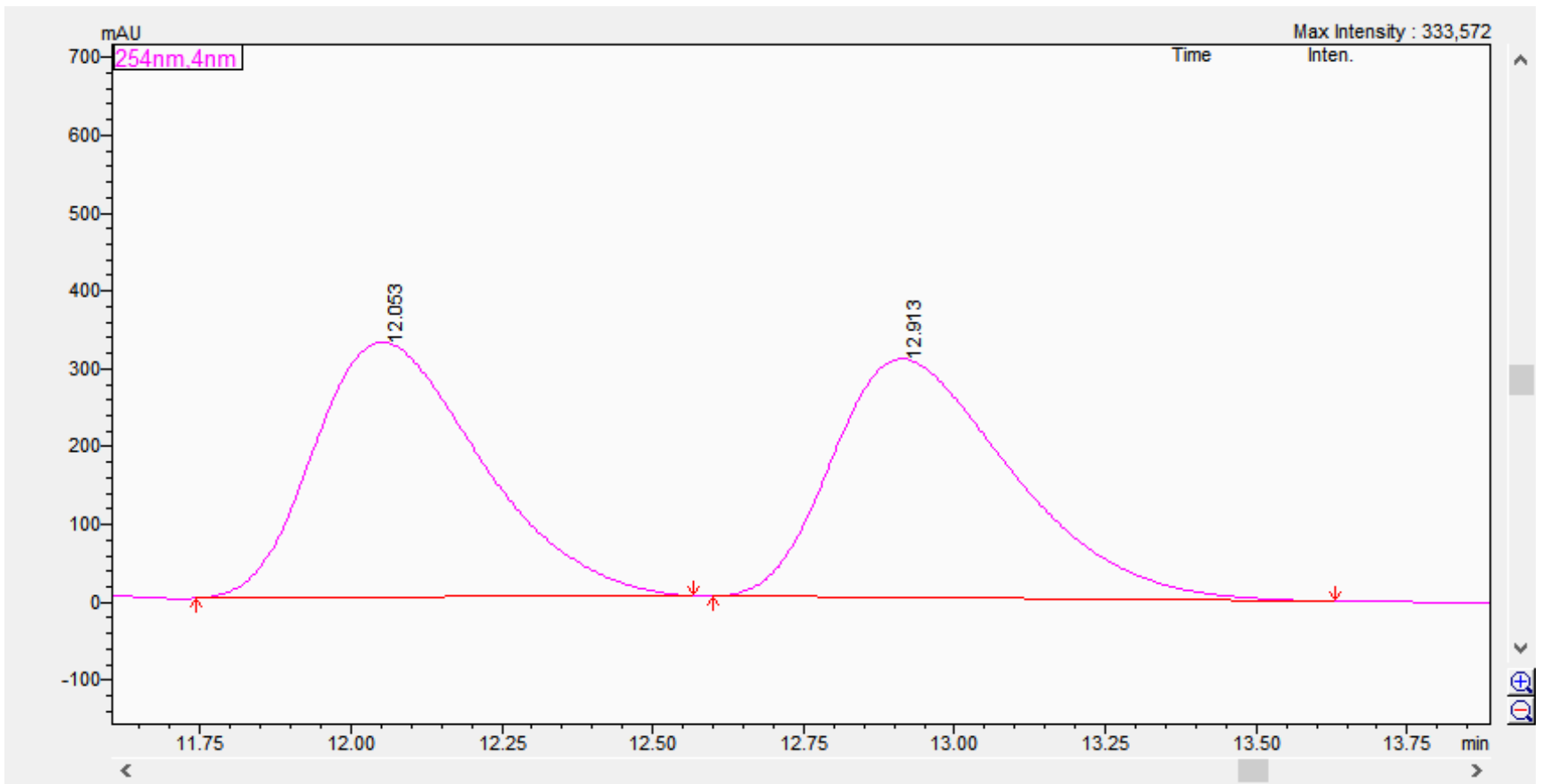

Peak#	Ret. Time	Area	Area%
1	12.053	6313386	49.787
2	12.913	6367334	50.213
Total		12680719	100.000

Enantioenriched (*R*)-**3v** (HPLC analysis):

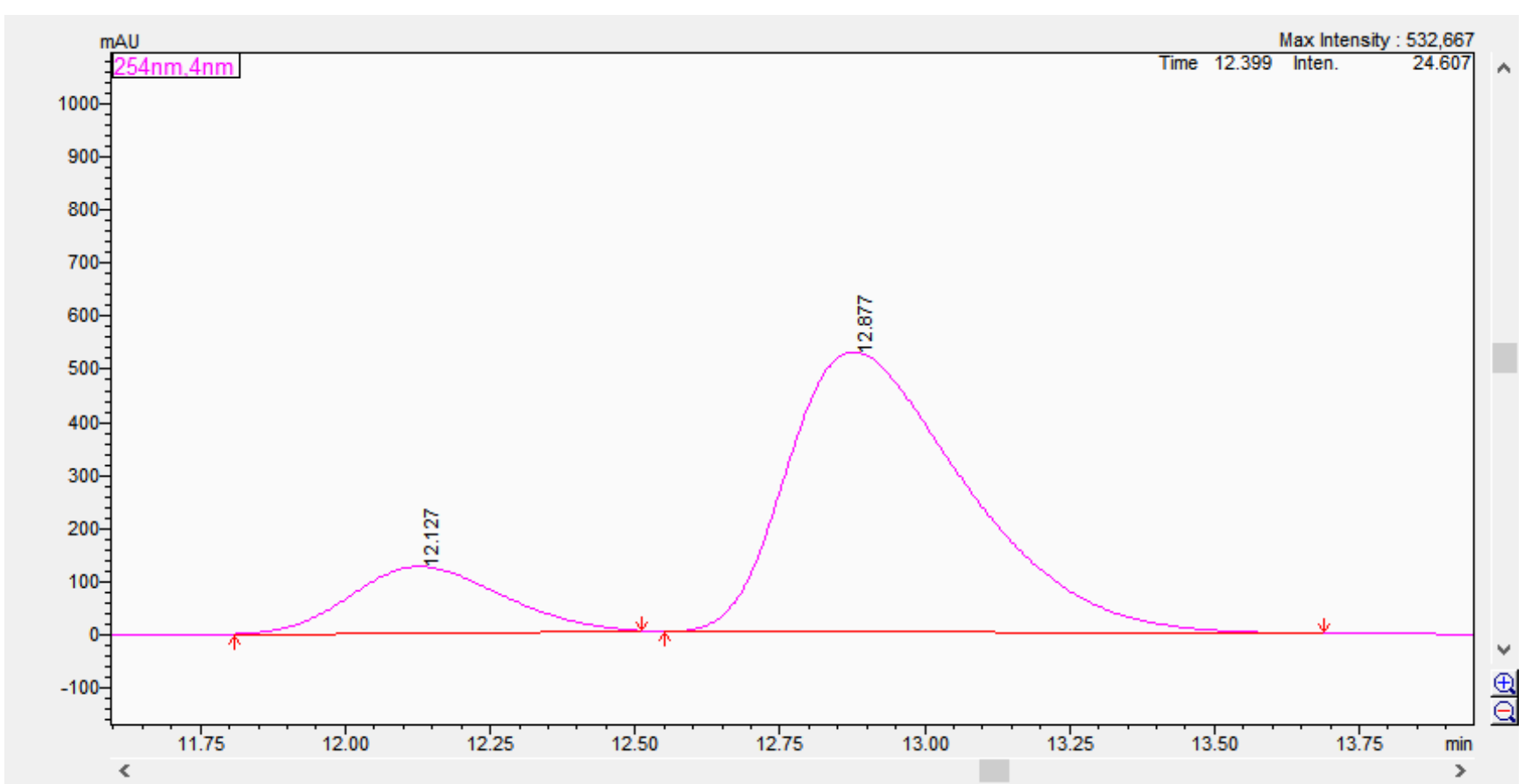

Peak#	Ret. Time	Area	Area%
1	12.127	2245538	16.588
2	12.877	11291601	83.412
Total		13537139	100.000

(*R*)-2-methoxy-6-(2-phenylpropyl)pyridine (3w)

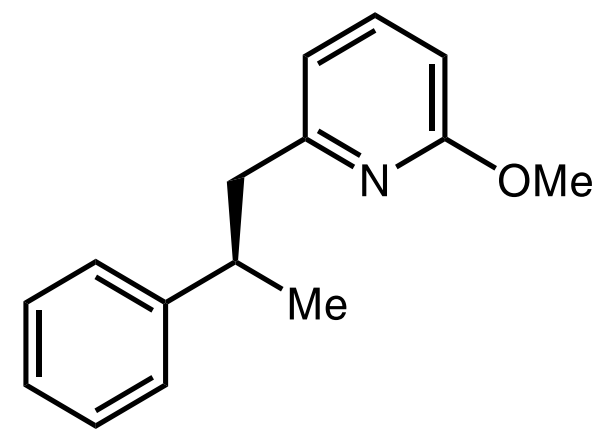

Racemic **3w** (HPLC analysis):

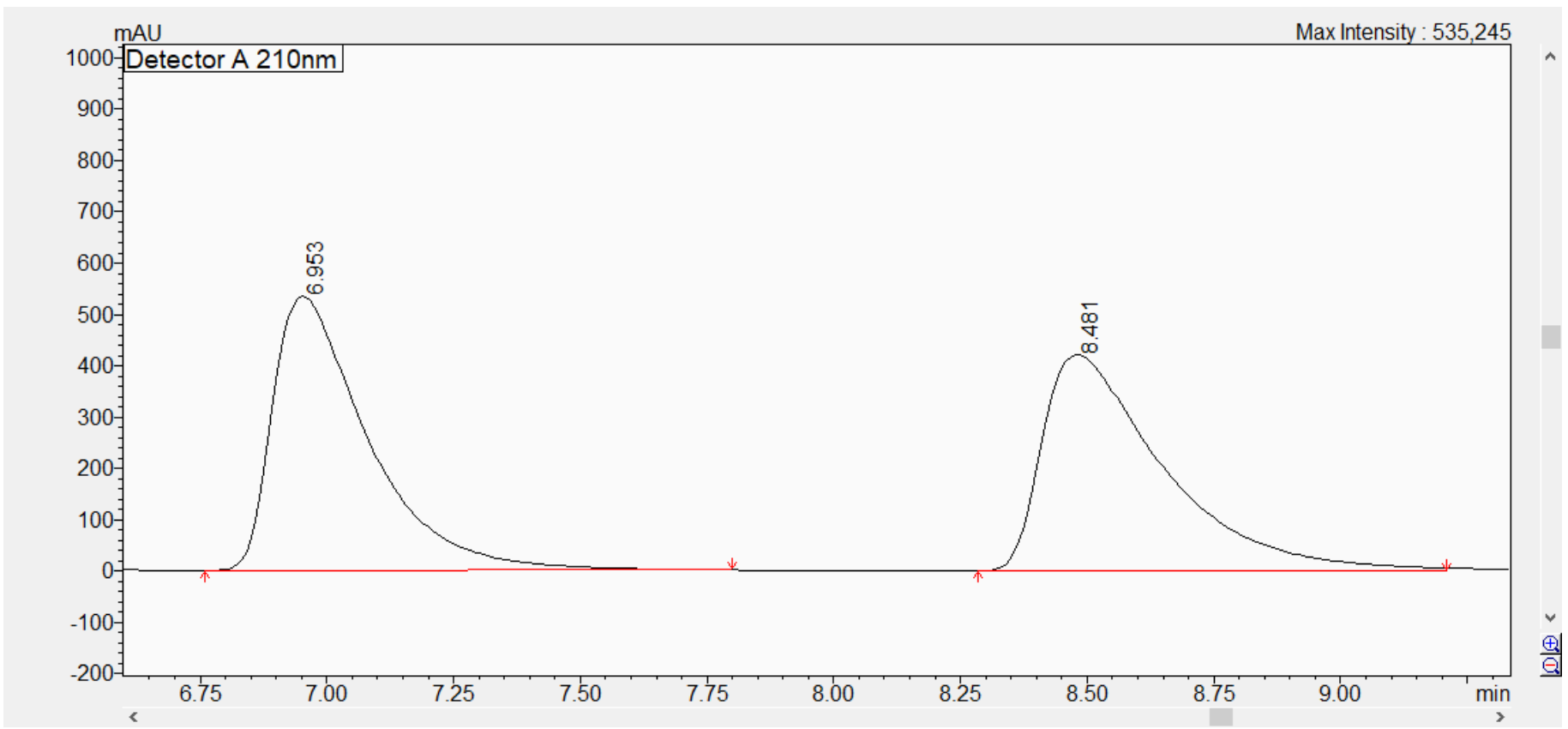

Peak#	Height	Ret. Time	Area	Area%
1	533346	6.953	7006275	49.923
2	421233	8.481	7027750	50.077
Total	954579		14034026	100.000

Enantioenriched (*R*)-**3w** (HPLC analysis):

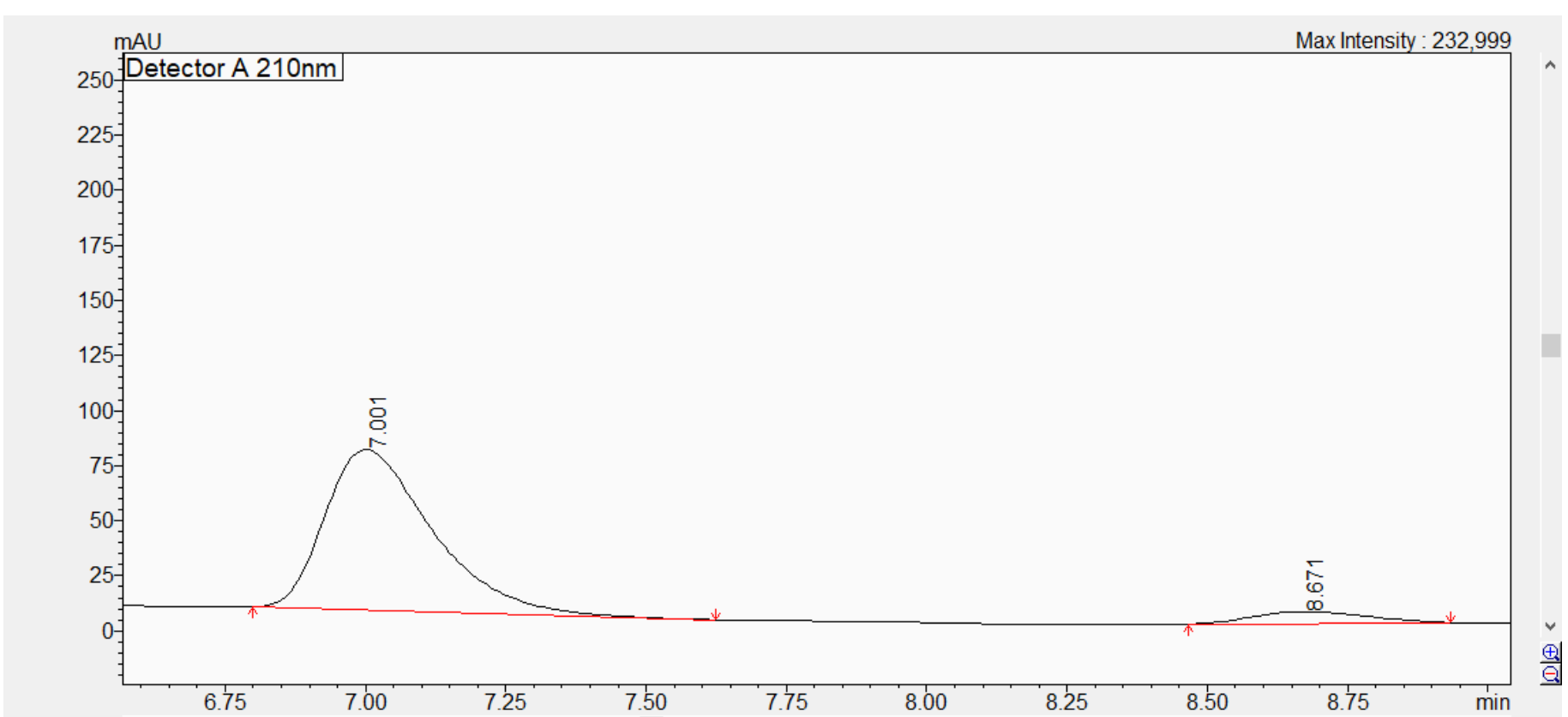

Peak#	Height	Ret. Time	Area	Area%
1	72945	7.001	971138	92.941
2	5373	8.671	73763	7.059
Total	78318		1044902	100.000

X. Computational Studies

Density functional theory calculations

Density functional theory (DFT) calculations were performed to investigate the enantioselectivity on the S_H2 radical substitution step using a truncated active site model, in which porphine and imidazole were used as a model for the porphyrin ligand and the axial histidine ligand, respectively.[11] DFT calculations were carried out using the Gaussian 16 software.[12] Geometries of intermediates and transition states were optimized using the dispersion-corrected[13] B3LYP-D3(BJ) functional[14] with the SDD basis set[15] for Fe atom and 6-31G(d) basis set for other atoms. Vibrational frequency calculations were performed at the same level of theory as their respective geometry optimizations to confirm that the obtained transition states connect their respective minima. Quasi-harmonic approximations with the Cramer and Truhlar approach[16] were performed using the GoodVibes package[17], in which all vibrational frequencies below 100 cm^{-1} were shifted to 100 cm^{-1} in entropy calculations. Single-point energy calculations were carried out using the B3LYP-D3(BJ) functional with the def2-TZVP basis set[18]. Solvation energy corrections were calculated in the single-point energy calculations using the SMD solvation model[19]. Diethyl ether solvent ($\varepsilon = 4.3$) was used to mimic the actual dielectric constant within enzyme active site[20]. Only the ground spin states were considered in the DFT calculations[21], namely the doublet state for the Fe–porphyrin alkyl intermediate (IPA) and the triplet state for the S_H2 radical substitution transition states.

Classical molecular dynamics simulations

Classical molecular dynamics (MD) simulations were performed to investigate the preferred substrate binding pose and active site residue–radical intermediate interactions with the secondary benzylic radical in their near-attack conformations[22] (NACs) prior to the radical substitution transition states. The starting structures of *Ape*Pgb W59A Y60G and *Ape*Pgb^{LGGTS} variants were generated using the AlphaFold 3 Server[23].

Classical MD simulations were carried out using the pmemd module[24] of the GPU-accelerated Amber 24 software[25]. The Amber ff19SB force field[26] was used in all classical MD simulations. The general Amber force field (gaff2)[27] was used for the benzylic radical. Force field parameters for the IPA intermediate were generated using the MCPB.py module[28]. Using the Merz-Singh-

Kollman scheme[29], RESP charge fitting[30] on electrostatic potential generated at the B3LYP/6-31G(d) level of theory was performed to generate partial charges for IPA intermediate at the DFT-computed ground states (i.e., doublet spin states) and the benzylic radical. Protonation states of enzyme residues were determined using the H++ server[31]. The enzyme was then put into a solvated cuboid box with periodic boundary condition using the OPC3 water model[32]. The minimum distance between the enzyme surface and the edge of the water box was set to 10 Å. Water molecules were treated with the SHAKE algorithm[33]. The system was neutralized by adding Na^+ counterions. Long-range electrostatic was calculated using the particle-mesh-Ewald method[34]. Lennard-Jones and electrostatic interaction cut-offs were set to 12 Å.

We first performed a 30,000-step energy minimization with positional restraints for the protein and the substrate by applying a force constant of 500 $kcal\cdot mol^{-1}\cdot Å^{-2}$. Next, the system was gradually heated from 0 K to 300 K in 200 ps, which was followed by an equilibration using the isothermal–isobaric ensemble (NPT) in the next 50 ns. Finally, 1,000 ns production MD simulations were performed using the same conditions as the equilibration with a time step of 2 fs. In the NAC simulations, the distance between the benzyl radical's benzylic carbon atom and the Cα atom of Fe-alkyl intermediate was restrained in a range of 2.7–2.9 Å with a harmonic potential of 200 $kcal\cdot mol^{-1}\cdot Å^{-2}$. After the MD simulations, clustering analysis was carried out using the cpptraj module[35] to identify the most populated structure from the classical MD simulations. The RMSD value was used as the distance metric in the clustering analysis.

DFT-optimized S_H2 radical substitution transition states using a truncated model

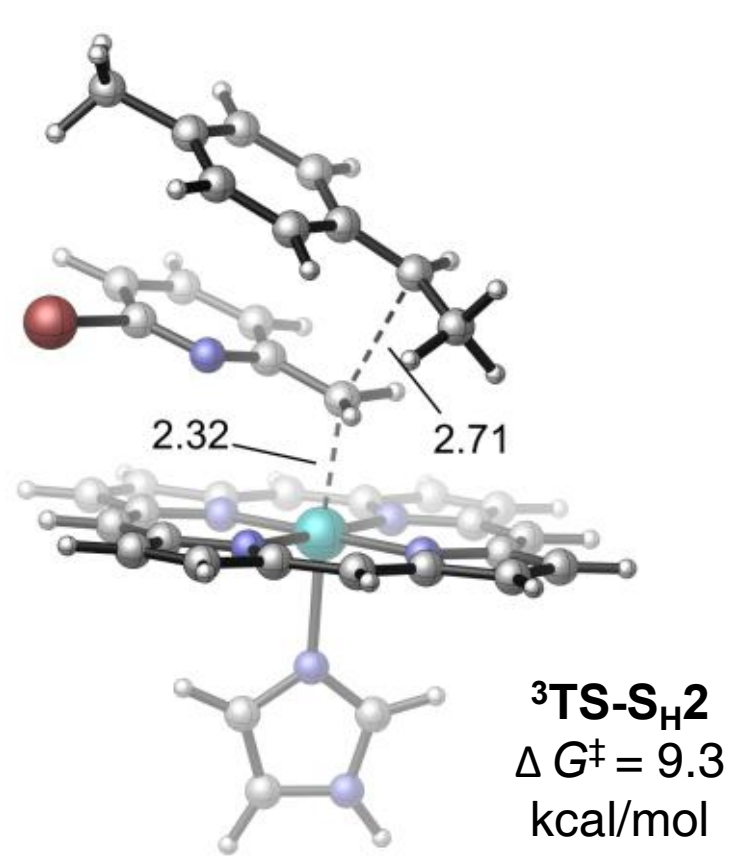

Figure S14. Optimized S_H2 radical substitution transition states at triplet spin state. The Gibbs free energies are relative to the separated Fe-alkyl intermediate and the secondary benzylic radical. Bond distances are in Å.

Classical MD simulations of Fe-alkyl intermediates

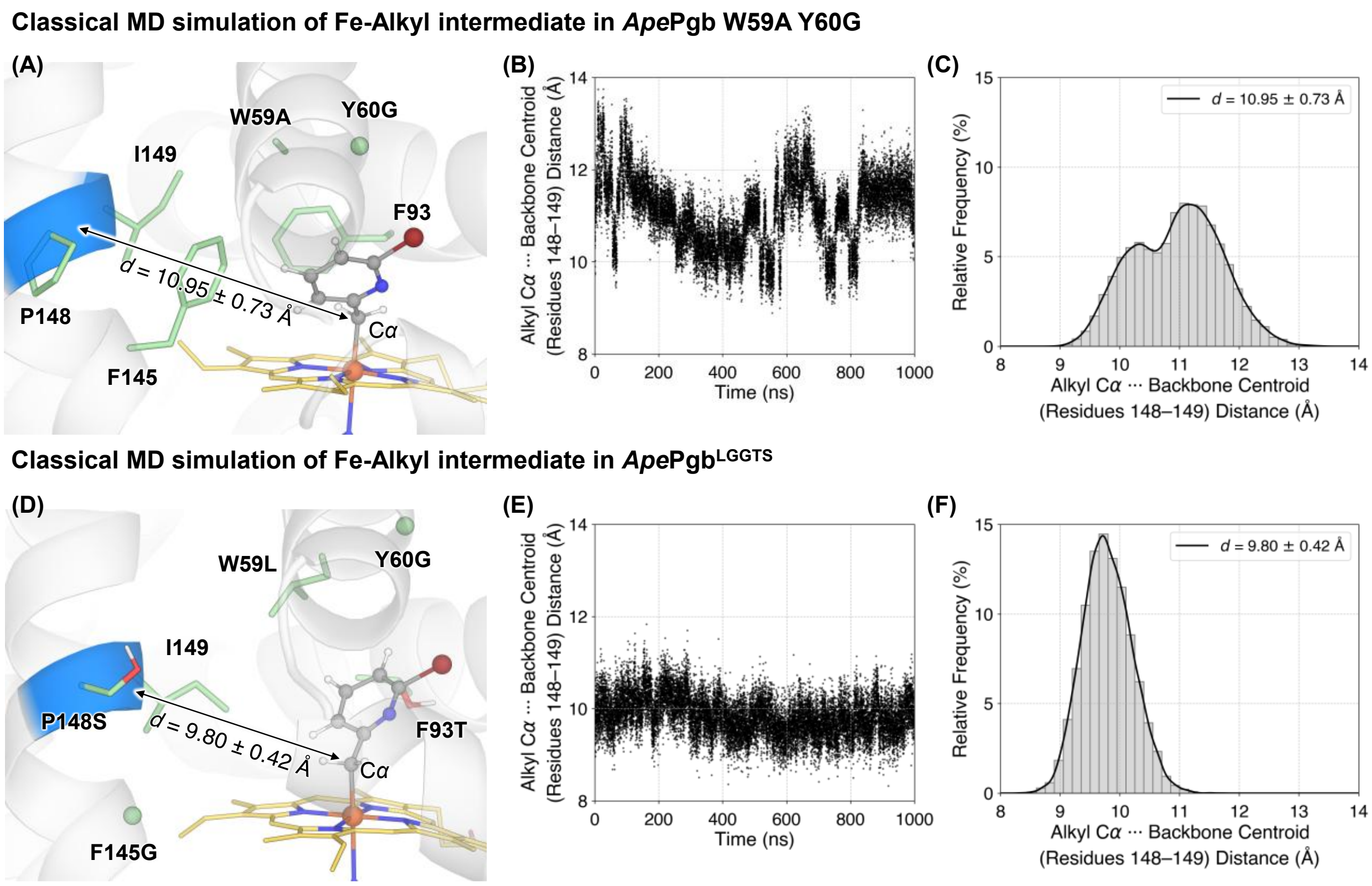

Figure S15. Classical MD simulations of Fe-alkyl intermediates in *Ape*Pgb W59A Y60G and *Ape*Pgb^{LGGTS}. (A) The most populated structure from clustering analysis of the MD trajectory of *Ape*Pgb W59A Y60G. (**B)** Time evolution of the alkyl's Cα···Helix backbone 148–149 distance in MD simulations of *Ape*Pgb W59A Y60G. (**C)** Distribution of the alkyl's Cα···Helix backbone 148–149 distance in the last 800 ns of the MD simulations of *Ape*Pgb W59A Y60G. **(D)** The most populated structure from clustering analysis of the MD trajectory of *Ape*Pgb^{LGGTS}. (**E)** Time evolution of the alkyl's Cα···Helix backbone 148–149 distance in MD simulations of *Ape*Pgb^{LGGTS}. (**F)** Distribution of the alkyl's Cα···Helix backbone 148–149 distance in the last 800 ns of the MD simulations of *Ape*Pgb^{LGGTS}.

Classical MD simulations of near-attack conformations (NACs) for the S_H2 substitution on the (*Si*)-face of the prochiral secondary alkyl radical

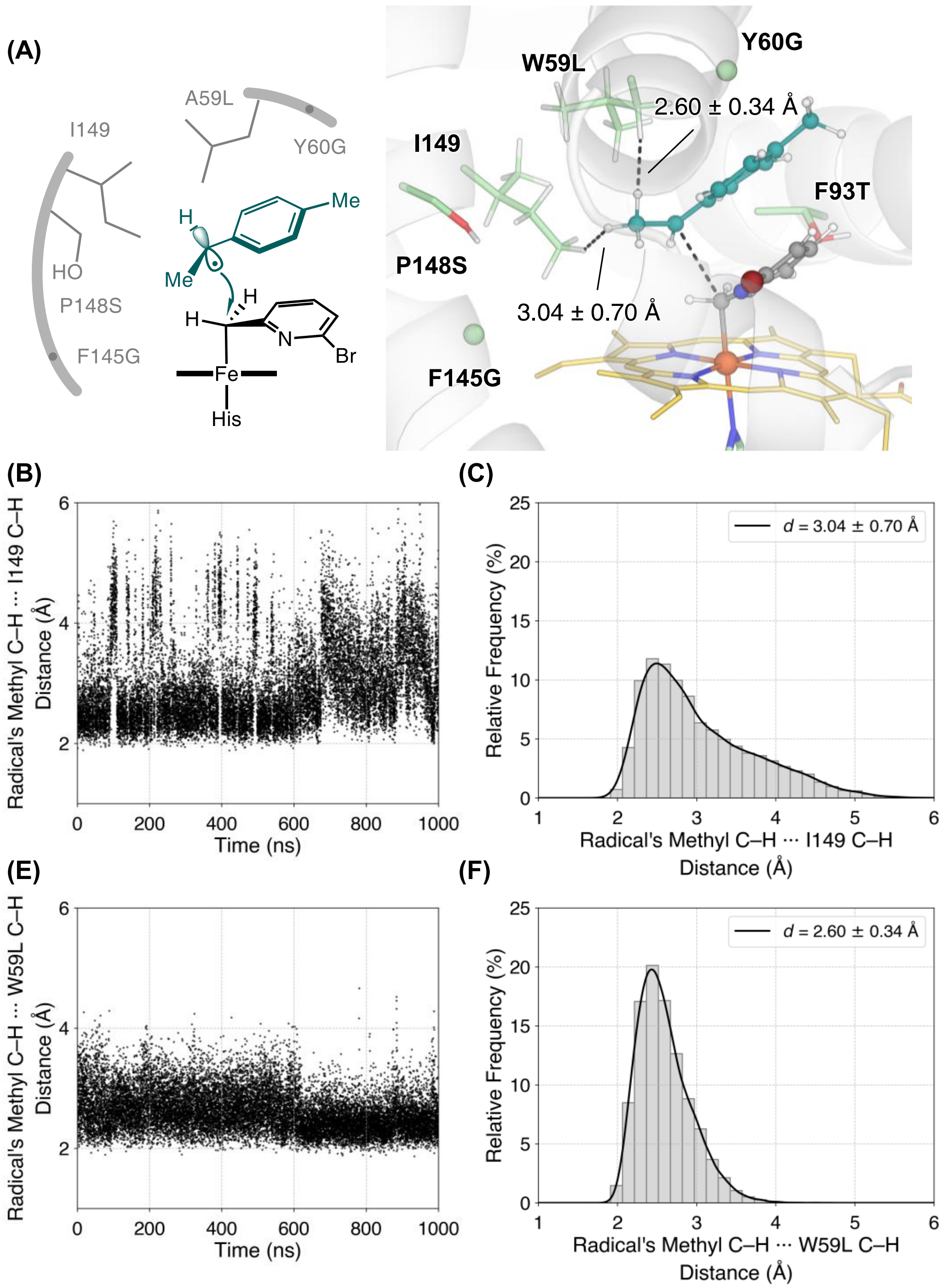

Figure S16. Classical MD simulations of the S_H2 substitution on the (*Si*)-face of the prochiral secondary alkyl radical in *Ape*Pgb^{LGGTS}. (A) The most populated structure from clustering

analysis of the NAC MD trajectory. (**B)** Time evolution of the radical's methyl C–H···I149 C–H distance in MD simulations. (**C)** Distribution of the radical's methyl C–H···I149 C–H distance in the last 800 ns of the MD simulations. (**E)** Time evolution of the radical's methyl C–H···W59L C–H distance in MD simulations. (**F)** Distribution of the radical's methyl C–H··· W59L C–H distance in the last 800 ns of the MD simulations.

Classical MD simulations of near-attack conformations (NACs) for the S_H2 substitution on the (*Re*)-face of the prochiral secondary alkyl radical

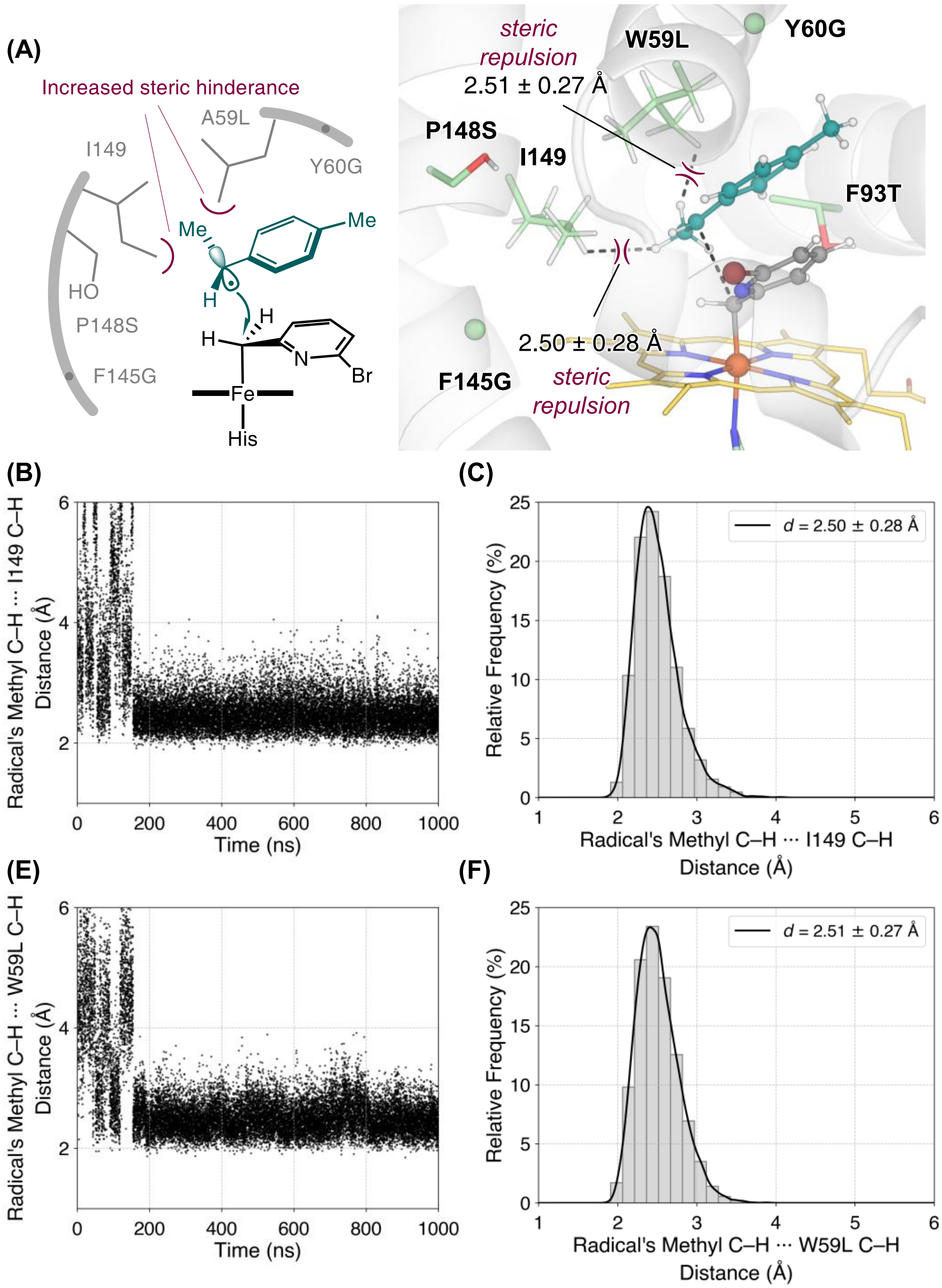

Figure S17. Classical MD simulations of the S_H2 substitution on the (*Re*)-face of the prochiral secondary alkyl radical in *Ape*Pgb^{LGGTS}. (A) The most populated structure from clustering

analysis of the NAC MD trajectory. (**B)** Time evolution of the radical's methyl C–H···I149 C–H distance in MD simulations. (**C)** Distribution of the radical's methyl C–H···I149 C–H distance in the last 800 ns of the MD simulations. (**E)** Time evolution of the radical's methyl C–H···W59L C–H distance in MD simulations. (**F)** Distribution of the radical's methyl C–H··· W59L C–H distance in the last 800 ns of the MD simulations.

Cartesian Coordinates and Energies of DFT-Computed Structures

^{2}Fe-alkyl

Charge: 0

Multiplicity: 2

B3LYP-D3(BJ)/6-31G(d)-SDD SCF energy (au):	-4196.55045373
B3LYP-D3(BJ)/6-31G(d)-SDD enthalpy (au):	-4196.07325573
B3LYP-D3(BJ)/6-31G(d)-SDD free energy (au):	-4196.16721973
B3LYP-D3(BJ)/def2-TZVP/SMD SCF energy (au):	-5339.76885529
B3LYP-D3(BJ)/def2-TZVP/SMD enthalpy (au):	-5339.29165729
B3LYP-D3(BJ)/def2-TZVP/SMD free energy (au):	-5339.38562129
B3LYP-D3(BJ)/def2-TZVP/SMD free energy (quasi-harmonic) (au):	-5339.37813356

Cartesian coordinates

ATOM	X	Y	Z
Fe	-0.3619610000	-0.2281900000	-0.1726940000
N	1.4243110000	0.3623250000	0.5441150000
N	-0.0480460000	-2.0471360000	0.6421240000
N	-0.7271840000	1.6229020000	-0.8975560000
N	-2.1745450000	-0.8063850000	-0.8325770000
C	1.9850380000	1.6054820000	0.3824830000
C	-0.8854450000	-3.1290300000	0.5494760000
C	2.3403940000	-0.3843720000	1.2440350000
C	1.0659630000	-2.4865200000	1.3169490000
C	0.1326310000	2.6914450000	-0.8436250000
C	-2.7111340000	-2.0646300000	-0.7208120000
C	-1.8320760000	2.0543650000	-1.5886650000
C	-3.0896860000	-0.0623360000	-1.5392780000
C	3.2942110000	1.6407500000	0.9916240000
C	-0.2877650000	-4.2810170000	1.1865350000
C	3.5132810000	0.4065010000	1.5284050000
C	0.9226460000	-3.8821410000	1.6638960000
C	-0.4545750000	3.8348500000	-1.5046160000
C	-4.0013250000	-2.1142400000	-1.3679710000
C	-1.6746110000	3.4401890000	-1.9656850000
C	-4.2379930000	-0.8713970000	-1.8734190000
H	3.9453930000	2.5055600000	1.0015540000
H	-0.7399050000	-5.2627340000	1.2277820000
H	4.3811990000	0.0492040000	2.0677430000
H	1.6678460000	-4.4655600000	2.1888270000
H	0.0208570000	4.8029340000	-1.5985380000

H	-4.6272060000	-2.9956570000	-1.4226290000
H	-2.4067030000	4.0160080000	-2.5173940000
H	-5.0981470000	-0.5203340000	-2.4290490000
C	1.3910140000	2.6866070000	-0.2564610000
C	-2.1207520000	-3.1436200000	-0.0789300000
C	-2.9421220000	1.2738080000	-1.8867890000
C	2.1768030000	-1.7125060000	1.6147580000
H	1.9620490000	3.6089510000	-0.3014360000
H	-2.6623620000	-4.0837920000	-0.0861520000
H	-3.7468800000	1.7380660000	-2.4482990000
H	2.9911520000	-2.1877100000	2.1523300000
C	-2.3176260000	1.1535050000	1.7921010000
C	-0.8599020000	0.0480340000	2.9608910000
C	-1.7077790000	0.6340040000	3.8622480000
N	-1.2532370000	0.3811280000	1.6814540000
N	-2.6303010000	1.3335620000	3.1016230000
H	-1.7442850000	0.6222120000	4.9401380000
H	-0.0028540000	-0.5844210000	3.1295530000
H	-2.8668130000	1.5825450000	0.9682410000
H	-3.4013650000	1.8803350000	3.4550850000
C	0.4895990000	-0.6427100000	-1.9675570000
H	1.0652040000	0.2555060000	-2.1951870000
H	-0.3630120000	-0.7461830000	-2.6387890000
C	1.3222410000	-1.8512920000	-1.9387310000
C	2.7075860000	-1.8007300000	-1.6984310000
N	0.6967890000	-3.0445730000	-2.0996590000
C	3.4291630000	-2.9845180000	-1.5927300000
H	3.1929740000	-0.8380510000	-1.5793750000
C	1.4022460000	-4.1398200000	-1.9735730000
C	2.7718450000	-4.2083340000	-1.7186610000
H	4.4990490000	-2.9613540000	-1.4019930000
H	3.2822340000	-5.1587420000	-1.6288430000
Br	0.4319010000	-5.8088450000	-2.1388240000

^{3}TS-S$_{H}$2

Charge: 0

Multiplicity: 3

B3LYP-D3(BJ)/6-31G(d)-SDD SCF energy (au):	-4546.14947980
B3LYP-D3(BJ)/6-31G(d)-SDD enthalpy (au):	-4545.48992680
B3LYP-D3(BJ)/6-31G(d)-SDD free energy (au):	-4545.61000480

B3LYP-D3(BJ)/def2-TZVP/SMD SCF energy (au): -5689.50413179
B3LYP-D3(BJ)/def2-TZVP/SMD enthalpy (au): -5688.84457879
B3LYP-D3(BJ)/def2-TZVP/SMD free energy (au): -5688.96465679
B3LYP-D3(BJ)/def2-TZVP/SMD free energy (quasi-harmonic) (au): -5688.95144099

Cartesian coordinates

ATOM	X	Y	Z
Fe	0.0618020000	-0.0779000000	-0.0792690000
N	1.6079830000	0.6486750000	1.0048110000
N	0.4058490000	-1.9363810000	0.6337700000
N	-0.2838940000	1.7914340000	-0.7805230000
N	-1.4587650000	-0.8102480000	-1.1803370000
C	2.0571320000	1.9479650000	1.0598960000
C	-0.2665290000	-3.0924710000	0.3149240000
C	2.4263040000	-0.0750740000	1.8370700000
C	1.3917380000	-2.2990610000	1.5167460000
C	0.4236070000	2.9368340000	-0.5049140000
C	-1.8744420000	-2.1148930000	-1.2727950000
C	-1.2349160000	2.1394010000	-1.7065930000
C	-2.2349410000	-0.0988110000	-2.0599320000
C	3.1881480000	2.0454690000	1.9524980000
C	0.3109210000	-4.2150770000	1.0186100000
C	3.4174200000	0.7902190000	2.4336320000
C	1.3385070000	-3.7228830000	1.7647810000
C	-0.1098640000	4.0449610000	-1.2639610000
C	-2.9484600000	-2.2313140000	-2.2330970000
C	-1.1374350000	3.5489090000	-2.0112250000
C	-3.1720900000	-0.9812410000	-2.7221250000
H	3.7259860000	2.9596000000	2.1699830000
H	-0.0331650000	-5.2376770000	0.9326730000
H	4.1819140000	0.4647840000	3.1279210000
H	2.0121010000	-4.2593050000	2.4211700000
H	0.2682250000	5.0587590000	-1.2261710000
H	-3.4365020000	-3.1595910000	-2.4985670000
H	-1.7746680000	4.0737320000	-2.7117310000
H	-3.8921830000	-0.6674710000	-3.4671780000
C	1.5077680000	3.0170490000	0.3614710000
C	-1.3376330000	-3.1798830000	-0.5632400000
C	-2.1397960000	1.2659830000	-2.3008850000
C	2.3273990000	-1.4406760000	2.0787270000

H	1.9730790000	3.9896270000	0.4910070000
H	-1.7569730000	-4.1635700000	-0.7463860000
H	-2.8256120000	1.6874440000	-3.0297640000
H	3.0507270000	-1.8754090000	2.7621920000
C	-1.4213350000	1.4334810000	2.3139320000
C	-2.2612350000	-0.5602540000	2.1920040000
C	-2.9113390000	0.0633950000	3.2247910000
N	-1.3412880000	0.3042110000	1.6394780000
N	-2.3642790000	1.3343350000	3.2904170000
H	-3.6872550000	-0.2613770000	3.9002440000
H	-2.3819390000	-1.5597690000	1.8028520000
H	-0.8282180000	2.3162000000	2.1266580000
H	-2.6197820000	2.0587170000	3.9449750000
C	1.4157080000	-0.2398720000	-1.9569730000
H	2.1794350000	0.4269110000	-1.5785570000
H	0.6355960000	0.1738040000	-2.5736340000
C	1.7000610000	-1.6370710000	-2.1030520000
C	2.8238150000	-2.2489400000	-1.5073220000
N	0.8173120000	-2.3711470000	-2.8326240000
C	3.0116110000	-3.6155820000	-1.6526180000
H	3.5043390000	-1.6472970000	-0.9141570000
C	1.0291050000	-3.6542920000	-2.9449400000
C	2.0961710000	-4.3651010000	-2.3955820000
H	3.8628030000	-4.1064800000	-1.1889720000
H	2.1921900000	-5.4344520000	-2.5322120000
Br	-0.3067070000	-4.6555720000	-3.9289110000
C	2.4489750000	0.5619970000	-4.3271150000
C	1.4065730000	1.6102670000	-4.5634110000
H	1.3722380000	2.3246280000	-3.7343960000
H	1.6122380000	2.1836730000	-5.4820740000
H	0.4028790000	1.1840920000	-4.6684440000
H	3.3420410000	0.8708910000	-3.7897130000
C	2.4898340000	-0.7102560000	-4.9503480000
C	3.5925120000	-1.5767440000	-4.7226460000
C	3.6188490000	-2.8617700000	-5.2383790000
C	2.5611490000	-3.3567210000	-6.0171440000
C	1.4762400000	-2.5001590000	-6.2696240000
C	1.4307820000	-1.2176380000	-5.7498120000
H	4.4171900000	-1.2222460000	-4.1100500000
H	4.4682930000	-3.5067510000	-5.0265430000

H	0.6409810000	-2.8650640000	-6.8617350000
H	0.5614520000	-0.5977060000	-5.9411310000
C	2.5581930000	-4.7737190000	-6.5274250000
H	2.2778870000	-4.8211210000	-7.5866830000
H	3.5414030000	-5.2424460000	-6.4157240000
H	1.8294550000	-5.3793630000	-5.9731570000

Secondary benzylic radical

Charge: 0

Multiplicity: 2

B3LYP-D3(BJ)/6-31G(d)-SDD SCF energy (au):	-349.578333811
B3LYP-D3(BJ)/6-31G(d)-SDD enthalpy (au):	-349.397149811
B3LYP-D3(BJ)/6-31G(d)-SDD free energy (au):	-349.443685811
B3LYP-D3(BJ)/def2-TZVP/SMD SCF energy (au):	-349.724746504
B3LYP-D3(BJ)/def2-TZVP/SMD enthalpy (au):	-349.543562504
B3LYP-D3(BJ)/def2-TZVP/SMD free energy (au):	-349.590098504
B3LYP-D3(BJ)/def2-TZVP/SMD free energy (quasi-harmonic) (au):	-349.588202693

Cartesian coordinates

ATOM	X	Y	Z
C	3.1208120000	0.1507840000	-3.8426780000
C	2.4087500000	-0.8287720000	-4.7213940000
H	2.6756570000	-1.8578550000	-4.4650510000
H	1.3151200000	-0.7390200000	-4.6365740000
H	2.6491890000	-0.6788350000	-5.7848010000
H	3.7902260000	-0.2364360000	-3.0786580000
C	2.9815840000	1.5535040000	-3.9379650000
C	2.1343560000	2.1778030000	-4.8958490000
C	2.0206770000	3.5589340000	-4.9603610000
C	2.7333300000	4.3973200000	-4.0889400000
C	3.5733720000	3.7905940000	-3.1349240000
C	3.6978270000	2.4159280000	-3.0576850000
H	1.5655930000	1.5623430000	-5.5859450000
H	1.3632870000	4.0055540000	-5.7030140000
H	4.1335950000	4.4189410000	-2.4457800000
H	4.3519200000	1.9715190000	-2.3112990000
C	2.6214790000	5.8980130000	-4.1745560000
H	3.5453520000	6.3474900000	-4.5631870000
H	1.8040740000	6.2007000000	-4.8366990000
H	2.4400740000	6.3440960000	-3.1890630000

XI. References

(1) Arslan, E.; Schulz, H.; Zufferey, R.; Kuzler, P.; Thny-Meyer, L. Overproduction of the Bradyrhizobium japonicum c-Type Cytochrome Subunits of the cbb3 Oxidase in *Escherichia coli*. *Biochem. Biophys. Res. Commun.* **1998**, *251*, 744-747.

(2) Kille, S.; Acevedo-Rocha, C. G.; Parra, L. P.; Zhang, Z-G.; Opperman, D. J.; Reetz, M. T.; Acevedo, J. P. Reducing codon redundancy and screening effort of combinatorial protein libraries created by saturation mutagenesis. *ACS Synth. Biol.* **2013**, *2*, 83-92.

(3) Lelyveld, V. S.; Brustad, E.; Arnold, F. H.; Jasanoff, A. Metal-Substituted Protein MRI Contrast Agents Engineered for Enhanced Relaxivity and Ligand Sensitivity. *J. Am. Chem. Soc.* **2011**, *133*, 649-651.

(4) Smith, P. K.; Krohn, R.I.; Hermanson, G. T.; Mallia, A. K.; Gartner, F. H.; Provenzano, M. D.; Fujimoto, E. K.; Goeke, N. M.; Olson, B. J.; Klenk, D. C. Measurement of protein using bicinchoninic acid. *Anal. Biochem.* **1985**, *150*, 76-85.

(5) Barr, I.; Guo, F. Pyridine Hemochromagen Assay for Determining the Concentration of Heme in Purified Protein Solutions. *Bio-protocol* **2015**, *5*, e1594.

(6) Molander, G. A.; Cavalcanti, L. N. Oxidation of Organotrifluoroborates via Oxone. *J. Org. Chem.* **2011**, *76*, 623-630.

(7) Wang, T-C.; Zhang, X.; Zhang, Z.; Rao, G.; Bo, Z.; Xie, P-P.; Li, J.; Yang, Q.; Li, X.; Britt, R. D.; Wang, J.; Liu, P.; Yang, Y. Directed Evolution of Threonine Aldolases as Radical C–C Bond Forming Enzymes for Photobiocatalytic Synthesis of β-Branched-α-Tetrasubstituted Non-Canonical Amino Acids. *ChemRxiv*. **2025**, doi:10.26434/chemrxiv-2025-gcljn.

(8) Roy, S.; Wang, Y.; Zhao, X.; Dayananda, T.; Chu, J.-M.; Zhang, Y.; Fasan, R. Stereodivergent Synthesis of Pyridyl Cyclopropanes via Enzymatic Activation of Pyridotriazoles. *J. Am. Chem. Soc.* **2024**, *146*, 19673−19679.

(9) Zalessky, I.; Wootton, J. M.; Tam, J. K. F.; Spurling, D. E.; Glover-Humphreys, W. C.; Donald, J. R.; Orukotan, W. E.; Duff, L. C.; Knapper, B. J.; Whitwood, A. C.; Tanner, T. F. N.; Miah, A. H.; Lynam, J. M.; Unsworth, W. P. A Modular Strategy for the Synthesis of Macrocycles and Medium-Sized Rings via Cyclization/Ring Expansion Cascade Reactions. *J. Am. Chem. Soc.* **2024**, *146*, 5702−5711.

(10) Jing, C.; Mao, W.; Bower, J. F. Iridium Catalyzed Enantioselective Alkene Hydroalkylation via a Heteroaryl Directed Enolization-Decarboxylation Sequence. *J. Am. Chem. Soc.* **2023**, *145*, 23918−23924.

(11) Derat, E.; Cohen, S.; Shaik, S.; Altun, A.; Thiel, W.; Principal Active Species of Horseradish Peroxidase, Compound I: A Hybrid Quantum Mechanical/Molecular Mechanical Study. *J. Am. Chem. Soc.* **2005**, *127,* 13611-13621.

(12) Frisch, M. J.; Trucks, G. W.; Schlegel, H. B.; Scuseria, G. E.; Robb, M. A.; Cheeseman, J. R.; Scalmani, G.; Barone, V.; Petersson, G. A.; Nakatsuji, H.; Li, X.; Caricato, M.; Marenich, A. V.; Bloino, J.; Janesko, B. G.; Gomperts, R.; Mennucci, B.; Hratchian, H. P.; Ortiz, J. V.; Izmaylov, A. F.; Sonnenberg, J. L.; Williams, F.; Lipparini, F.; Egidi, F.; Goings, J.; Peng, B.; Petrone, A.; Henderson, T.; Ranasinghe, D.; Zakrzewski, V. G.; Gao, J.; Rega, N.; Zheng, G.; Liang, W.; Hada, M.; Ehara, M.; Toyota, K.; Fukuda, R.; Hasegawa, J.; Ishida, M.; Nakajima, T.; Honda, Y.; Kitao, O.; Nakai, H.; Vreven, T.; Throssell, K.; Montgomery Jr., J. A.; Peralta, J. E.; Ogliaro, F.; Bearpark, M. J.; Heyd, J. J.; Brothers, E. N.; Kudin, K. N.; Staroverov, V. N.; Keith, T. A.; Kobayashi, R.; Normand, J.; Raghavachari, K.; Rendell, A. P.; Burant, J. C.; Iyengar, S. S.; Tomasi, J.; Cossi, M.; Millam, J. M.; Klene, M.; Adamo, C.; Cammi, R.; Ochterski, J. W.; Martin, R. L.; Morokuma, K.; Farkas, O.; Foresman, J. B.; Fox, D. J.; Gaussian 16 Rev. C.01 (Wallingford, CT, 2016).

(13) S. Grimme, S. Ehrlich, L. Goerigk, Effect of the damping function in dispersion corrected density functional theory. *J. Comput. Chem.* **32**, 1456-1465 (2011).

(14) (a) C. Lee, W. Yang, R. G. Parr, Development of the Colle-Salvetti correlation-energy formula into a functional of the electron density. *Phys. Rev. B* **37**, 785-789 (1988). (b) A. D. Becke, Density-functional thermochemistry. III. The role of exact exchange. *J. Chem. Phys.* **98**, 5648-5652 (1993).

(15) (a) Dolg, M.; Wedig, U.; Stoll, H.; Preuss, H. Energy-adjusted *ab initio* pseudopotentials for the first row transition elements. *J. Chem. Phys.* **1987,** *86*, 866-872. (b) Martin, J. M. L.; Sundermann, A. Correlation consistent valence basis sets for use with the Stuttgart–Dresden–Bonn relativistic effective core potentials: The atoms Ga–Kr and In–Xe. *J. Chem. Phys.* **2001,** *114*, 3408-3420.

(16) R. F. Ribeiro, A. V. Marenich, C. J. Cramer, D. G. Truhlar, Use of solution-phase vibrational frequencies in continuum models for the free energy of solvation. *J. Phys. Chem. B* **115**, 14556-14562 (2011).

(17) G. Luchini, J. V. Alegre-Requena, I. Funes-Ardoiz, R. S. Paton, GoodVibes: Automated thermochemistry for heterogeneous computational chemistry data. *F1000 Research* **9**, 219 (2020).

(18) F. Weigend, R. Ahlrichs, Balanced basis sets of split valence, triple zeta valence and quadruple zeta valence quality for H to Rn: Design and assessment of accuracy. *Phys. Chem. Chem. Phys.* **7**, 3297-3305 (2005).

(19) A. V. Marenich, C. J. Cramer, D. G. Truhlar, Universal solvation model based on solute electron density and on a continuum model of the solvent defined by the bulk dielectric constant and atomic surface tensions. *J. Phys. Chem. B* **113**, 6378-6396 (2009).

(20) (a) C. N. Schutz, A. Warshel, What are the dielectric "constants" of proteins and how to validate electrostatic models? *Proteins* **44**, 400-417 (2001). (b) J. Rui, Q. Zhao, A. J. Huls, J. Soler, J. C. Paris, Z. Chen, V. Reshetnikov, Y. Yang, Y. Guo, M. Garcia-Borràs, X. Huang, Directed evolution of nonheme iron enzymes to access abiological radical-relay C(sp^3)−H azidation. *Science* **376**, 869-874 (2022).

(21) Wang, H., Mai, B.K., Li, C. *et al.* Stereoselective photometallobiocatalytic cross-coupling of organoboron reagents and diazo compounds via an outer-sphere mechanism. *Nat Catal* **9**, 530–541 (2026). https://doi.org/10.1038/s41929-026-01532-9

(22) (a) Hur, S.; Bruice, T. C. The near attack conformation approach to the study of the chorismate to prephenate reaction. *Proc. Natl. Acad. Sci. U.S.A.* **2003,** *100*, 12015-12020. (b) A. Knorrscheidt, J. Soler, N. Hünecke, P. Püllmann, M. Garcia-Borràs, M. J. Weissenborn, Accessing Chemo- and Regioselective Benzylic and Aromatic Oxidations by Protein Engineering of an Unspecific Peroxygenase. *ACS Catal.* **11**, 7327-7338 (2021). (c) L. Schaus, A. Das, A. M. Knight, G. Jimenez-Osés, K. N. Houk, M. Garcia-Borràs, F. H. Arnold, X. Huang, Protoglobin-Catalyzed Formation of cis-Trifluoromethyl-Substituted Cyclopropanes by Carbene Transfer. *Angew. Chem. Int. Ed.* **62**, e202208936 (2023).

(23) J. Abramson, J. Adler, J. Dunger, R. Evans, T. Green, A. Pritzel, O. Ronneberger, L. Willmore, A. J. Ballard, J. Bambrick, S. W. Bodenstein, D. A. Evans, C.-C. Hung, M. O'Neill, D. Reiman, K. Tunyasuvunakool, Z. Wu, A. Žemgulytė, E. Arvaniti, C. Beattie, O. Bertolli, A. Bridgland, A. Cherepanov, M. Congreve, A. I. Cowen-Rivers, A. Cowie, M. Figurnov, F. B. Fuchs, H. Gladman, R. Jain, Y. A. Khan, C. M. R. Low, K. Perlin, A. Potapenko, P. Savy, S. Singh, A. Stecula, A. Thillaisundaram, C. Tong, S. Yakneen, E. D. Zhong, M. Zielinski, A. Žídek, V. Bapst, P. Kohli, M. Jaderberg, D. Hassabis, J. M. Jumper, Accurate structure prediction of biomolecular interactions with AlphaFold 3. *Nature* **630**, 493-500 (2024).
(24) R. Salomon-Ferrer, A. W. Götz, D. Poole, S. Le Grand, R. C. Walker, Routine microsecond molecular dynamics simulations with AMBER on GPUs. 2. Explicit solvent particle mesh Ewald. *J. Chem. Theory Comput.* **9**, 3878-3888 (2013).
(25) D. A. Case, H. M. Aktulga, K. Belfon, I. Y. Ben-Shalom, J. T. Berryman, S. R. Brozell, F. S. Carvahol, D. S. Cerutti, I. T.E. Cheatham, G. A. Cisneros, V. W. D. Cruzeiro, T. A. Darden, N. Forouzesh, M. Ghazimirsaeed, G. Giambaşu, T. Giese, M. K. Gilson, H. Gohlke, A. W. Goetz, J. Harris, Z. Huang, S. Izadi, S. A. Izmailov, K. Kasavajhala, M. C. Kaymak, I. Kolossvary, A. Kovalenko, T. Kurtzman, T. S. Lee, P. Li, Z. Li, C. Lin, J. Liu, T. Luchko, R. Luo, M. Machado, M. Manathunga, K. M. Merz, Y. Miao, O. Mikhailovskii, G. Monard, H. Nguyen, K. A. O'Hearn, A. Onufriev, F. Pan, S. Pantano, A. Rahnamoun, D. R. Roe, A. Roitberg, C. Sagui, S. Schott-Verdugo, A. Shajan, J. Shen, C. L. Simmerling, N. R. Skrynnikov, J. Smith, J. Swails, R. C. Walker, J. Wang, J. Wang, X. Wu, Y. Wu, Y. Xiong, Y. Xue, D. M. York, C. Zhao, Q. Zhu, P. A. Kollman, Amber 2025 (University of California, San Francisco, CA, 2025).
(26) B. Tian, K. Kasavajhala, K. A. A. Belfon, L. Raguette, H. Huang, A. N. Migues, J. Bickel, Y. Wang, J. Pincay, Q. Wu, C. Simmerling, ff19SB: Amino-Acid-Specific Protein Backbone Parameters Trained against Quantum Mechanics Energy Surfaces in Solution. *J. Chem. Theory Comput.* **16**, 528-552 (2020).
(27) X. He, V. H. Man, W. Yang, T.-S. Lee, J. Wang, A fast and high-quality charge model for the next generation general AMBER force field. *J. Chem. Phys.* **153**, 114502 (2020).
(28) P. Li, K. M. Merz, MCPB.py: A python based metal center parameter builder. *J. Chem. Inf. Model.* **56**, 599-604 (2016).
(29) U. C. Singh, P. A. Kollman, An approach to computing electrostatic charges for molecules.

J. Comp. Chem. **5**, 129-145 (1984). B. H. Besler, K. M. Merz Jr, P. A. Kollman, Atomic charges derived from semiempirical methods. *J. Comp. Chem.* **11**, 431-439 (1990).

(30) C. I. Bayly, P. Cieplak, W. Cornell, P. A. Kollman, A well-behaved electrostatic potential based method using charge restraints for deriving atomic charges: the RESP model. *J. Phys. Chem.* **97**, 10269-10280 (1993).

(31) R. Anandakrishnan, B. Aguilar, A. V. Onufriev, H++ 3.0: Automating pK prediction and the preparation of biomolecular structures for atomistic molecular modeling and simulations. *Nucleic Acids Res.* **40**, W537-W541 (2012).

(32) S. Izadi, A. V. Onufriev, Accuracy limit of rigid 3-point water models. *J. Chem. Phys.* **145**, 074501 (2016).

(33) J.-P. Ryckaert, G. Ciccotti, H. J. C. Berendsen, Numerical integration of the cartesian equations of motion of a system with constraints: Molecular dynamics of *n*-alkanes. *J. Comput. Phys.* **23**, 327-341 (1977).

(34) T. Darden, D. York, L. Pedersen, Particle mesh Ewald: An $N\cdot\log(N)$ method for Ewald sums in large systems. *J. Chem. Phys.* **98**, 10089-10092 (1993).

(35) D. R. Roe, T. E. Cheatham, PTRAJ and CPPTRAJ: Software for processing and analysis of molecular dynamics trajectory data. *J. Chem. Theory Comput.* **9**, 3084-3095 (2013).

XII. ^{1}H, ^{13}C and ^{19}F spectra of compounds

Attached as a separate .pdf file.

^{1}H NMR (CDCl₃, 400 MHz) spectrum of 7-(methylthio)-[1,2,3]triazolo[1,5-c]pyrimidine (2e)

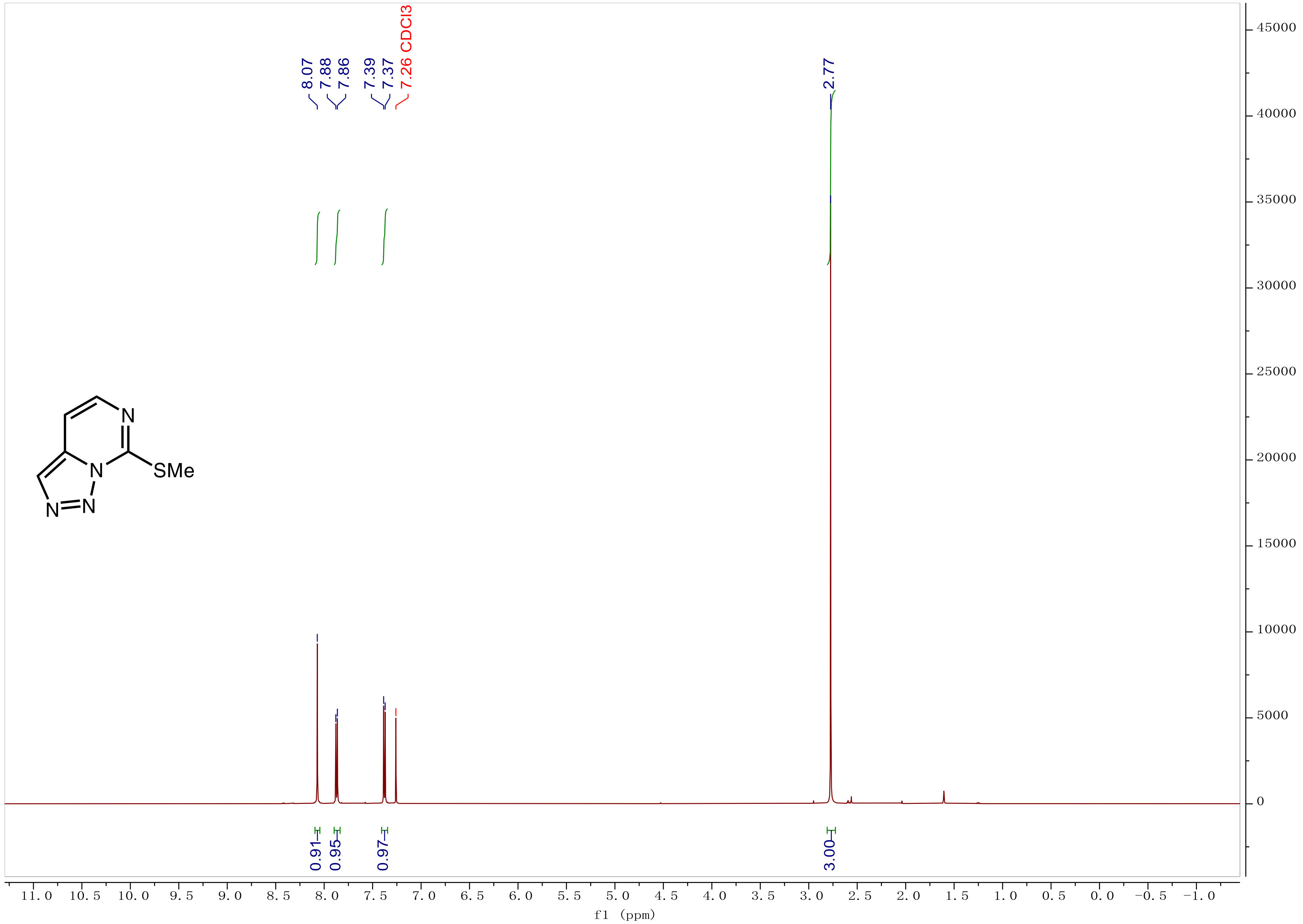

^{13}C NMR ($CDCl_3$, 100 MHz) spectrum of 7-(methylthio)-[1,2,3]triazolo[1,5-c]pyrimidine (2e)

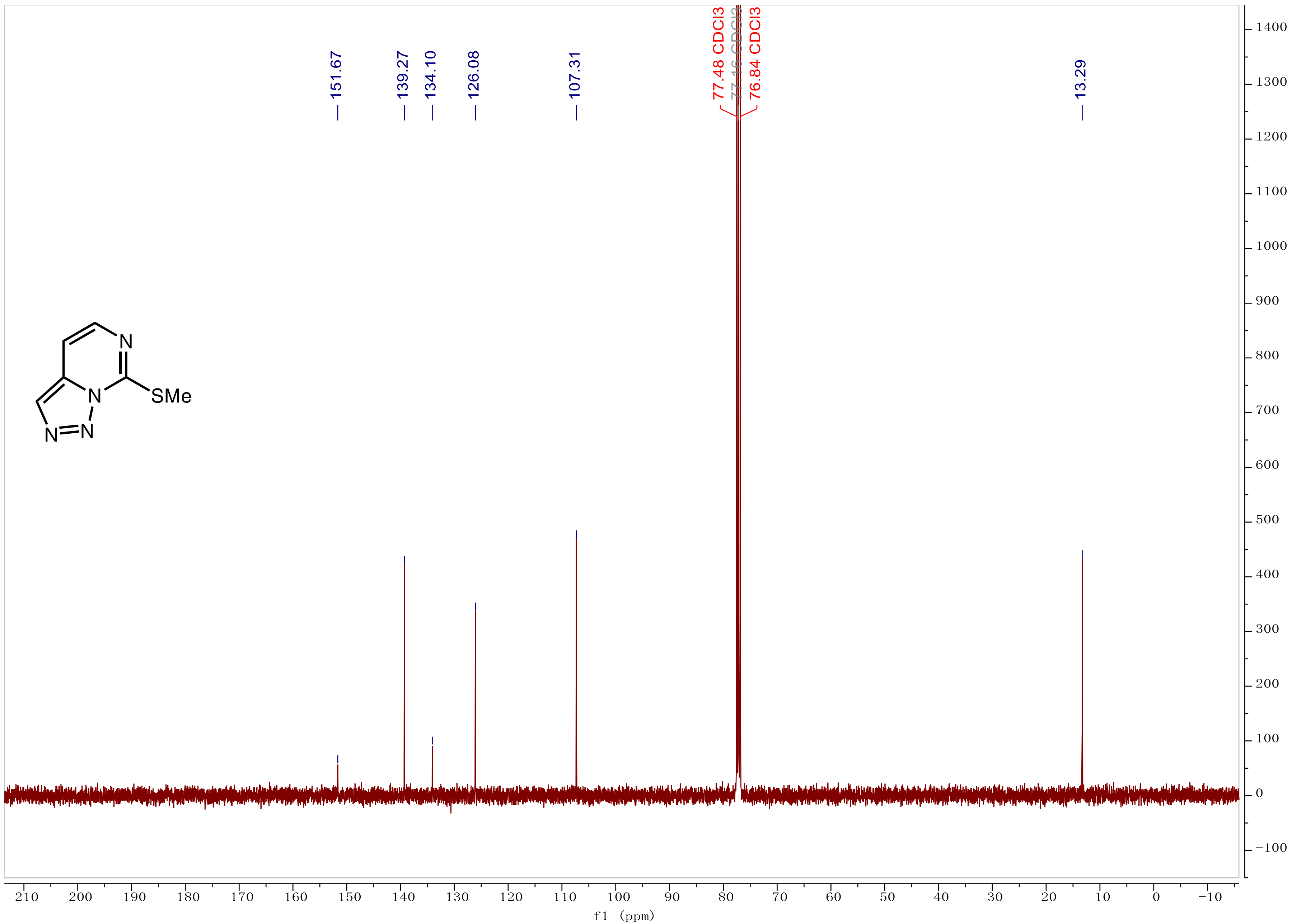

^{1}H NMR ($CDCl_3$, 400 MHz) spectrum of 2-Bromo-6-(2-(*p*-tolyl)propyl)pyridine (3a)

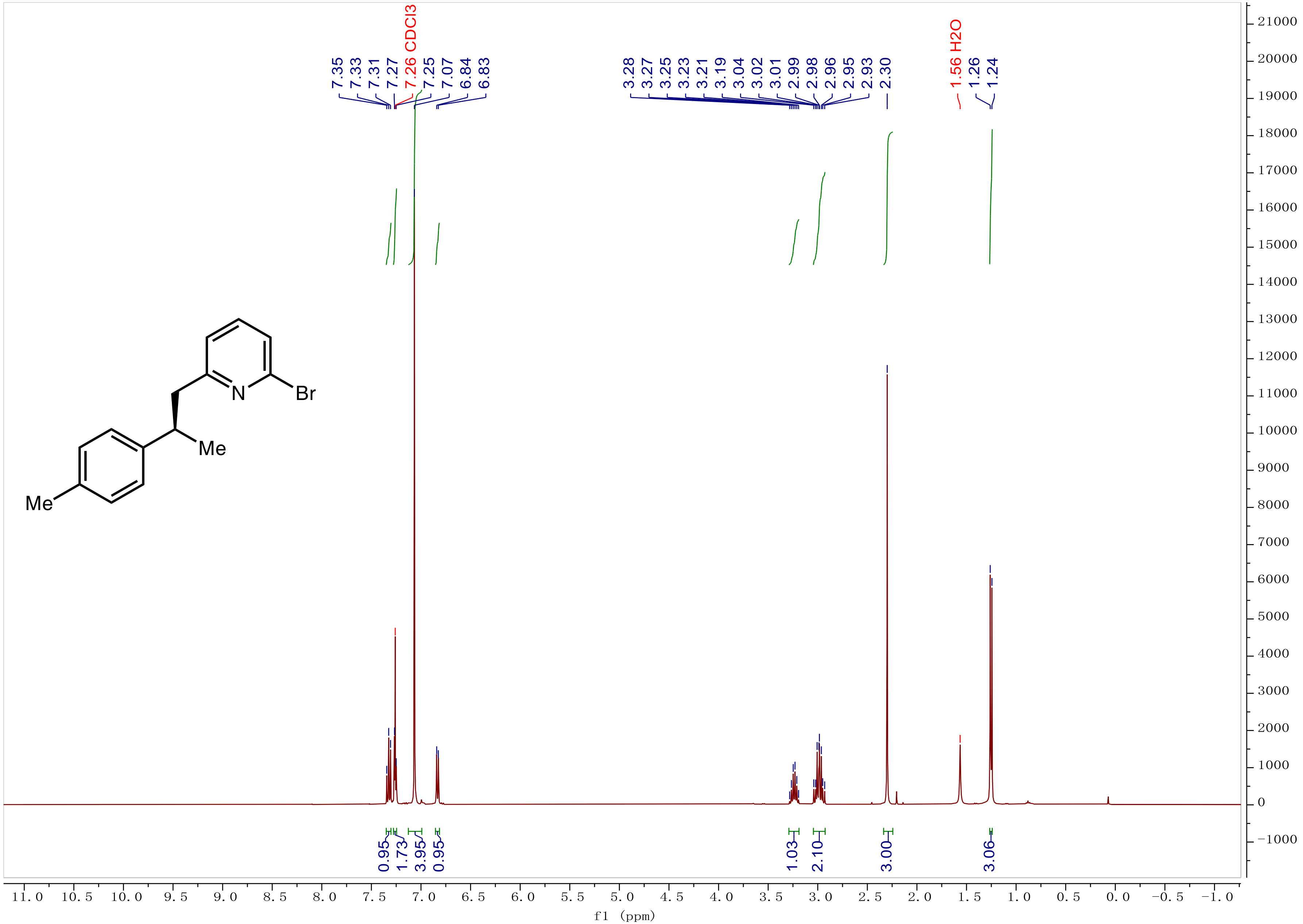

^{13}C NMR ($CDCl_3$, 100 MHz) spectrum of 2-Bromo-6-(2-(*p*-tolyl)propyl)pyridine (3a)

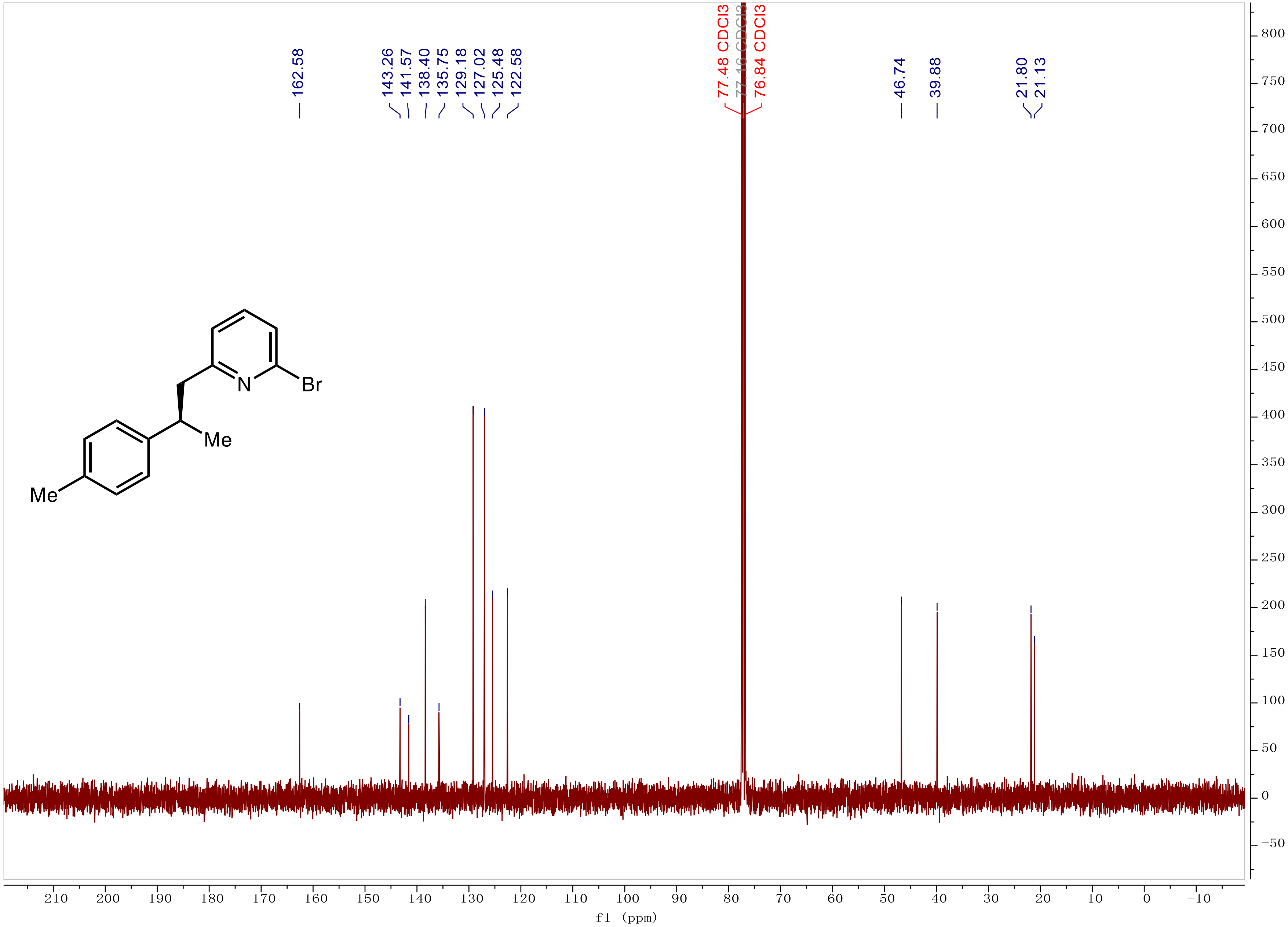

^{1}H NMR ($CDCl_3$, 400 MHz) spectrum of 2-Bromo-6-(2-(*o*-tolyl)propyl)pyridine (3b)

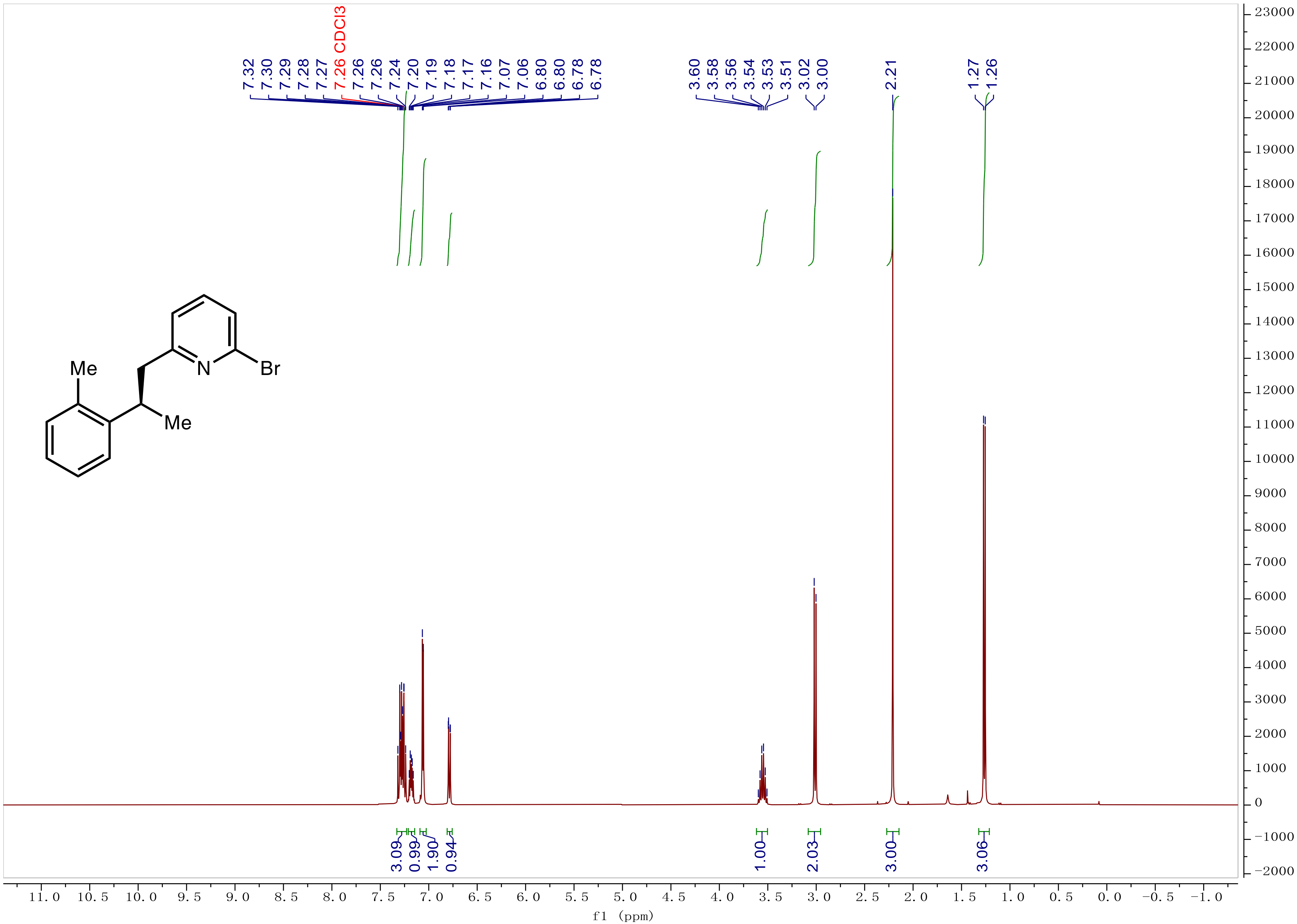

^{13}C NMR ($CDCl_3$, 100 MHz) spectrum of 2-Bromo-6-(2-(*o*-tolyl)propyl)pyridine (3b)

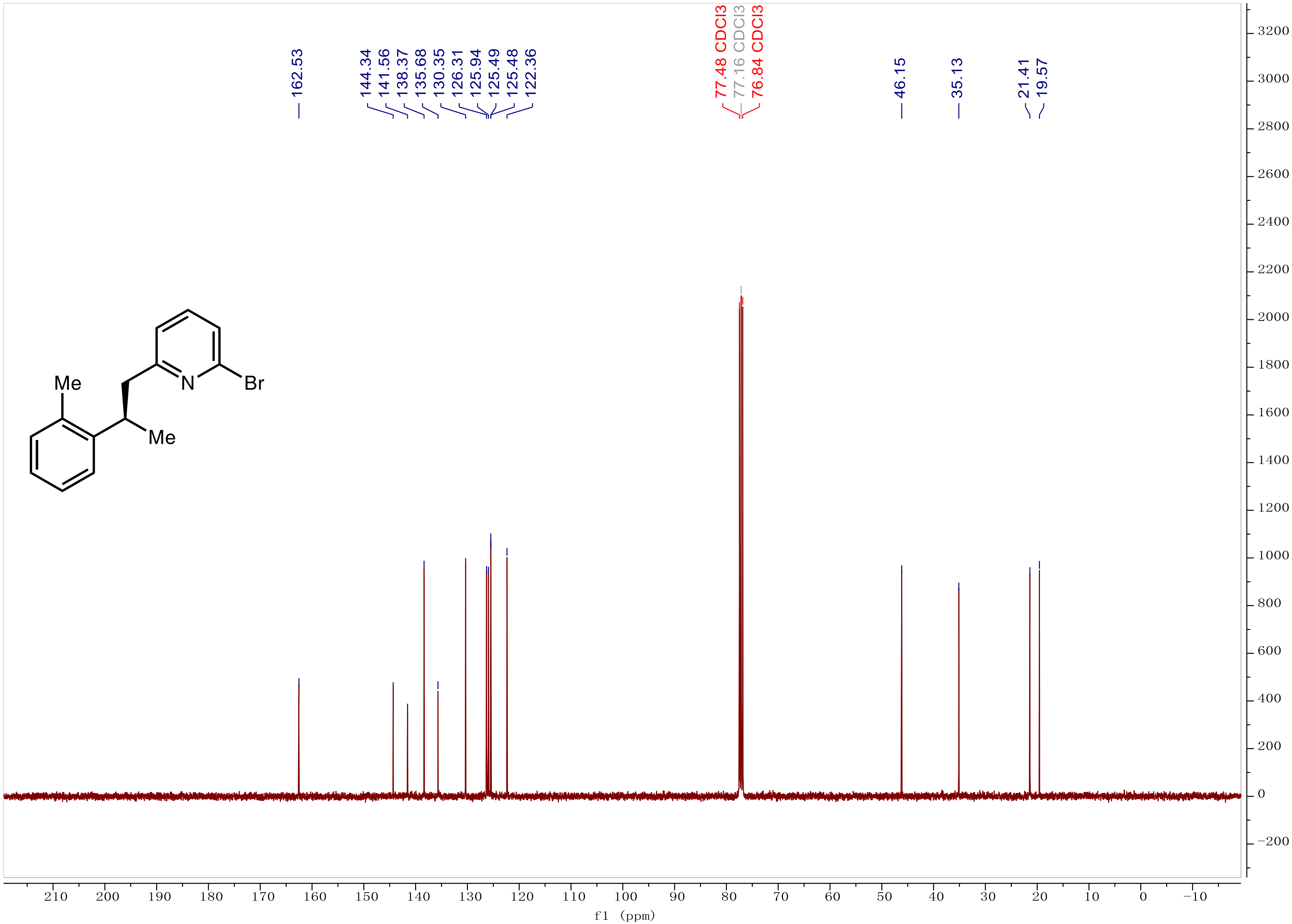

^{1}H NMR ($CDCl_3$, 400 MHz) spectrum of 2-Bromo-6-(2-(m-tolyl)propyl)pyridine (3c)

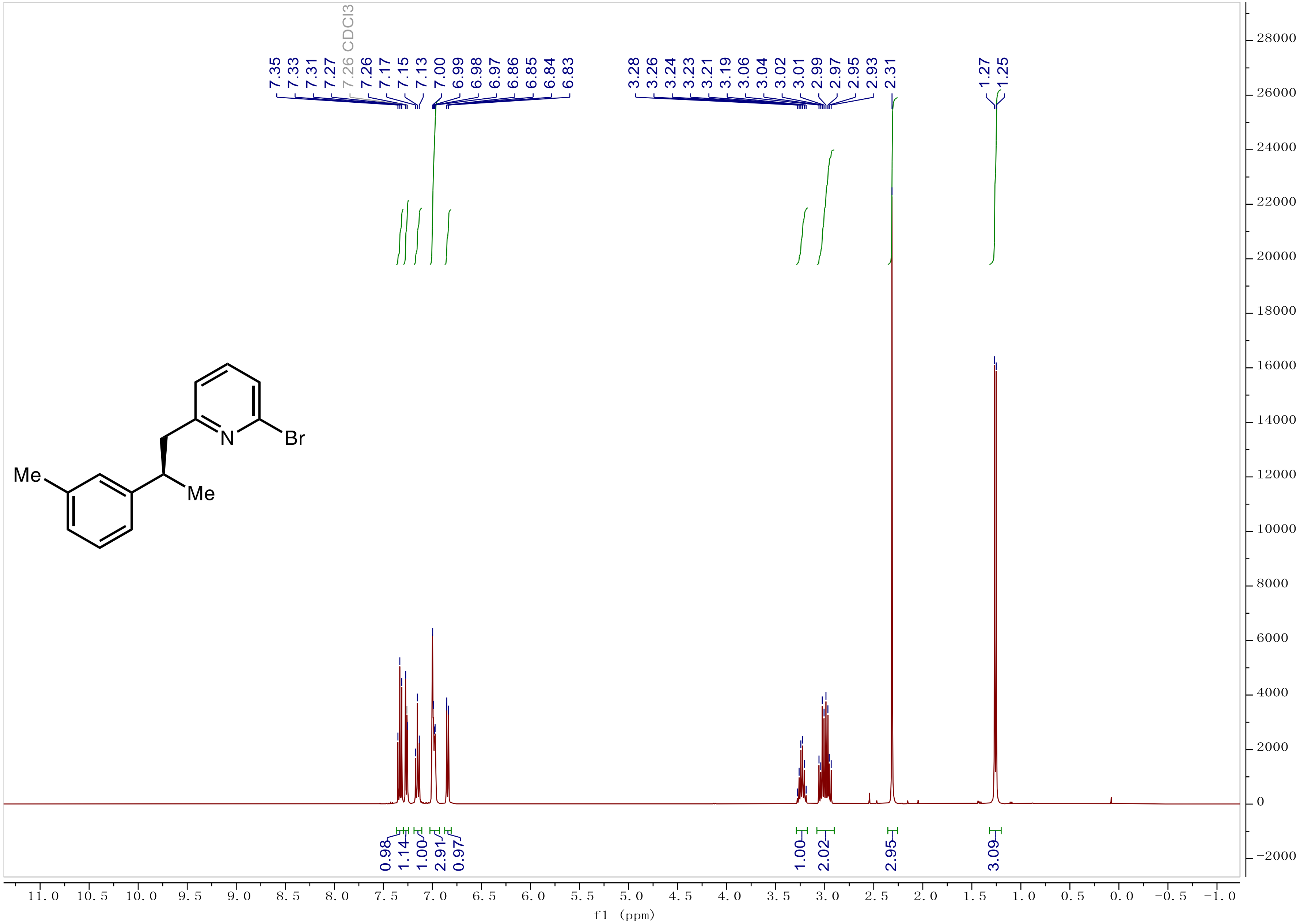

^{13}C NMR ($CDCl_3$, 100 MHz) spectrum of 2-Bromo-6-(2-(m-tolyl)propyl)pyridine (3c)

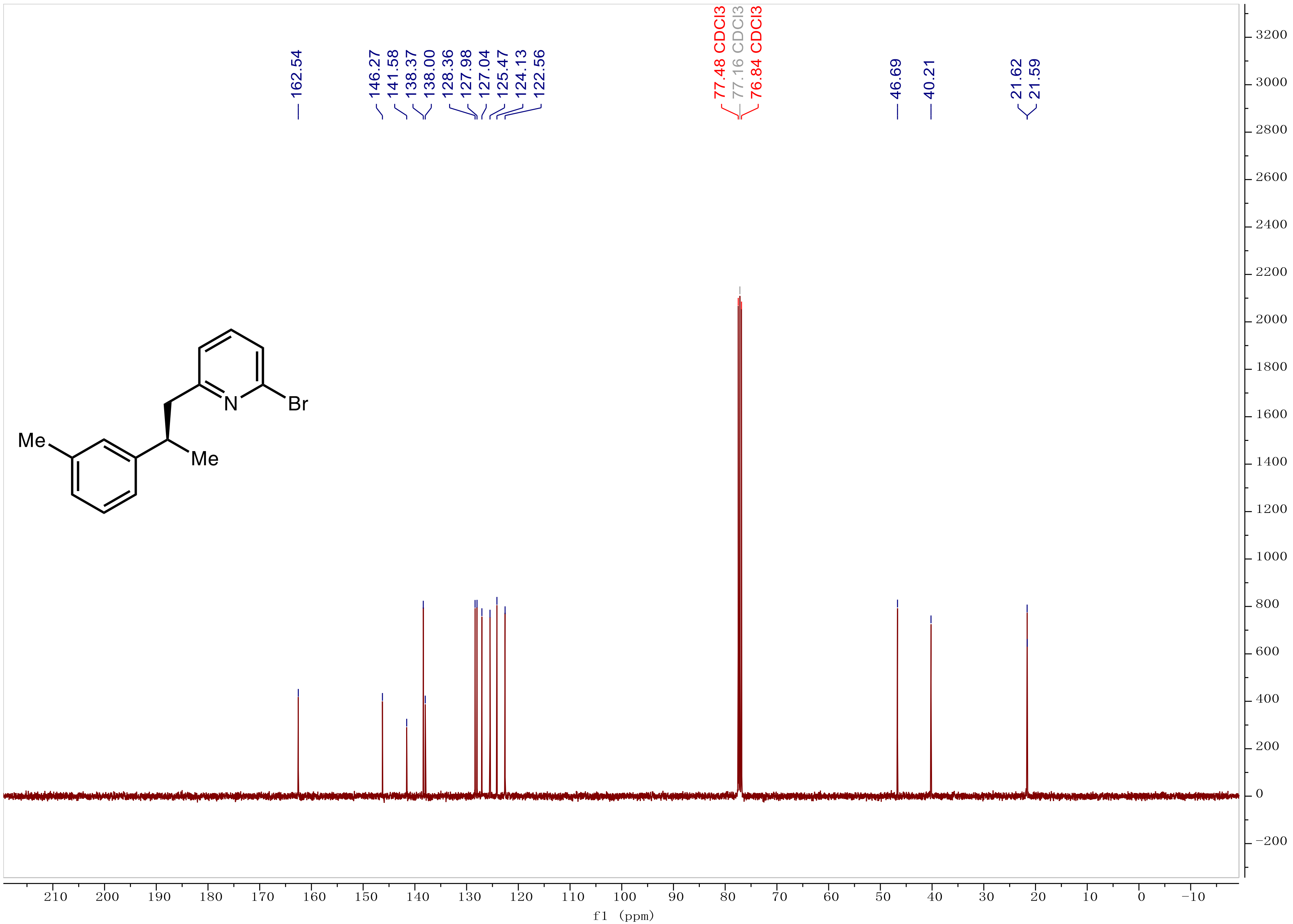

^{1}H NMR ($CDCl_3$, 400 MHz) spectrum of 2-Bromo-6-(2-(4-methoxyphenyl)propyl)pyridine (3d)

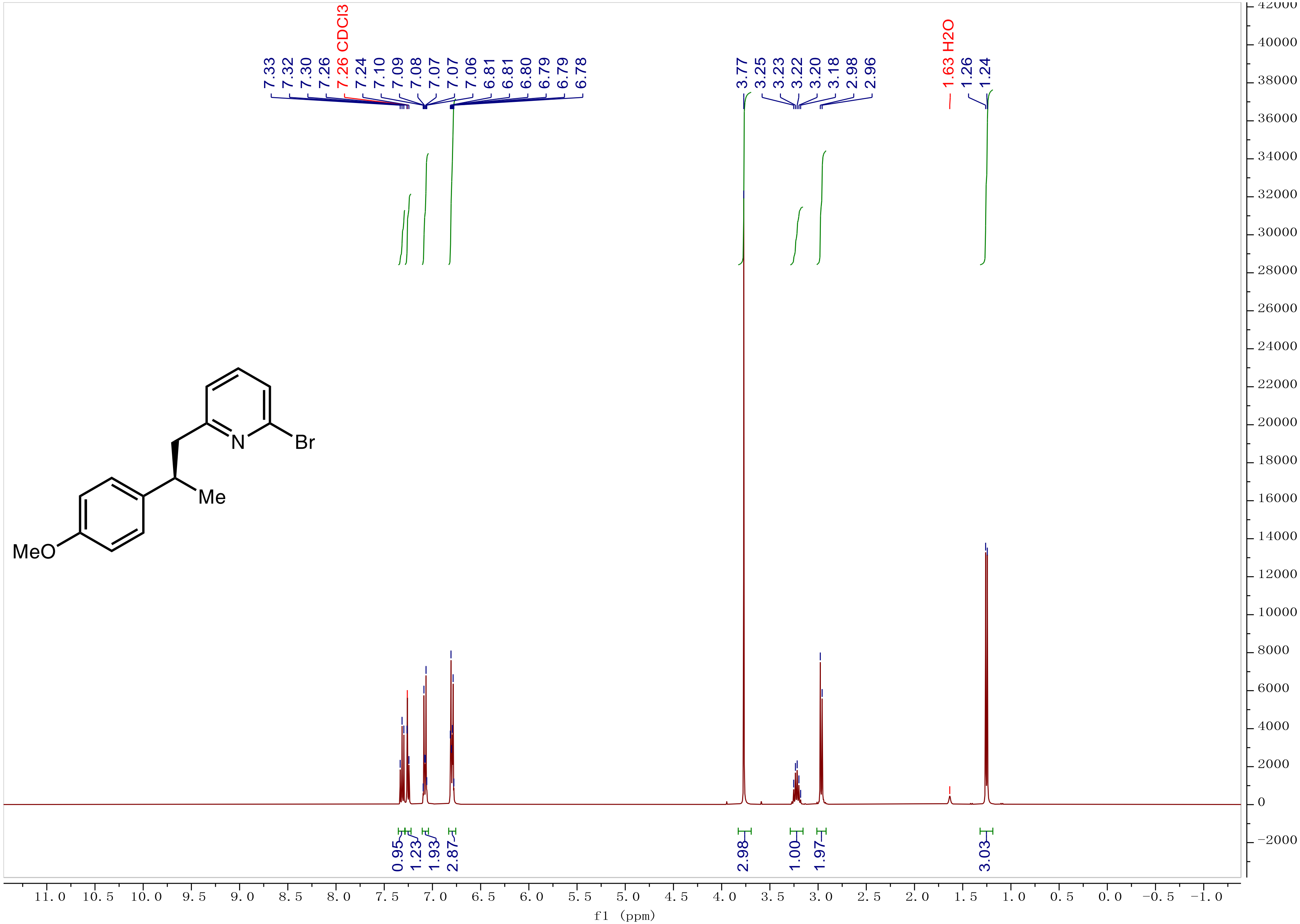

^{13}C NMR (CDCl3, 100 MHz) spectrum of 2-Bromo-6-(2-(4-methoxyphenyl)propyl)pyridine (3d)

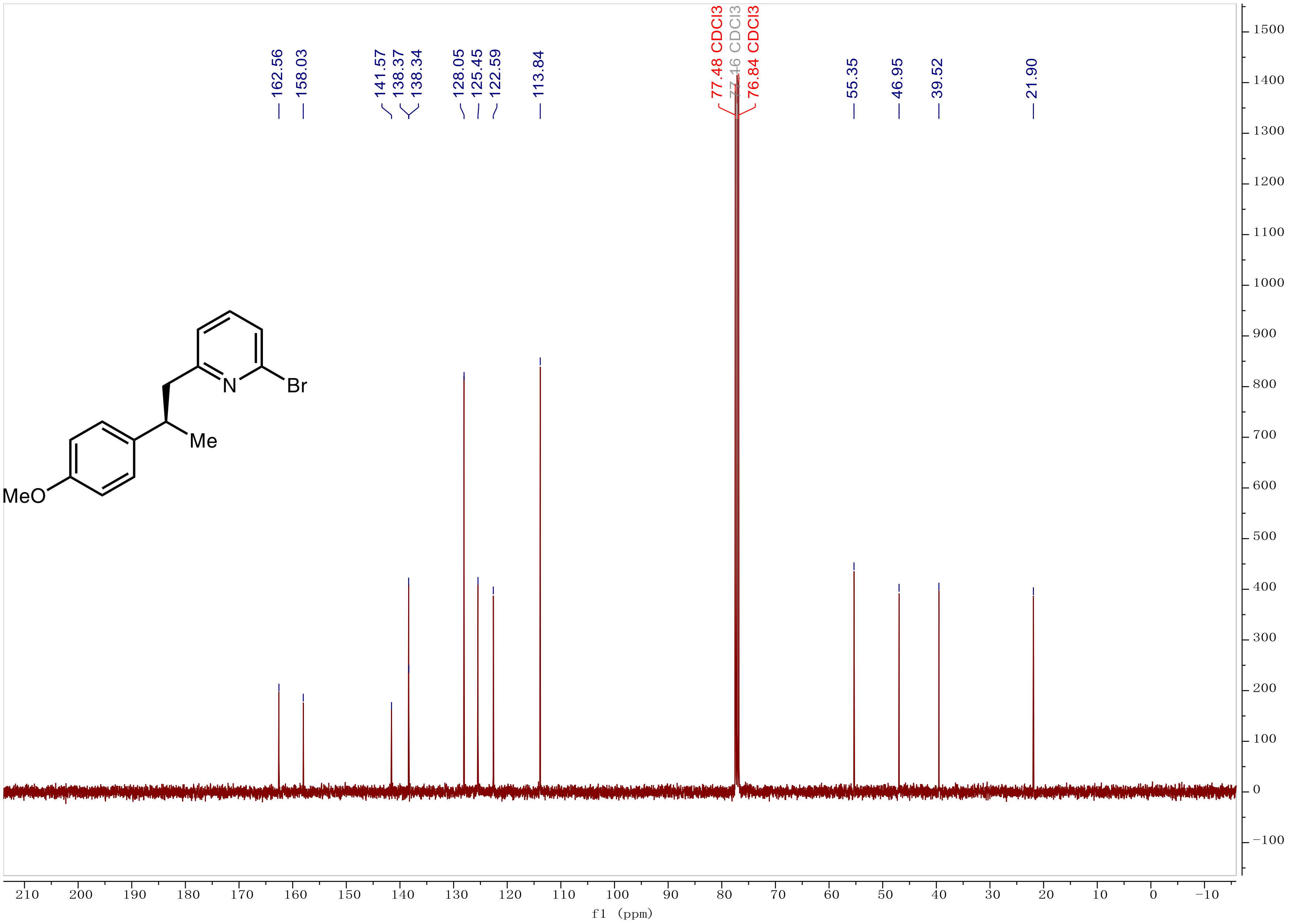

^{1}H NMR ($CDCl_3$, 400 MHz) spectrum of 2-Bromo-6-(2-(4-(methylthio)phenyl)propyl)pyridine (3e)

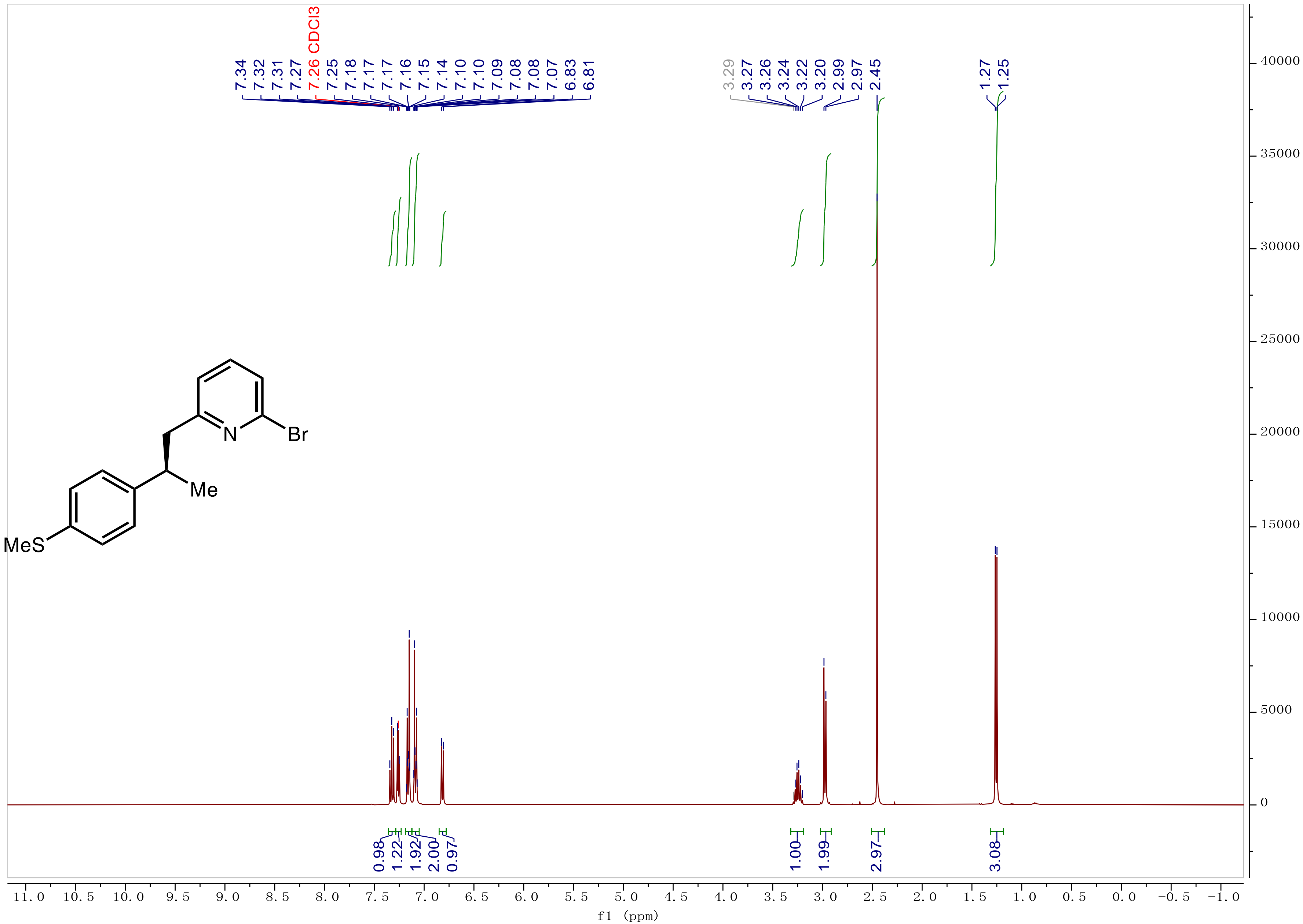

^{13}C NMR ($CDCl_3$, 100 MHz) spectrum of 2-Bromo-6-(2-(4-(methylthio)phenyl)propyl)pyridine (3e)

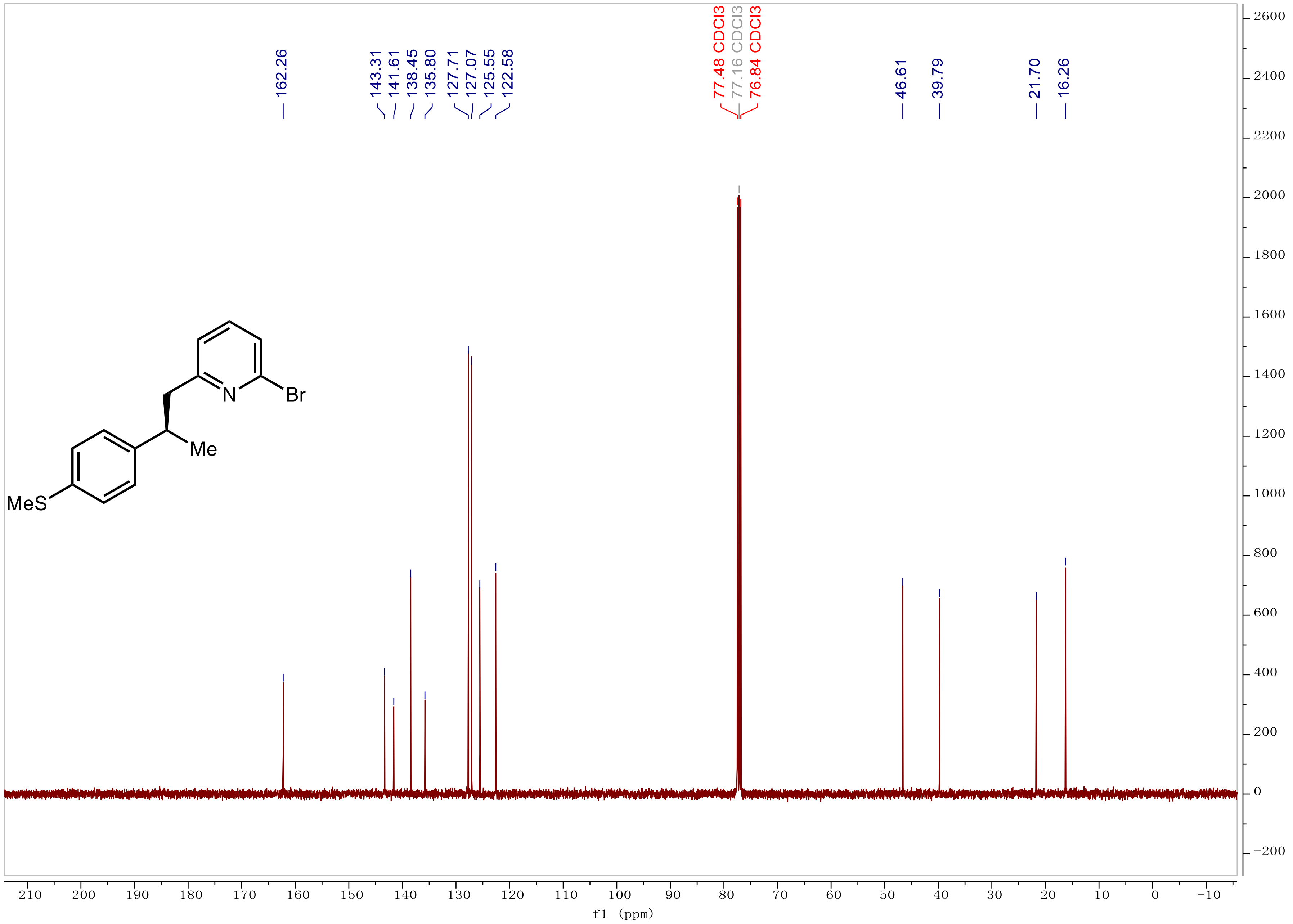

^{1}H NMR ($CDCl_3$, 400 MHz) spectrum of 2-bromo-6-(2-(4-(trifluoromethoxy)phenyl)propyl)pyridine (3f)

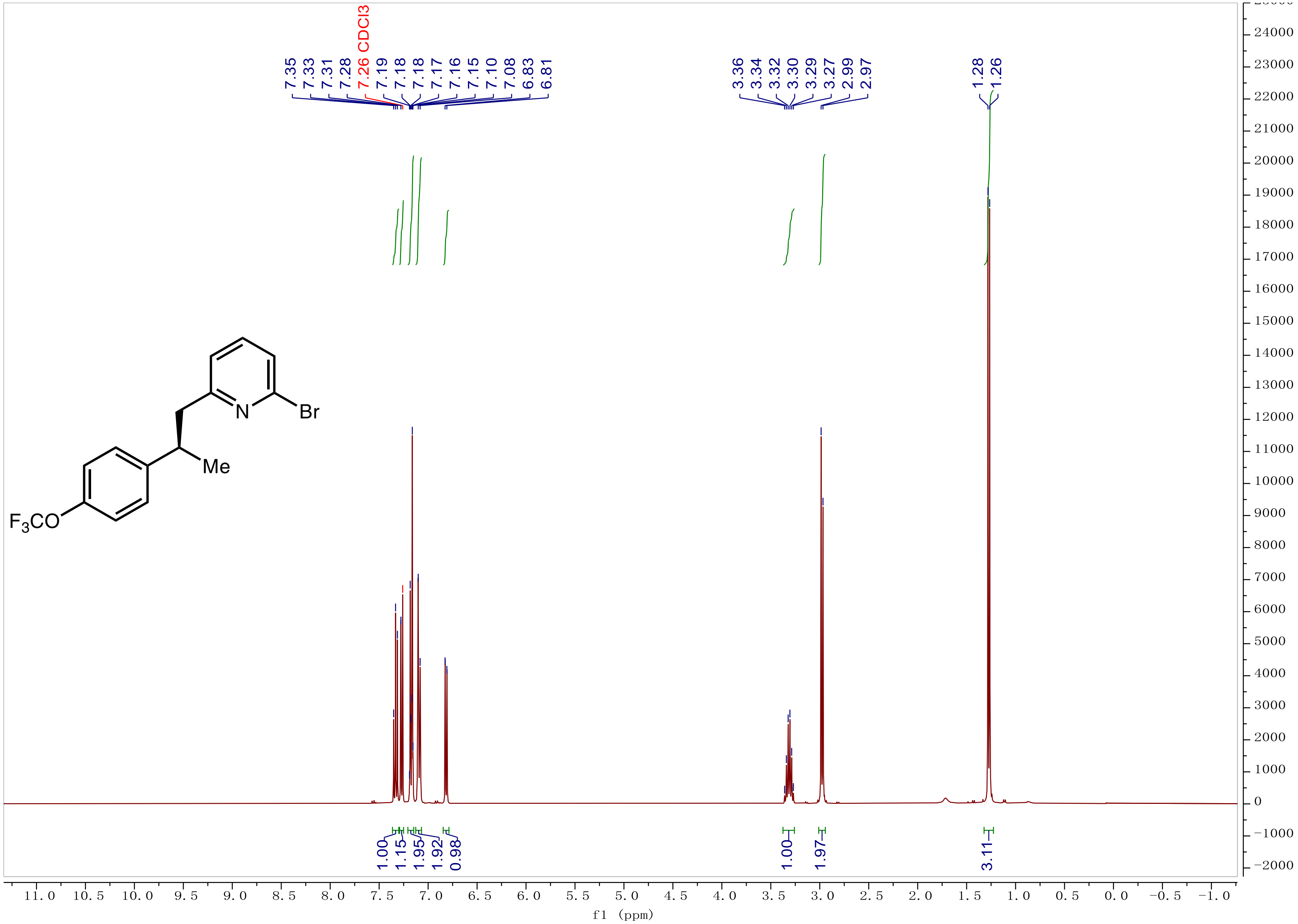

^{13}C NMR ($CDCl_3$, 100 MHz) spectrum of 2-bromo-6-(2-(4-(trifluoromethoxy)phenyl)propyl)pyridine (3f)

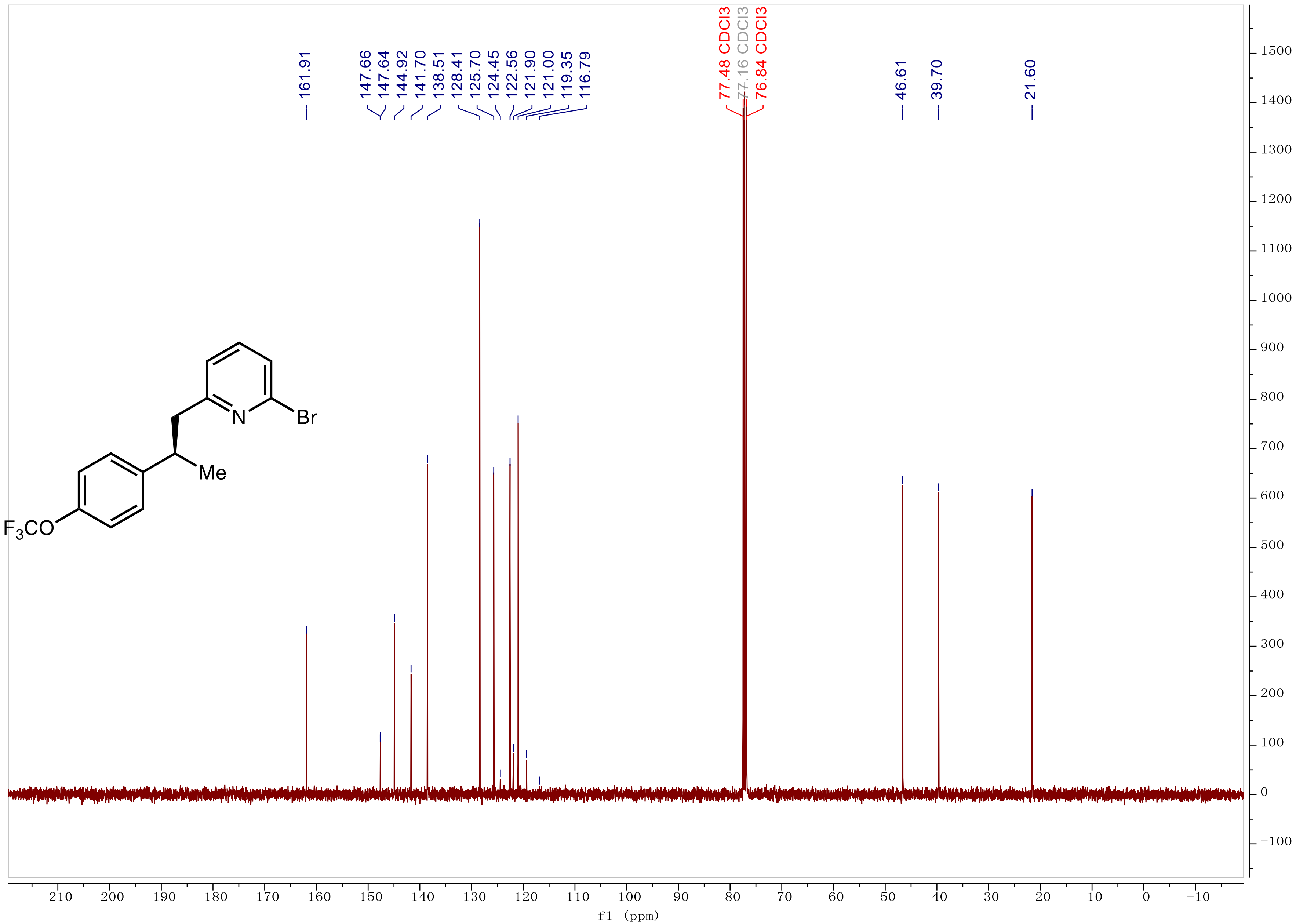

^{19}F NMR ($CDCl_3$, 376 MHz) spectrum of 2-bromo-6-(2-(4-(trifluoromethoxy)phenyl)propyl)pyridine (3f)

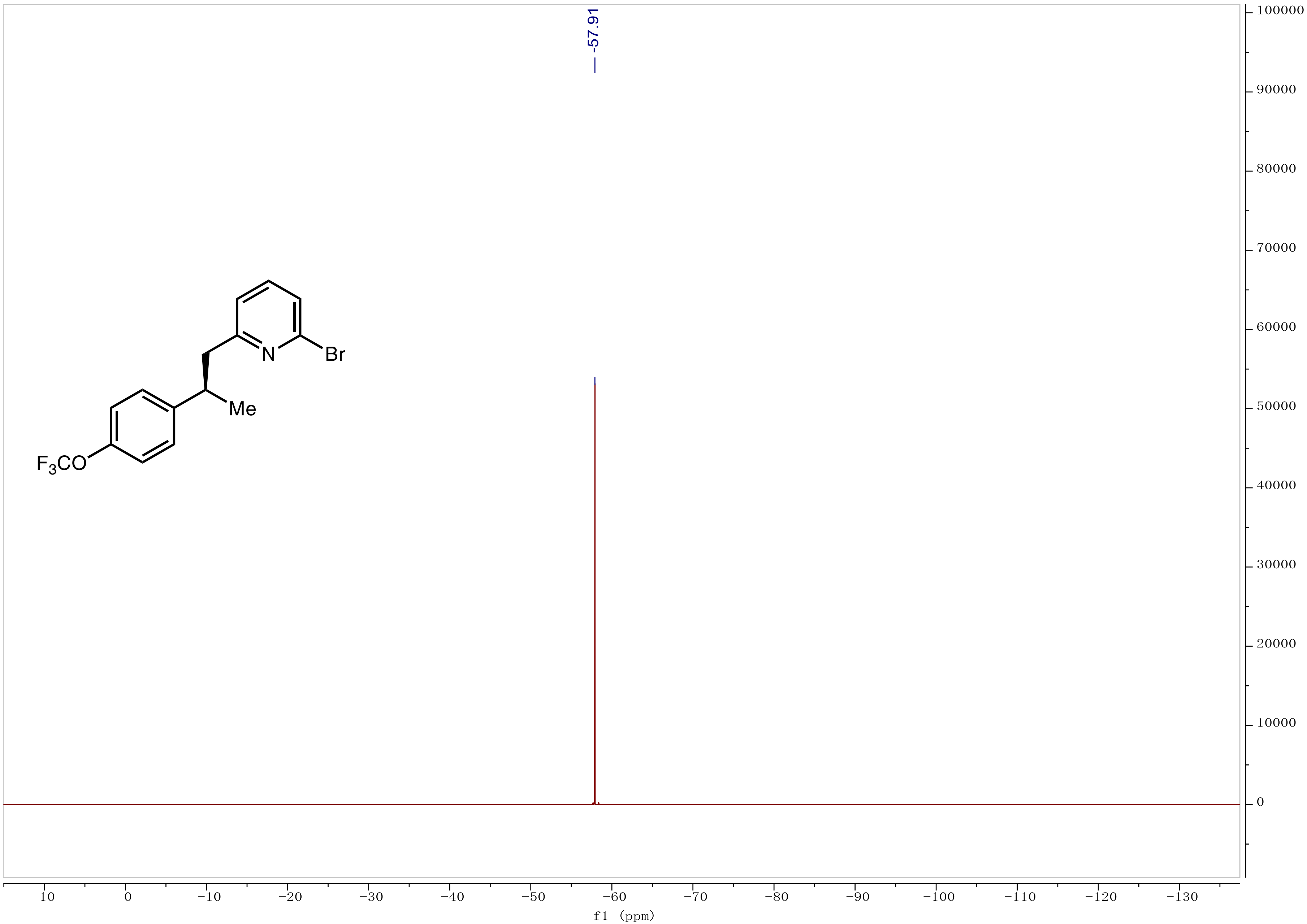

^{1}H NMR ($CDCl_3$, 400 MHz) spectrum of 2-Bromo-6-(2-(4-fluorophenyl)propyl)pyridine (3g)

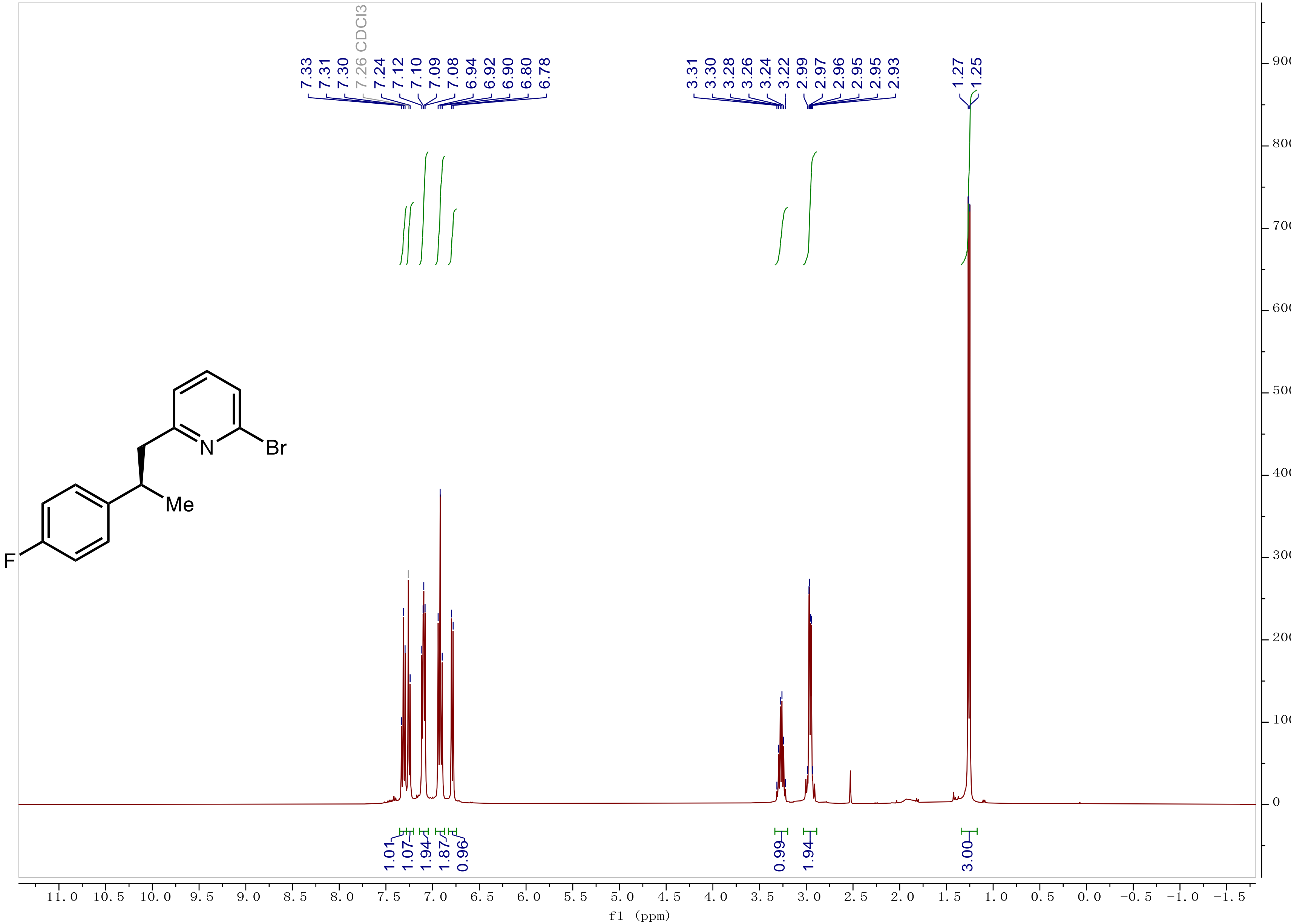

^{13}C NMR ($CDCl_3$, 100 MHz) spectrum of 2-Bromo-6-(2-(4-fluorophenyl)propyl)pyridine (3g)

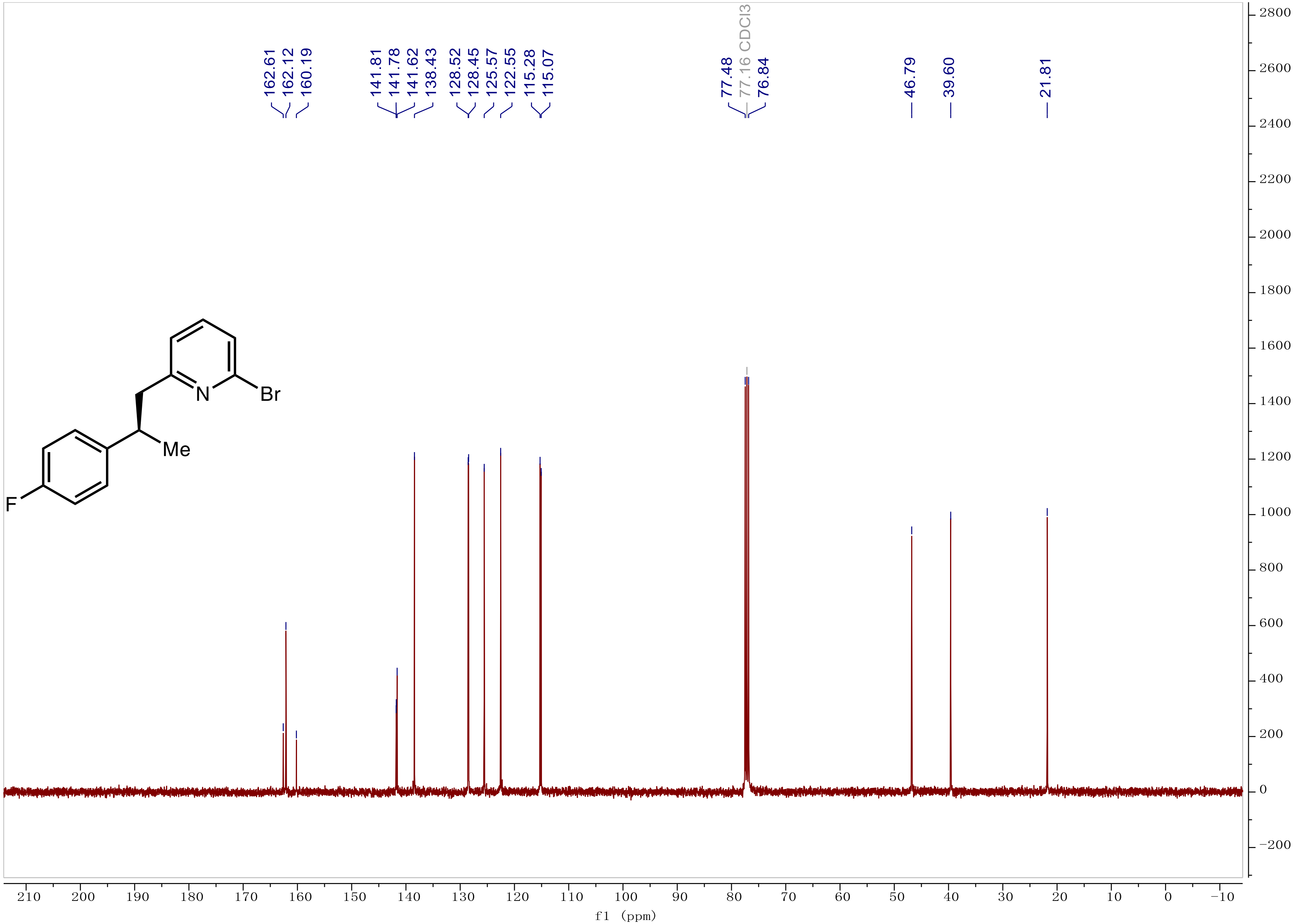

^{19}F NMR ($CDCl_3$, 376 MHz) spectrum of 2-Bromo-6-(2-(4-fluorophenyl)propyl)pyridine (3g)

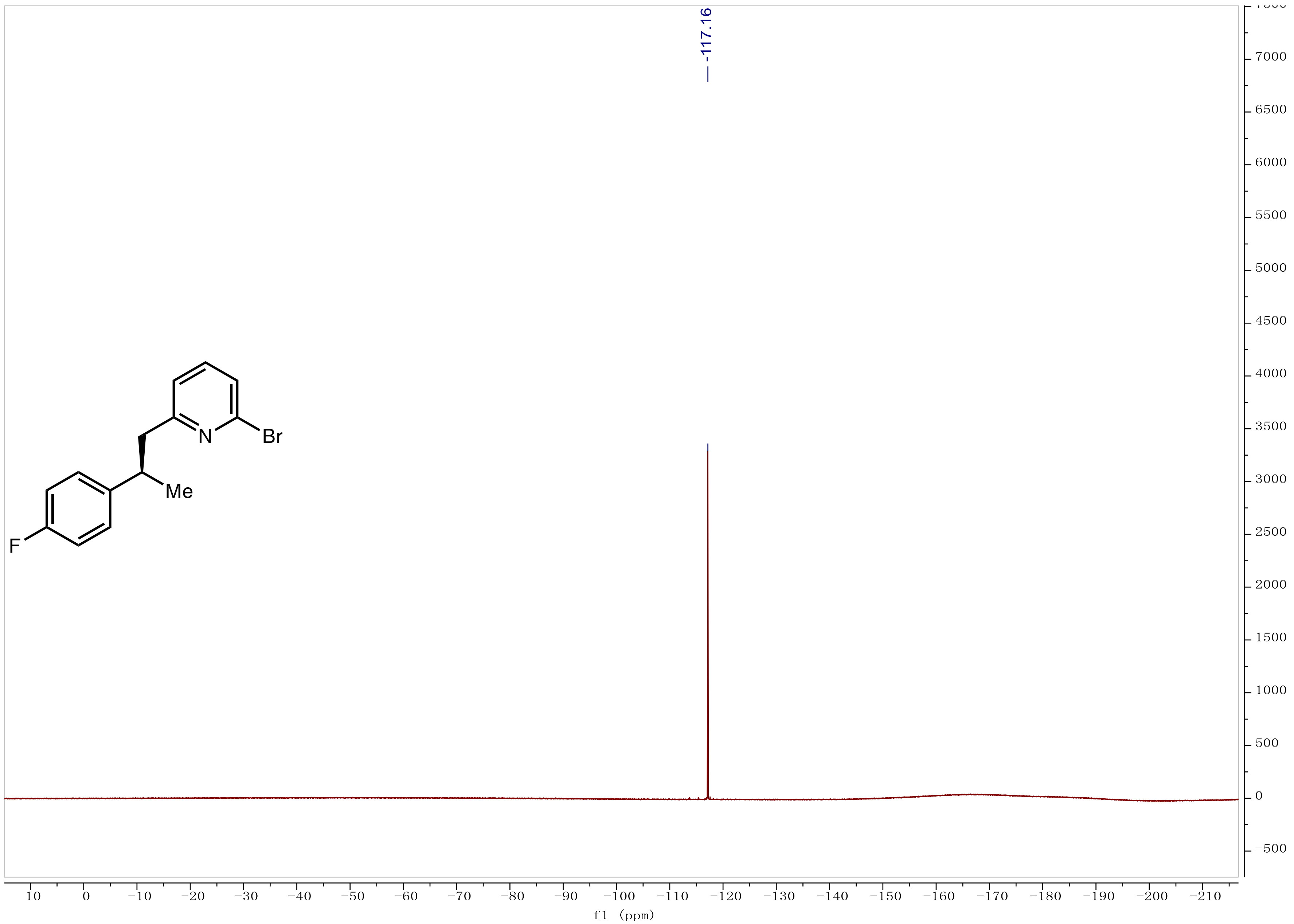

^{1}H NMR ($CDCl_3$, 400 MHz) spectrum of 2-Bromo-6-(2-(4-fluorophenyl)propyl)pyridine (3h)

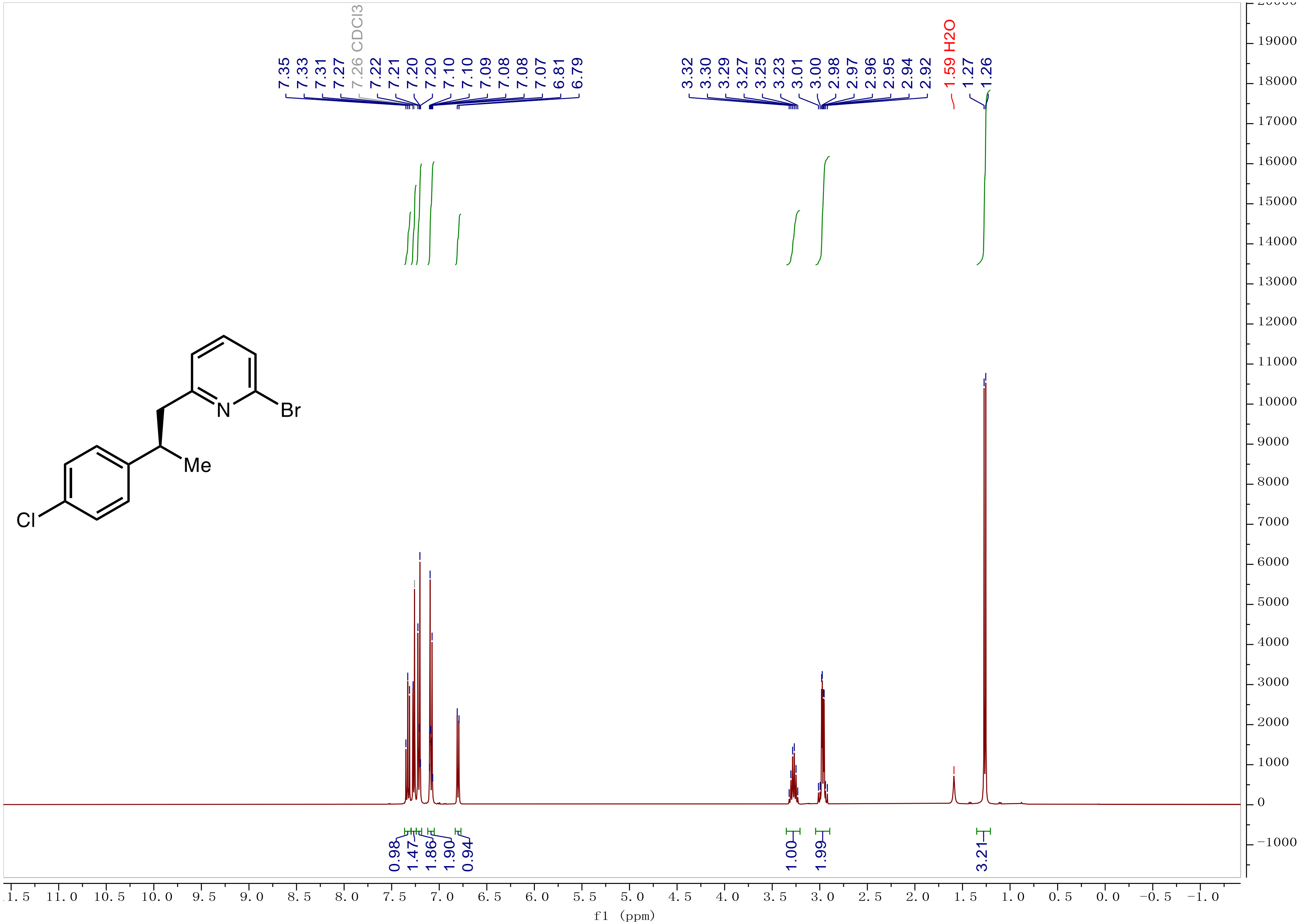

^{13}C NMR ($CDCl_3$, 100 MHz) spectrum of 2-Bromo-6-(2-(4-fluorophenyl)propyl)pyridine (3h)

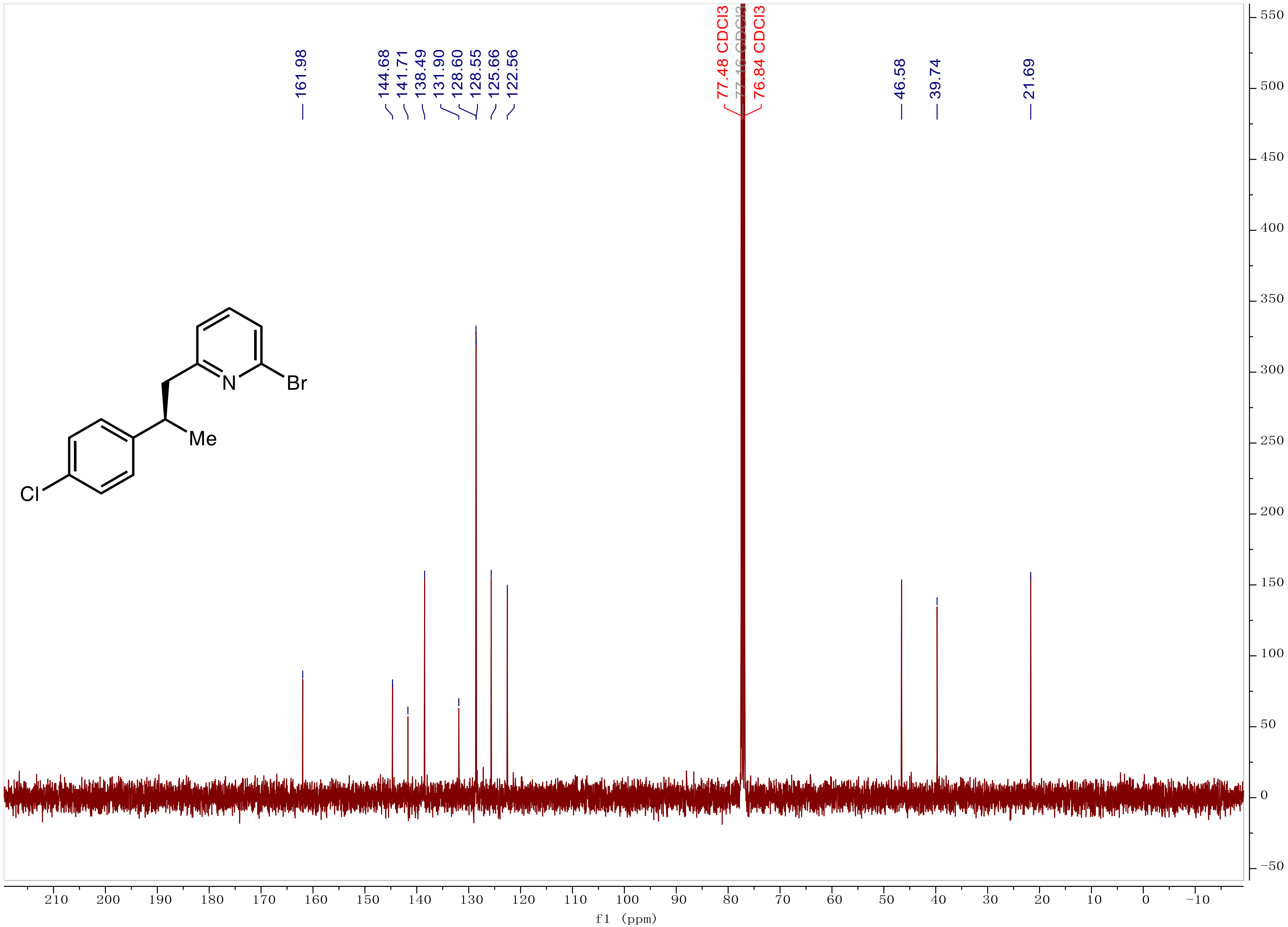

^{1}H NMR ($CDCl_3$, 400 MHz) spectrum of 2-Bromo-6-(2-(4-bromophenyl)propyl)pyridine (3i)

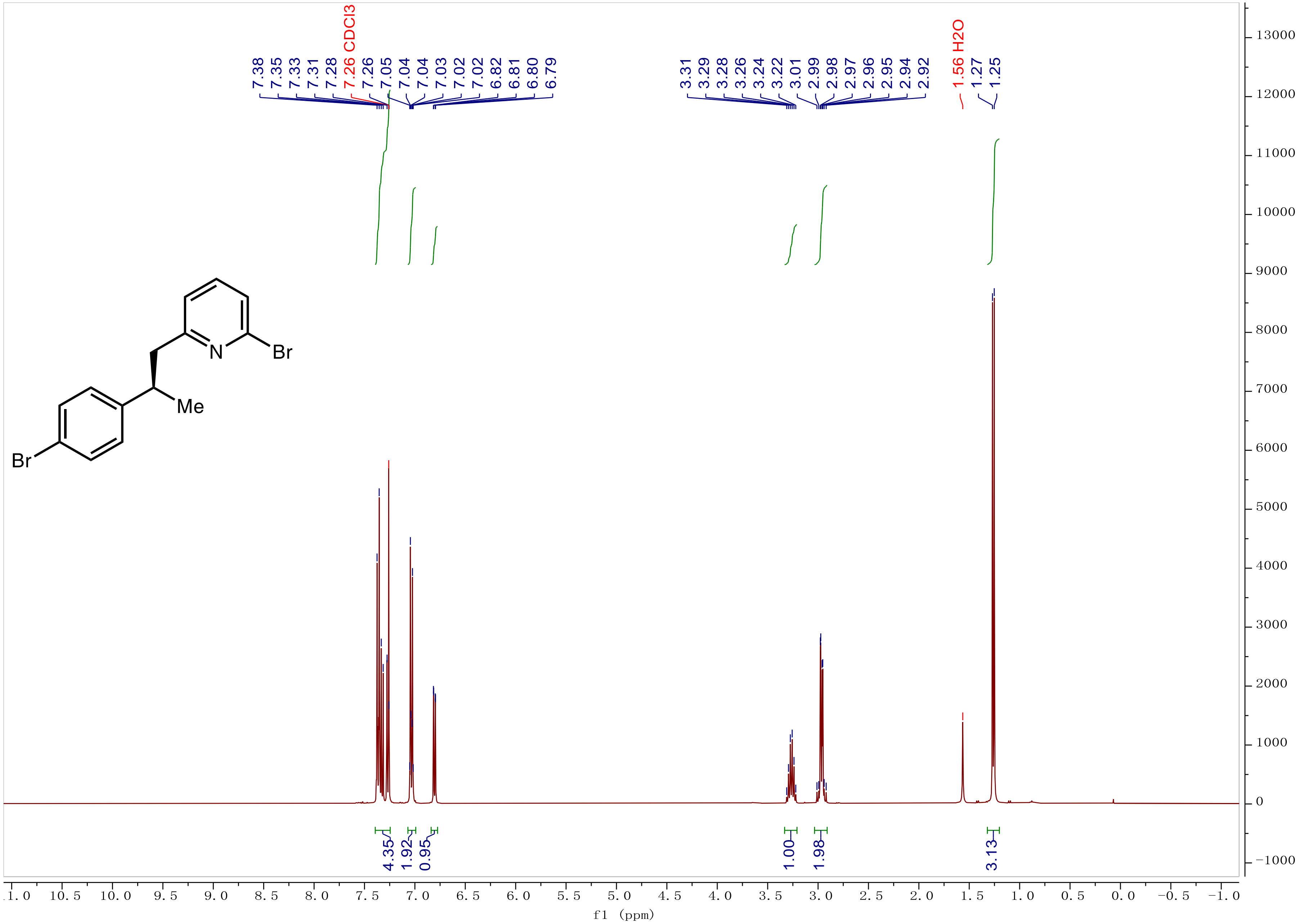

^{13}C NMR ($CDCl_3$, 100 MHz) spectrum of 2-Bromo-6-(2-(4-bromophenyl)propyl)pyridine (3i)

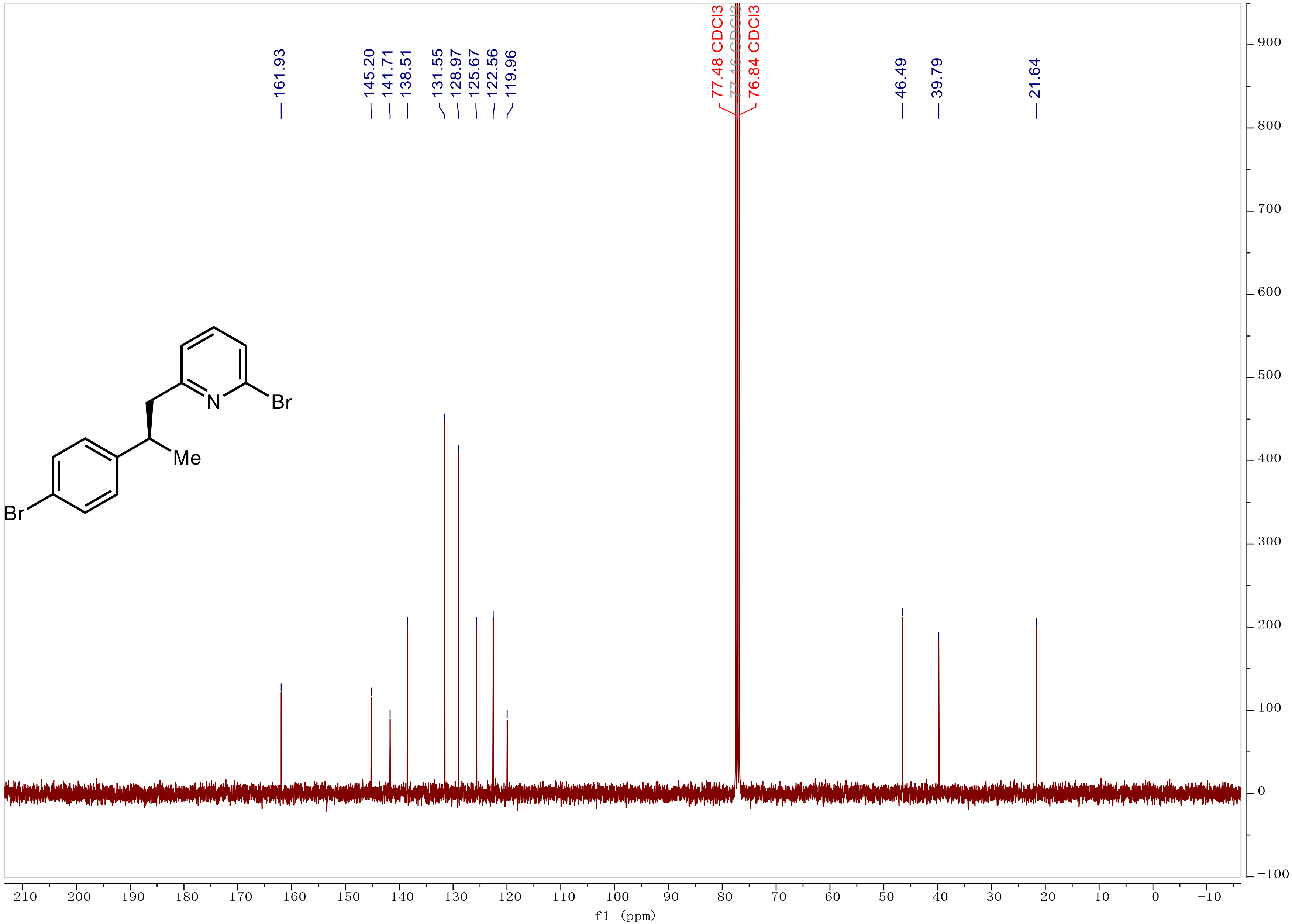

^{1}H NMR ($CDCl_3$, 400 MHz) spectrum of 2-Bromo-6-(2-(4-iodophenyl)propyl)pyridine (3j)

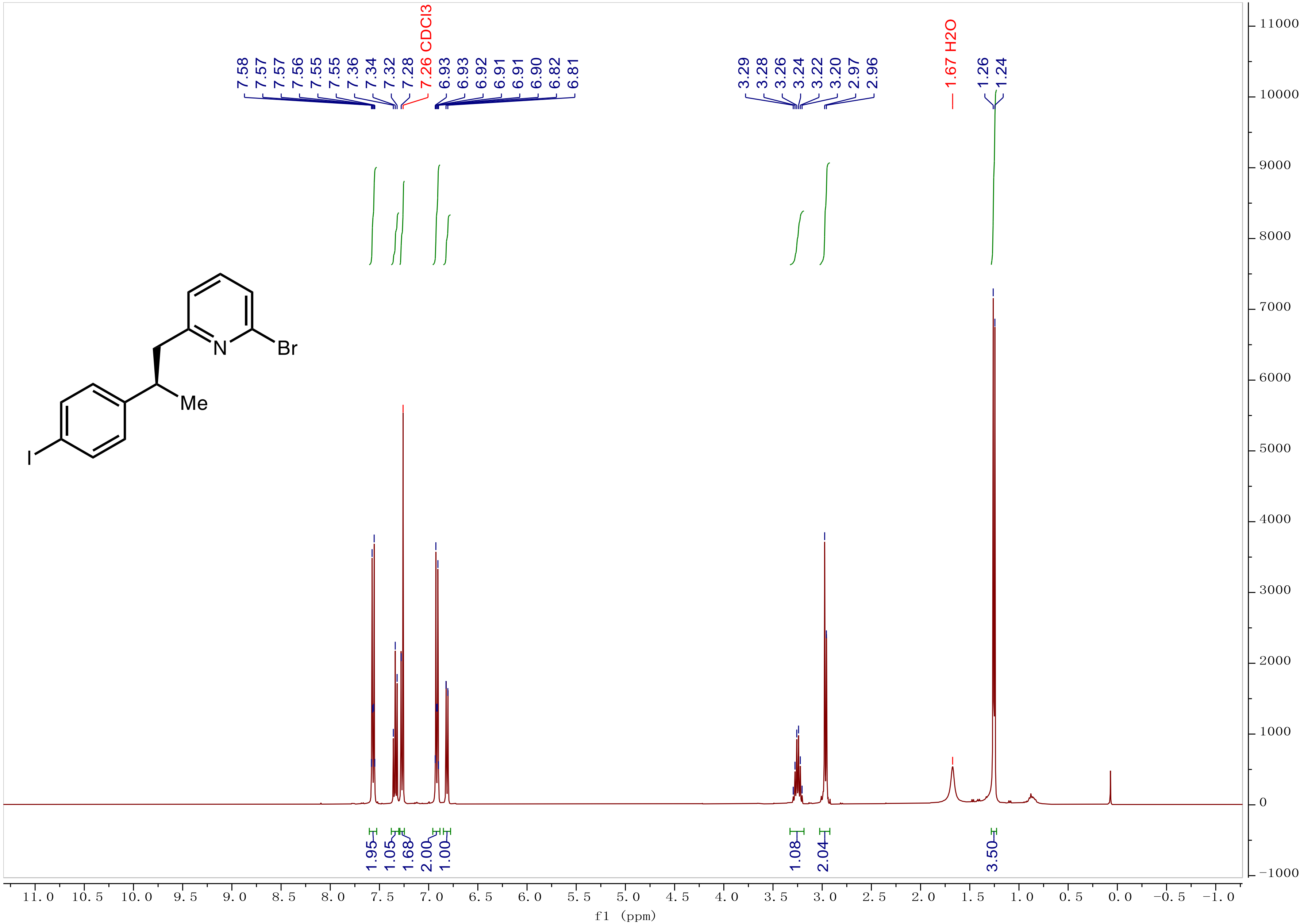

^{13}C NMR (CDCl$_3$, 100 MHz) spectrum of 2-Bromo-6-(2-(4-iodophenyl)propyl)pyridine (3j)

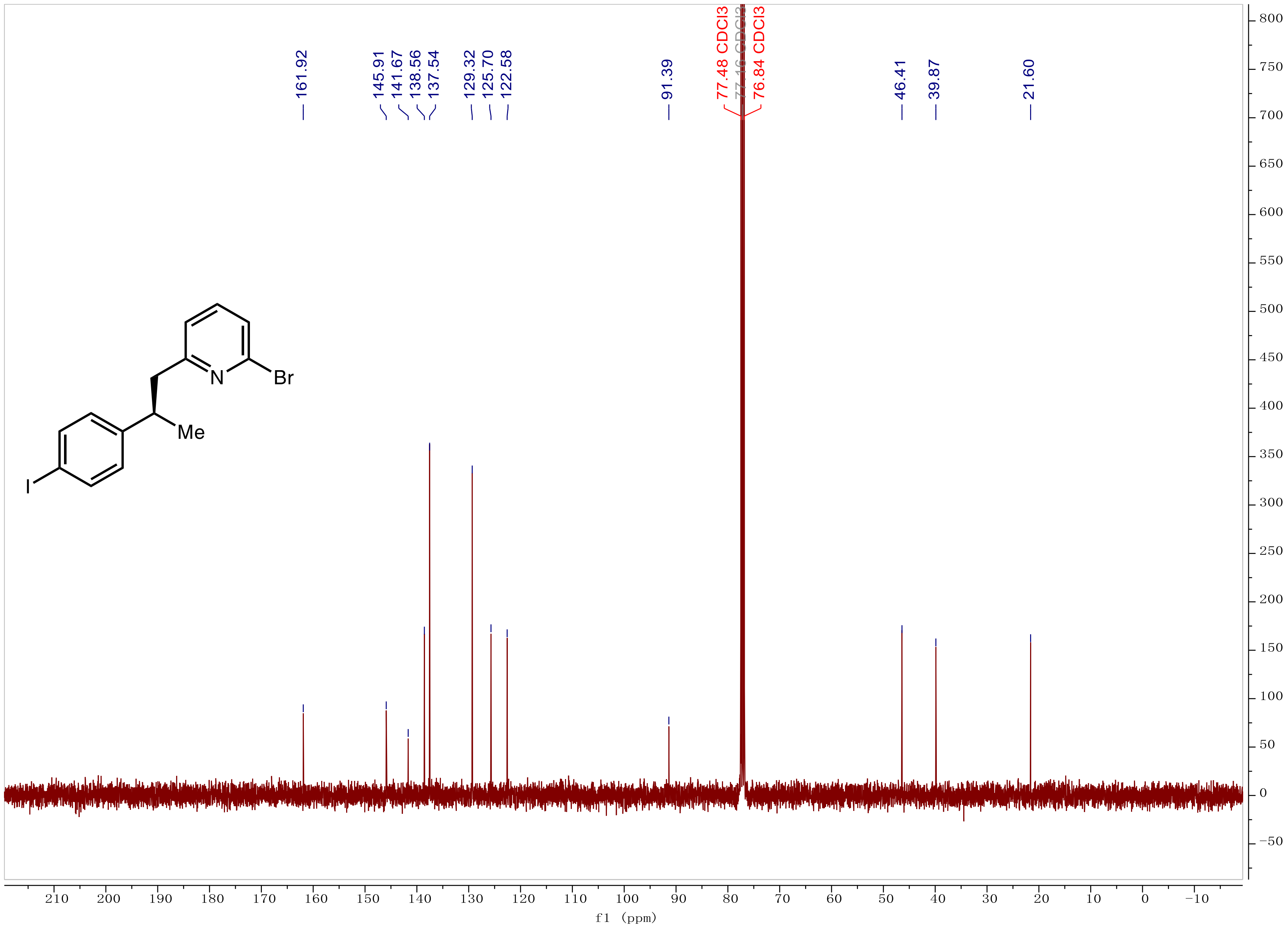

^{1}H NMR ($CDCl_3$, 400 MHz) spectrum of 2-Bromo-6-(2-(3-(trifluoromethyl)phenyl)propyl)pyridine (3k)

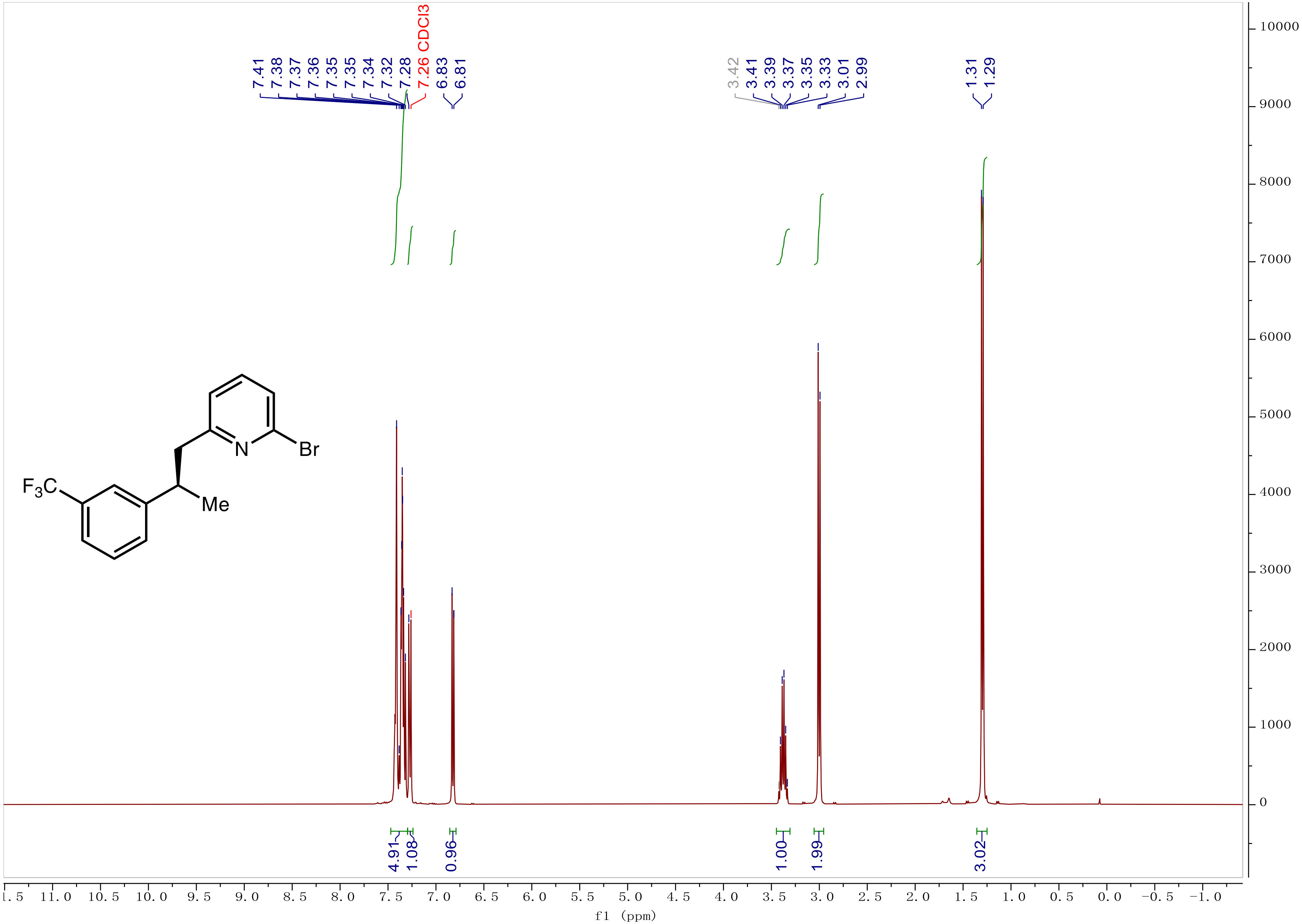

^{13}C NMR ($CDCl_3$, 100 MHz) spectrum of 2-Bromo-6-(2-(3-(trifluoromethyl)phenyl)propyl)pyridine (3k)

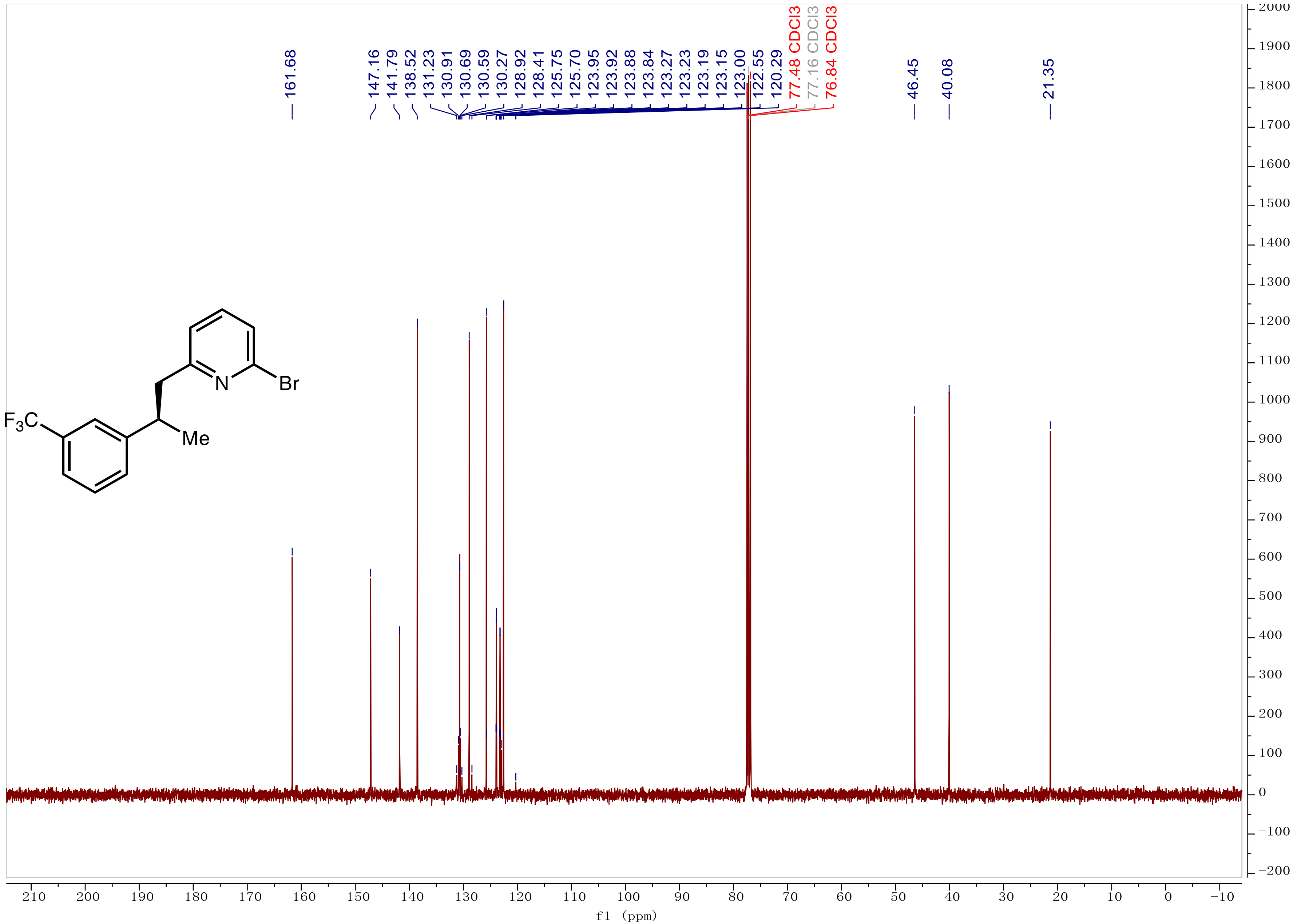

^{19}F NMR ($CDCl_3$, 376 MHz) spectrum of 2-Bromo-6-(2-(3-(trifluoromethyl)phenyl)propyl)pyridine (3k)

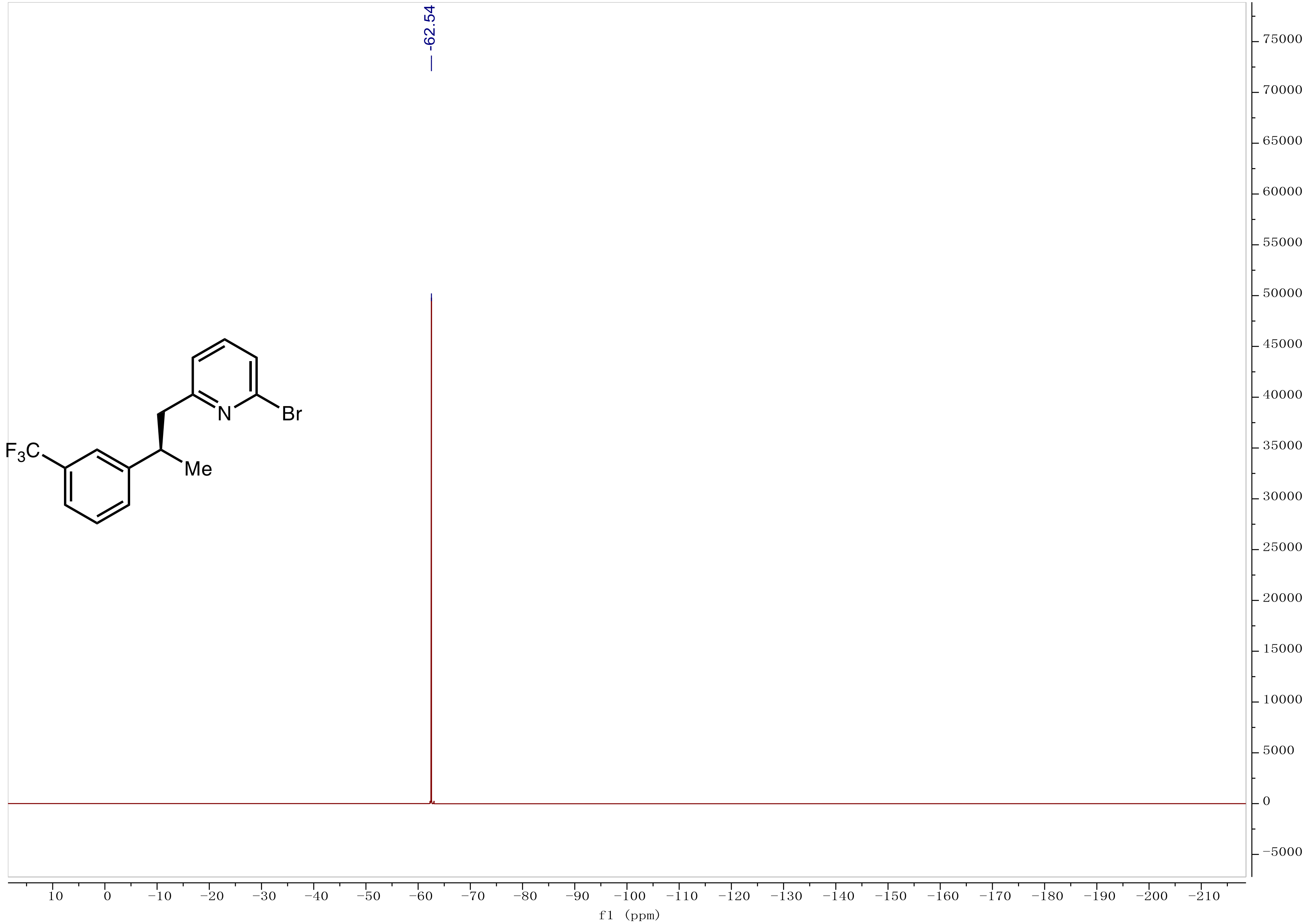

^{1}H NMR ($CDCl_3$, 400 MHz) spectrum of 5-(1-(6-bromopyridin-2-yl)propan-2-yl)-2-methoxypyridine (3l)

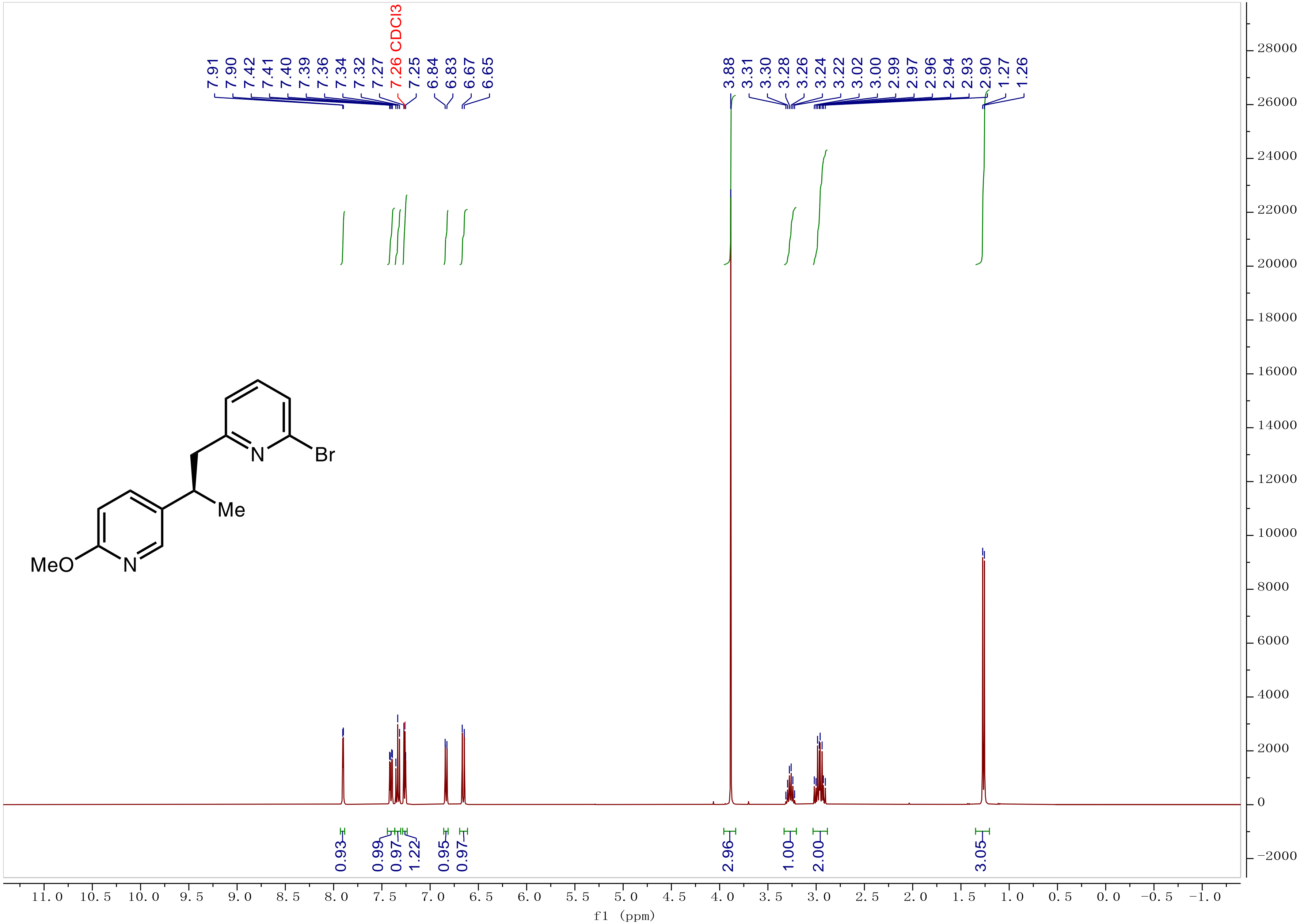

^{13}C NMR (CDCl₃, 100 MHz) spectrum of 5-(1-(6-bromopyridin-2-yl)propan-2-yl)-2-methoxypyridine (3l)

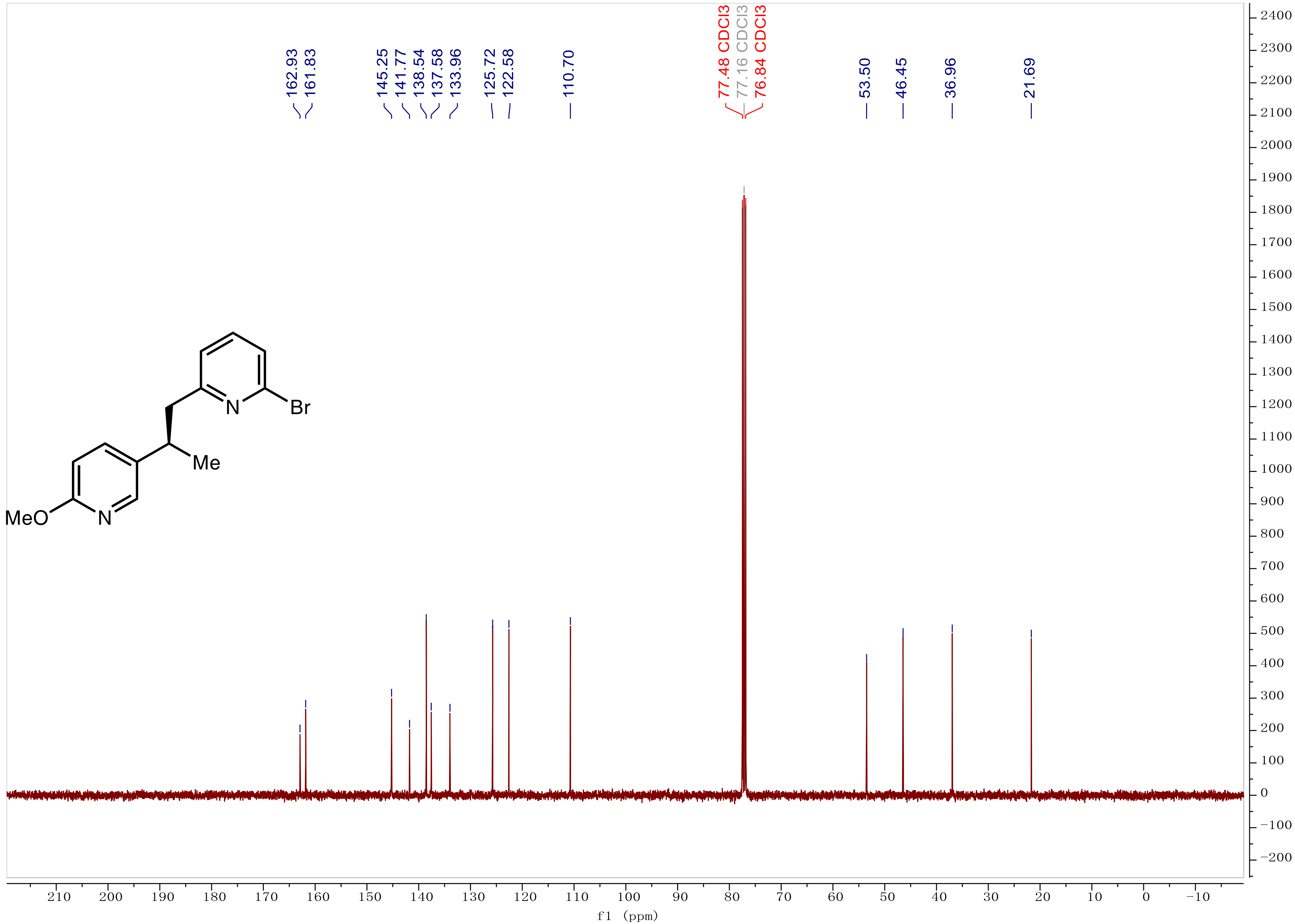

^{1}H NMR ($CDCl_3$, 400 MHz) spectrum of 2-Bromo-6-(2-(4-bromophenyl)propyl)pyridine (3m)

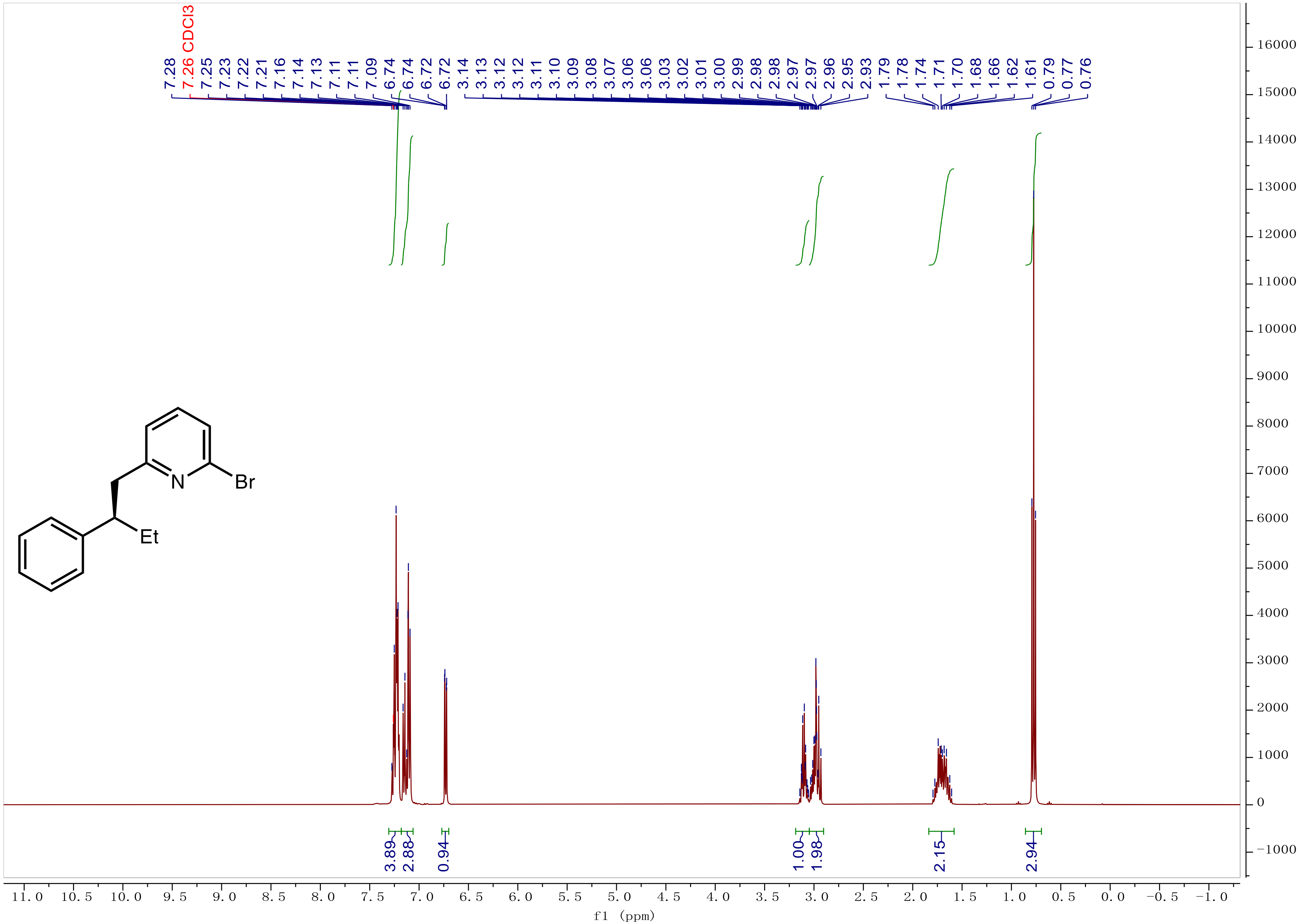

^{13}C NMR ($CDCl_3$, 100 MHz) spectrum of 2-Bromo-6-(2-(4-bromophenyl)propyl)pyridine (3m)

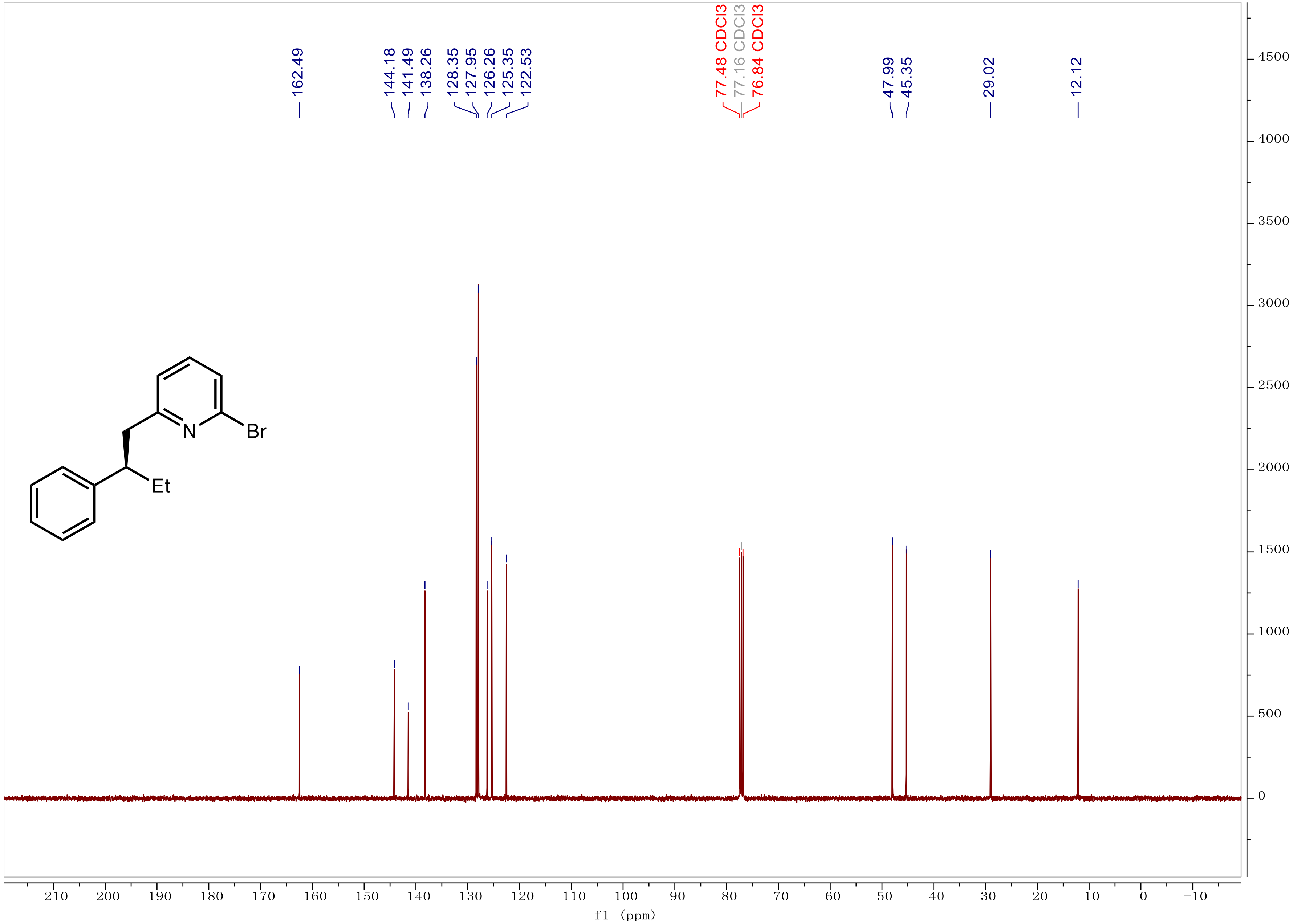

^{1}H NMR ($CDCl_3$, 400 MHz) spectrum of 2-Bromo-6-(2-phenylpentyl)pyridine (3n)

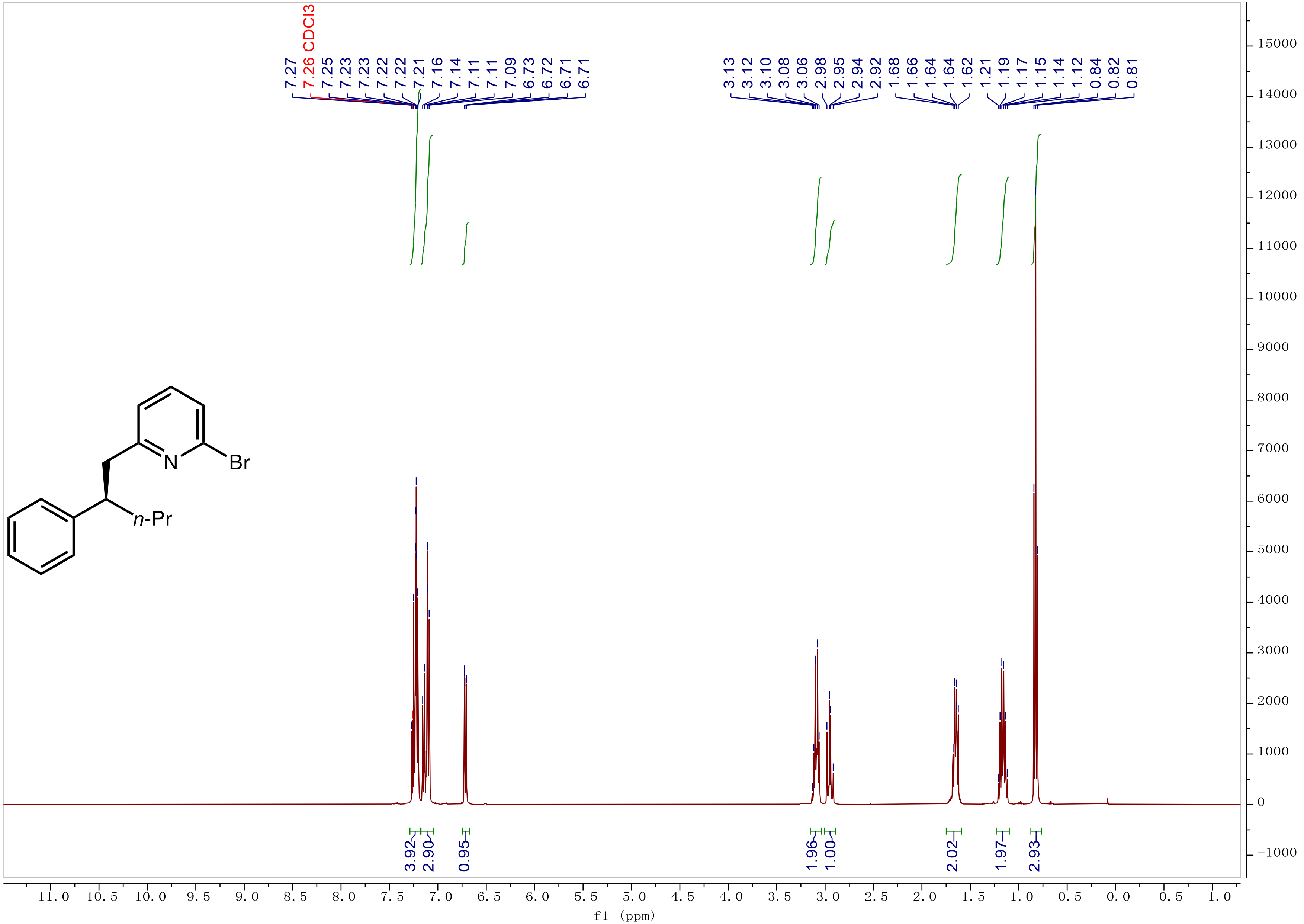

^{13}C NMR ($CDCl_3$, 100 MHz) spectrum of 2-Bromo-6-(2-phenylpentyl)pyridine (3n)

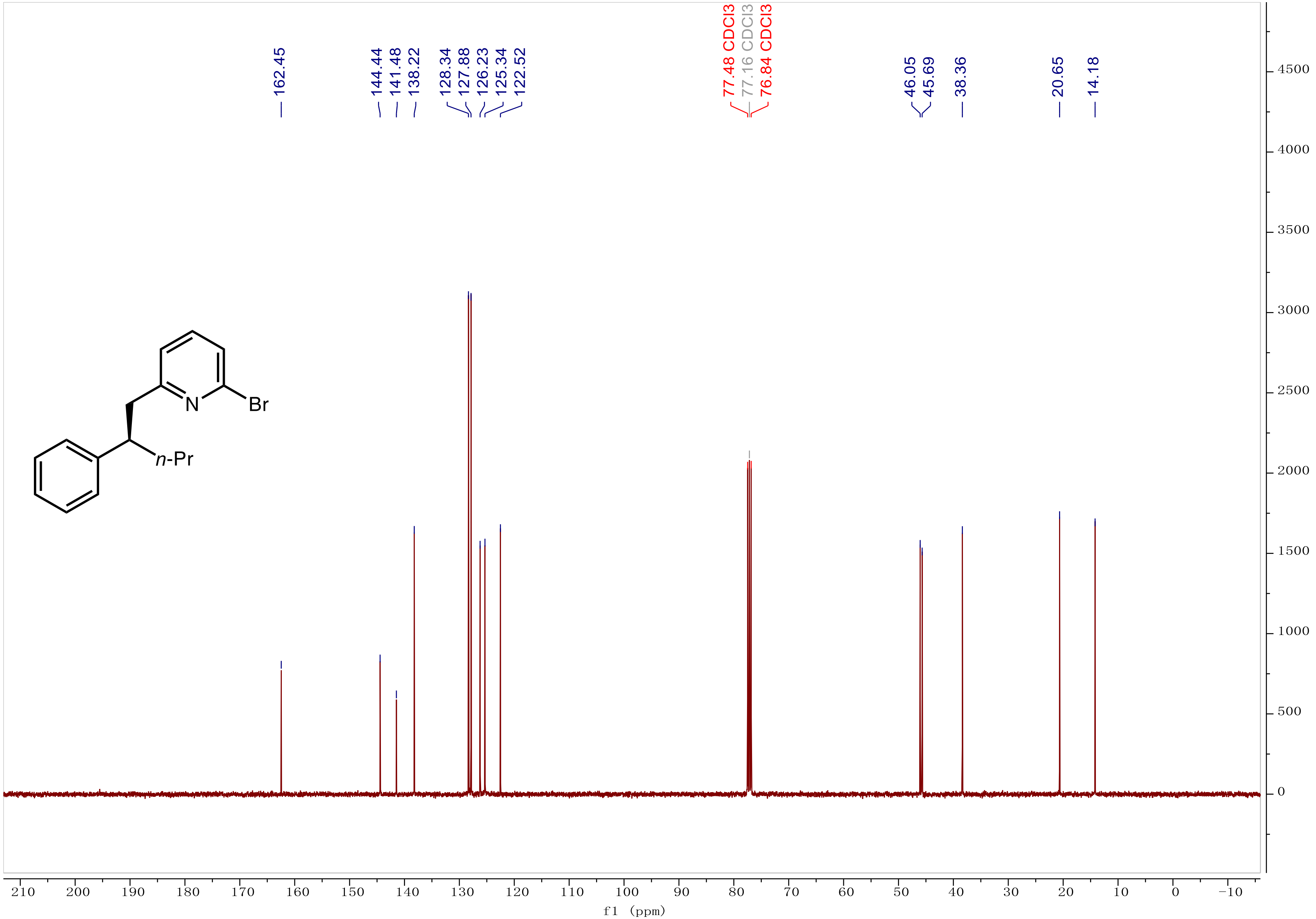

^{1}H NMR (CDCl$_3$, 400 MHz) spectrum of 2-Bromo-6-((2,3-dihydro-1H-inden-1-yl)methyl)pyridine (3o)

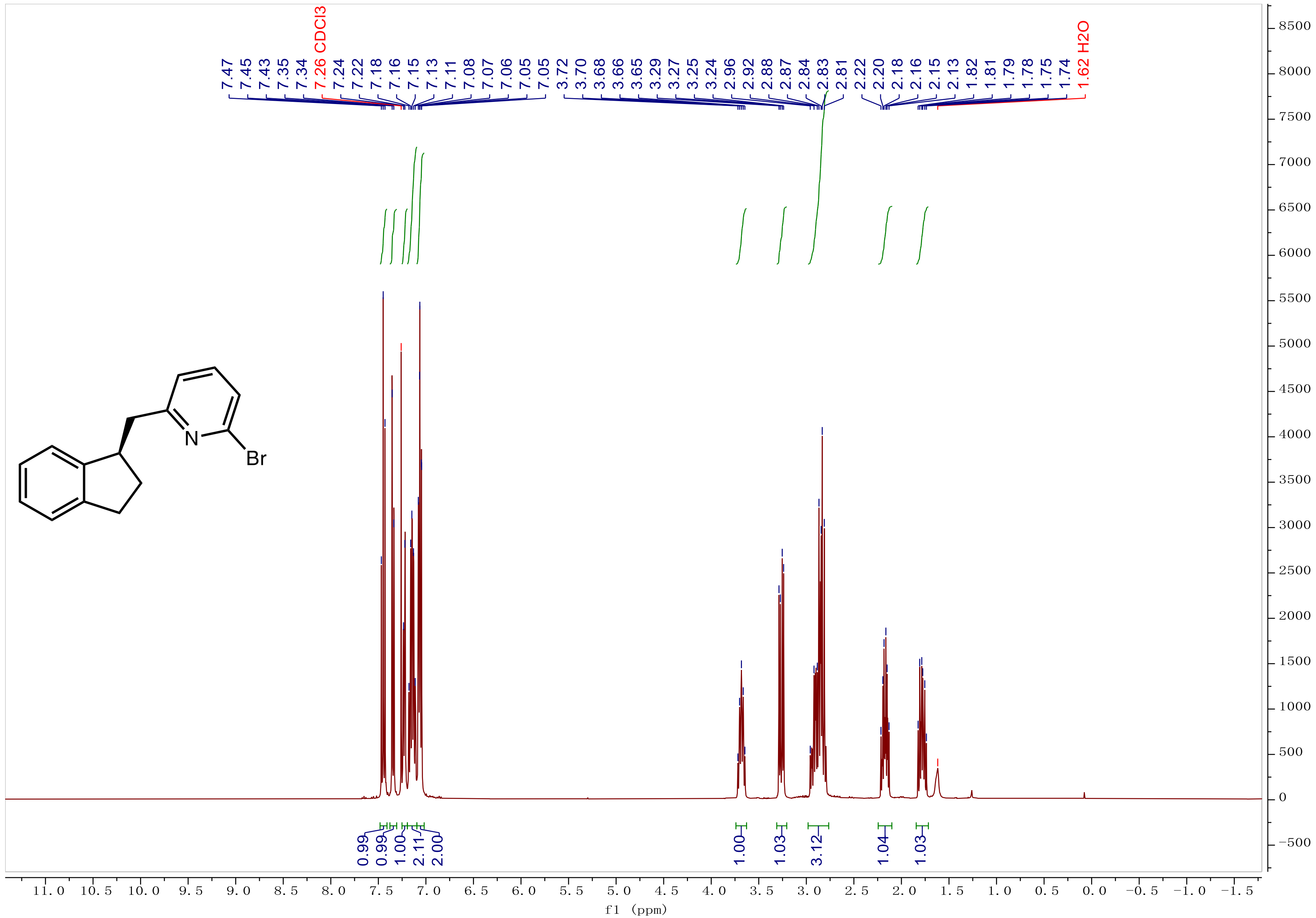

^{13}C NMR ($CDCl_3$, 100 MHz) spectrum of 2-Bromo-6-((2,3-dihydro-1H-inden-1-yl)methyl)pyridine (3o)

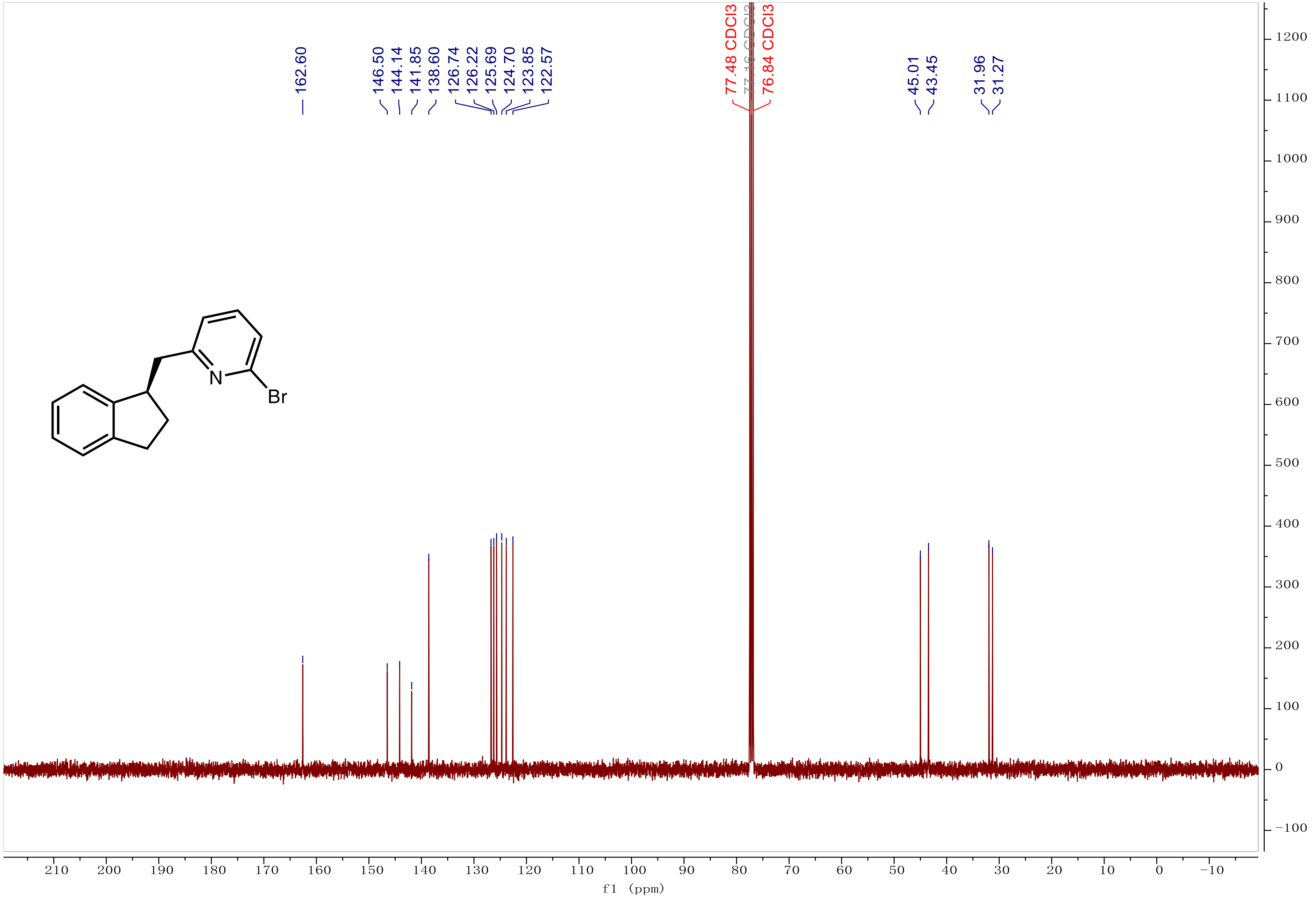

^{1}H NMR (CDCl$_3$, 400 MHz) spectrum of 2-Bromo-6-((1,2,3,4-tetrahydronaphthalen-1-yl)methyl)pyridine (3p)

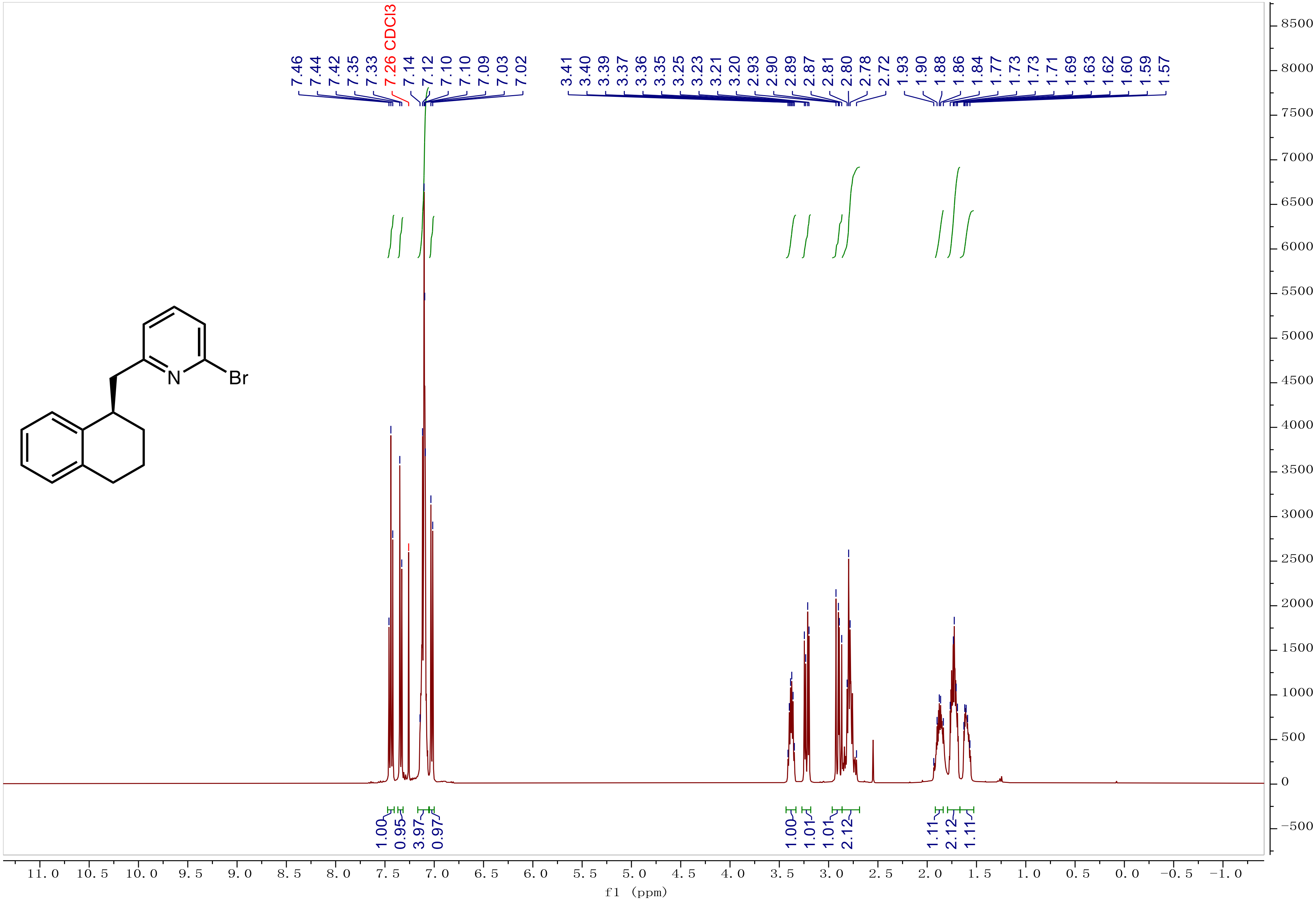

^{13}C NMR ($CDCl_3$, 100 MHz) spectrum of 2-Bromo-6-((1,2,3,4-tetrahydronaphthalen-1-yl)methyl)pyridine (3p)

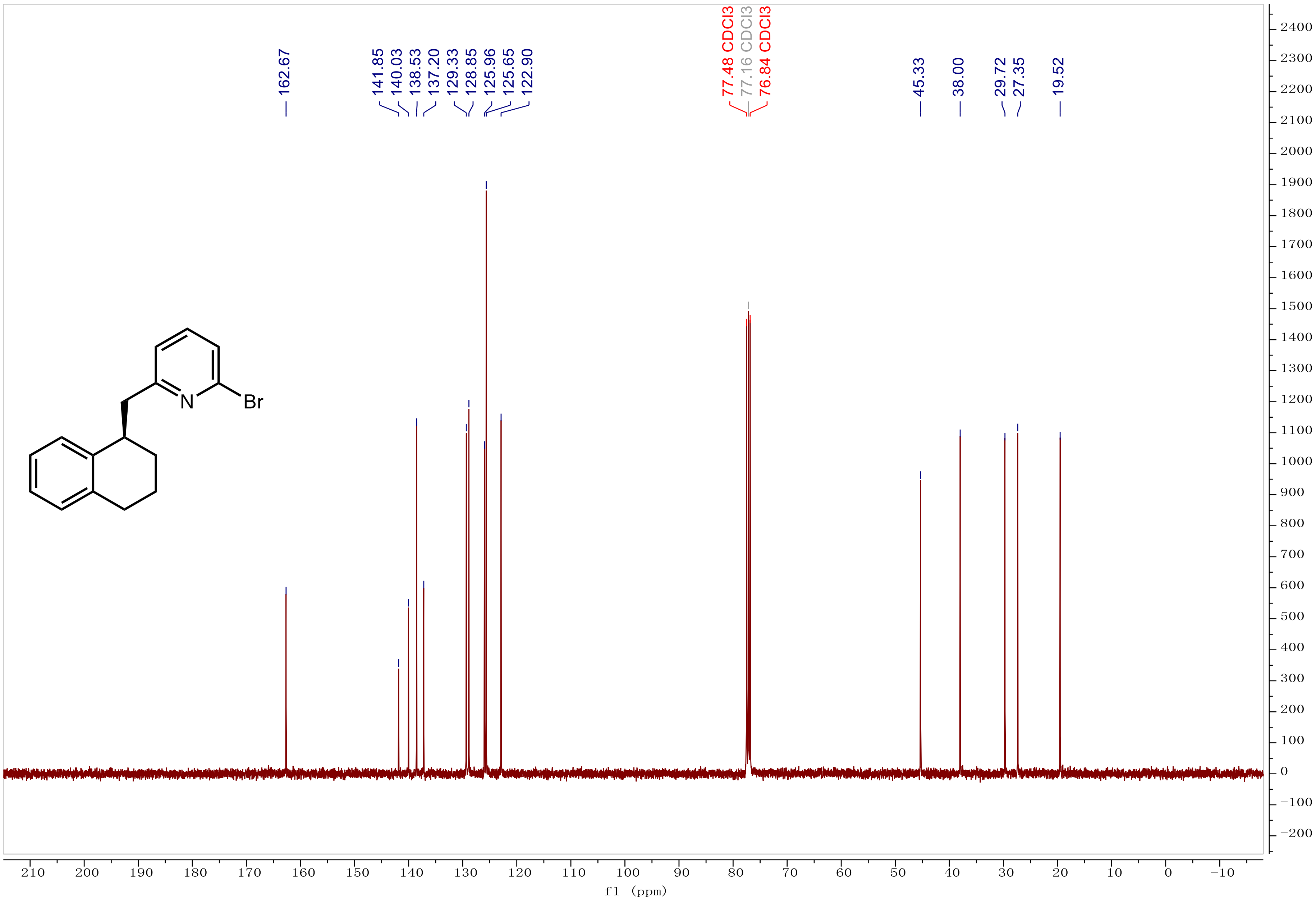

^{1}H NMR ($CDCl_3$, 400 MHz) spectrum of 2-Bromo-6-((7-fluoro-1,2,3,4-tetrahydronaphthalen-1-yl)methyl)pyridine (3q)

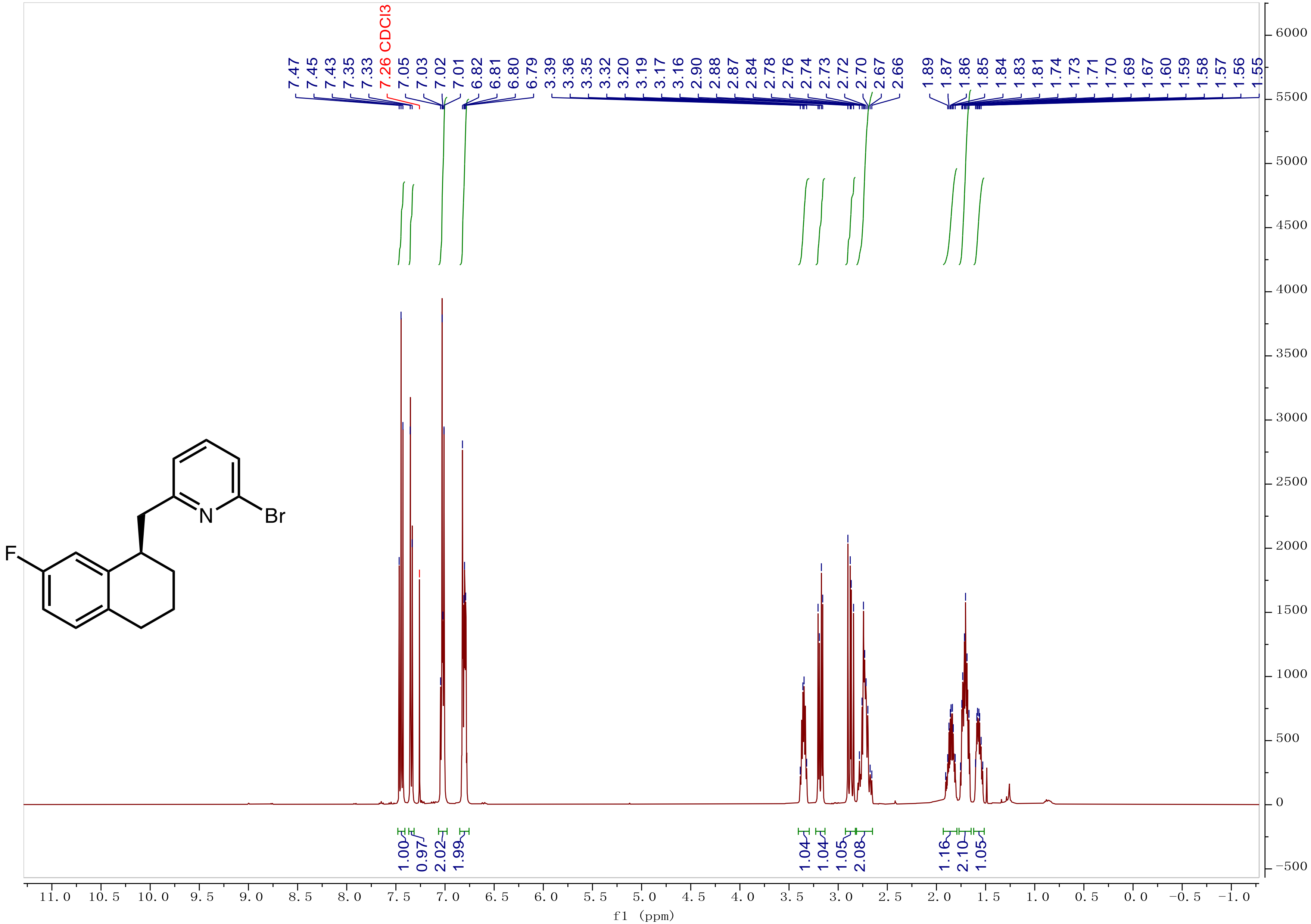

^{13}C NMR ($CDCl_3$, 100 MHz) spectrum of 2-Bromo-6-((7-fluoro-1,2,3,4-tetrahydronaphthalen-1-yl)methyl)pyridine (3q)

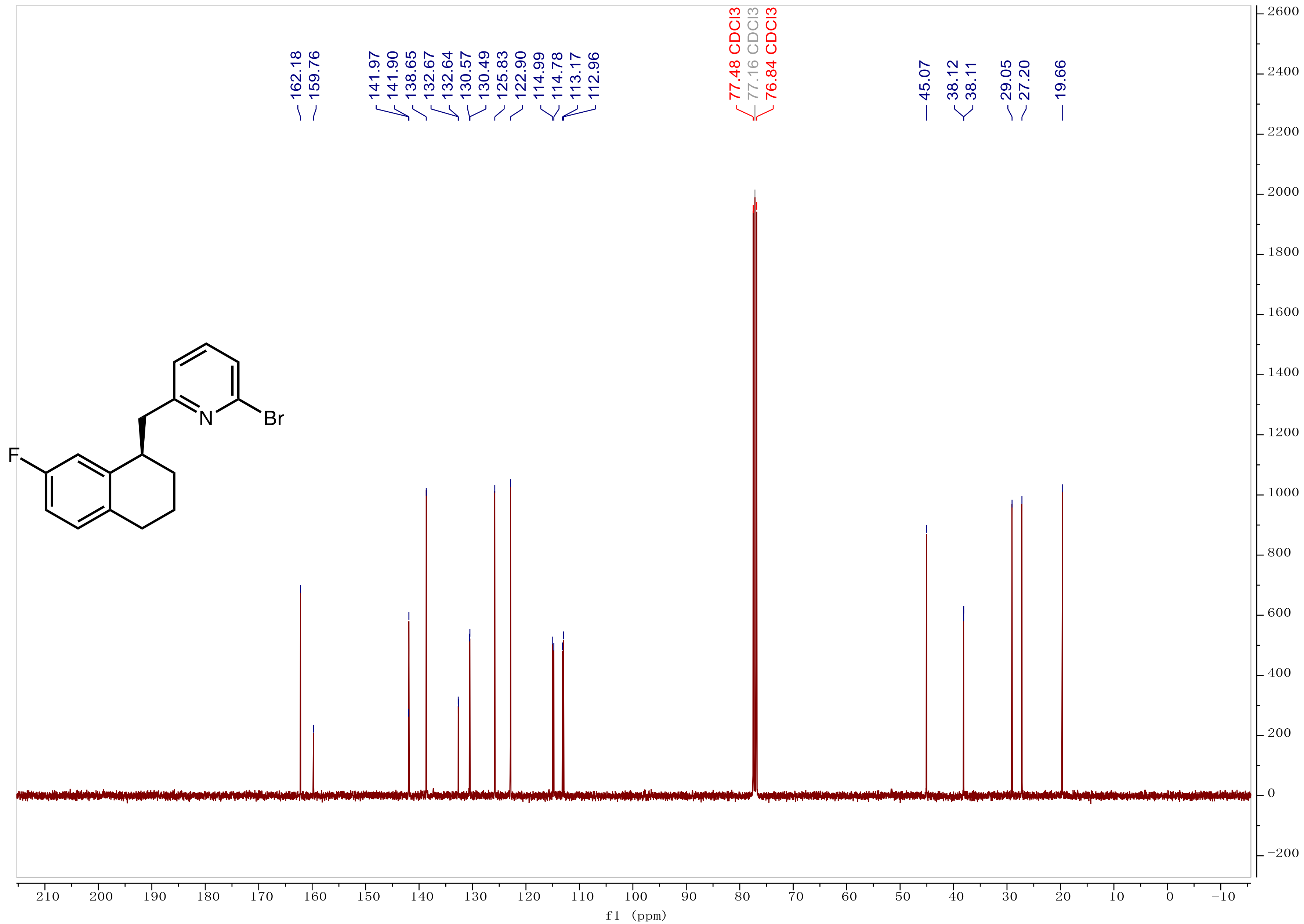

^{19}F NMR ($CDCl_3$, 376 MHz) spectrum of 2-Bromo-6-((7-fluoro-1,2,3,4-tetrahydronaphthalen-1-yl)methyl)pyridine (3q)

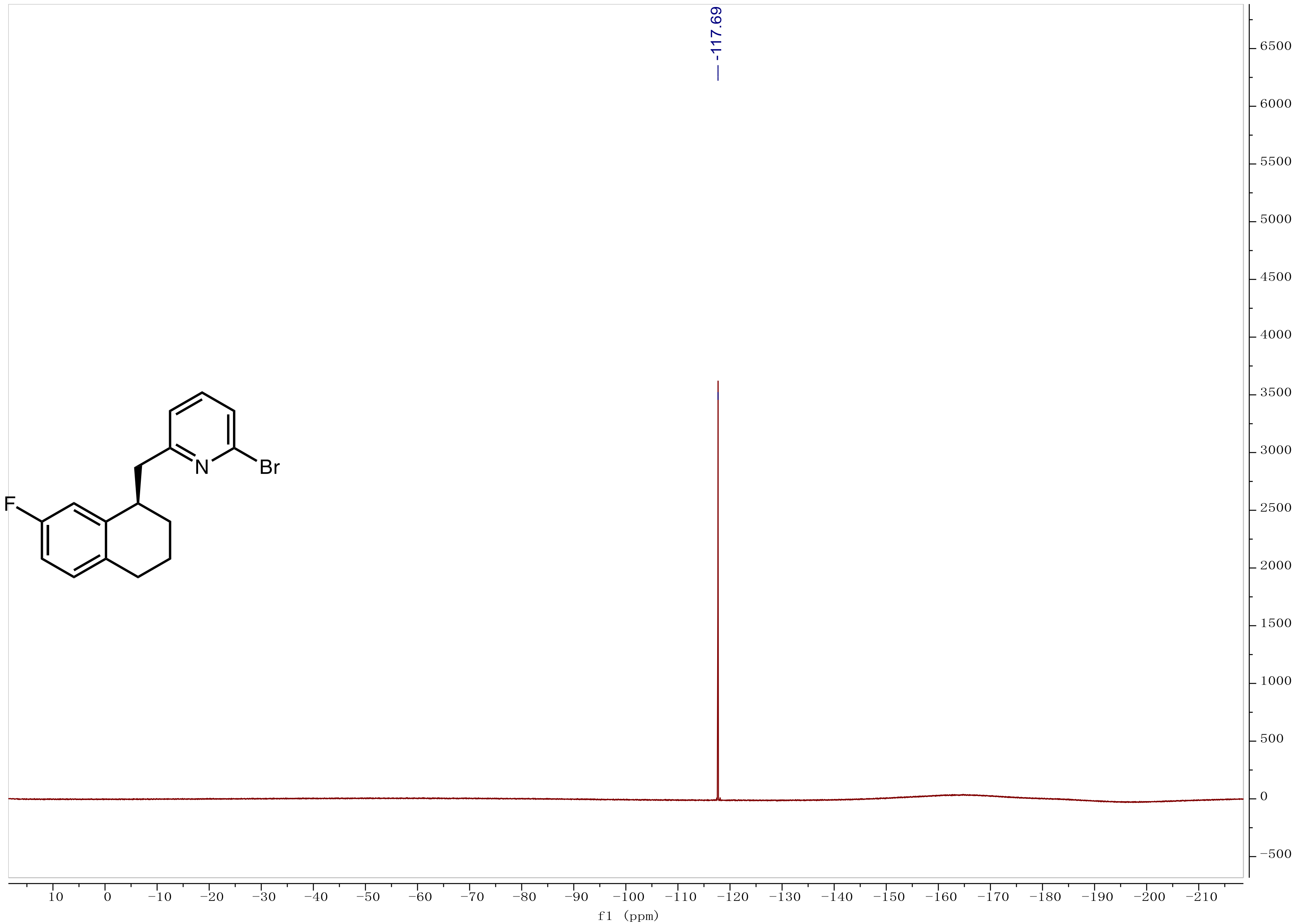

^{1}H NMR ($CDCl_3$, 400 MHz) spectrum of 2-Bromo-6-(chroman-4-ylmethyl)pyridine (3r)

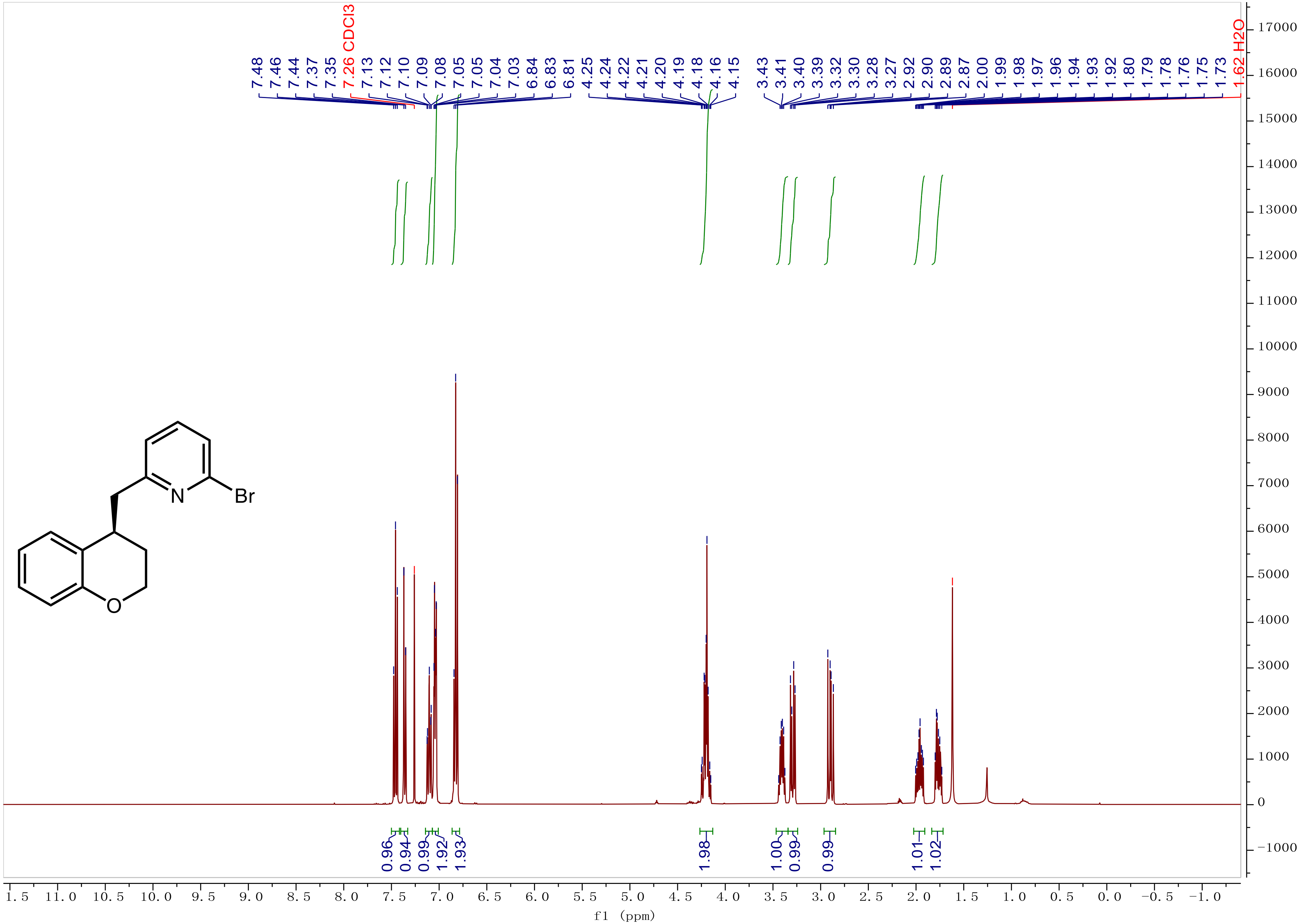

^{13}C NMR ($CDCl_3$, 100 MHz) spectrum of 2-Bromo-6-(chroman-4-ylmethyl)pyridine (3r)

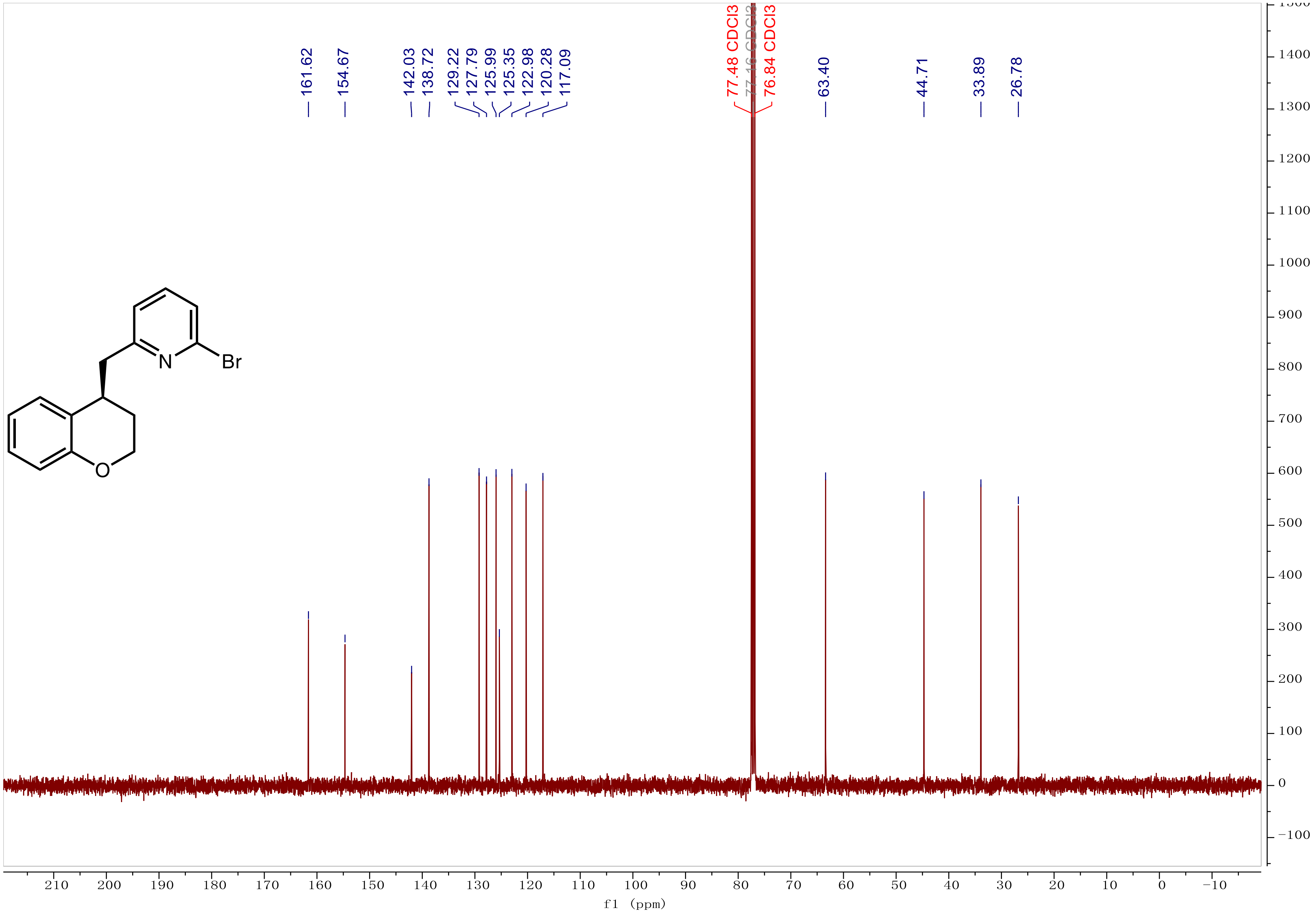

^{1}H NMR ($CDCl_3$, 400 MHz) spectrum of 2-Chloro-6-(2-(p-tolyl)propyl)pyridine (3s)

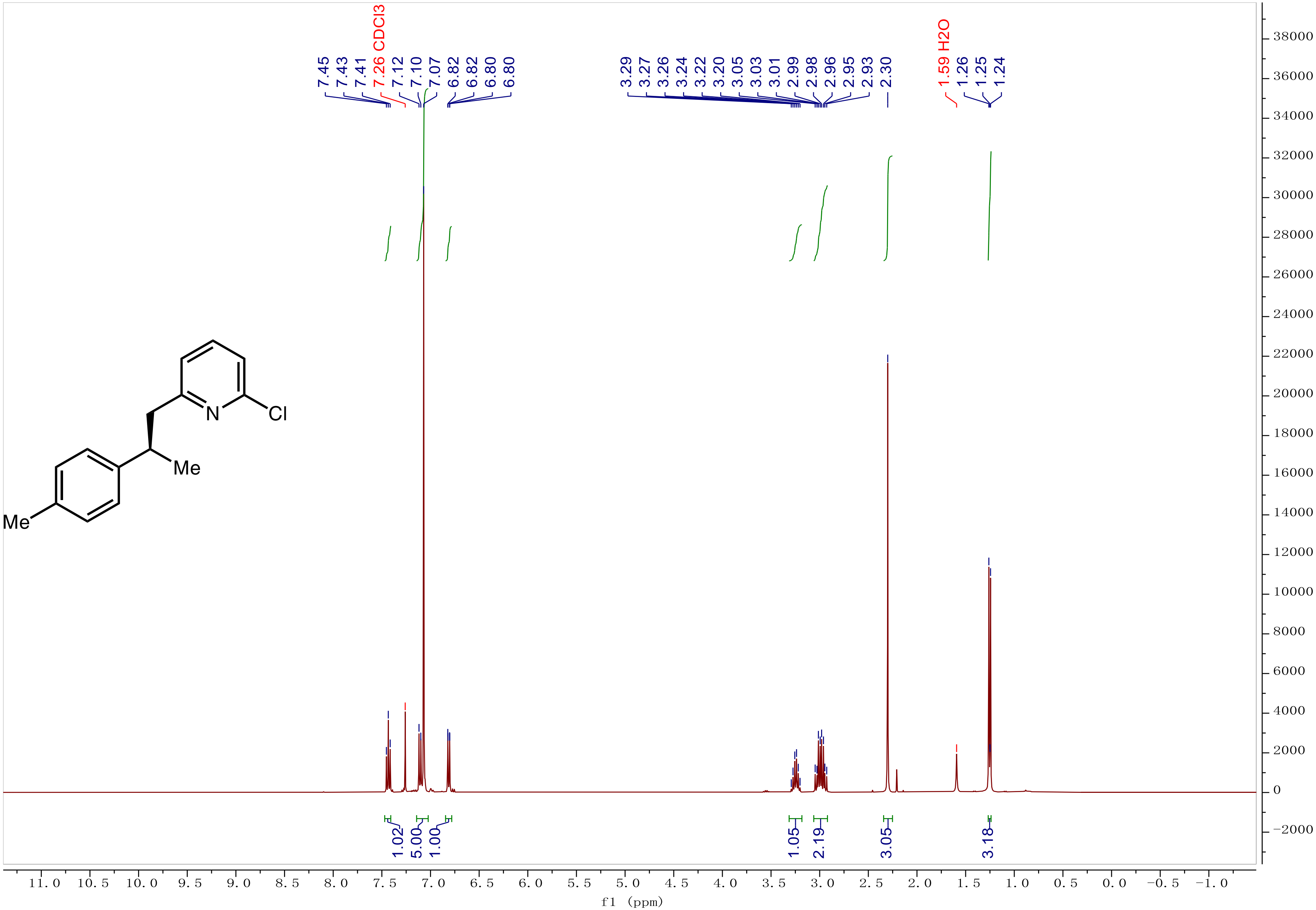

^{13}C NMR ($CDCl_3$, 100 MHz) spectrum of 2-Chloro-6-(2-(p-tolyl)propyl)pyridine (3s)

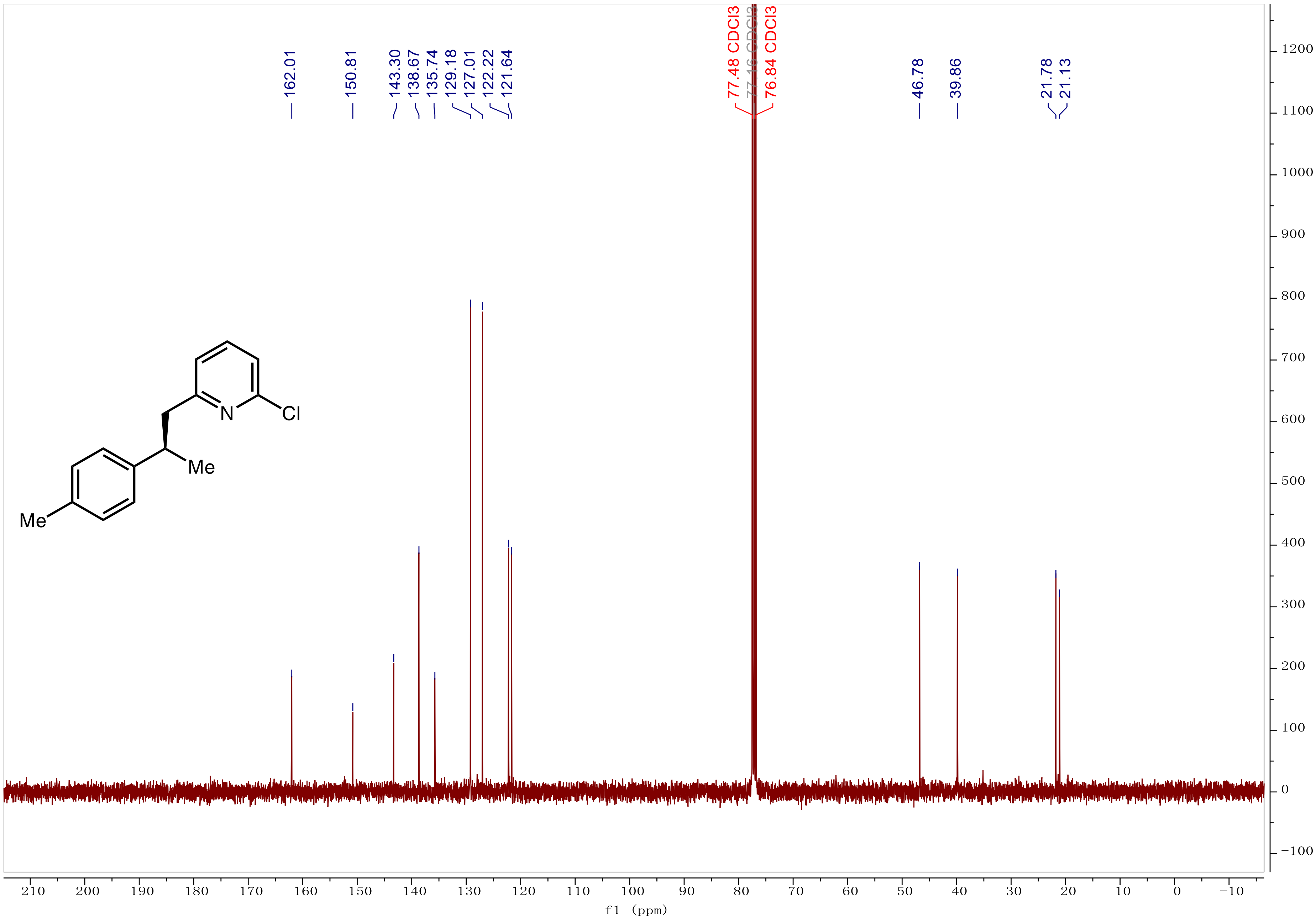

^{1}H NMR ($CDCl_3$, 400 MHz) spectrum of 2-Iodo-6-(2-(p-tolyl)propyl)pyridine (3t)

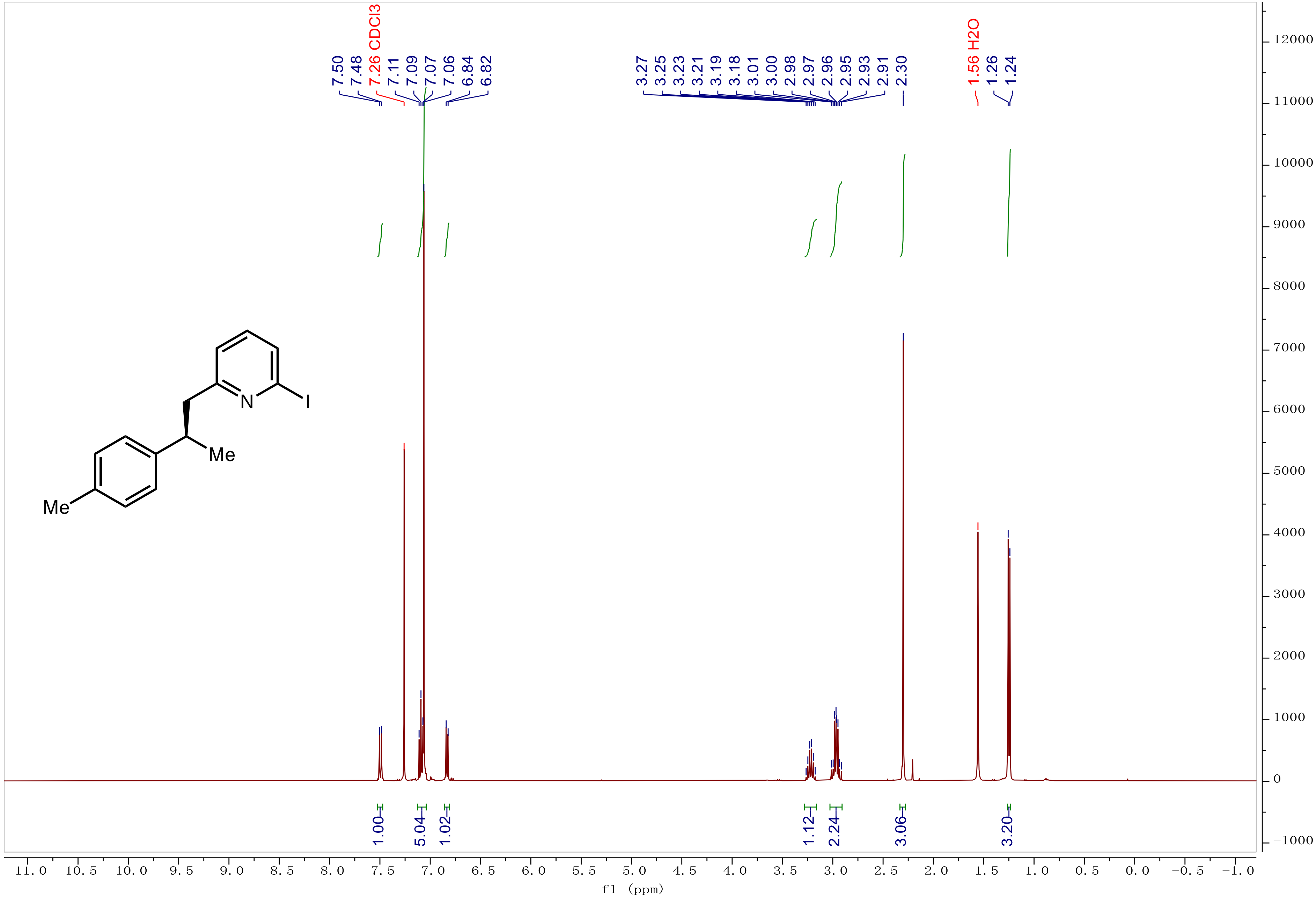

^{13}C NMR ($CDCl_3$, 100 MHz) spectrum of 2-Iodo-6-(2-(p-tolyl)propyl)pyridine (3t)

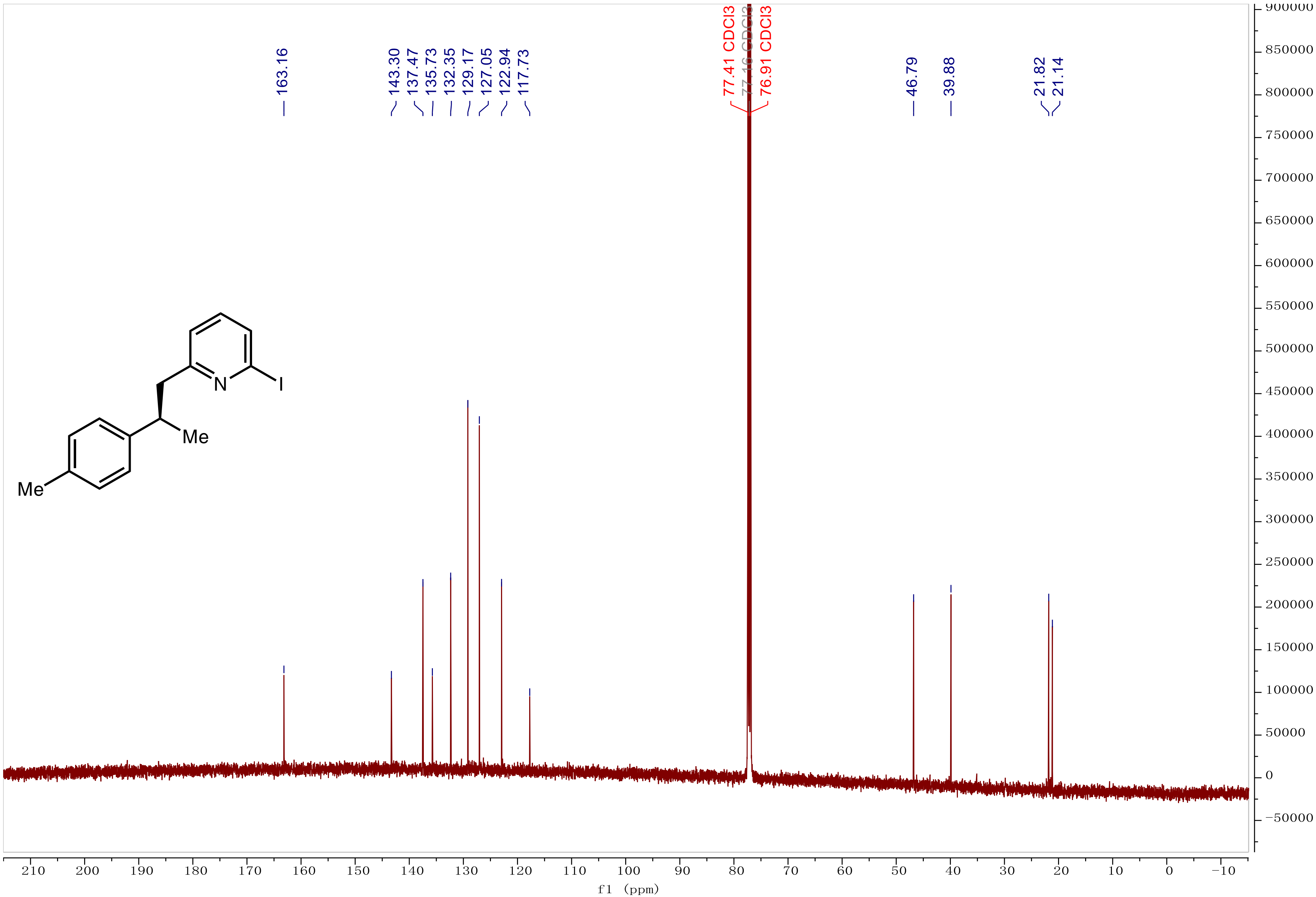

^{1}H NMR ($CDCl_3$, 400 MHz) spectrum of 2-methoxy-6-(2-(p-tolyl)propyl)pyridine (3u)

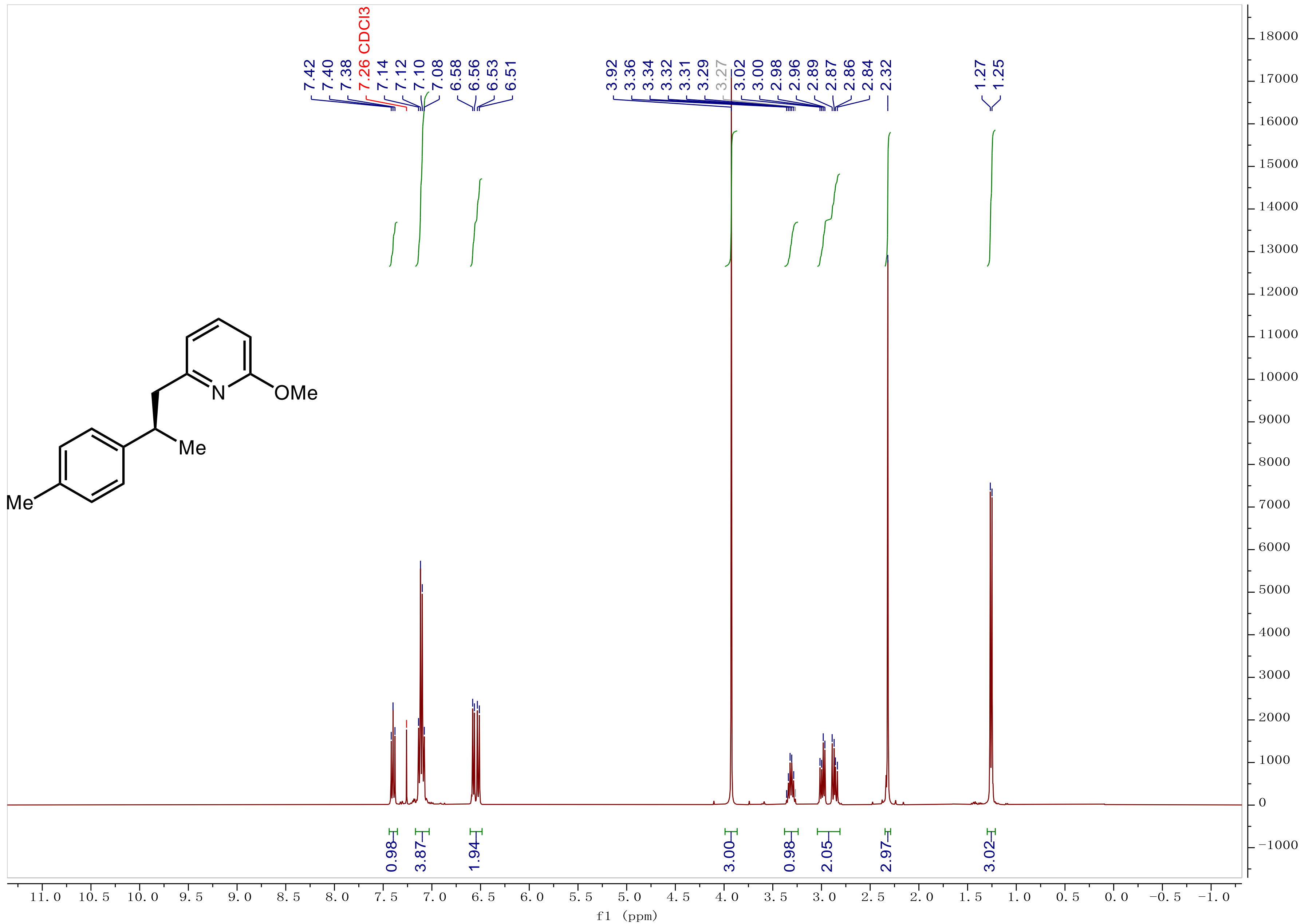

^{13}C NMR ($CDCl_3$, 100 MHz) spectrum of 2-methoxy-6-(2-(p-tolyl)propyl)pyridine (3u)

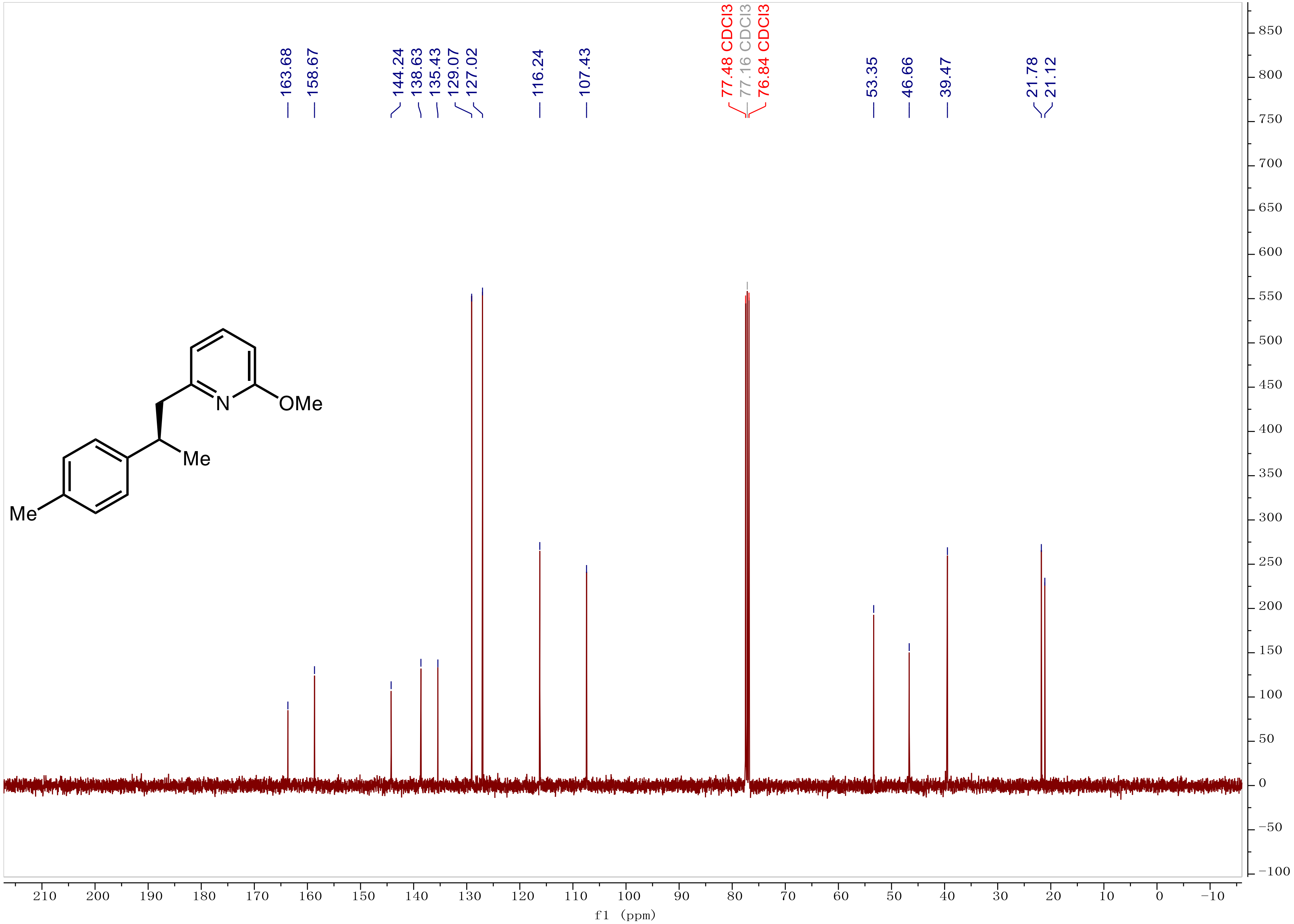

^{1}H NMR ($CDCl_3$, 400 MHz) spectrum of 2-(Methylthio)-4-(2-(p-tolyl)propyl)pyrimidine (3v)

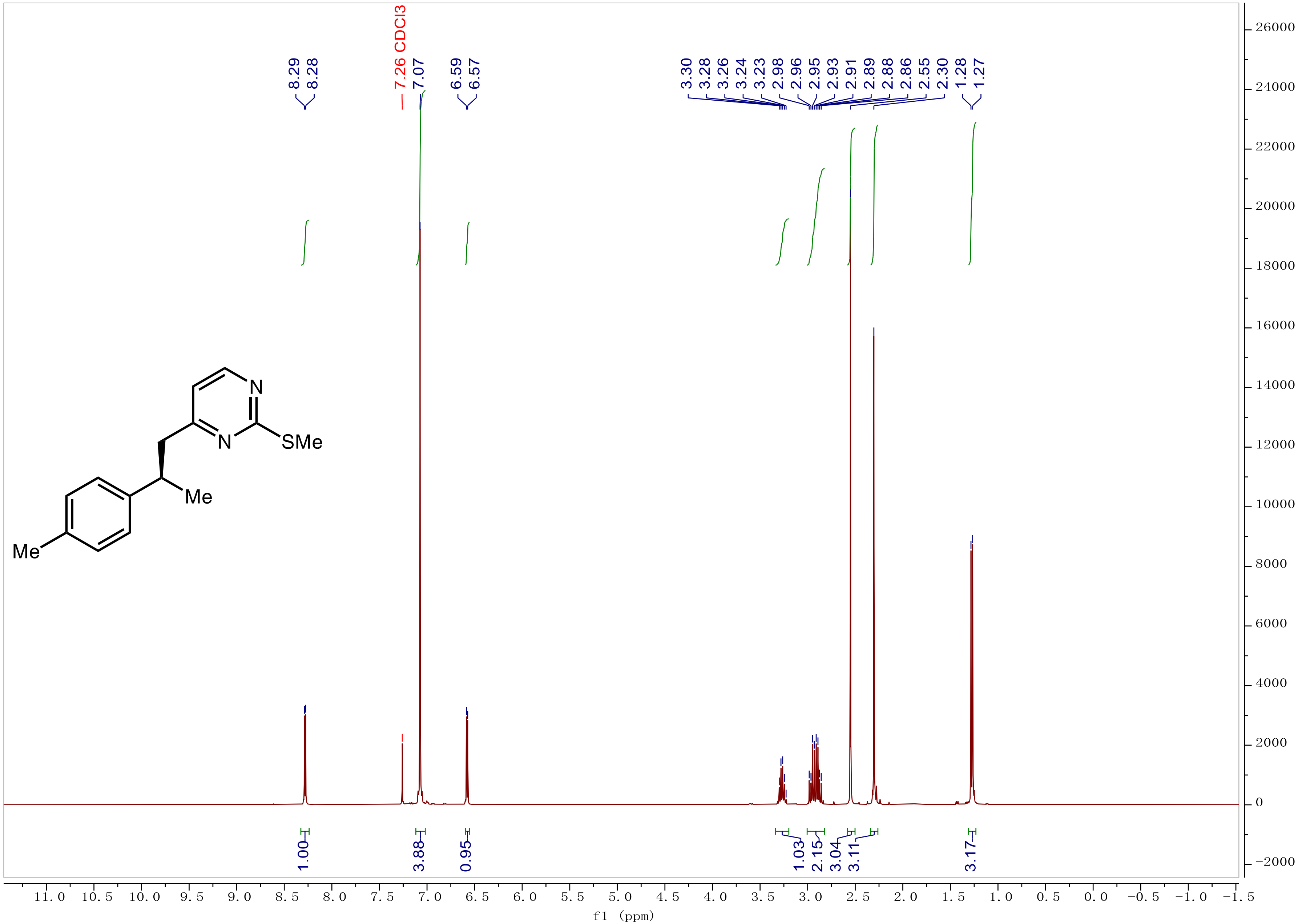

^{13}C NMR ($CDCl_3$, 100 MHz) spectrum of 2-(Methylthio)-4-(2-(p-tolyl)propyl)pyrimidine (3v)

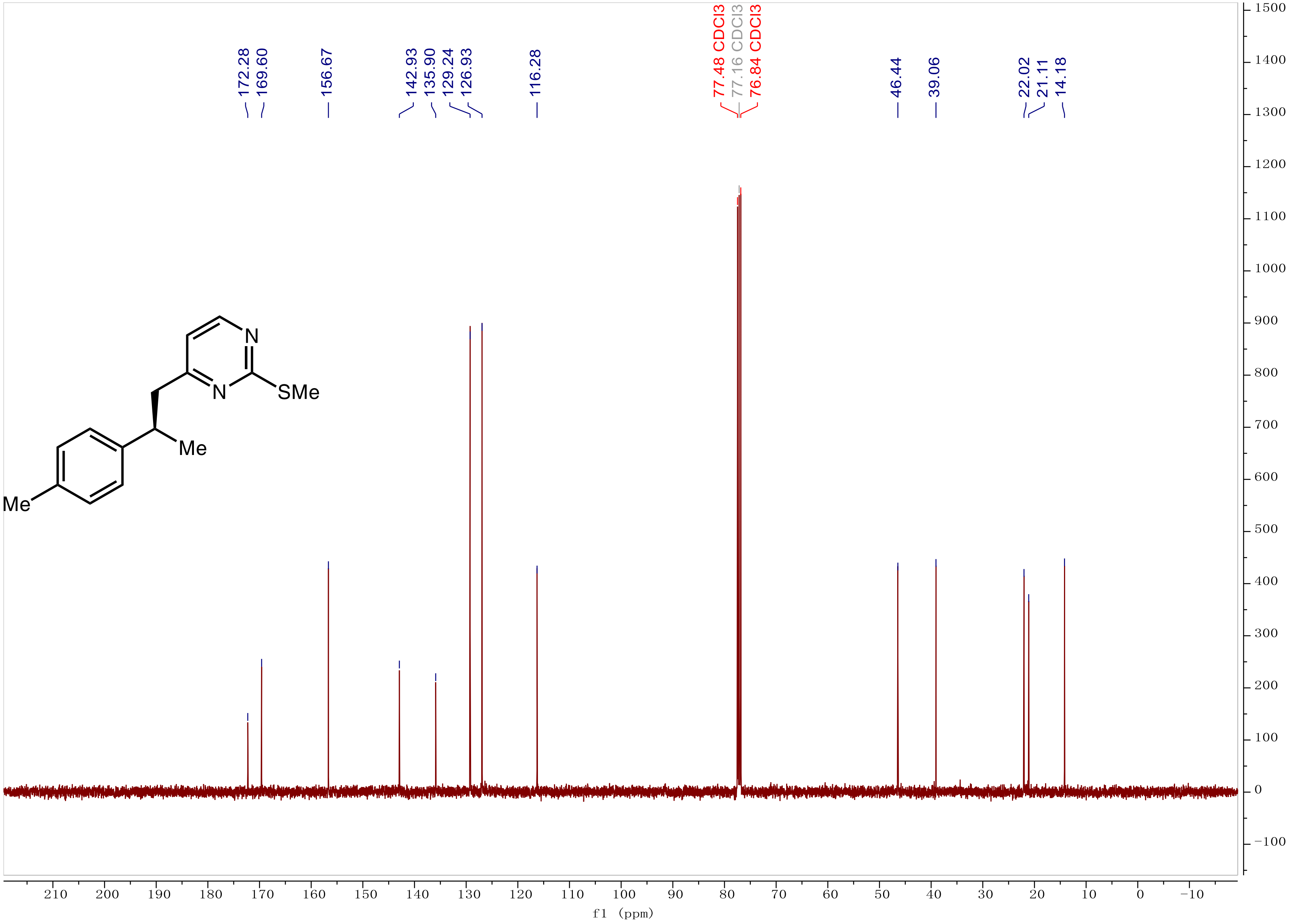

^{1}H NMR ($CDCl_3$, 400 MHz) spectrum of (*R*)-2-Methoxy-6-(2-phenylpropyl)pyridine (3w)

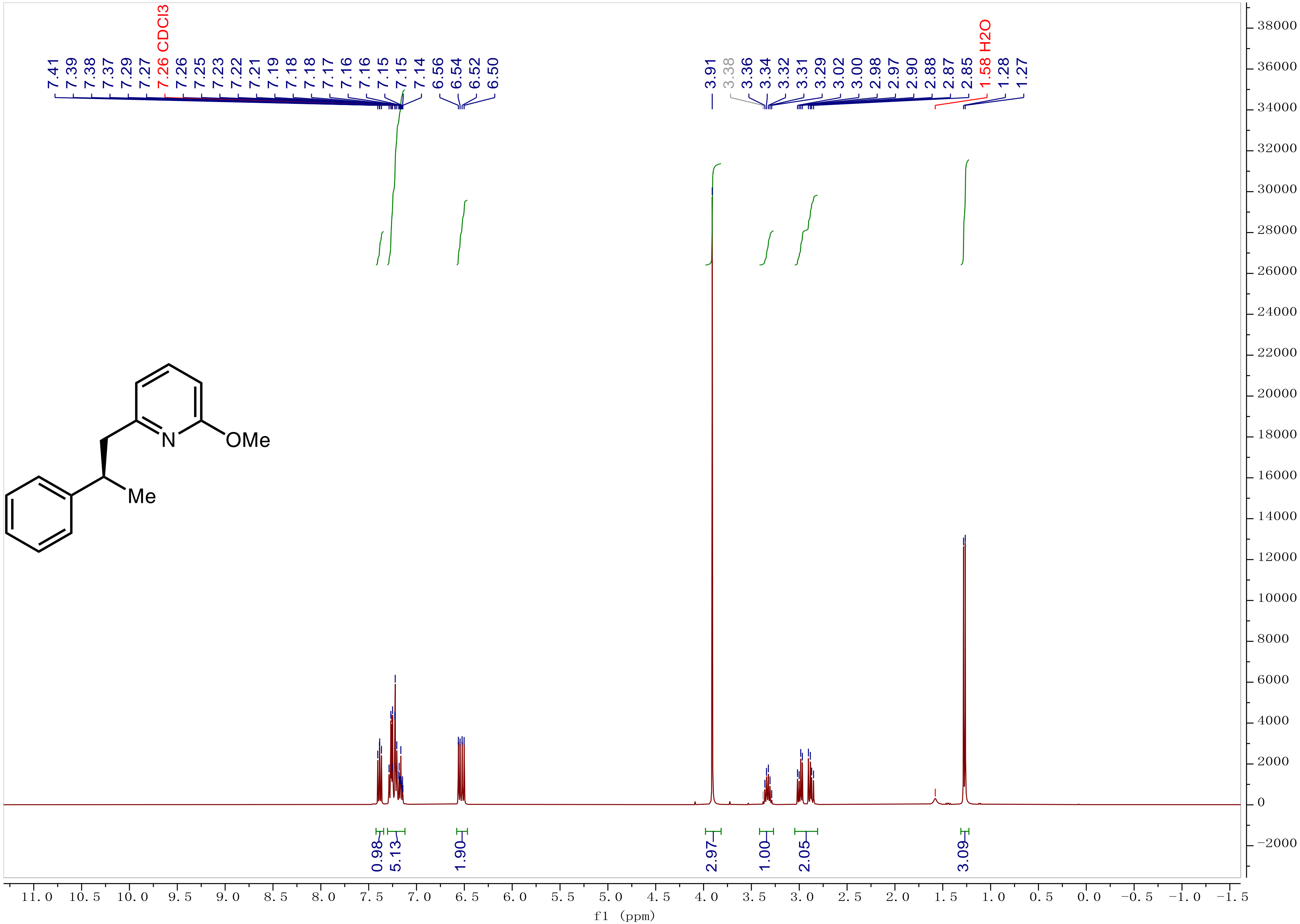

^{13}C NMR ($CDCl_3$, 100 MHz) spectrum of (*R*)-2-Methoxy-6-(2-phenylpropyl)pyridine (3w)

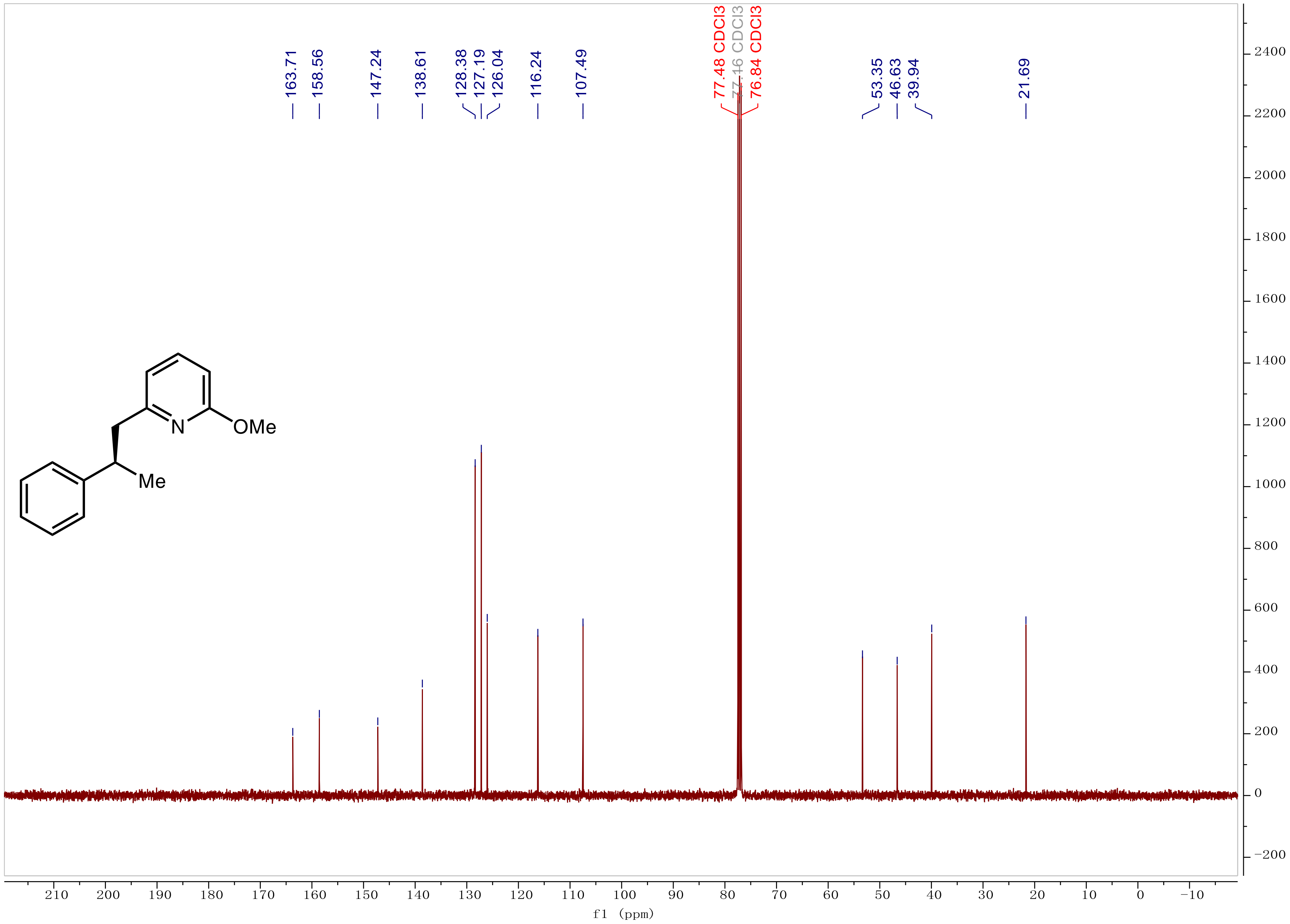

Supplement: Supplementary file 1 [file ANIE-65-e7251439-s001.pdf]
